# Exercise promotes lipophagy and mitigates cognitive impairment in Alzheimer’s disease through downregulating microglial TMEM9

**DOI:** 10.1186/s40035-026-00583-z

**Published:** 2026-10-19

**Authors:** Liying Zhang, Shiyin Li, Xiaomeng Ma, Shimei Cheng, Ziying Ye, Shuhua Liu, Luping Qin, Huimin Dong, Yifeng Feng, Mingyue Li, Jinghui Xu, Lei Cai, Xiquan Hu, Xiaofei He

**Affiliations:** 1 Department of Rehabilitation Medicine, The Third Affiliated Hospital Sun Yat-Sen University, 600 Tianhe Road, Guangzhou, 510630 China; 2 Department of Neurology, Mental and Neurological Diseases Research Center, The Third Affiliated Hospital Sun Yat-Sen University, Guangzhou, 510630 China; 3 Guangdong Provincial Biotechnology Research Institute (Guangdong Provincial Laboratory Animals Monitoring Center), Guangzhou, China; 4 Department of Nuclear Medicine, The Third Affiliated Hospital Sun Yat-Sen University, Guangzhou, 510630 China; 5 Department of Clinical Laboratory, The Third Affiliated Hospital Sun Yat-Sen University, Guangzhou, 510630 China

**Keywords:** Alzheimer’s disease, Physical exercise, Lipophagy, Microglia, TMEM9, ATP6AP1, LKB1

## Abstract

**Background:**

Autophagy-lysosomal dysfunction contributes to lipid droplet (LD) accumulation, which drives pathological progression in Alzheimer's disease (AD). Exercise is reported to restore autophagy-lysosomal function and attenuate cognitive impairment in AD, but its regulation of LD deposition remains unknown.

**Methods:**

5×FAD transgenic mice underwent a three-month exercise intervention. Cognitive and emotional behaviors were assessed using the Morris water maze, novel object recognition, and open field test. LD deposition was evaluated by oil red O and BODIPY staining. Proteomic sequencing was performed to identify proteins affected by exercise. Pathological changes were examined by immunofluorescence, western blotting, and transmission electron microscopy. Intracerebroventricular injection of AAV was used to overexpress or knock down microglial TMEM9 to investigate the mechanisms by which physical exercise affects behavior and pathology in AD. In vitro, BV2 cells were cultured and incubated with oligomeric amyloid-β (oAβ), with or without oleic acid to induce LD formation. Chloroquine and rapamycin, which regulate autophagy-lysosomal function, were used to examine the effects of LDs on mitochondrial integrity under glucose deprivation conditions. Lentiviral or plasmid transfection was used to explore the key role of TMEM9 in regulating lipophagy, and the interaction between TMEM9 and ATP6AP1 was detected by co-immunoprecipitation. Clinically, plasma TMEM9 and ATP6AP1 levels in AD patients were measured by ELISA, and receiver operating characteristic (ROC) analysis was performed to evaluate diagnostic performance.

**Results:**

Exercise mitigated cerebral LD deposition through enhanced lipophagy, preserving mitochondrial integrity and attenuating cognitive decline. Mechanistically, exercise suppressed microglial TMEM9 expression, restoring LD degradation. *Tmem9* downregulation facilitated LKB1 lysosomal translocation, activating AMPK signaling. In vitro, oleic acid-induced LDs preserved mitochondrial membrane potential and increased ATP production in BV2 microglia under glucose deprivation and oAβ exposure. *Tmem9* overexpression exacerbated lipophagy impairment and mitochondrial dysfunction, whereas *Tmem9* knockdown enhanced lipophagy and cytoprotection. Aβ clearance was negatively regulated by TMEM9. Clinically, AD patients exhibited characteristic Aβ deposition and glucose hypometabolism, with plasma TMEM9 levels strongly correlating with p-tau217 progression. Plasma TMEM9 and the TMEM9/ATP6AP1 ratio emerged as potential AD diagnostic biomarkers (AUC=0.791 and 0.853, respectively).

**Conclusion:**

These findings establish exercise-induced TMEM9 inhibition as a novel therapeutic strategy targeting lipidostasis to combat AD progression.

**Supplementary Information:**

The online version contains supplementary material available at https://doi.org/10.1186/s40035-026-00583-z.

## Introduction

Alzheimer’s disease (AD) is a neurodegenerative disorder characterized by progressive cognitive decline and memory loss, representing the most common cause of dementia [1]. Currently, more than 50 million individuals worldwide are affected by AD, which imposes a substantial socioeconomic burden [2]. The core neuropathological hallmarks of AD include extracellular β-amyloid (Aβ) plaque deposition and intracellular neurofibrillary tangles composed of hyperphosphorylated tau protein, accompanied by neuroinflammation, synaptic loss and mitochondrial dysfunction [3, 4]. Current therapies, such as cholinesterase inhibitors and NMDA antagonists, provide only symptomatic relief [5]. Anti-Aβ immunotherapies approved by Food and Drug Administration (FDA), including lecanemab and donanemab, have shown partial clinical efficacy but are associated with risks of side effects. There is an urgent need for precision therapies that target multifactorial mechanisms [6, 7].

Disruption of lipid metabolism has been proposed as a hallmark feature of immune dysfunction in myeloid cells [8]. In the AD brain, microglia with increased accumulation of lipid droplets (LDs) display a dysfunctional pro-inflammatory state and impaired phagocytic function, leading to reduced Aβ clearance efficiency and contributing to tau phosphorylation, thereby resulting in neurotoxicity [8]. Reduction of LDs significantly enhances the microglial phagocytic activity and decreases Aβ accumulation [9, 10]. However, as storage organelles at the center of lipid and energy homeostasis, LDs can sequester fatty acids and protect the cells from lipotoxicity [11]. More importantly, neutral lipids in the LD core can release free fatty acids, which are directed to mitochondria for fatty acid oxidation to fuel ATP production under conditions of energy deficiency [12]. Therefore, it remains unclear whether LDs in microglia play a benign, protective or damaging role in AD pathogenesis.

LDs are degraded through lipophagy, a form of selective autophagy. During lipophagy, LDs are specifically recognized by autophagy receptors and targeted to lysosomes for hydrolysis [12]. Impairment of lipophagy in microglia causes LDs accumulation and exacerbates neuroinflammation, contributing to cognitive decline [13]. Enhancement of autophagy facilitates the breakdown of LDs and reduces lipid accumulation, which is cytoprotective [14]. Nevertheless, the regulatory mechanisms underlying lipophagy in AD remain largely unexplored. A recent study proposed that lipotoxicity induces lysosomal dysfunction, which disrupts the autophagic flux and exacerbates the decline in mitochondrial function, subsequently resulting in LD accumulation and impaired mitochondrial function [14]. Besides, lysosome dysfunction arises early in AD, contributing to neuritic dystrophy, reduced clearance of Aβ, synaptic plasticity deficits, and neurodegeneration [15]. Restoring lysosomal functionality decreases the LD accumulation and improves cognition in AD [16, 17].

Physical exercise is recommended as a powerful physiologic intervention that protects against cognitive impairment in AD. It inhibits neuroinflammation, promotes Aβ clearance and ameliorates cognitive impairment [18]. Furthermore, exercise training is reported to reverse autophagy–lysosomal deficits and improve cognition in AD [18]. Recent studies indicate that exercise remodels the abdominal subcutaneous adipose tissue to attenuate inflammatory pathways and regulates the LD–mitochondria contacts, facilitating fatty acid transport from LDs to mitochondria [19–21]. In this study, we aimed to investigate the regulatory mechanisms through which physical exercise modulates LD deposition and explore potential biomarkers of the autophagy-lysosomal status in AD brains.

## Materials and methods

### Animals

5×FAD mice bred in Guangdong Laboratory Animal Monitoring Institute were sourced from the Jackson Laboratory (Cat # 034848, Bar Harbor, ME). Eight C57BL/6 J mice were regarded as wild type (WT), and 48 male 5×FAD mice (5 months old, 25–30 g) were used in this study. The mice were housed with free access to food and water, at controlled temperature and humidity under a 12:12 h light: dark cycle (light on during 07:00−19:00).The male 5×FAD mice were randomly assigned to control, physical exercise (designated as PE group), AAV-RNAi-*Tmem9*, PE+AAV-OE-*Tmem9*, AAV-Scramble, and PE+AAV-NC groups. Mice in the control and the RNAi-*Tmem9*-AAV groups were housed in a polypropylene cage of a regular size (36 cm length × 20 cm width × 14 cm height). Mice in the PE and the PE+OE-*Tmem9*-AAV groups were housed in a polypropylene cage of the same size, but equipped with a running wheel of 16-cm diameter. The mice were bred under these conditions for three months. All animal experimental procedures were designed in accordance with the ARRIVE guidelines and adhered to the rules stipulated by the animal ethics Committee, and no animals were excluded from the analysis. All animal experiments were approved by the Institutional Animal Care and Use Committee (IACUC) of Guangdong Laboratory Animal Monitoring Institute.

### Proteomic analysis

Following 3-month physical exercise, cortical tissues from control-5×FAD and PE-5×FAD groups (*n*=3/group) were homogenized in SDT buffer, sonicated, and centrifuged. Proteins were quantified using the bicinchoninic acid (BCA) assay (Beyotime, Shanghai, China), with 20 μg aliquots verified by SDS-PAGE. For filter-aided sample preparation (FASP) enzymolysis, proteins were reduced with DTT (Sigma, St. Louis, MO, 43819-5G). Subsequently, the detergent, DTT and other low-molecular-weight constitutes were removed using UA buffer (8 M Urea, 150 mM Tris–HCl, pH 8.5) through repeated ultrafiltration. Then, iodoacetamide (Sigma, I1149-5G) was added to block the reduced cysteine residues, and samples were incubated for 30 min in darkness. The filters were washed by UA buffer and NH_4_HCO_3_ (Sigma, A6141-25G) buffer. Finally, the protein suspension was digested with trypsin (Promega, Madison, WI, V5117) at 37 °C overnight, and the resultant peptides were collected as a filtrate. Peptide segments were desalted using C18 column (IonOpticks, Melbourne, Victoria, Australia). Peptide content was estimated by UV light spectral density at 280 nm. LC–MS/MS analysis was performed on an Orbitrap Exploris 480 mass spectrometer (Thermo Fisher Scientific, Waltham, MA) coupled to Easy nLC (Thermo Fisher Scientific). Data were processed using MaxOuant (version 1.6.17.0) at a false discovery rate (FDR) of < 0.01. Differentially expressed proteins were identified based on LFQ intensity normalization, applying a fold change threshold of |FC|> 1.2 and a *t*-test *P*-value < 0.05. Functional annotation was performed with Blast2GO to assign Gene Ontology (GO) terms, and enriched GO categories were determined using Fisher’s exact test.

### Intracerebroventricular injection of AAV

Male mice at five months old were used throughout the experiments. For specific knockdown of microglial transmembrane protein 9 (*Tmem9*), a recombinant adeno-associated virus serotype 9 (AAV9) vector containing siRNA targeting *Tmem9* (AAV9-*Tmem9*-RNAi) under the control of F4/80 promoter (AAV-F4/80p-EGFP-mir155siRNA (*Tmem9*)-SV40 PolyA) was constructed by Genechem (Shanghai, China) [22, 23]. The miR-155 scaffold was selected due to its well-documented efficacy and precision in mediating target gene knockdown in vivo [24]. The sequence of sh*Tmem9* was as follows: 5’-TGCCTATACTGAGCAGCTGCA-3’. To overexpress microglial *Tmem9*, *Tmem9* cDNA (NM_001160145.1) was obtained from the cDNA library of Genechem (Shanghai, China), and the 597-base-pair PCR product was cloned into a linearised AAV vector plasmid GV650: pAAV-F4/80p-MCS-EGFP-3Flag-SV40 PolyA (Genechem) with T4 DNA ligase and transfected into competent *Escherichia coli* cells. Positive clones were selected by ampicillin and then sequenced by ABI3730 sequencing analysis (Invitrogen, Shanghai, China). The *Tmem9* overexpression AAV (AAV-Tmem9) was packaged in HEK293T cells, concentrated and purified using 0.001% Pluronic F-68 solution (Poloxamer 188 Solution, PFL01-100ML, Caisson Laboratories, Smithfield, UT) and PEG 8000 (configured by Shanghai Genechem Co., Ltd.). The endpoint dilution method was used to determine the viral titer. In this construct, *Tmem9*, along with an enhanced green fluorescent protein (EGFP) reporter gene, was driven by the microglia-specific F4/80 promoter (AAV-F4/80-MCS-EGFP-3Flag-SV40 PolyA).

For injection, 5 μL of AAV9-*Tmem9*-RNAi (1 × 10^13^ genomic copies/mL) was injected intracerebroventricularly into the sedentary mice to knockdown the microglial *Tmem9*, which was designated as the RNAi-*Tmem9* group. Meanwhile, 5 μL of AAV-*Tmem9* (1 × 10^13^ genomic copies/mL) was intracerebroventricularly injected in the PE-5×FAD mice, which was designated as the PE+OE-*Tmem9* group. The injections were conducted one week prior to the initiation of physical exercise (Fig. 2a). Specifically, mice were anesthetized with 4% isoflurane in oxygen (flow rate: 0.8 L/min) for induction, followed by anesthesia maintenance with 2% isoflurane (flow rate: 0.4 L/min). Subsequently, animals were secured in a stereotaxic frame (RWD Life Science, Shenzhen, China), and the left lateral ventricle was targeted using the following coordinates relative to bregma: −0.5 mm anteroposterior (AP), +1.0 mm mediolateral (ML), and −2.5 mm dorsoventral (DV). A microsyringe containing 5 μL AAV liquid was slowly pumped into the lateral ventricles at 0.5 μL/min using a mini-pump controller (RWD Life Science, Shenzhen, China). Following a 10-min infusion period, the microsyringe was carefully withdrawn, and the scalp incision was sutured. Finally, the overexpression and downregulation of microglial TMEM9 were confirmed by Western blotting and immunofluorescent staining (Fig. S2). In addition, we injected control AAV to exclude the effects of AAV injection on the motor activity of animals. Specifically, AAV-Scramble (Cat. No. AAV9CON578, Genechem, sequence: 5’-TTCTCCGAACGTGTCACGT-3’) was injected as a control for AAV-RNAi-*Tmem9* and AAV-Negative control (AAV-NC, Cat. No. AAV9CON46l) as a viral vector-only control for AAV-*Tmem9* in the PE mice.

### Open field test

Open field test was performed to detect anxiety and exploratory activity [25]. Specifically, the apparatus comprised a 50 × 50 cm^2^ square arena with 40-cm high walls. A video camera was positioned directly above the arena. The mice were placed in the center of the arena, and their spontaneous locomotor activity over a 5-min period test was recorded. The video camera was used to track the trajectories of mice, and animal behavior analysis software (Xinxin Technology Co., LTD., Shanghai, China) was utilized to record the time spent in the center (region of interest, ROI).

### Novel object recognition

The novel object recognition was utilized to quantify the novelty preference index of mice after the open field test. Behavioral testing was performed in a rectangular apparatus (white polyethylene base, 40 cm^3^ inner volume; Xinxin Tech, Shanghai, China) with blue polystyrene walls. On the training day, two identical objects (cylindrical red blocks with 3-cm diameter) were fixed at the southeast and northwest corners, respectively. The mice were allowed 10 min for free exploration. On the testing day, the southeast object was replaced with a novel object (a square blue block with 3-cm diameter). The exploratory trajectories were digitally captured for 5 min using a camera positioned above the arena. The duration of nose-pointing of the novel and familiar objects (identified as nose tip within 2 cm from the object) was recorded. Novel object exploratory behavior was assessed as the discrimination index (DI), calculated as following: time exploring the novel object / (time exploring the familiar object + time exploring the novel object).

### Morris water maze

Morris water maze was performed to assess the spatial learning and memory of mice [26]. The apparatus consisted of a circular pool of 120-cm diameter and 50-cm height, with an overhead video camera and automated software (Xinxin Tech). The pool was divided into four quadrants, and mice were introduced into the pool from the midpoint of the wall of each quadrant. A white circular platform with a diameter of 10 cm was positioned 1 cm beneath the water surface in the center of a quadrant. During training, each mouse was placed into the water facing the wall. The swimming paths and the latency to locate the platform were recorded. Mice that failed to find the platform within 60 s were manually guided to the platform and remained there for 10 s. Training was performed four trials per day for five consecutive days. On day 6, a 60-s probe trial was performed with the platform removed. The swimming speed and the number of crossings over the target area were recorded and analyzed.

### Immunofluorescence staining

Mice were deeply anesthetized with 1% sodium pentobarbital (P-010, Macklin, Shanghai, China), then perfused transcardially with 0.9% physiological saline (HH02001-1, BIOFIVEN, Guangzhou, China) and 4% (*w*/*v*) paraformaldehyde (PFA) (DF0135-500, Biosharp, Hefei, China). The brains were removed and fixed in 4% PFA overnight, followed by gradual dehydration with 20% and 30% sucrose (SJ-T6-01-01DM, Damao, Tianjin, China) until complete precipitation. Subsequently, the samples were embedded with optimal cutting temperature compound (4583, SAKURA, Tokyo, Japan), and brain sections from bregma −1.7 mm to −2.4 mm were cut coronally at a thickness of 10 µm using a cryostat microtome (Leica CM 1950; Leica Biosystem, Heidelberg, Germany). The samples were stored at −80 ℃ for later experiments. For immunofluorescent staining, the sections were microwaved in citrate buffer (pH 6.0) (BL619A, Biosharp) for 5 min, and then cooled and incubated with immunostaining blocking buffer (P0102, Beyotime) at room temperature for 1 h. The sections were then incubated with primary antibodies overnight at 4 °C, followed by incubation with secondary antibodies (Table S1). Finally, the sections were mounted using DAPI-containing Fluoroshield™ (F6057, Sigma). Images of the right cerebral cortex and/or dentate gyrus (DG) were captured using a confocal microscope (Leica, DM6000, Germany). ROIs were selected based on anatomical landmarks according to the mouse brain atlas. For the hippocampus, the DG was delineated by its distinct cellular layer morphology under DAPI staining. For the cerebral cortex, the parietal cortex was selected based on its location dorsal to the hippocampus. For each animal, four sections at intervals of 100 μm (three fields per section) were selected for analysis using the ImageJ software (National Institutes of Health, Bethesda, MD).

### Western blots

Total protein was extracted with lysis buffer and quantified using the BCA Protein Assay Kit (P0012, Beyotime Biotechnology). Lysosomal protein extractions were performed with the Lysosomal Protein Extraction Kit (EX1230, Solarbio, Beijing, China) according to the manufacturer’s instructions. Proteins (20 µg/lane) were loaded in sodium dodecyl sulfate polyacrylamide gels, segregated by electrophoresis for 90 min at 120 V, and transferred to the polyvinylidene difluoride membrane (ISEQ00010, Millipore, Darmstadt, Germany) for 2 h at 100 V. Subsequently, the membrane was blocked in 5% skim milk (1172GR500, Biofroxx, Einhausen, Germany) or BSA (10735108001, Roche, Basel, Switzerland) for 1 h. Then the membrane was incubated at 4 °C overnight with primary antibodies, followed by incubation with secondary antibodies for 1 h (Table S1). At last, protein bands were detected by the ECL blotting kit (SQ202L, Epizyme, Shanghai, China) and visualized by the chemiluminescence imaging system (ProteinSimple, Santa Clara, CA). Gray scale values of the protein bands were analyzed using ImageJ software.

### Transmission electron microscope (TEM)

The mice were deeply anesthetized and intracardial perfusion with 40 mL of pre-cooled 0.9% sodium chloride. Cortical samples were collected and preserved overnight in 2% PFA and 2.5% glutaraldehyde (G6257, Sigma) in 0.1 M phosphate buffer solution (PBS) (pH 7.4) (G0002-2L, Servicebio, Wuhan, China). The samples were rinsed 3 times with 0.1 M PBS (10 min each time), and post-fixed with 1% osmic anhydride (SPI-Pon™ 812, Structure Probe, Inc., West Chester, PA) at 4 °C for one hour. Then the samples were rinsed with 0.1 M PBS (15 min per wash) and dehydrated in ascending ethanol solutions (50%, 70%, 80%, and 90% for 10 min; 100% for 10 min 2 times). Then they were dehydrated in acetylacetone (A800842, Macklin), and gradually embedded in a mixture of acetylacetone and EPON812 (45345, Macklin) (ratios of 3:1 for 0.5 h, then 1:1 for 4 h at room temperature), and finally embedded in pure EPON812 overnight. The samples were cured in a 60 °C oven for 48 h, then cut into 100 nm ultra-thin sections using an ultramicrotome (Leica UC7). The sections were stained with 1% uranyl acetate for 20 min, and subsequently with lead citrate for 12 min. Images were obtained using a Tecnai Spirit transmission electron microscope (FEI, Hillsboro, OR). Microglia were identified by their characteristic ultrastructural morphology under TEM: nuclei are small, typically oval or slightly elongated. Furthermore, the microglial nuclei display a comparatively higher proportion of heterochromatin relative to other neural cell types; dense, electron-dense heterochromatin aggregates are prominently observed in peripheral regions adjacent to the nuclear membrane [27, 28].

### Oil red O staining

Slices were stained by oil red O to assess LD accumulation in the brains. Specifically, a saturated oil red O solution (Servicebio, G1015) was mixed with distilled water at a ratio of 3:2 (*v*:*v*). After filtration, the frozen slices were stained in the diluted oil red O working solution at room temperature for 8–10 min in darkness. Subsequently, the slices were rinsed in 60% isopropanol-water solution (80109218, Sinopharm Chemical Reagent Co., Ltd, Shanghai, China) for 3 s and 5 s, and in distilled water for 2×10 s. After hematoxylin (G1004, Servicebio) re-staining for 3–5 min, the slices were sealed with glycerogelatin (G1402, Servicebio) and photographed by a microscope system (Leica, Germany). The lipid deposition in tissue was quantified by using the ImageJ software.

### Cell culture and treatments

Murine microglia cell line BV2 was obtained from Shanghai Cell Research Center (Shanghai, China). Cells were cultured in Dulbecco’s Modified Eagle’s Medium (DMEM) (C11995500BT, Gibco, Waltham, MA) in a 5% CO_2_ atmosphere, with 10% (*v*/*v*) fetal bovine serum (FBS) (10099–141, Gibco) and 1% penicillin/ streptomycin (15140122, Gibco) at 37 °C. The cells were seeded into different well plates or poly-L-lysine-coated glass coverslips (3×10^5^ cells per well for six-well plate and 3×10^4^ per well for 48-well plate) for western blots or immunofluorescent staining. After cell attachment, the cell culture medium was replaced with DMEM containing 10% FBS and 25 mM glucose with 500 μM oleic acid (T2O2668, TargetMol, Boston, MA) for 24 h for LD accumulations, and then cultured in DMEM containing 10% FBS and 5 mM glucose with 5 μM Aβ oligomers (oAβ) for 24 h. Then, the cells were fixed or collected for further analysis. For drug treatment, cells were pretreated with Compound C (5 μM, HY-13418A, MedChemExpress, NJ) or concanamycin A (1 μM, GC17519, GLPBIO, Montclair, CA) for 1 h before incubation with oAβ for 24 h to block AMP-activated protein kinase (AMPK) phosphorylation and V-ATPase activity, respectively. Chloroquine (CQ, 20 μM, TargetMol, T8689) and rapamycin (100 nM, TargetMol, T1537) were added simultaneously with oAβ to block or activate the autophagic process.

### Aβ_42_ oligomer preparation

Briefly, the Aβ_42_ peptides (52487, Glbiochem, Shanghai, China) treated with HFIP (hexafluoroisopropanol) (H811027, Macklin) were re-suspended in dimethyl sulfoxide (DMSO) (0219605590, MP Biomedicals, Irvine, CA), and subsequently diluted to 500 μM in Ham’s F12 (Pm150813, Procell, Wuhan, China). After incubation at 4 °C for 24 h, soluble oAβ was acquired from the supernatant by centrifuging at 14,000 g for 10 min.

### Lentivirus transfection

Lentiviral reagents containing negative control (Catalog #: LPP-NEG-Lv201-050), *Tmem9*-cDNA (Catalog #: LPP-Mm08429-Lv201-100), scramble control (Catalog #: LPP-CSHCTR001-LVRU6GP) and *Tmem9*-ShRNA (Catalog #: LPP-MSH033519-LVRU6GP-f-200) were procured from Guangzhou iGenebio Biotechnology Co., Ltd (Guangzhou, China). Mouse BV2 microglia were transfected with these lentiviruses according to manufacturer’s instructions and previous literature [23].

### Adenosine triphosphate (ATP) assay

Intracellular ATP levels were quantified using the Beyotime ATP Detection Kit (Catalog No. S0026) according to the manufacturer’s instructions. Upon reaching 80%–90% confluence, cells were harvested, and lysed with the provided lysis buffer on ice. The lysate was centrifuged at 12,000× *g* for 5 min on ice to collect the supernatant. A series of ATP standard solutions were prepared, and the luminescent reaction was initiated by mixing the lysate with the ATP detection reagent according to the kit protocol. RLU values were measured using a luminometer. ATP concentrations in the samples were determined from the standard curve and expressed as relative levels. Protein concentrations of samples were determined using the BCA protein assay kit (P0012S, Beyotime), and ATP concentrations were normalized to protein content (nmol/mg protein).

### Plasmid construction and cell transfection

The *Mus musculus* *Tmem9* gene coding sequence was ligated into the pEZ-Lv242 vector (EX-Mm08429-Lv242; GeneCopoeia, Inc., Rockville, MD) to construct the *Tmem9*-overexpression (OE) plasmid. Lv242 empty vectors were used as negative controls (NC). MSH102359-LVRUP-a was used as *Tmem9*-knockdown plasmid (ShRNA) while CSHCTR001-LVRU6P was used as control (Scramble).

BV2 cells were seeded into 24-well plates and transiently transfected with the plasmid and their respective controls via Opti-MEM I and Invitrogen™ Lipofectamine™ 3000 Transfection Reagent (L3000015, Life Technologies, Carlsbad, CA) when they reached approximately 80% confluence. Then the transfection medium was replaced with high-glucose DMEM containing 10% FBS after 12 h. All transiently transfected cells were harvested after 48 h. Transfection efficiency was evaluated by Western blots.

### Co-Immunoprecipitation

To investigate the interaction between TMEM9 and ATP6AP1 in NC-flag-plasmid (EX-NEG-Lv242) and OE-*Tmem9*-flag-plasmid (EX-Mm08429-Lv242) BV2 microglial cells, co-immunoprecipitation was performed. Cells were harvested and lysed in IP buffer (Thermo Scientific™, 87787) for 30 min on ice. The lysate was centrifuged at 13,000× *g* for 10 min to pellet the cell debris at 4 °C, and the supernatant was incubated with anti-flag antibodies (1:200, T0003, Affinity Biosciences, Jiangsu, China) overnight at 4 °C. Protein A/G agarose beads (EA-IP-007 M, Elabscience, Wuhan, Chian) were then added to the mixture and incubated for an additional 2 h at 4 °C to capture the antibody-antigen complexes. The beads were washed three times with PBST buffer, and the bound proteins were eluted by adding PBS and 5× loading buffer and heating at 95 °C for 5 min. The eluted proteins were analyzed by Western blots using specific antibodies against ATP6AP1 to confirm their interaction.

### Assessment of mitochondrial quality control

The fluorescent dye tetramethylrhodamine ethyl ester (TMRE) (HY-D0985A, Storage concentration of 5 mM, MCE, NJ) was used to measure changes in mitochondrial membrane potential (ΔΨm). Cells were incubated with TMRE (final concentration of 1 μM) for 30 min at 37 °C. After incubation, cells were washed twice with PBS to remove excess dye and subsequently captured under a confocal microscope (Leica, DM6000, Germany). The mean densities in ROIs were measured by ImageJ software, and normalized to the control group.

To evaluate mitochondrial oxidative stress in BV2 microglial cells, dihydroethidium (DHE) (MCE, HY-D0079, Storage concentration of 5 mM) fluorescence was utilized. Cells were incubated with DHE (final concentration 5 µM) for 30 min at 37 °C in the dark, washed twice with PBS to remove excess dye, and fixed with 4% PFA for 10 min. The fluorescence intensity of DHE was captured using a confocal microscope (Leica, DM6000, Germany).

For in vitro TMRE and DHE assays, signals were normalized to cell density (number of DAPI-stained nuclei per well), and fluorescence intensity was quantified using ImageJ software.

### Reverse-transcription quantitative polymerase chain reaction (RT-qPCR)

Total RNA was extracted from cultured cells using the RNA-Quick Purification Kit (RN001, ES Science, Shanghai, China) according to the manufacturer’s protocol. Briefly, cells were washed twice with ice-cold PBS and lysed directly in the culture dish using lysis buffer provided in the kit. RNA concentration and purity were measured with a NanoDrop One Microvolume UV–Vis Spectrophotometer (Thermo Scientific, Bremen, Germany). Reverse transcription was performed using the *Evo M-MLV* Reverse Transcription Premix Kit (with gDNA removal reagent for qPCR) (AG11728, Accurate Biology, Changsha, China) following the manufacturer’s instructions. Real-time RT-qPCR was carried out in a total reaction volume of 10 μL, containing 2 ng of cDNA, 0.2 μM of each forward and reverse primer, and the SYBR Green Premix Pro Taq HS qPCR (AG11701, Accurate Biology). Amplification was conducted on a Roche LightCycler96 Real-Time PCR System (Roche). The thermal cycling program consisted of an initial denaturation at 95 °C for 30 s, followed by 40 cycles of 95 °C for 3 s and 60 °C for 30 s. Relative mRNA expression were calculated using the 2^−ΔΔCT^ method, with β-actin serving as the endogenous control. Primers were synthesized by Tsingke Biotechnology (Beijing, China), and their sequences are as follows: *β-actin*-F: GTGACGTTGACATCCGTAAAGA, *β-actin*-R: GCCGGACTCATCGTACTCC; *Tmem9*-F: GTCCGCCTTACAGAAACATCA, *Tmem9*-R: CTCGTACCTACACTCGCAGAG.

### Participants

This clinical trial was registered in the Chinese Clinical Trial Registry (registration number: ChiCTR2400085313, registration data: June 5, 2024), and this study was approved by the Ethics Committee of the Third Affiliated Hospital of Sun Yat-sen University (Approval Number: SL-RG2024-082-03) according to the guidelines of the Helsinki declaration, and written informed consent was obtained from all participants. AD was diagnosed according to the 2024 Alzheimer’s Association criteria [29]. In this study, 39 AD participants (17 males, 22 females) and 42 age-matched controls (19 males, 23 females) were recruited. Plasma samples were collected from 28 controls and 26 AD patients, and plasma phosphorylated tau 217 (p-tau217) levels of 25 AD patients (11 males, 14 females) were measured by enzyme linked immunosorbent assay (ELISA). Cerebrospinal fluid (CSF) samples were collected from 6 controls and 16 AD patients, and CSF p-tau181 levels of 15 AD patients (7 male, 8 female) were measured by ELISA. Nine controls and 9 AD patients underwent ^18^F-AV45 PET-CT; 4 controls and 8 AD patients underwent ^18^F-FDG PET-CT. Among these, 4 AD patients simultaneously underwent plasma TMEM9, ATP6AP1, and p-tau217 ELISA measurement and ^18^F-FDG PET-CT scanning; 6 AD patients simultaneously underwent plasma TMEM9, ATP6AP1, and p-tau217 ELISA measurement and ^18^F-AV45 PET-CT scanning; 4 AD patients simultaneously underwent CSF TMEM9, ATP6AP1, and p-tau181 ELISA measurement and ^18^F-AV45 PET-CT scanning; 4 AD patients simultaneously underwent CSF TMEM9, ATP6AP1, and p-tau181 ELISA measurement and ^18^F-FDG PET-CT scanning; and 5 AD patients simultaneously underwent ^18^F-AV45 PET-CT and ^18^F-FDG PET-CT scanning.

### ELISA

Concentrations of p-tau217 in plasma and p-tau181 in the CSF were detected by ELISA kits according to the manufacturer’s instructions (MM-62576H1 & MM-63615H1, Jiangsu Meimian Industrial Co., Ltd, Wuxi, China). The remaining plasma and CSF were stored at −80 °C for further analysis of TMEM9 and ATP6AP1 levels. The TMEM9 and ATP6AP1 concentrations in plasma and CSF were examined using ELISA kits according to the manufacturer’s instructions (MM-65258H1 & MM-64851H1, Jiangsu Meimian Industrial Co., Ltd). Specifically, whole blood from patients was collected in the anticoagulant-treated tubes containing EDTA. The samples were centrifugated at 4 ℃ for 10 min (3000 rpm/min), and the supernatants were collected and transferred into a new tube for ELISA. CSF was collected and centrifugated at 4 ℃ for 10 min (3000 rpm/min) and the supernatants were collected and transferred into a new tube for ELISA. Then 10 μL of sample and 40 μL of sample diluent were added into a 96-well plate containing purified human TMEM9 or ATP6AP1 antibody, and incubated at 37℃ for 30 min. After five washes, HRP-conjugate reagent was added and incubated for 30 min at 37 ℃. Then, chromogen solution was added and incubated for 10 min at 37℃ and stop solution was added to terminate the reaction. Absorbance was measured using a microtiter plate reader (BioTek Synergy H1, Winooski, VT) at a wavelength of 450 nm.

### Brain PET-CT imaging protocol and analysis

Brain PET-CT imaging was performed using a GE Discovery Elite scanner, covering the skull vertex to base. For ^18^F-FDG PET, patients were fasted for 4–6 h before receiving 125–740 MBq of tracer, followed by a 60-min uptake period and 20-min scan [30]. ^18^F-AV45 PET (conducted the next day) involved injecting 185–370 MBq of ^18^F-AV45, followed 50 min later by scanning. Images were reconstructed using CT-based attenuation correction and 30% adaptive statistical iterative reconstruction (ASiR) with VUE Point FX. Analysis included visual assessment of cortical versus white matter uptake (focusing on temporal, frontal, posterior cingulate/precuneus, and parietal regions in suspected AD) and semi-quantitative standardized uptake value ratio (SUVr) calculations. The SUVr was calculated as the mean SUV in predefined cortical ROIs divided by the mean SUV in the cerebellar gray matter, which served as the reference region [31].

### Statistical analysis

All data were analyzed by a researcher blinded to the group allocation. Immunofluorescence and Western blotting results were analyzed by ImageJ. Statistical analysis was performed using GraphPad Prism 8.0. Data of the escape latency in the water maze training were analyzed with repeated measures two-way analysis of variance, followed by Tukey’s post-hoc multiple comparison tests. Other data were analyzed with one-way analysis of variance (ANOVA), followed by Tukey’s post-hoc multiple comparison tests. All data are expressed as mean ± SD, and *P* < 0.05 was considered as statistically significant. Associations between biomarkers were analyzed with linear regressions or Spearman rank correlation analysis.

## Results

### Physical exercise inhibits LD accumulation in microglia by down-regulating TMEM9 expression

**Fig. 1:**
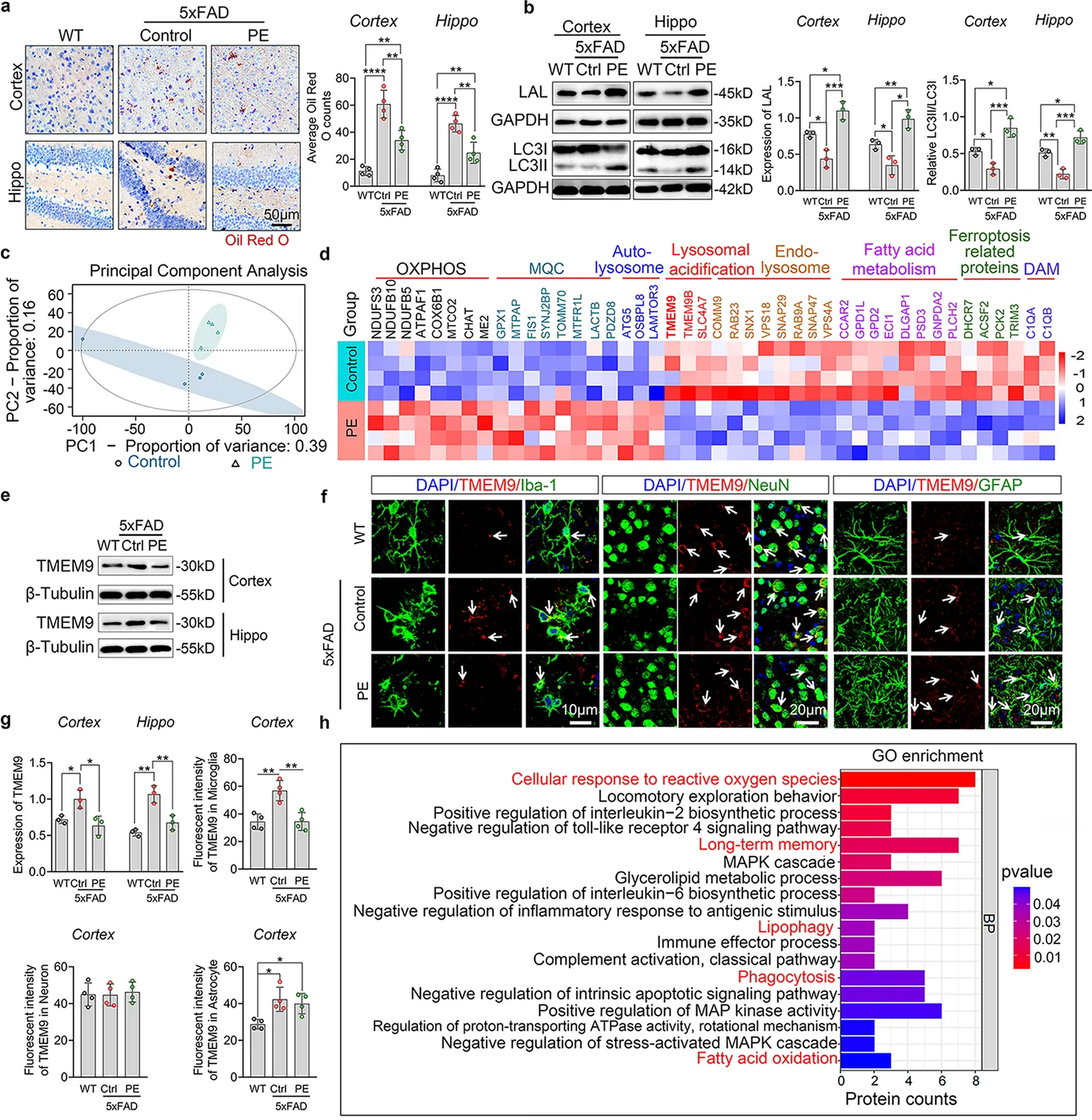
Physical exercise reduces lipid droplet (LD) accumulation and downregulates microglial TMEM9 in 5×FAD mice. **a** Representative Oil Red O staining images and quantitative analysis of LD accumulation in WT, Control-5×FAD (Ctrl), and 5×FAD with physical exercise (PE), *n* = 4 male mice per experimental group, 4 sections per mice, 3 fields per section, aged 8 months. **b** Western blots analysis of lysosomal acid lipase (LAL) and LC3II/LC3I ratio, *n* = 3 male mice per experimental group, aged 8 months. **c** Principal component analysis (PCA) plot depicting the global distribution of differentially expressed proteins (DEPs) in control- and PE-treated 5×FAD mice. **d** Heatmap visualization of DEPs in the right cerebral cortex and hippocampus of PE-treated versus control 5×FAD mice, *n* = 3 male mice per experimental group, aged 8 months. **e** Representative western blotting images of TMEM9 protein in the three experimental groups. **f** Immunofluorescence images showing TMEM9 in microglia, astrocytes and neurons. **g** Statistical analysis of western blots in **e** (*n* = 3 male mice per experimental group, aged 8 months) and immunofluorescence images in **f** (*n* = 4 male mice per experimental group, aged 8 months). **h** Gene Ontology (GO) enrichment analysis of DEPs in biological processes identified in **d**. Data are expressed as the mean±SD. One-way analysis of variance (ANOVA), followed by Tukey’s multiple comparisons test. *P < 0.05; **P < 0.01; ***P < 0.001; ****P < 0.0001

We measured the accumulation of intracellular LDs using Oil Red O, a dye for neutral lipids. The 5×FAD brain showed an abundance of perinuclear Oil Red O^+^ lipid bodies, which are similar to the original description of adipose saccules in glial cells of postmortem brain tissue of AD patients [9]. Surprisingly, physical exercise decreased the LD accumulation in 5×FAD mice (Fig. 1a). We next examined cellular distribution of LDs by immunostaining using antibodies for perilipin-2 (a major LD protein), microglial marker Iba-1, mature astrocyte marker GFAP (glial fibrillary acidic protein), and neuronal marker NeuN. As expected, we observed a remarkably increased accumulation of LDs in microglia in 5×FAD mice, which was decreased by exercise (Fig. S1a). However, the perilipin-2-positive LDs were not seen in astrocytes or neurons of 5×FAD (Ctrl-5×FAD) mouse brain (Fig. S1b). Surprisingly, physical exercise did not affect the expressions of CD36 (Fig. S1c), a membrane-localized fatty acid translocase for the entrance of fatty acids in the microglia [32]. This indicated that physical exercise did not affect the uptake of fatty acids, but may decrease the LDs via promoting LD degradation. We then detected the lipase including ATGL (adipose triglyceride lipase) and HSL (hormone-sensitive lipase), which were also not significantly different among the WT, Control- and PE-5×FAD groups (Fig. S1d). However, the level of lysosomal acid lipase (LAL), which is involved in lipophagy, was obviously decreased in Ctrl-5×FAD mice than in WT mice, and was up-regulated in PE-5×FAD compared with the Ctrl-5×FAD group (Fig. 1b). Besides, the microtubule-associated protein A/B-light chain 3 II/light chain 3 I (LC3II/LC3I) ratio was also decreased in Ctrl-5×FAD mice compared with WT mice, which was remarkably increased in the PE-5×FAD mice, in both cortex and hippocampus (Fig. 1b). All together, these results indicated that physical exercise decreased LDs in microglia via promoting lipophagy.

To investigate the mechanism by which physical exercise regulates microglial lipophagy, we performed proteomics to screen for differentially expressed proteins between 5×FAD mice with and without physical exercise. Differentially expressed proteins were initially selected using unadjusted *P* < 0.05 to maximize sensitivity, given the hypothesis-generating nature of the analysis. Physical exercise up-regulated the proteins involved in autophagy (ATG5, OSBPL8, LAMTOR3, ERLIN1), oxidative phosphorylation (OXPHOS) (ATPAF1, NDUFB5, NDUFB10, ME2), mitochondrial quality control (GPX1, MTPAP, TOMM70, PDZD8, LACTB, MTFR1L) and mitochondrial fragmentation (FIS1, SYNJ2BP). It also down-regulated the proteins involved in the regulation of lysosomal acidification (TMEM9, TMEM9B, SLC4A7), the endosome-lysosomal pathway (COMMD9, RAB23, SNX1, VPS18, SNAP29, RAB9A, SNAP47, VPS4A), fatty acid metabolism (CCAR2, GPD1L, GPD2, EC1, DLGAP1, PSD3, GNPDA2, PLCH2, DHCR7), ferroptosis (ACSF2, PCK2, TRIM3) and microglial activation (C1QA, C1QB) (Fig. 1c, d, Additional file 2). Among these down-regulated proteins, TMEM9 attracted our attention due to its critical role in regulating v-ATPase assembly for vesicular acidification and lysosomal protein degradation [33], as well as its essential function in autophagy [34]. Western blots verified the higher protein levels of TMEM9 in 5×FAD mice than in WT mice, which were down-regulated by physical exercise, in both cortex and hippocampus (Fig. 1e, g). Immunofluorescence staining showed that the TMEM9 down-regulation by PE was mainly localized in microglia (Fig. 1f, g). The TMEM9 intensity in neurons was not significantly different among WT, control- and PE-5×FAD mouse brains. The astrocytic TMEM9 was increased in 5×FAD mice compared with WT, which was not affected by exercise (Fig. 1f, g). GO enrichment analysis revealed enrichment of the differential proteins in biological process including cellular response to reactive oxygen species, long-term memory, lipophagy, phagocytosis and fatty acid oxidation (Fig. 1h).

### Physical exercise promotes microglial lipophagy and decreases LD accumulation by down-regulating TMEM9 in AD mouse brain

**Fig. 2:**
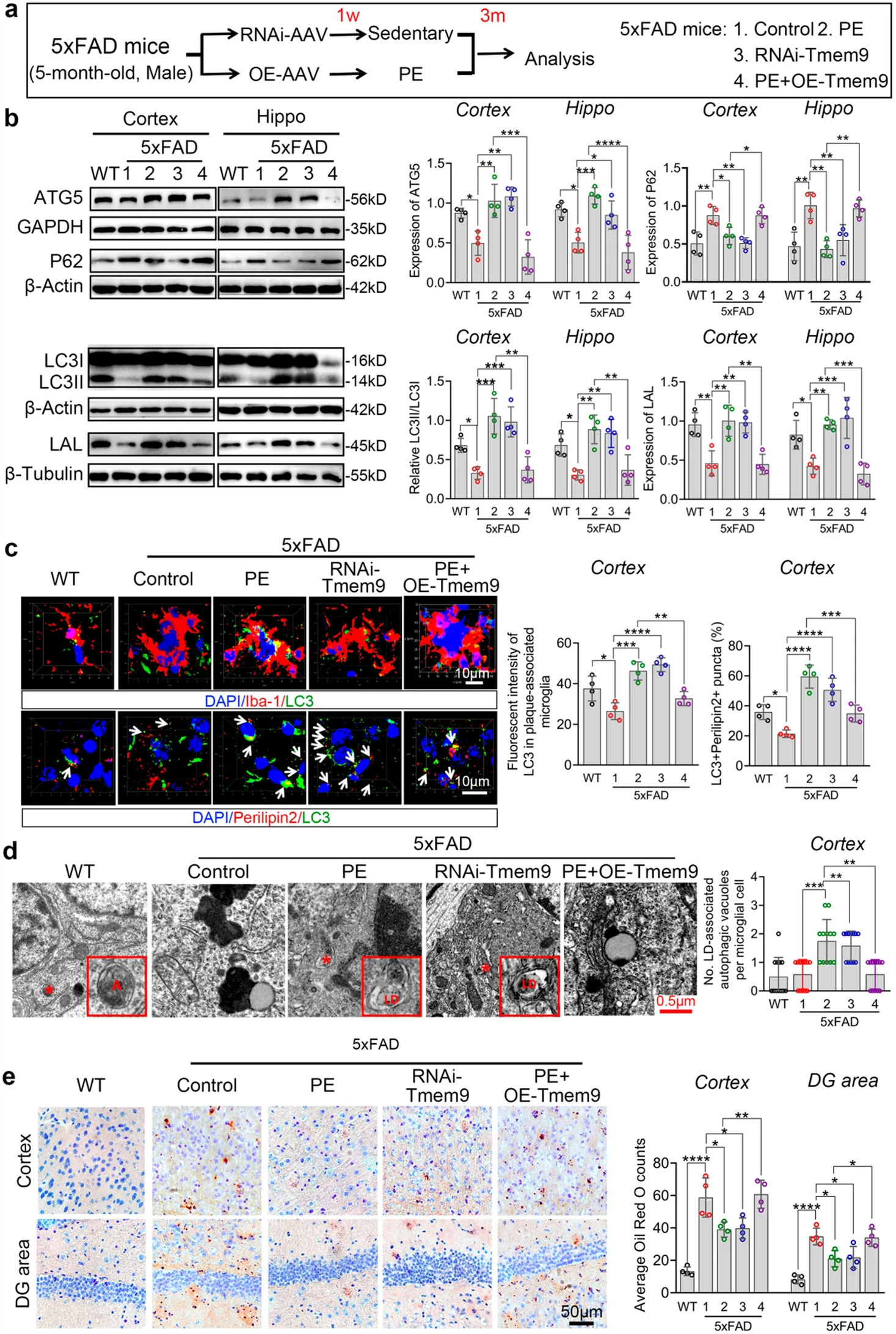
Physical exercise promotes microglial lipophagy and decreases LD accumulation by down-regulating TMEM9. **a** Experimental timeline illustrating adeno-associated virus (AAV) delivery (RNAi-*Tmem9* or OE-*Tmem9*), physical exercise intervention (PE), and analysis in WT and 5×FAD mice. **b** Western blots of ATG5, P62, LC3II/LC3I and LAL expression in WT, Ctrl-5×FAD, PE-5×FAD, RNAi-*Tmem9* and PE+OE-*Tmem9* groups, *n* = 4 male mice per experimental group, aged 8 months. **c** Representative immunofluorescent images and average flourescence intensity of LC3, as well as LC3^+^ Perilipin2^+^ puncta in microglia across experimental groups, *n* =4 male mice per experimental group, aged 8 months. **d** Transmission electron microscopy analysis of cortical tissues from experimental mice reveals the fusion of autophagosomes (A) with lipid droplets (LD) in microglia. *n*=4 cells per mouse, 3 mice per group. Asterisks mark autolysosomes undergoing hydrolysis, with a magnified view shown in the red box. **e** Oil Red O staining and quantification of LD accumulation in brain tissues, *n* = 4 male mice per experimental group, aged 8 months. Data are expressed as the mean ± SD. One-way analysis of variance (ANOVA), followed by Tukey’s multiple comparisons test. *P < 0.05, **P < 0.01, ***P < 0.001, ****P < 0.0001

To further investigate the role of the down-regulated TMEM9 in physical exercise-related microglial lipophagy, we administered AAVs into the brains of mice with physical exercise, to overexpress microglial *Tmem9* (designated as the PE+OE-*Tmem9*-AAV group). Additionally, we injected *Tmem9* RNAi-AAV with a F4/80 promoter into the brains of 5×FAD mice (designated as the RNAi-*Tmem9* group) (Fig. 2a). Western blots and immunofluorescence staining confirmed the downregulation and upregulation of TMEM9 protein levels in the RNAi-*Tmem9 *and PE+OE-*Tmem9*-AAV groups, respectively (Fig. S2). We next detected autophagy-associated proteins involved in lipophagy and lysosomal lipolysis, including ATG5, SQSTM1/P62, LC3 and lysosomal LAL (Fig. 2b). As anticipated, the protein level of ATG5, LC3II/LC3I ratio, and LAL protein level were significantly reduced in the Ctrl-5×FAD group compared to the WT group in both cortex and hippocampus. Physical exercise and RNAi-*Tmem9* treatment markedly increased these autophagy-associated proteins. However, OE-*Tmem9*-AAV injection notably decreased their protein levels in PE-5×FAD mice. In addition, the protein level of P62 was significantly increased in the Ctrl-5×FAD group compared to the WT group in both cortex and hippocampus, while physical exercise and RNAi-*Tmem9 *treatments markedly decreased the P62 level. OE-*Tmem9*-AAV injection notably increased the P62 protein level in PE-5×FAD mice (Fig. 2b).

Furthermore, the pixel intensity of LC3 and the pixel ratio of perilipin-2 co-localized with LC3 in plaque-associated microglia were significantly decreased in the 5×FAD mice compared to the WT. In contrast, physical exercise or *Tmem9*-RNAi treatments markedly increased both LC3B intensity and the ratio of perilipin-2 co-localized with LC3B. However, this upregulation in PE-5×FAD mice was notably inhibited by OE-*Tmem9*-AAV injection (Fig. 2c). Besides, co-staining for Iba1/LC3/Bodipy in vivo showed that the Bodipy fluorescence intensity was significantly increased in the 5×FAD group compared to the WT. However, PE or *Tmem9*-RNAi treatment markedly decreased the Bodipy fluorescence intensity. The reduction of Bodipy in PE-5×FAD mice was notably inhibited by OE-*Tmem9*-AAV injection. Meanwhile, the percentage of Bodipy co-localized with LC3 in microglia was significantly decreased in the 5×FAD mice compared to the WT. However, PE or *Tmem9*-RNAi treatment markedly increased the percentage, and this upregulation in PE-5×FAD mice was notably inhibited by OE-*Tmem9*-AAV injection (Fig. S3).

TEM revealed a significant accumulation of LDs in the 5×FAD mice compared to WT mice. The LDs fused with autophagosomes in 5×FAD mice with physical exercise or *Tmem9*-RNAi treatment, indicative of lipophagy. However, overexpression of *Tmem9* abolished the fusion between LDs and autophagosomes in PE-5×FAD mice. The number of LD-associated autophagic vacuoles per microglial cell was significantly increased in the 5×FAD mice with physical exercise or *Tmem9*-RNAi treatment compared to the control-5×FAD group. However, the upregulation in PE-5×FAD mice was notably inhibited by OE-*Tmem9*-AAV injection (Fig. 2d). Compared to the control-5×FAD group, Oil Red O-stained LDs were markedly reduced in both PE and RNAi-*Tmem9*-treated groups. Notably, the reduction of LDs in the brains of PE-treated 5×FAD mice was significantly attenuated by OE-*Tmem9*-AAV injection (Fig. 2e).

### Physical exercise alleviates microglial mitochondrial dysfunction and mitigates lipotoxicity in the brains of 5×FAD mice by down-regulating TMEM9

**Fig. 3:**
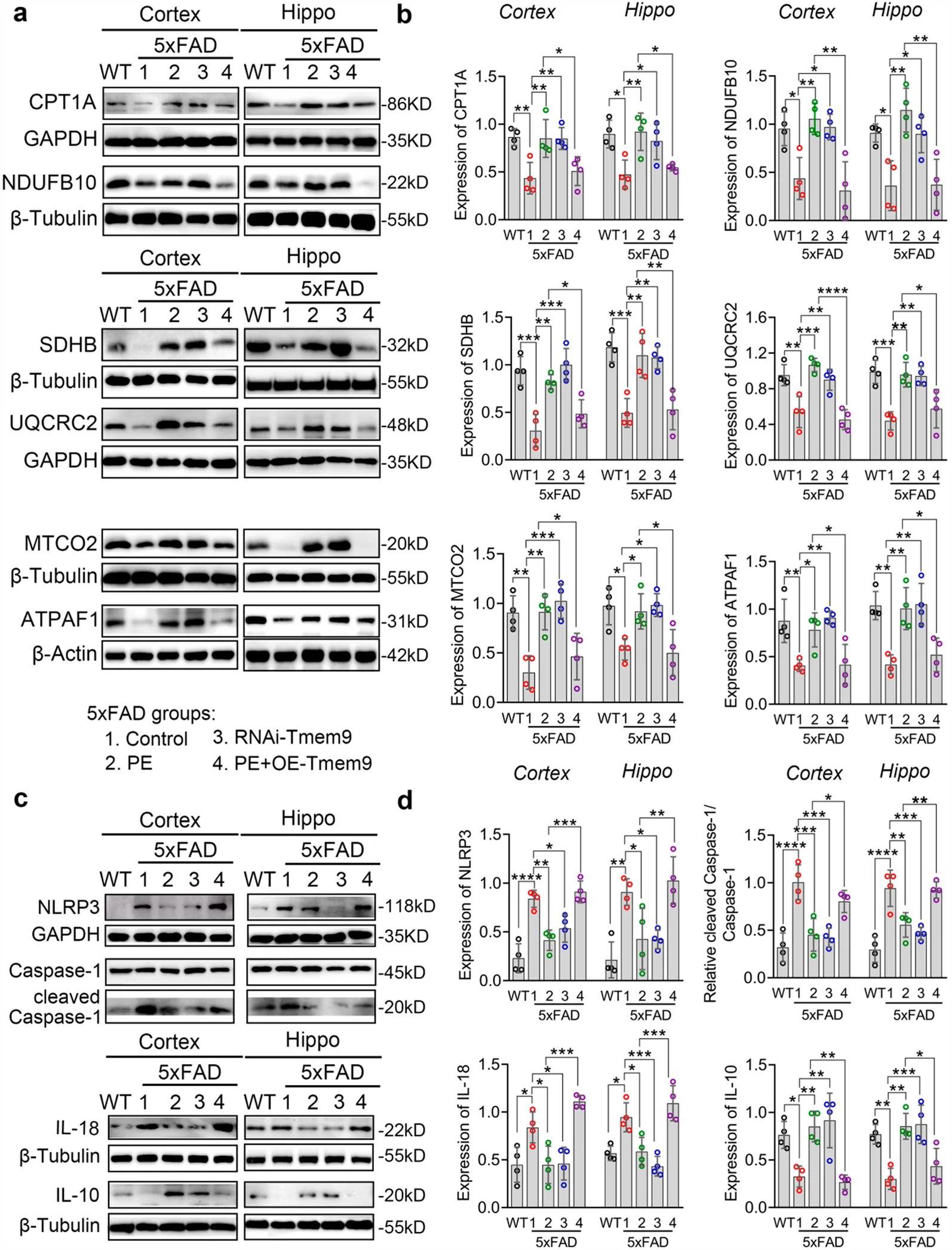
Physical exercise ameliorates mitochondrial dysfunction and attenuates neuroinflammation in 5×FAD mice by down-regulating microglial TMEM9. **a**, **b** Western blots analysis of carnitine palmitoyltransferase 1A (CPT1A), a key regulator of fatty acid oxidation, and mitochondrial electron transport chain components (NDUFB10, SDHB, UQCRC2, MTCO2 and ATPAF1) in WT, Ctrl-5×FAD, PE-5×FAD, RNAi-*Tmem9*, and PE+OE-*Tmem9* groups. **c, d** Western blots analysis of the pro- and anti-inflammatory cytokines including NLRP3, cleaved Caspase-1, IL-18 and IL-10 among WT, Ctrl-5×FAD, PE-5×FAD, RNAi-*Tmem9* and PE+OE-*Tmem9* groups. Data are expressed as the mean±SD, *n*=4 male mice per experimental group, aged 8 months. One-way analysis of variance, followed by Tukey’s multiple comparisons test. *P < 0.05, **P < 0.01, ***P < 0.001, ****P < 0.0001

As proteomics analysis showed that physical exercise upregulated proteins involved in oxidative phosphorylation and mitochondrial quality control (Fig. 1d), we next investigated the proteins associated with the electron transport chain (Fig. 3). The protein level of carnitine palmitoylcarnitine transferase 1 A (CPT1A), which catalyzes the rate-limiting step for mitochondrial long-chain fatty acid β-oxidation [35], was up-regulated in the 5×FAD mice with physical exercise or RNAi-*Tmem9* treatment compared to the control 5×FAD group, while no significant differences were observed between the control 5×FAD and the PE+OE-*Tmem9* groups (Fig. 3a, b). Besides, we verified that the proteins comprising all five complexes (I-V) in the mitochondrial respiratory chain, including NDUFB10, SDHB, UQCRC2, MTCO2 and ATPAF1, were decreased in the brains of 5×FAD mice compared to the WT group. Physical exercise upregulated these proteins, which could be mimicked by RNAi-*Tmem9* treatment but diminished by OE-*Tmem9* treatment (Fig. 3a, b). The protein levels of NLRP3, cleaved caspase-1 and IL-18 were increased, whereas IL-10 was decreased, in 5×FAD mice compared to WT mice (Fig. 3c, d). In contrast, physical exercise or RNAi-*Tmem9* significantly decreased the protein levels of these proinflammatory factors while increasing the IL-10 level. However, these protective effects of physical exercise were notably abolished by AAV-*Tmem9* injection (Fig. 3c, d). These results suggest that physical exercise or AAV-RNAi-*Tmem9 *treatment may enhance the release of fatty acids and facilitate their entry into mitochondria for fatty acid oxidation, thereby sustaining the mitochondrial respiratory function and mitigating lipotoxicity.

### Lipophagy protects microglia against oxidative stress induced by oAβ under energy-deficient conditions in vitro

**Fig. 4:**
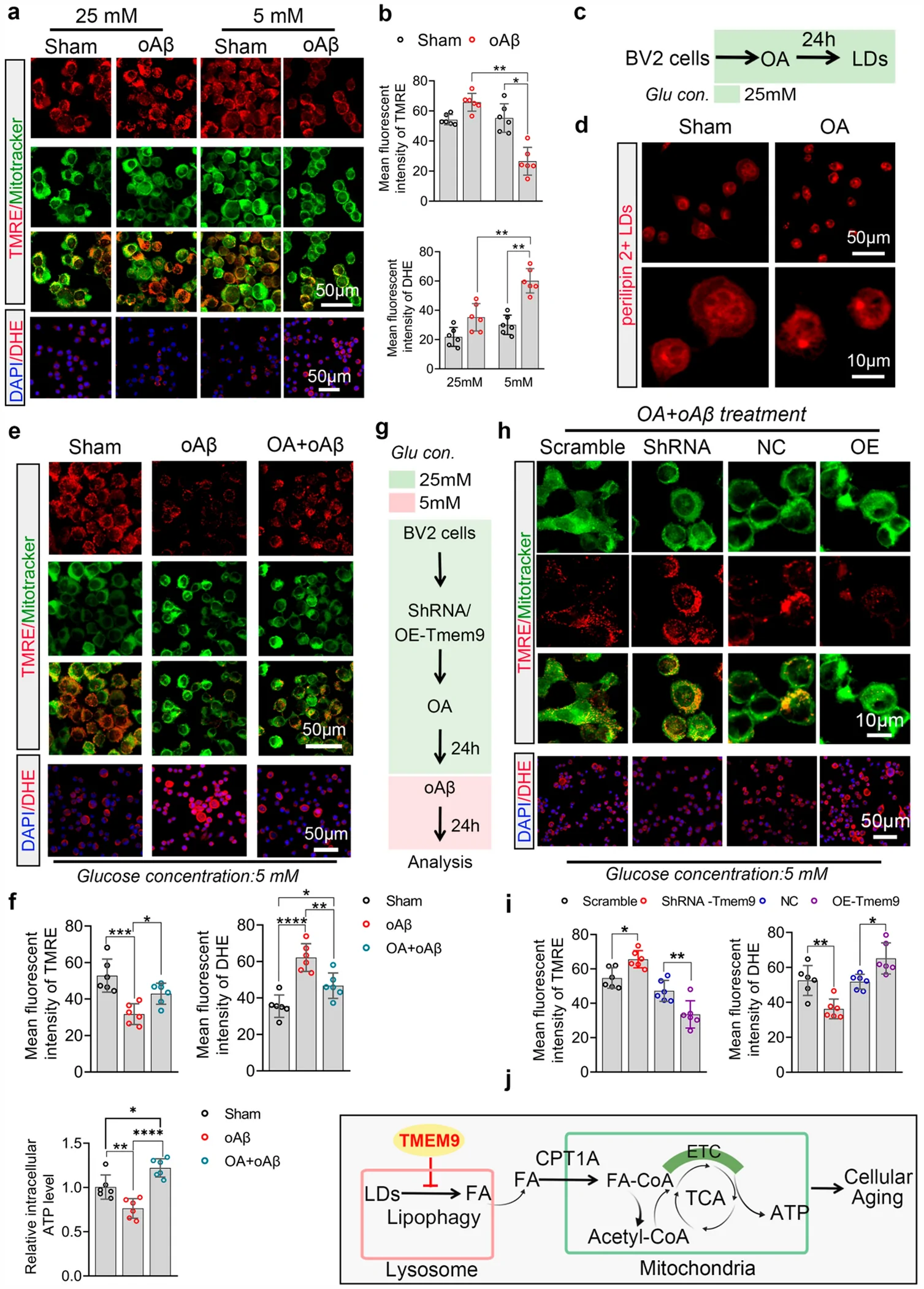
*Tmem9* knockdown alleviates oligomeric Aβ (oAβ)-induced oxidative stress under conditions of glucose insufficiency in vitro. **a**, **b** Representative images and quantitative analysis of mitochondrial membrane potential (TMRE) and dihydroethidium (DHE) detecting reactive oxygen species in BV2 cells treated with or without oAβ under normal (25 mM glucose) or restricted (5 mM glucose) conditions. **c, d** Schematic of oleic acid (OA)-induced lipid droplet (LD) formation in BV2 cells, confirmed by Perilipin 2 immunostaining (Upper panel: 20 × objective, 5 × zoom; lower panel: 2 × magnification in the upper panel). **e** TMRE and DHE fluorescence staining in BV2 cells under glucose restriction (5 mM) following treatment with vehicle (Sham), oAβ alone, or OA+oAβ combination. **f** Quantification of TMRE and DHE fluorescence intensity as well as intracellular ATP levels in BV2 cells under glucose restriction (5 mM) with different treatments (Sham, oAβ, or OA+oAβ). **g** Experimental timeline for transfection of shRNA-*Tmem9* or OE-*Tmem9* lentiviral constructs, followed by OA+oAβ treatment in BV2 cells under glucose restriction conditions. **h, i** Effects of scramble control, shRNA-*Tmem9*, negative control (NC), and OE-*Tmem9* on TMRE and DHE fluorescence in OA+oAβ-treated BV2 cells under glucose restriction. **j** Schematic diagram depicting the proposed mechanism by which TMEM9 inhibits lipophagy-mediated ATP production to facilitate cellular senescence. *Tmem9* knockdown and overexpression was implemented through plasmid transfection. Data are expressed as mean ± SD. *n* = 2 fields per well, 3 wells per group. One-way analysis of variance, followed by Tukey’s multiple comparisons test. *P < 0.05; **P < 0.01; ***P < 0.001; ****P < 0.0001

It has been reported that under energy-deficient conditions, LDs protect against lipotoxicity by sequestering fatty acids, and these free fatty acids are released from LDs and directed to mitochondria for fatty acid oxidation and ATP production [11]. We subsequently examined the mechanism by which the TMEM9-regulated lipophagy contributes to microglial mitochondrial quality control in AD brain. First, we observed that under normal glucose condition (25 mM), the pixel intensities of TMRE and DHE showed no significant differences between sham and oAβ-treated BV2 cells. Conversely, under restricted glucose condition (5 mM), oAβ incubation decreased TMRE intensity and enhanced DHE intensity (Fig. 4a, b), indicating oxidative stress-induced damage mediated by oAβ. We treated the BV2 cells with oleic acid to form LDs (Fig. 4c, d), and then incubated these cells with oAβ in the presence of 5 mM glucose to establish an in vitro AD model under condition of glucose insufficiency. Oleic acid treatment significantly ameliorated the oxidative stress damage induced by oAβ under conditions of glucose insufficiency, as evidenced by increased TMRE intensity and decreased DHE intensity (Fig. 4e, f). TMRE intensity in BV2 cells with oleic acid and oAβ treatments showed no difference compared with that in sham group whereas DHE intensity showed slightly elevated. Moreover, under conditions of glucose insufficiency, oAβ incubation decreased intracellular ATP level, while oleic acid treatment significantly increased it (Fig. 4f). These results indicate that the oleic acid-induced LDs protect microglia from oAβ-induced mitochondrial oxidative stress under energy-deficient conditions.

To further investigate the role of TMEM9 in LD-related mitochondrial protection in microglia, we transfected BV2 cells with shRNA-*Tmem9* or OE-*Tmem9* lentivirus to knockdown and overexpress *Tmem9*, respectively, with scramble control or negative control lentivirus as control (Fig. 4g). After 24 h of oleic acid treatment in the presence of 25 mM glucose, these transfected cells were incubated with oAβ in the presence of 5 mM glucose (Fig. 4g). The knockdown and overexpression of TMEM9 were validated by Western blots (Fig. S4a, b). Transfection of lentivirus did not affect LD formation, and no significant differences were observed in perilipin-2 intensity among the blank, scramble, negative control, shRNA-*Tmem9*, and OE-*Tmem9* groups (Fig. S4c). Compared to the scramble group receiving oleic acid plus oAβ treatments, the shRNA-*Tmem9* group displayed significantly increased TMRE intensity and decreased DHE intensity. Conversely, the OE-*Tmem9* group displayed decreased TMRE intensity and increased DHE intensity compared to the NC group (Fig. 4h, i). Western blotting results verified that the shRNA-*Tmem9* transfection increased autophagy-related proteins involved in lipophagy, including ATG5 and the LC3II/LC3I ratio compared to the scramble group, while OE-*Tmem9* transfection decreased these proteins compared to the negative control group (Fig. S5a, b).

We also transfected BV2 cells with shRNA-*Tmem9* or OE-*Tmem9* plasmid to knockdown and overexpress *Tmem9* for immunofluorescence assay. The knockdown and overexpression of *Tmem9* were validated by RT-qPCR (Fig. S5c). Autophagic flux was evaluated by examining the colocalization patterns of a tandem fluorescent mCherry-GFP-LC3 reporter vector. Within autophagosomes, GFP and mCherry signals overlap; however, upon fusion with lysosomes to form autolysosomes, the acidic environment quenches the green fluorescence of the fusion protein while preserving the red signal, thereby transforming this system into a dual-state sensor for distinguishing between autophagosomes and autolysosomes [36]. Autophagic flux results verified that the shRNA-*Tmem9* transfection increased the autolysosome/autophagosome ratio compared to the scramble group, while OE-*Tmem9* transfection decreased the autolysosome/autophagosome ratio compared to the negative control group under the oleic acid+oAβ condition (Fig. S5d, e). Western blots showed significantly higher levels of proteins comprising all five mitochondrial complexes (I–V) in the shRNA-*Tmem9* group compared to the scramble control, whereas these proteins were decreased in the OE-*Tmem9* group (Fig. S5f, g). In contrast, the pro-inflammatory factors, including NLRP3, inducible nitric oxide synthase (iNOS) and cleaved IL-1β, were decreased in the shRNA-*Tmem9* group compared to the scramble group, whereas they were increased in the OE-*Tmem9* group. The IL-10 level showed opposite changes (Fig. S5f–i).

We next performed lysosome inhibition experiments using chloroquine in BV2 cells following oleic acid and oAβ incubation (Fig. S6a). In the DMSO-treated groups, shRNA-*Tmem9* cells exhibited increased levels of ATG5 and an elevated LC3II/LC3I ratio, along with decreased P62 level, compared to the scramble control cells. However, chloroquine treatment attenuated the increases in ATG5, and elevated LC3II/LC3I ratio and P62 levels in the shRNA-*Tmem9* cells (Fig. S6b, d). Furthermore, the shRNA-*Tmem9* cells exhibited increased autolysosome/autophagosome ratio compared to scramble control cells in the DMSO-treated groups. But chloroquine treatment attenuated this increase in shRNA-*Tmem9* cells (Fig. S6c, d). Mitochondrial electron transport chain proteins including UQCRC2, SDHB, ATPAF1, NDUFB10, and MTCO2 were increased in shRNA-*Tmem9* cells compared to scramble control under DMSO treatment, but chloroquine treatment decreased these proteins in shRNA-*Tmem9* cells. Conversely, chloroquine treatment increased the levels of NLRP3 and cleaved IL-1β, while decreasing the anti-inflammatory cytokine IL-10, in shRNA-*Tmem9* cells compared to the DMSO group (Fig. S6e–h). Collectively, these findings establish that LDs maintain mitochondrial homeostasis by neutralizing the oAβ-induced oxidative stress under glucose-insufficiency conditions, thereby preserving the electron transport chain integrity and delaying cellular senescence. Crucially, lysosomal TMEM9 orchestrates the cytoprotective function of LDs: *Tmem9* overexpression disrupts lipophagy and blocks fatty acid mobilization, a process critical for ATP synthesis under glucose insufficiency, whereas *Tmem9* depletion enhances lipophagy to improve the mitochondrial adaptive capacity (Fig. 4j).

**Fig. 5:**
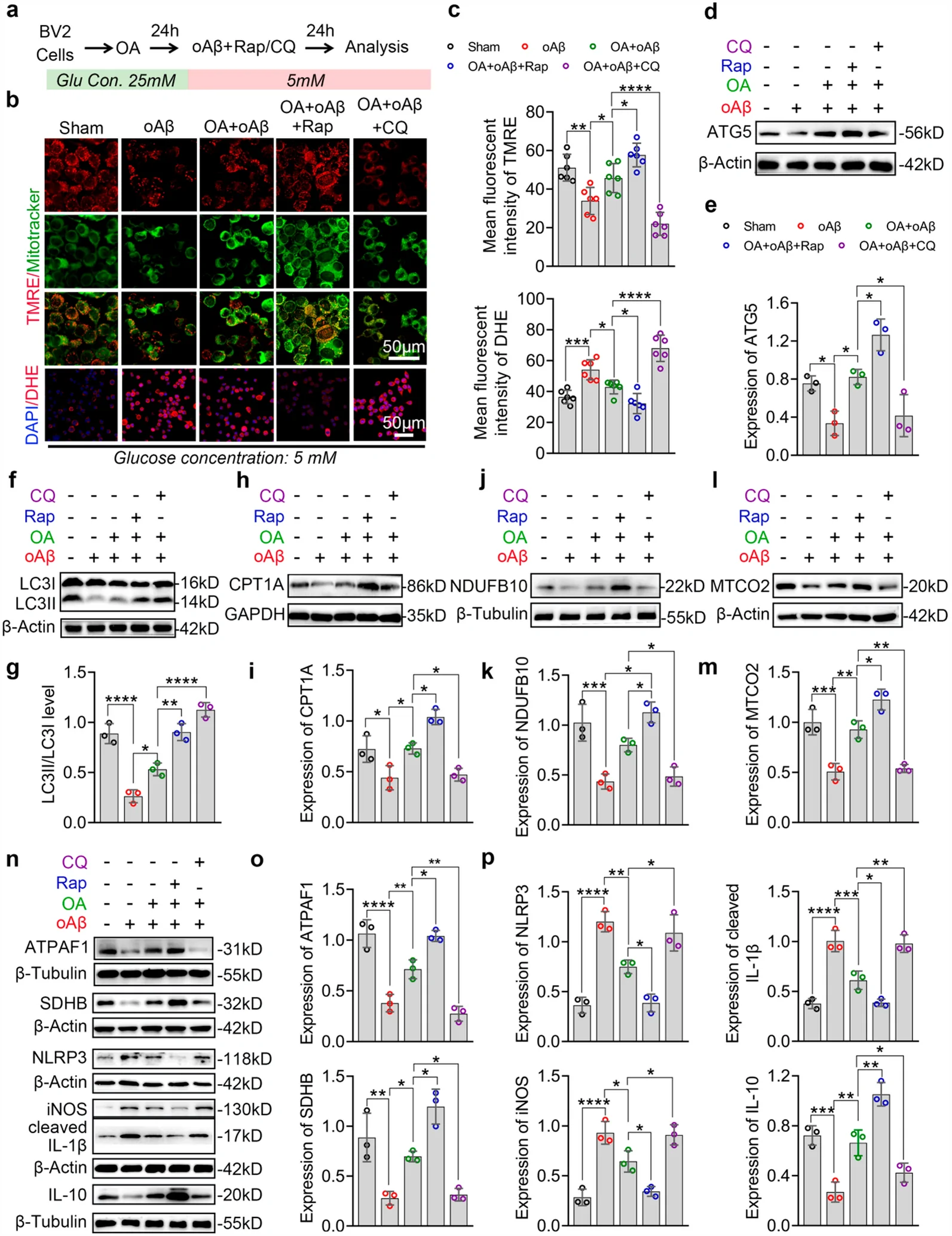
Lipophagy protects microglia against oligomeric Aβ (oAβ)-induced oxidative stress under conditions of glucose insufficiency in BV2 microglial cells. **a** Experimental timeline for oleic acid (OA) and oAβ treatment combined with autophagy regulators rapamycin (Rap) and chloroquine (CQ) in BV2 cells under conditions of glucose insufficiency. **b, c** Representative fluorescence images and quantitative analysis of TMRE and DHE in sham, oAβ, OA+oAβ, OA+oAβ+Rap (autophagy inducer), OA+oAβ+CQ (autophagy inhibitor) groups, *n* =2 fields per well, 3 wells per group. **d–g** Western blots of autophagy markers (ATG5 and LC3II/LC3I ratio) among different treatment groups, *n* = 3 wells per group. **h–m** Western blots analysis of CPT1A, NDUFB10, and MTCO2 among different treatment groups, *n* = 3 wells per group. **n–p** Western blots analysis of ATPAF1, SDHB, IL-10, NLRP3, iNOS and IL-1β among these different treatment groups. Data are expressed as the mean ± SD, *n* = 3 wells per group. One-way analysis of variance, followed by Tukey’s multiple comparisons test. *P < 0.05, **P < 0.01, ***P < 0.001, ****P < 0.0001

To confirm the role of lipophagy in the protective function of LDs in maintaining microglial mitochondrial integrity, BV2 cells were treated with oleic acid and then exposed to either rapamycin or chloroquine in combination with oAβ (Fig. 5a). Compared to cells treated with oleic acid plus oAβ alone, rapamycin significantly increased TMRE intensity while reducing DHE intensity. In contrast, chloroquine decreased TMRE intensity and elevated DHE intensity (Fig. 5b, c). Western blots further revealed that rapamycin upregulated autophagy-related proteins involved in lipophagy, including ATG5, whereas chloroquine downregulated these markers. However, both rapamycin and chloroquine upregulated the LC3II/LC3I ratio (Fig. 5d–g). Moreover, rapamycin increased the expression of CPT1A, mitochondrial respiratory chain proteins and anti-inflammatory cytokines, effects that were suppressed by chloroquine **(**Fig. 5h–p). Conversely, pro-inflammatory cytokines were reduced by rapamycin but were elevated upon chloroquine treatment (Fig. 5n–p).

### TMEM9 downregulation enhances lipophagy via activation of lysosomal AMPK signaling

**Fig. 6:**
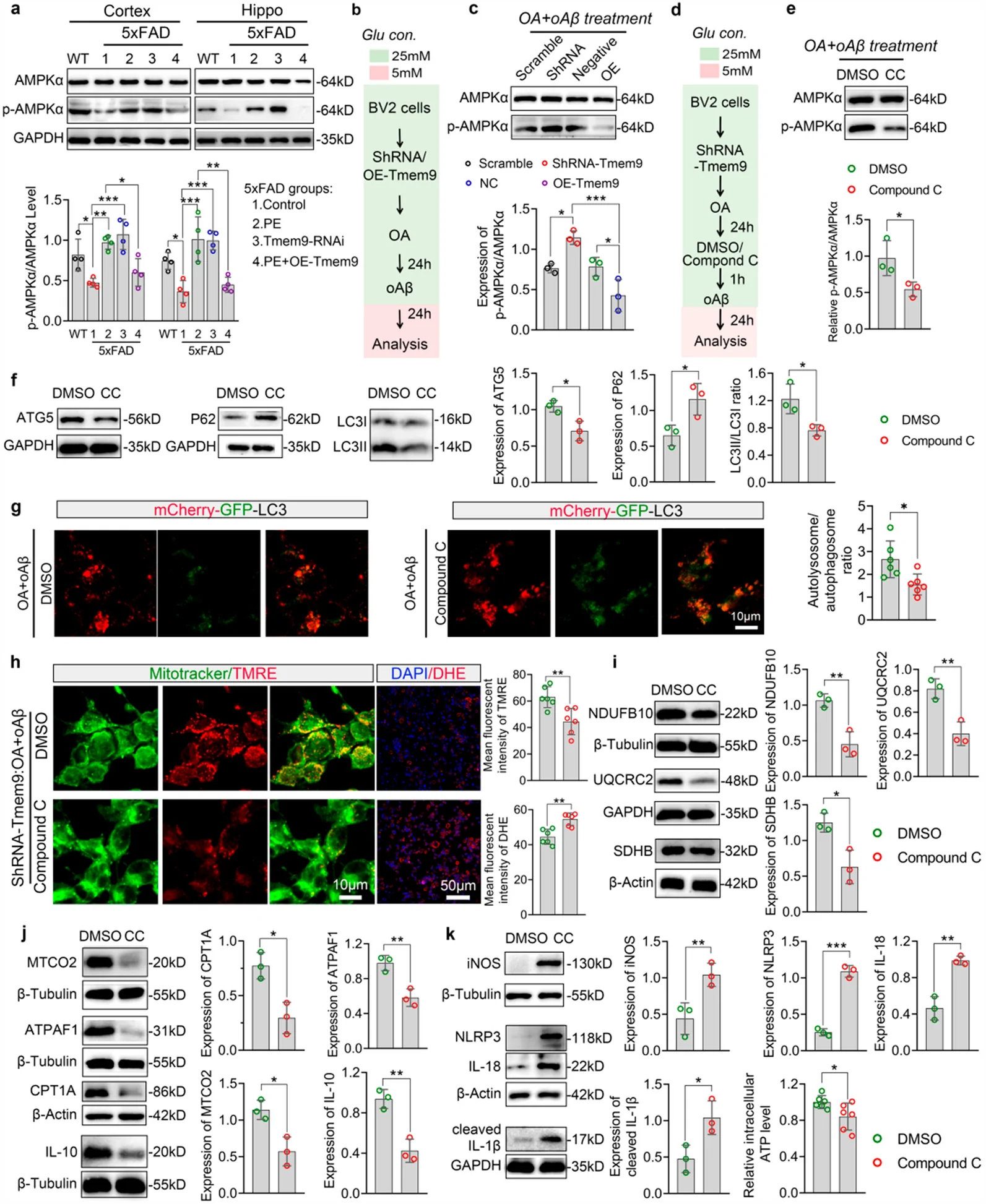
*Tmem9* knockdown promotes lipophagy and maintains mitochondrial function through activation of lysosomal AMPKα signaling under condition of glucose insufficiency. **a** Western blotting analysis of phosphorylated AMPKα (p-AMPKα) and total AMPKα in cortical and hippocampal lysates from WT, Ctrl-5×FAD, PE-5×FAD, RNAi-*Tmem9*, and PE+OE-*Tmem9* mice, *n* = 4 male mice per group, aged 8 months. One-way analysis of variance (ANOVA), followed by Tukey’s multiple comparisons test. **b** Experimental timeline for oleic acid (OA) and oligomeric Aβ (oAβ) treatment in BV2 cells following *Tmem9* knockdown (shRNA) or overexpression (OE). **c** Western blotting analysis of p-AMPKα/AMPKα in scramble control, shRNA-*Tmem9*, negative control (NC), and OE-*Tmem9* groups, *n*=3 wells per group. One-way analysis of variance (ANOVA), followed by Tukey’s multiple comparisons test. **d** Experimental design for OA+oAβ treatment in shRNA-*Tmem9*-transfected BV2 cells treated with either DMSO or Compound C (an AMPK inhibitor) under glucose restriction conditions. **e** Effect of Compound C on AMPKα phosphorylation (p-AMPKα/AMPKα) in shRNA-*Tmem9* BV2 cells. Statistical significance was assessed using independent-samples *t*-test. **f** Effect of Compound C on autophagy markers (ATG5, SQSTM1/P62, LC3II/LC3I) in DMSO- and Compound C-treated groups. **g** Effect of Compound C on the autophagy flux (autolysosome/autophagosome ratio) in DMSO- and Compound C-treated groups. **h** Representative images and quantification of TMRE and DHE following AMPK inhibition in the shRNA-*Tmem9* cells following OA+oAβ treatment under condition of glucose insufficiency. Statistical significance was assessed using independent-samples* t*-test. *Tmem9* knockdown was implemented through plasmid transfection. **i-k** Western blotting analysis of mitochondrial electron transport chain components (NDUFB10, UQCRC2, SDHB, MTCO2, ATPAF1), CPT1A, anti-inflammatory cytokine IL-10, and pro-inflammatory cytokines, including iNOS, NLRP3, IL-18, and IL-1β. Statistical significance was assessed using independent-samples *t*-test. *Tmem9* knockdown and overexpression was implemented through lentivirus transfection. Data are expressed as mean ± SD. *P < 0.05; **P < 0.01; ***P < 0.001; ****P < 0.0001

AMPK acts as a central cellular energy sensor, which is activated at the lysosome independently of elevated AMP levels at energy-deficient states such as glucose deprivation, to restore cellular energy homeostasis [37, 38]. Therefore, we investigated the effect of physical exercise on AMPK activation in AD brain, as well as the regulatory role of lysosomal TMEM9. As shown in Fig. 6a, phosphorylated AMPKα level was significantly decreased in the control 5×FAD group compared to the WT, but increased with physical exercise and the RNAi-*Tmem9* treatment. However, in the PE+OE-*Tmem9* group, the phosphorylated AMPKα levels were downregulated compared to the PE group, in both cortex and hippocampus (Fig. 6a). In BV2 microglial cells pre-treated with oleic acid and subsequently incubated with oAβ under low-glucose condition (Fig. 6b), transfection with shRNA-*Tmem9* significantly increased AMPKα phosphorylation compared to the scramble control group. Conversely, *Tmem9* overexpression inhibited AMPKα phosphorylation compared to the negative control group (Fig. 6c). We then treated the shRNA-*Tmem9*-transfected BV2 cells with compound C (CC, an AMPK inhibitor) or DMSO (vehicle control), followed by incubation with oAβ in low-glucose medium (Fig. 6d, e). As expected, CC treatment decreased the lipophagy-related protein ATG5 and the LC3II/LC3I ratio, compared to the DMSO group (Fig. 6f). CC treatment also decreased the autolysosome/autophagosome ratio compared to the DMSO group (Fig. 6g). Furthermore, CC treatment reduced TMRE intensity and increased DHE intensity compared to the DMSO group (Fig. 6h). Similarly, protein levels of mitochondrial electron transport chain proteins and the anti-inflammatory cytokine IL-10 were reduced in the CC group. Conversely, the levels of pro-inflammatory cytokines (iNOS, NLRP3, IL-18, IL-1β) were elevated in the CC group compared to the DMSO group (Fig. 6i–k). Collectively, these results demonstrate that downregulation of *Tmem9* enhances microglial lipophagy and protects against mitochondrial dysfunction by activating AMPK signaling, an effect that can be blocked by AMPK inhibitor.

### TMEM9 suppresses lysosomal AMPK activation through interactions with ATP6AP1 in AD, an effect reversed by physical exercise

**Fig. 7:**
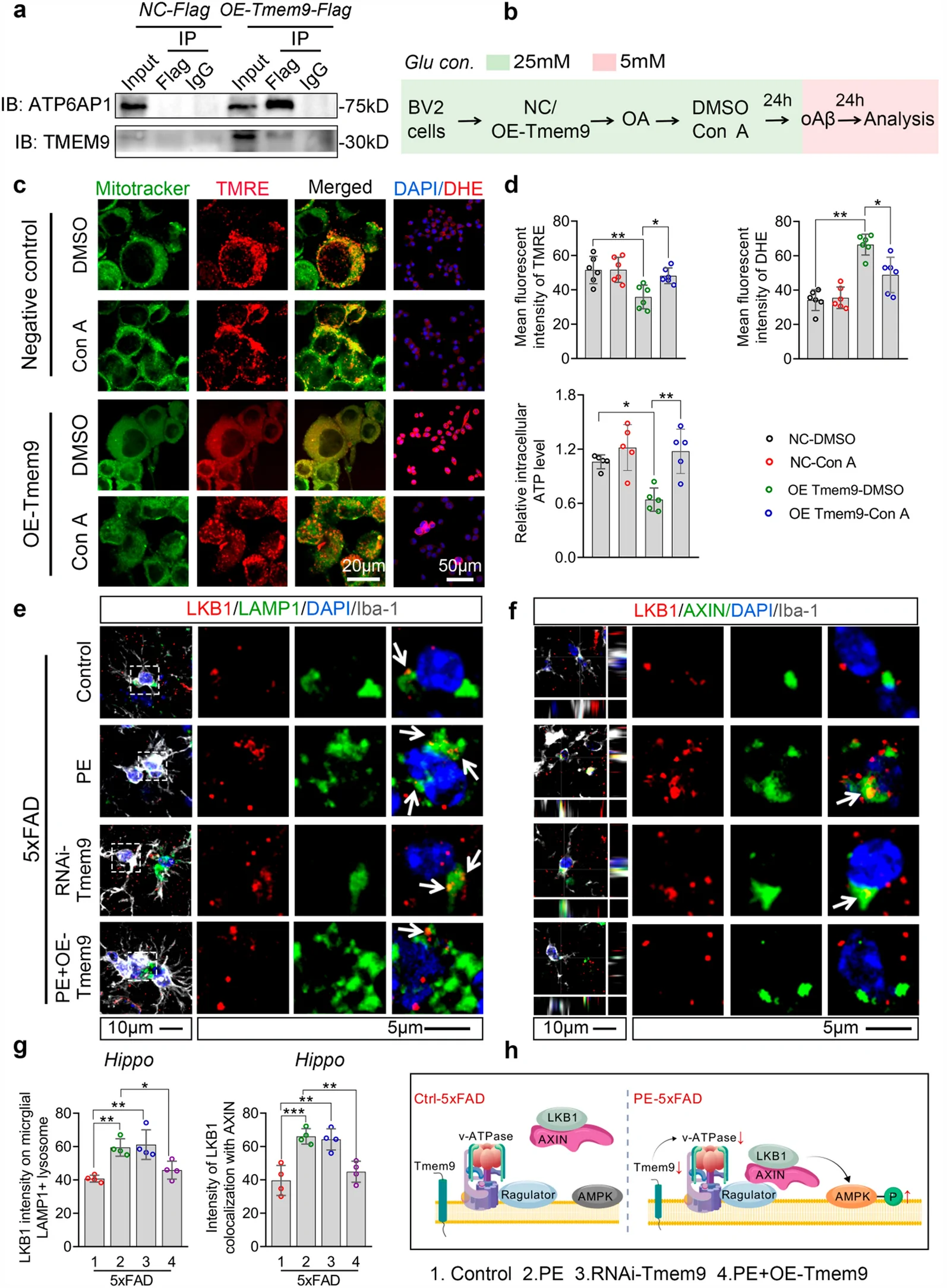
*Tmem9* downregulation promotes lysosomal AMPK activation through LKB1 translocation. **a** Co-immunoprecipitation demonstrating TMEM9–ATP6AP1 interaction in BV2 cells transfected with control (NC-Flag-Lv) or *Tmem9*-overexpressing (OE-*Tmem9*-Flag-Lv) vectors. **b** Experimental timeline for OA+oAβ treatment in BV2 cells transfected with control (NC) or OE-*Tmem9* vectors, followed by DMSO or concanamycin A (Con A, V-ATPase inhibitor) treatment. **c, d** Representative images and quantitative analysis of TMRE and DHE mean intensities among NC+DMSO, NC+Con A, OE-*Tmem9*+DMSO and OE-*Tmem9* + Con A groups, *n* =2 fields per well, 3 wells per group. **e–g** Representative immunofluorescent images and quantitative analysis of LKB1 localization on LAMP1-positive lysosomal membranes, and LKB1-AXIN colocalization among Ctrl-5×FAD, PE-5×FAD, RNAi-*Tmem9*, and PE+OE-*Tmem9* groups, *n* = 4 male mice per group, at age of 8 months. **h** Proposed mechanistic diagram illustrating that exercise-induced activation of AMPK occurs through TMEM9-regulated lysosomal LKB1 translocation. *Tmem9* overexpression in vitro was implemented through plasmid transfection. Data are expressed as mean±SD. *P < 0.05, **P < 0.01, ***P < 0.001, ****P < 0.0001. One-way analysis of variance, followed by Tukey’s multiple comparisons test

TMEM9 has been reported to regulate microglial function by interacting with V-ATPase [23], and inhibition of V-ATPase is a prerequisite for lysosomal AMPK activation [39]. We therefore analyzed the interaction between TMEM9 and ATP6AP1 (ATPase H^+^ transporting accessory protein 1, also known as Ac45), an accessory factor of V-ATPase in brain. Co-immunoprecipitation assays in BV2 cells confirmed a direct interaction between TMEM9 and ATP6AP1 (Fig. 7a). Specifically, Flag-tagged *Tmem9* overexpression enhanced this interaction, while the Flag-tagged negative control plasmid failed to pull down ATP6AP1 (Fig. 7a). To further assess the involvement of ATP6AP1 in TMEM9-induced lipophagy dysfunction and oAβ-induced mitochondrial damage, *Tmem9*-overexpressing BV2 cells were pre-treated with concanamycin A (Con A) or DMSO for 1 h in low-glucose medium, followed by oleic acid treatment and subsequent incubation with oAβ (Fig. 7b). In cells transfected with the NC lentivirus, TMRE intensity, DHE intensity, and ATP levels showed no significant differences between DMSO- and Con A-treated groups. In contrast, *Tmem9*-overexpressing cells exhibited markedly increased TMRE intensity and ATP levels, alongside decreased DHE intensity, in the Con A group compared to the DMSO-treated group (Fig. 7c, d).

Notably, glucose starvation has been reported to promote AMPK binding to the AXIN-LKB1 complex, leading to the formation of the AXIN–LKB1–AMPK complex and subsequent AMPK activation [38]. In addition, the V-ATPase-Ragulator complex serves as an endosomal docking site for LKB1-mediated AMPK activation by forming the V-ATPase-Ragulator-AXIN-LKB1-AMPK complex [38]. Therefore, we next examined the lysosomal AXIN-LKB1 complex in microglial cells to investigate the mechanism by which exercise regulates lysosomal AMPK activation, as well as the involvement of the TMEM9-ATP6AP1 axis. Consistent with previous report [40], the AMPK-activating kinase LKB1 exhibited lysosomal localization on lysosomal-associated membrane protein 1 (LAMP1)-positive membrane of cortical microglia of 5×FAD mice. Physical exercise and RNAi-*Tmem9* treatments enhanced lysosomal LKB1 levels, while microglial *Tmem9* overexpression abolished this effect in exercised mice (Fig. 7e–g). In vitro, we also verified that *Tmem9* knockdown enhanced LKB1 localization with lysosome and increased the total level and the lysosomal level of LKB1 (Fig. S7a–c). Furthermore, unlike the control-5×FAD group, LKB1 was observed colocalizing with AXIN in 5×FAD mice with physical exercise and RNAi-*Tmem9*. Conversely, *Tmem9* overexpression diminished this colocalization in 5×FAD mice with physical exercise (Fig. 7f, g). Since AXIN facilitates LKB1 translocation to the lysosomal surface, these results indicate that *Tmem9* knockdown promotes this process [41]. Collectively, these findings suggest that downregulation of *Tmem9* reduces its interaction with ATP6AP1, thereby promoting the translocation of LKB1 to the lysosomal surface. Similarly, in DMSO-treated cells, LKB1 and p-AMPKα levels were lower in *Tmem9*-overexpressing cells than in NC-transfected cells. Con A treatment increased LKB1 and p-AMPKα levels in *Tmem9*-overexpressing cells (Fig. S8a, b). Within DMSO-treated groups, proteins related to β oxidation and mitochondrial electron transport chain (CPT1A, SDHB, MTCO2, ATPAF1) and the anti-inflammatory cytokine IL-10 were decreased in *Tmem9*-overexpressing cells compared to negative control transfection. These reductions were rescued by Con A treatment (Fig. S8a, d). In contrast, pro-inflammatory mediators, including iNOS, NLRP3, IL-1β, and cleaved Caspase-1, were elevated in *Tmem9*-overexpressing cells compared to negative control transfection within DMSO-treated groups, and Con A treatment suppressed these elevations (Fig. S8d, f). These results indicate that the V-ATPase inhibitor rescues the oleic acid-associated protective mechanisms under the condition of TMEM9 increase.

### Physical exercise reduces Aβ plaque deposition and mitigates cognitive impairment in 5×FAD mice by down-regulating microglial TMEM9

**Fig. 8:**
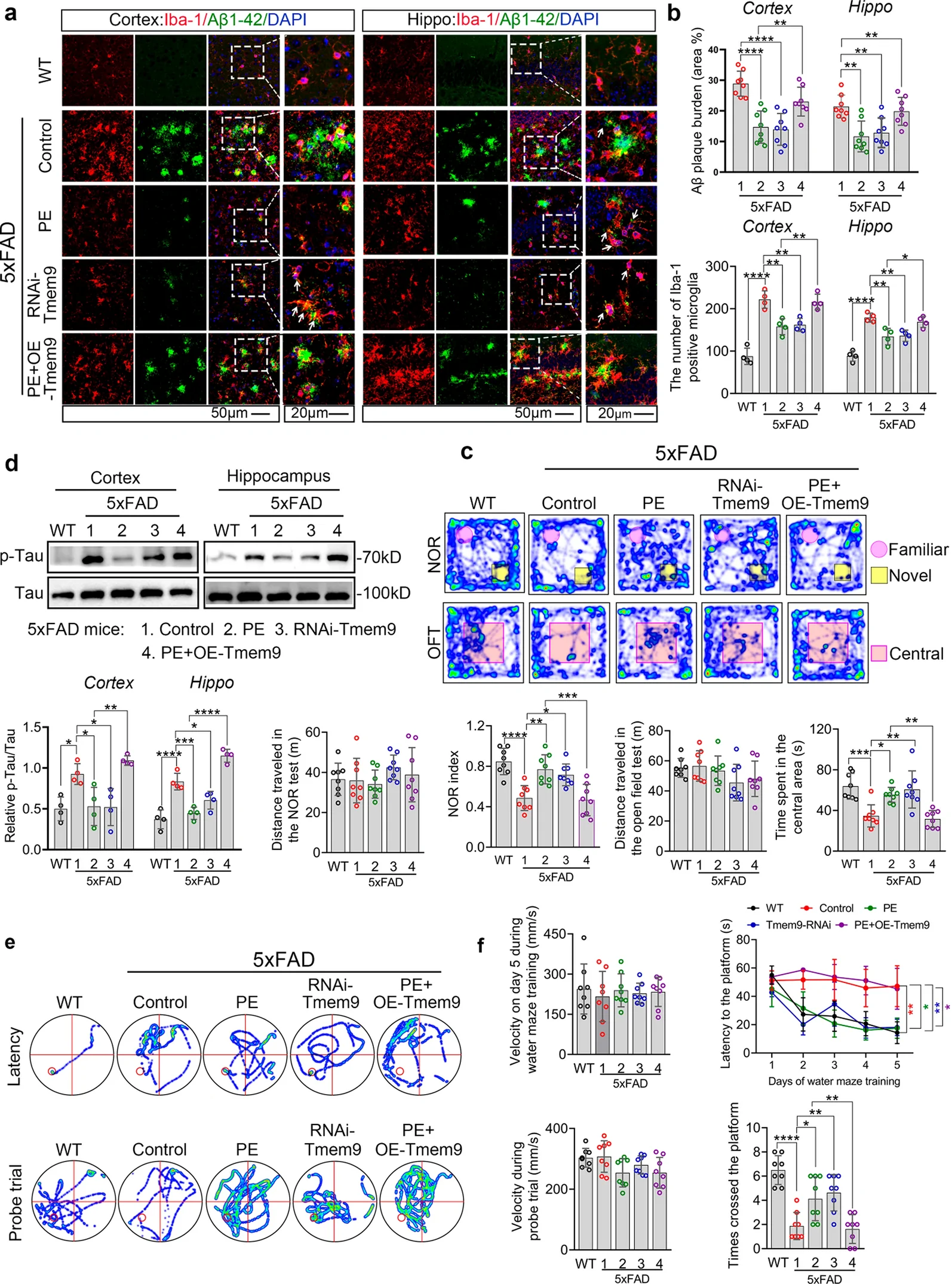
Physical exercise reduces Aβ plaque deposition, alleviates anxiety-like behaviors, and mitigates cognitive impairments in 5×FAD mice by down-regulating TMEM9. **a**, **b** Representative immunofluorescence images and quantitative analysis of microglial activation (Iba1) and amyloid-β plaque load (Aβ_1-42_) in parietal cortex and dentate gyrus of hippocampus in WT, Ctrl-5×FAD, PE-5×FAD, RNAi-*Tmem9*, and PE+OE-*Tmem9* groups, *n* = 4 male mice per group, 2 slices per mice, at age of 8 months. One-way analysis of variance, followed by Tukey’s multiple comparisons test. **c** Travelling path tracings of mice during the novel object recognition (NOR) and open field test (OFT), and quantitative analysis. *n* = 8 male mice per group, at age of 8 months. One-way analysis of variance, followed by Tukey’s multiple comparisons test. **d** Western blotting analysis of p-tau/tau. **e** Spatial learning trajectories during water maze training and the probe trial. **f** Velocities on day 5 of training and in the probe trial, as well as latencies to the platform during the training and in the probe trial. *n* = 8 male mice per group, at age of 8 months. For analysis of latency during 5-day training, statistical significance was assessed using two-way analysis of variance, followed by Tukey’s multiple comparisons test. Other data were analyzed by one-way analysis of variance, followed by Tukey’s multiple comparisons test. Data are expressed as mean ± SD. *P < 0.05, **P < 0.01, ***P < 0.001, ****P < 0.0001

Reprogramming of microglial lipid metabolism promotes Aβ phagocytosis and ameliorates cognitive dysfunction in AD [42]. We therefore investigated whether physical exercise reduces Aβ deposition and microglial activation in 5×FAD mouse brain through TMEM9 regulation. As expected, both Aβ deposition and microglial activation decreased following physical exercise or RNAi-*Tmem9* treatment in AD mice. Conversely, microglial *Tmem9* overexpression abolished the protective effects of physical exercise (Fig. 8a, b). Furthermore, Western blotting showed that physical exercise or RNAi-*Tmem9* treatment decreased the p-tau/tau levels in 5×FAD mice, while *Tmem9* overexpression abolished the protective effects of physical exercise (Fig. 8d).

In the novel object recognition test assessing recognition memory and the open field test, the distance traveled in both tests showed no significant differences among five groups. In the novel object recognition test, the 5×FAD mice showed significantly reduced discrimination index compared to WT. Both physical exercise and RNAi-*Tmem9* increased the discrimination index of the 5×FAD mice. Conversely, *Tmem9* overexpression abolished this improvement caused by physical exercise (Fig. 8d). In the open field test, 5×FAD mice spent significantly less time in the central area than WT. Physical exercise significantly increased the center time of 5×FAD mice, an effect replicated by *Tmem9* knockdown but attenuated by *Tmem9* overexpression (Fig. 8c). During Morris Water Maze training, the escape latencies showed no group differences on day 1. But on day 5, 5×FAD mice exhibited significantly prolonged latency versus WT. Both physical exercise and *Tmem9* knockdown shortened the latencies, whereas *Tmem9* overexpression in microglia reversed the beneficial effect of physical exercise. The swimming speed on day 5 showed no significant differences among five groups (Fig. 8e, f). In the probe trial, 5×FAD mice made fewer platform crossings than WT. Physical exercise of 5×FAD mice significantly increased crossings, an improvement similarly achieved by *Tmem9* knockdown but diminished by *Tmem9* overexpression. The swimming speed during the probe trial showed no significant differences among five groups (Fig. 8e, f). We also verified that AAV injection itself had no effect on the motor capacity of 5×FAD mice with and without physical exercise (Fig. S9a). The results demonstrate that physical exercise alleviates anxiety-like behaviors and cognitive deficits in AD mice, effects recapitulated by microglial *Tmem9* knockdown but abrogated by *Tmem9* overexpression.

### TMEM9/ATP6AP1 is a promising marker for male AD patients

PET-CT scans using the radiotracer ^18^F-AV45 revealed no cortical radiotracer distribution with clear gray-white matter contrast in control subjects. In contrast, AD patients showed obvious cortical radiotracer distribution with loss of gray-white matter differentiation, particularly in bilateral frontal, parietal, and temporal lobes, indicating Aβ deposition. Quantitative analysis also showed increased SUVR in bilateral intraparietal, frontal, occipital and temporal cortex in AD patients compared to control subjects (Fig. S10a). ^18^F-FDG PET-CT scans revealed significantly reduced radiotracer uptake across bilateral cerebral cortices in AD patients compared to controls, indicating characteristic cerebral glucose hypometabolism. Quantitative analysis also showed decreased SUVmax of bilateral basal ganglion and thalamus in AD patients compared to control subjects (Fig. S10b). However, there was no correlation between cerebral glucose uptake (^18^F-FDG PET-CT) and Aβ deposition levels (^18^F-AV45 PET-CT) (*R* = −0.127, *P* = 0.838).

**Fig. 9:**
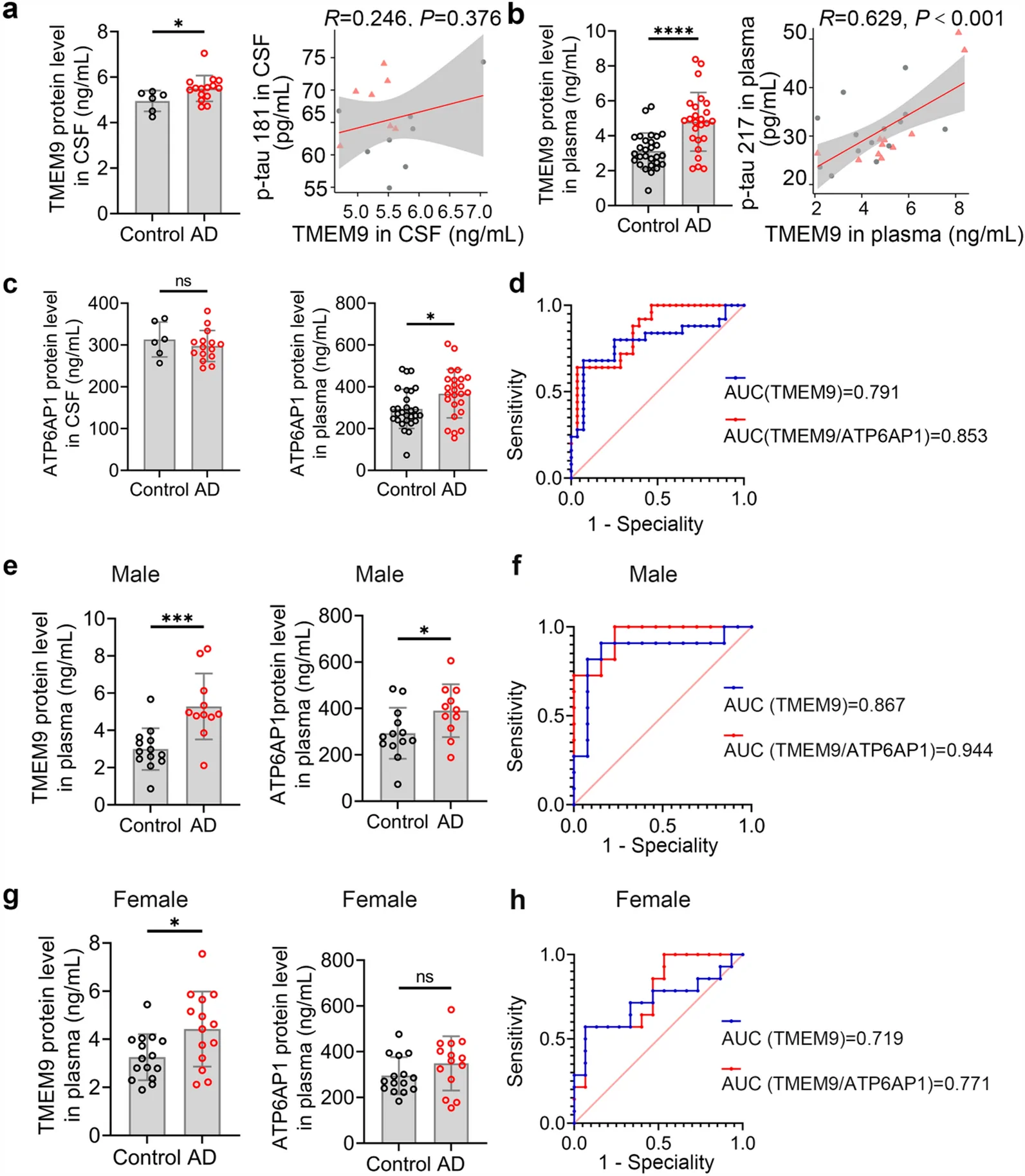
TMEM9/ATP6AP1 is a potential diagnostic biomarker and therapeutic target in AD patients. **a** ELISA quantification of TMEM9 protein in the CSF from control subject (*n* = 6) and AD patients (*n* = 15), and correlation analysis of CSF TMEM9 with CSF p-tau181 level in AD patients (*n* = 15, 8 females and 7 males). **b** ELISA quantification of TMEM9 protein in plasma from control subjects (*n* = 28) and AD patients (*n* = 25), and its correlation with plasma p-tau217 levels in AD patients (*n* = 25, 14 females and 11 males). **c** ELISA quantification of ATP6AP1 protein in CSF and plasma. **d** Receiver Operating Characteristic (ROC) analysis evaluating the diagnostic performance of TMEM9 and the TMEM9/ATP6AP1 ratio in distinguishing AD patients from controls. **e** ELISA quantification of TMEM9 and ATP6AP1 protein levels in plasma from male control subjects (*n* = 13) and male AD patients (*n* = 11). **f** ROC analysis evaluating the diagnostic performance of TMEM9 and the TMEM9/ATP6AP1 ratio in distinguishing male AD patients from male controls. **g** ELISA quantification of TMEM9 and ATP6AP1 protein levels in plasma from female control subjects (*n* = 15) and AD patients (*n* = 14). **h** ROC analysis evaluating the diagnostic performance of TMEM9 and the TMEM9/ATP6AP1 ratio in distinguishing male AD patients from female controls. Data are expressed as the mean ± SD. *P < 0.05, **P < 0.01, ***P < 0.001, ****P < 0.0001

We next quantified TMEM9 protein levels in CSF and plasma (Fig. 9a, b). Both CSF and plasma levels of TMEM9 were significantly elevated in AD patients compared to control subjects. Notably, CSF TMEM9 showed no correlation with CSF p-tau181 level (*R* = 0.246), and plasma TMEM9 correlated with plasma p-tau217 (*R* = 0.629) in the AD cohort. ATP6AP1 in the CSF showed no significant difference and ATP6AP1 in plasma was significantly elevated in AD patients compared to control subjects (Fig. 9c). We then generated receiver operating characteristic (ROC) curves for plasma TMEM9 levels and the TMEM9/ATP6AP1 ratio to evaluate their diagnostic reliability and model performance (Fig. 9d). The plasma TMEM9 protein demonstrated good diagnostic performance for AD dementia (AUC = 0.791, 95% CI = 0.660–0.922). Notably, the plasma TMEM9/ATP6AP1 ratio showed even higher diagnostic performance (AUC = 0.853, 95% CI = 0.753–0.953). In male subjects, plasma TMEM9 and ATP6AP1 levels were significantly elevated in AD patients compared to controls (Fig. 9e). The plasma TMEM9 level demonstrated diagnostic performance for AD in male subjects (AUC = 0.867, 95% CI = 0.698–1.000), and the plasma TMEM9/ATP6AP1 ratio showed even higher diagnostic performance (AUC = 0.944, 95% CI = 0.860–1.000) (Fig. 9f). For female subjects, the plasma TMEM9 level was significantly elevated in AD patients compared to controls, whereas the plasma ATP6AP1 level did not show significant difference between these two groups (Fig. 9g). The plasma TMEM9 protein demonstrated diagnostic performance for AD in female subjects (AUC = 0.719, 95% CI = 0.523–0.915), and the plasma TMEM9/ATP6AP1 ratio showed even higher diagnostic performance (AUC = 0.771, 95% CI = 0.599–0.944) (Fig. 9h).

We next performed correlation analyses of plasma and CSF biomarkers (p-tau 181, TMEM9, ATP6AP1) with cerebral glucose metabolism (SUVRmax on ^18^F-FDG PET-CT) and Aβ deposition (SUVR on ^18^F-AV45 PET-CT) (Fig. S10c, d, Tables S3 and S4). The results revealed a statistically significant positive correlation between CSF p-tau181 levels and amyloid burden in the right intraparietal cortex, left intraparietal cortex, and right temporal cortex. In contrast, a negative correlation was observed between CSF TMEM9 levels and cerebral glucose uptake in the left basal ganglion, right basal ganglion, left thalamus, and right thalamus.

## Discussion

In the present study, we found that physical exercise promotes lipophagy in a TMEM9-dependent manner in the AD brain, thereby protecting microglial mitochondria from oxidative stress damage and enhancing ATP production. Consequently, this process leads to a reduction in pro-inflammatory cytokines and an increase in phagocytic capacity, which ultimately results in decreased Aβ deposition and alleviation of cognitive impairment in AD.

Microglia act as a double-edged sword in AD: they initially facilitate the clearance of Aβ and tau aggregates, but their sustained activation can trigger chronic neuroinflammation, leading to synaptic dysfunction and neuronal loss [43]. In this study, we treated 5×FAD mice from 5 months of age to 8 months of age and analyzed all behavioral and pathological outcomes at the 8-month age. Our results demonstrated that physical exercise reduces Aβ accumulation and mitigates cognitive impairment by promoting microglial protection. We selected the 5-month age time point because it corresponds to an early pathological stage characterized by initial amyloid deposition and microglial activation [44], overlapping with the emergence of disease-associated microglia [45, 46]. Initiating intervention at this stage enables assessment of early microglial modulation, while analysis at 8-month age captures its impact on more advanced pathology [44, 47]. Physical exercise directs microglia toward a protective phenotype through several mechanisms. First, it decreases LD accumulation in microglia, as LD-associated microglia are characterized by defective phagocytosis, increased production of reactive oxygen species, and elevated release of pro-inflammatory cytokines [48]. Second, physical exercise may enhance microglial phagocytosis of Aβ and reduce plaque burden by activating AMPK signaling, a crucial regulator that facilitates the polarization of microglia from a pro-inflammatory to an anti-inflammatory phenotype [49]. Consistently, our results suggest that physical exercise enhances AMPK phosphorylation in the AD brain. Third, physical exercise may support microglial protection by enhancing the autophagy–lysosomal pathway, as evidenced by upregulated ATG5 expression, increased LC3II/LC3I ratio, and decreased P62/SQSTM1 levels, as autophagy–lysosomal impairment is mechanistically linked to microglial senescence [50]. However, Svensson et al. reported that voluntary running did not reduce neuroinflammation or insoluble Aβ in 5×FAD mice [51]. This discrepancy may be attributed to two reasons. First, the intervention duration and the timing differed between studies. Svensson et al. initiated running at two months of age for six months, whereas we began at five months of age and continued for three months. Second, the difference in the sex of animals likely contributed to the divergent outcomes, as Svensson et al. used female mice while we used males. It has been demonstrated that the biological mechanisms underlying the beneficial effects of exercise on the brain may differ between males and females [52, 53]. In our study, intervention was initiated at 5 months and analysis performed at 8 months. The 5-month age represents early pathology with emerging disease-associated microglia [44, 45], while the 8-month age reflects more advanced disease [44, 47]. This design enables evaluation of how early intervention impacts subsequent disease progression. However, only male 5×FAD mice were used in this study. Given that females exhibit earlier and more severe amyloid pathology and distinct microglial responses [44, 54], sex-dependent effects may influence our findings. Therefore, although the use of male mice reduces experimental variability, the current findings may not fully reflect sex-specific effects, and future studies including both sexes are warranted.

A key finding of this study is that microglial lipophagy critically mediates the neuroprotective effects of physical exercise. This protective effect operates through two principal mechanisms. First, excessive LDs, indicative of dysregulated lipid metabolism, contribute to AD pathogenesis by promoting accumulation of Aβ and phosphorylated tau, exacerbating mitochondrial oxidative stress, and inducing neuroinflammation [55]. Second, lipophagy liberates fatty acids from LDs. These fatty acids undergo mitochondrial β-oxidation, elevating ATP levels within microglia, a process essential for Aβ clearance. Given the early impairment of glucose metabolism in AD (as established by PET-CT scans in our study) [56], microglia may compensate by mobilizing lipid metabolism to rapidly generate ATP for energy supply and immune surveillance beyond glucose utilization [42]. We thus propose that physical exercise sustains microglial ATP production under glucose-limited conditions via CPT1A-mediated fatty acid transport into mitochondria, followed by β-oxidation and the citric acid cycle. Notably, CPT1A expression is upregulated by physical exercise, counteracting its reported downregulation in senescent cells, which triggers mitochondrial dysfunction [57]. Additionally, we demonstrated that intact lipophagy enables LDs to elevate ATP levels during glucose insufficiency in BV2 cells.

The second key finding of our study is that physical exercise promotes lipophagy and reduces LD accumulation by downregulating microglial TMEM9. TMEM9 upregulation impairs microglial lipophagy, resulting in mitochondrial oxidative stress damage and neuroinflammatory responses, thereby contributing to the pathogenesis of AD. TMEM9 may inhibit lipophagy through multiple mechanisms. First, TMEM9 may dysregulate lysosomal acidification. TMEM9 interacts with V-ATPase to induce aberrant lysosomal acidification [23]. This aligns with evidence that both excessive and insufficient acidification impair lysosomal hydrolase activity and disrupt autophagic flux [58]. Crucially, our study reveals a novel interaction between TMEM9 and ATP6AP1, a key regulator of lysosomal acidification [59]. Second, TMEM9 may downregulate LAL, an enzyme essential for lysosomal neutral lipid hydrolysis [60]. Notably, LAL expression was reduced in AD brains but restored by physical exercise in our models. Third, TMEM9 may disrupt LD delivery to lysosomes—a process requiring integral lysosomal membrane fatty acid transporters or luminal protonation [61]. Finally, as a resident protein in late endosomes/lysosomes, TMEM9 may regulate chloride/proton exchangers critical for autophagy progression [62, 63].

Our results demonstrated that downregulation of TMEM9 promotes lipophagy by enhancing autophagic flux, as evidenced by increased ATG5 expression, elevated LC3II/LC3I ratio, augmented mCherry-GFP-LC3 puncta, and reduced P62 levels. In contrast, Baek et al. reported that TMEM9 activates Rab9-dependent alternative autophagy via Beclin1 interaction without affecting conventional LC3-dependent flux [64]. This discrepancy may be explained by two possible mechanisms. First, Beclin1 serves as a scaffold for the PI3KC3 complex, which regulates both canonical and alternative autophagy pathways [65]; thus, TMEM9 may dually modulate both pathways in a context-dependent manner. Second, TMEM9 may regulate LC3-dependent autophagy by modulating mTOR-Akt signaling [66, 67]. We previously demonstrated that physical exercise decreases TMEM9, which interacts with ATP6V0D1, thereby attenuating C1q dependent synaptic impairment in the AD brain [23]. In the present study, we found that physical exercise promotes microglial lipophagy through the TMEM9/ATP6AP1/AMPK axis. These two mechanisms may synergistically contribute to the neuroprotective effects of physical exercise. On one hand, ATP6AP1, as an essential adaptor of the V-ATPase complex, has been reported to interact with the proton channel subunit ATP6V0D1 to regulate V-ATPase-dependent acidification, which is involved in C1q activation [23, 68]. On the other hand, ATP6V0D1, as the ATPase H⁺ transporting V0 subunit d1, has been reported to regulate mTORC1 activation [69], which is involved in the regulation of lipophagy [70]. It is noteworthy that TMEM9b, a related homolog of TMEM9, was also downregulated by physical exercise in our proteomics results. Both TMEM9 and TMEM9b localize to lysosomes and endosomes, regulating the activity of the inflammatory response [71, 72]. Therefore, the possibility that TMEM9b may play overlapping roles with TMEM9 in the AD brain cannot be excluded and should be explored in future studies.

Our findings demonstrate that LDs can exert either protective or detrimental effects depending on the lipophagy status. Inhibition of lipophagy, whether induced by chloroquine treatment or *Tmem9* overexpression, shifts LDs toward a pathogenic state. This shift is evidenced by elevated reactive oxygen species, increased pro-inflammatory cytokine production, and reduced expression of electron transport chain proteins in microglia. These results align with prior reports that microglial LDs confer neuroprotection by shielding adjacent neural stem cells and neurons from lipotoxicity, while also mitigating endoplasmic reticulum stress [61]. Conversely, lipophagy dysfunction impairs LD turnover and is directly implicated in human neurodegeneration [73]. It is noteworthy that the alterations in key mitochondrial electron transport chain proteins detected in our proteomic analysis (Fig. 1), including NDUFB10, MTCO2, and ATPAF1, were further validated by Western blotting (Fig. 3). However, it remains unclear whether the observed effects on mitochondrial lipid metabolism and mitochondrial function occur exclusively in microglia or in both microglia and neuronal cells, which is a limitation of the present study. To address this question in future investigations, fluorescence-activated cell sorting should be employed to isolate microglia and neurons from mouse brain homogenates, followed by quantitative proteomics or western blotting for these proteins, in order to delineate the cell-type-specific contributions. Furthermore, we exclusively used male mice in this study to minimize variability arising from sex-dependent hormonal fluctuations and their subsequent impact on neuroinflammation and microglial activation [74, 75]. Since female 5×FAD mice are known to exhibit accelerated and more severe amyloid pathology compared to males [44, 54], using a single sex allowed us to isolate the therapeutic effects of our intervention without the confounding influence of disease progression rate. This is another limitation of the current study, and future investigations incorporating both sexes are warranted.

Critically, our study demonstrates that microglial *Tmem9* downregulation promotes lipophagy via AMPK signaling. Both physical exercise and RNAi-*Tmem9* effectively phosphorylated AMPKα and enhanced lipophagy. Notably, Compound C abolished the protective effects of *Tmem9* knockdown, redirecting LDs toward lipotoxicity. We propose that *Tmem9* downregulation activates AMPKα through lysosomal translocation of AXIN and LKB1, based on two key observations: LKB1 phosphorylates AMPK during energy stress, while AXIN scaffolds LKB1 to amplify its kinase activity [76]. Second, overexpressed TMEM9 binds the ATP6AP1 subunit of V-ATPase, enhancing its activity and suppressing lysosomal AMPK signaling, which is consistent with the evidence that V-ATPase inhibition activates lysosomal AMPK independent of the AMP/ADP levels [39]. Conversely, physical exercise downregulates TMEM9 to activate AMPK. Crucially, our imaging data confirmed increased LAMP1-co-localization with lysosomal membrane-bound LKB1 and AXIN. This directly aligns with prior reports that V-ATPase inhibition drives AXIN/LKB1 translocation to lysosomal membranes for phosphorylation-dependent AMPK activation [38].

**Fig. 10:**
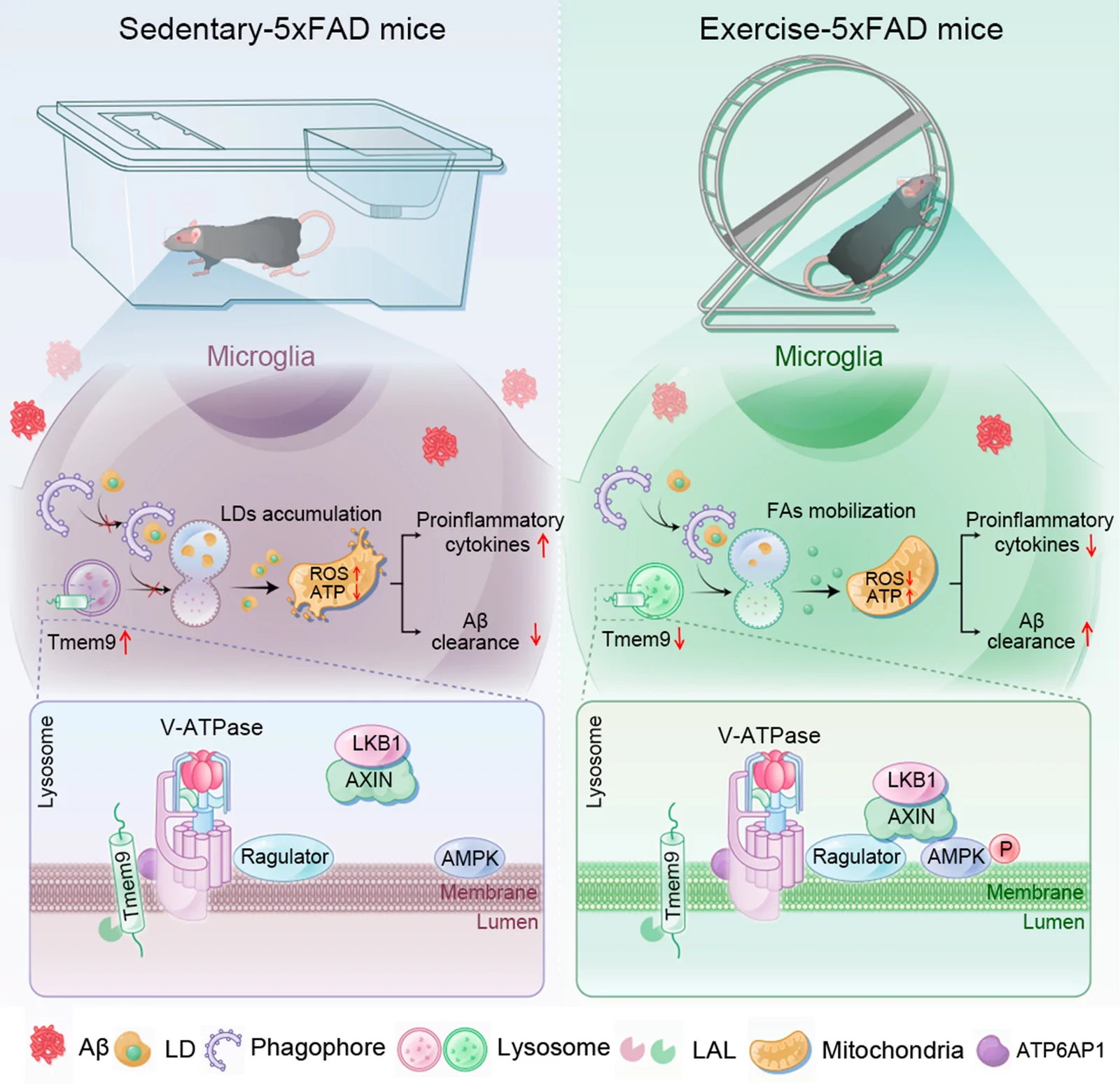
Schematic illustrating that physical exercise promotes microglial lipophagy and mitigates AD pathologies via downregulating TMEM9. In the AD brain, microglial TMEM9 is upregulated, where it interacts with ATP6AP1 and suppresses LKB1 translocation, thereby inhibiting AMPK activation. This impairment disrupts the lipophagy-mediated lipid mobilization, leading to LD accumulation and mitochondrial dysfunction, exacerbating AD-related pathologies. Conversely, physical exercise downregulates TMEM9 expression and reduces ATP6AP1 levels, facilitating LKB1 lysosomal translocation and subsequent AMPK signaling activation. This cascade promotes lipophagy, enhances fatty acid (FA) transport into mitochondria, and boosts ATP production to sustain microglial phagocytic activity, ultimately contributing to the attenuation of AD-related pathological progression

In summary, our study identifies microglial TMEM9—through its interaction with ATP6AP1—as a potential diagnostic biomarker and therapeutic target for AD. TMEM9 upregulation induces lipophagy dysfunction, mitochondrial damage, impaired Aβ clearance, and cognitive decline. Critically, physical exercise counteracts this cascade by downregulating microglial TMEM9 expression, thereby promoting lipophagy, restoring LD homeostasis, attenuating mitochondrial injury, reducing Aβ deposition, and ultimately alleviating cognitive impairment (Fig. 10). To our knowledge, this is the first study establishing that the TMEM9–ATP6AP1 complex interfaces with the LKB1/AXIN/AMPK axis. This crosstalk underscores the dual role of TMEM9 in regulating both lysosomal function and cellular energy sensing, highlighting its promise as a therapeutic target for maintaining LD homeostasis in AD.

## Supplementary Information

Additional file 1 **Fig. S1**. Physical exercise (PE) enhanced microglial lipophagy in 5×FAD mice. **Fig. S2**. Verification of adeno-associated lentivirus transfection. **Fig. S3**. Physical exercise enhanced microglial lipophagy in 5×FAD mice by down-regulating microglia TMEM9. **Fig. S4**. Transfection of lentivirus did not affect the LDs formation induced by Oleic Acid (OA) in BV2 cells. **Fig. S5**. Knockdown of *Tmem9* promoted autophagy, increased mitochondrial proteins and decreased pro-inflammatory cytokines induced by oAβ in BV2 Cells under glucose-insufficiency conditions, whereas overexpression of *Tmem9* exerted opposing effects. **Fig. S6**. Knockdown of *Tmem9* promoted autophagy, increased mitochondrial protein and decreased pro-inflammatory cytokines induced by oAβ in BV2 Cells under glucose-insufficiency conditions, whereas chloroquine treatment diminished its effects. **Fig. S7**. Knockdown of *Tmem9* promotes LKB1 translocation to lysosome in vitro. **Fig. S8**. Concanamycin A (Con A) treatment reactivated AMPKα signaling, maintained mitochondrial homeostasis, and reduced pro-inflammatory cytokines in BV2 cells with *Tmem9* overexpression following oleic acid (OA) and oAβ incubation. **Fig. S9**. AAV injection itself had no effect on motor capacity in behavioral tests. **Fig. S10**. CSF TMEM9 levels are negatively correlated with SUVR on ^18^F-FDG PET-CT. **Table S1**. Antibodies for IF and WB. **Table S2**. Characteristics of control and AD patients cohort. **Table S3**. Correlation analyses between biomarkers and ^18^F-AV45 PET-CT. **Table S4**. Correlation analyses between biomarkers and ^18^F-FDG PET-CT.

Additional file 2 Differentially expressed proteins in the Z-score dataset

Additional file 3 Original blots

## Data Availability

The data that support the findings of this study are available from the corresponding author upon reasonable request.
