# Supplementary material for: Exercise promotes lipophagy and mitigates cognitive impairment in Alzheimer’s disease through downregulating microglial TMEM9
Source: Transl Neurodegener. 2026 Oct 19;15:56. doi: 10.1186/s40035-026-00583-z (PMC13679003; doi:10.1186/s40035-026-00583-z)

Figure 1.

Lanes within the red dotted boxes, arranged left to right: WT group, Control-5xFAD group, PE-5xFAD group.

Figure 1b. Lal, LC3II/LC3I

Sample 1 Lipa-cortex

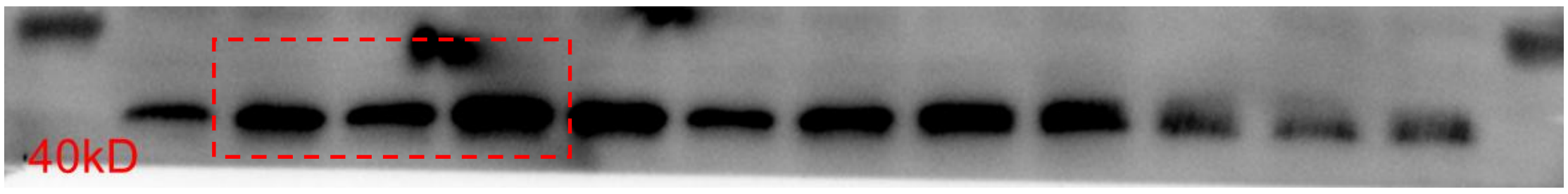

Sample 1 Gapdh

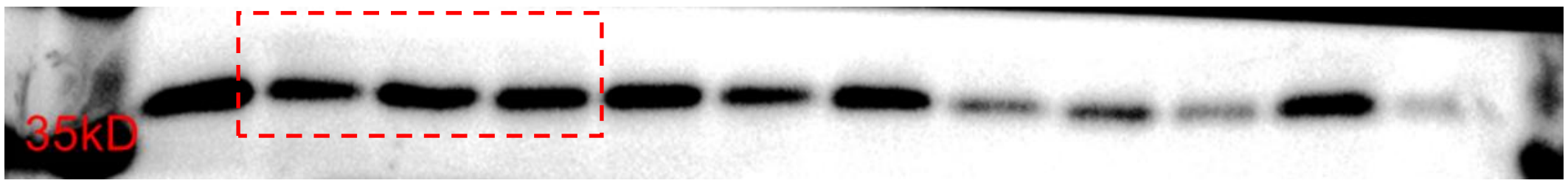

Sample 2+Sample 3 Lal-cortex

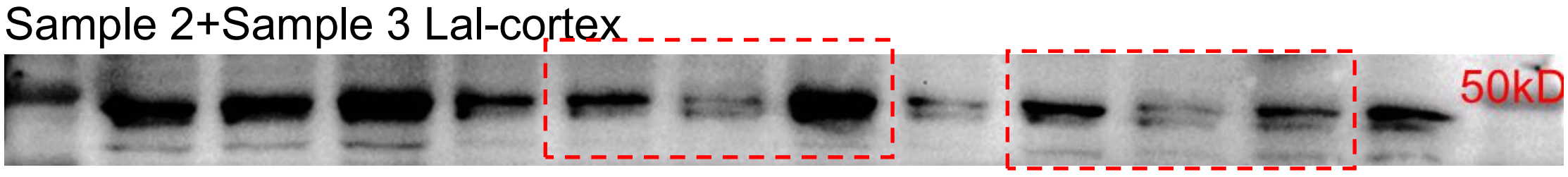

Sample 2+Sample 3 Gapdh-cortex

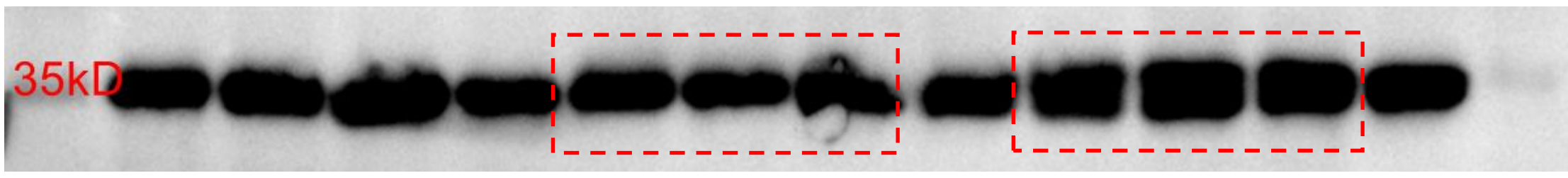

Sample 1+Sample 2 Lal-Hippo

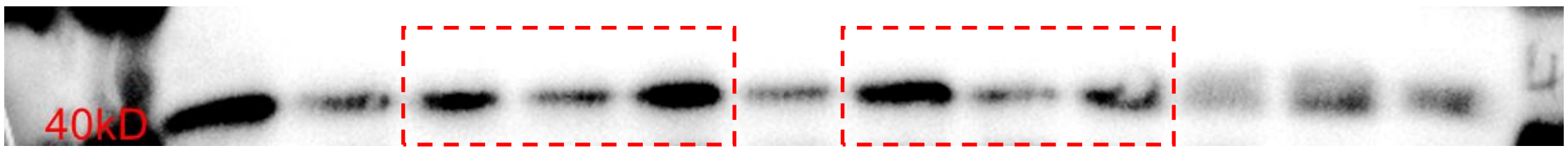

Sample 1+Sample 2 Gapdh-Hippo

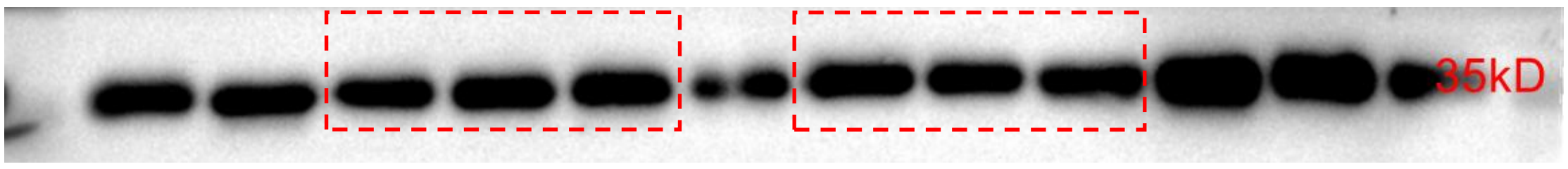

Sample 3 Lal-Hippo

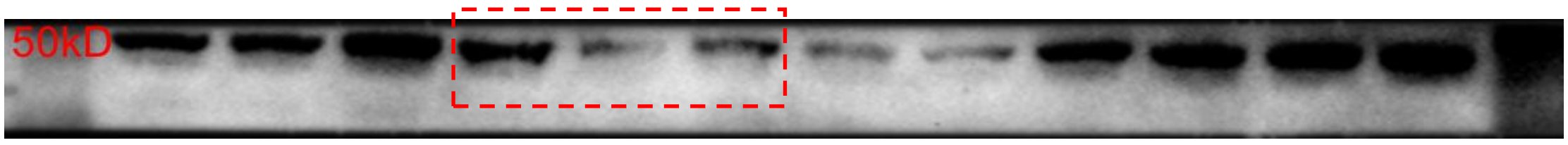

Sample 3 Gapdh-Hippo

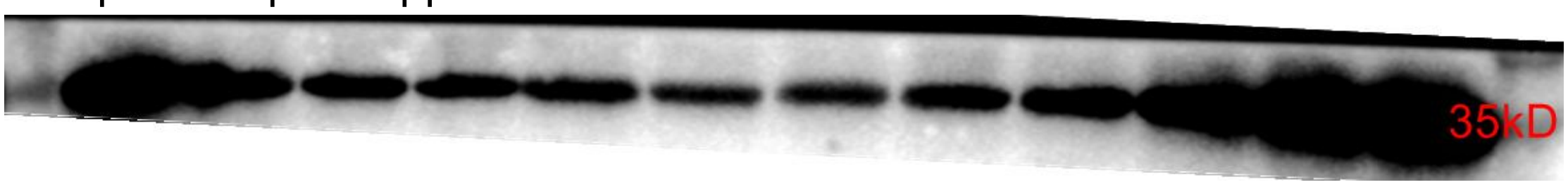

LC3II/LC3I

Sample 1 Lc3II/Lc3I cortex

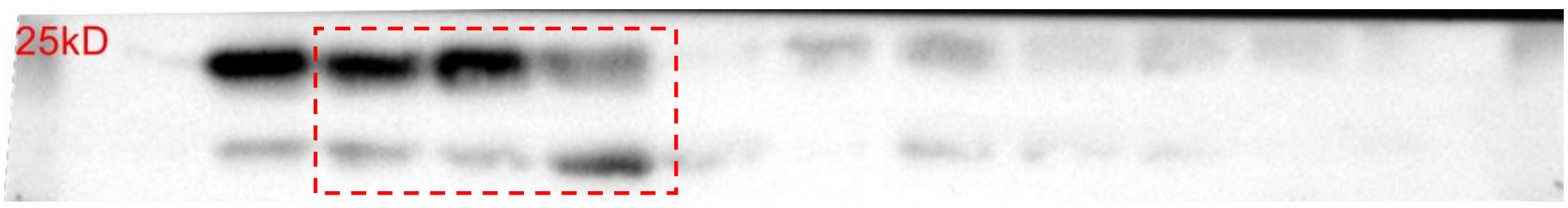

Sample 1 Gapdh cortex

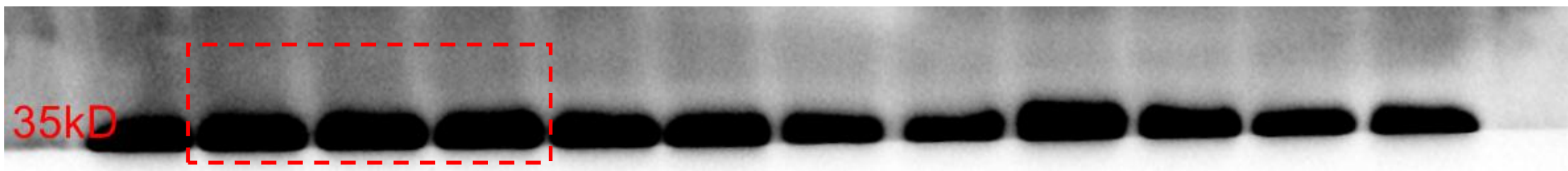

Sample 1+Sample 2 Lc3 Hippo

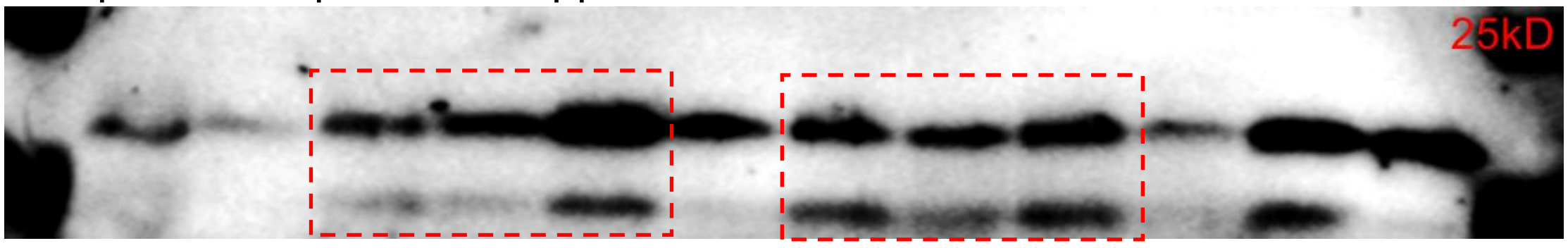

Sample 1+Sample 2 gapdh Hippo

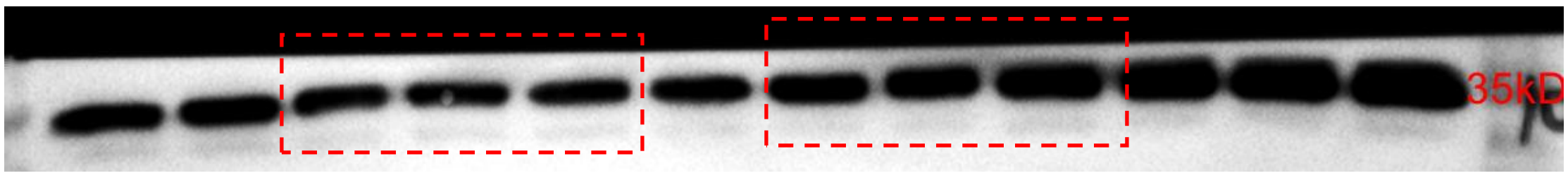

Figure 1 f.Tmem9

Sample 1+Sample 1 Tmem9 cortex+Hippo

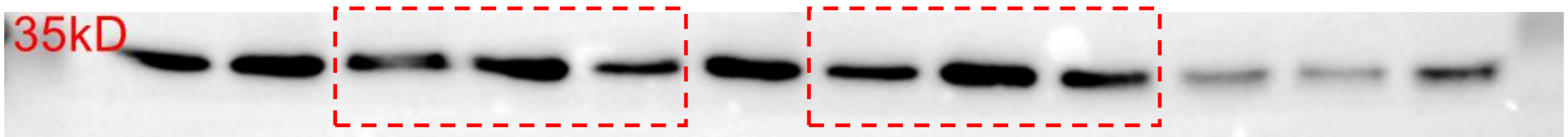

Sample 1+Sample 1 β-Tubulin cortex+Hippo

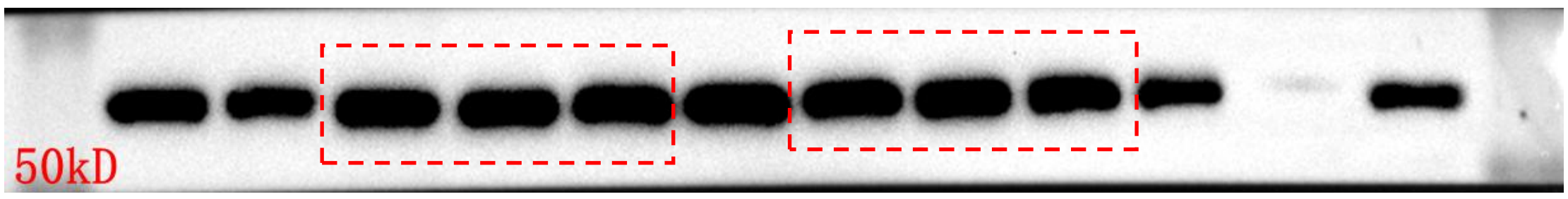

Sample 2+Sample 2 Tmem9 cortex+Hippo

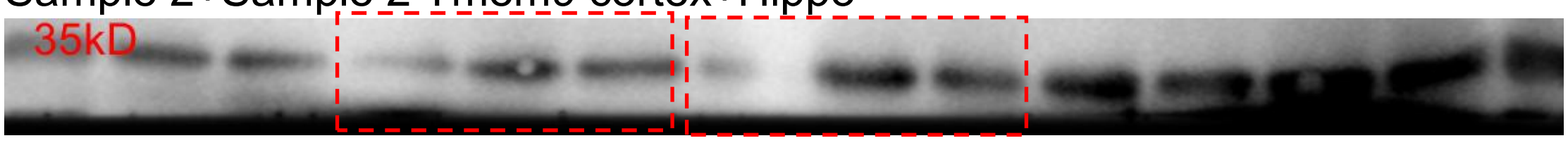

Sample 2+Sample 2 β-Tubulin cortex+Hippo

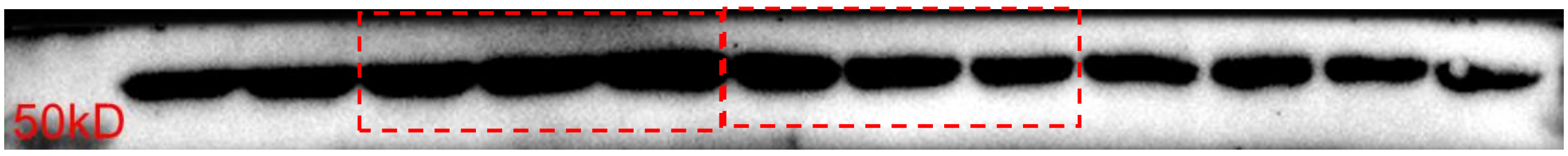

Sample 3+Sample 2 Tmem9 cortex+Hippo

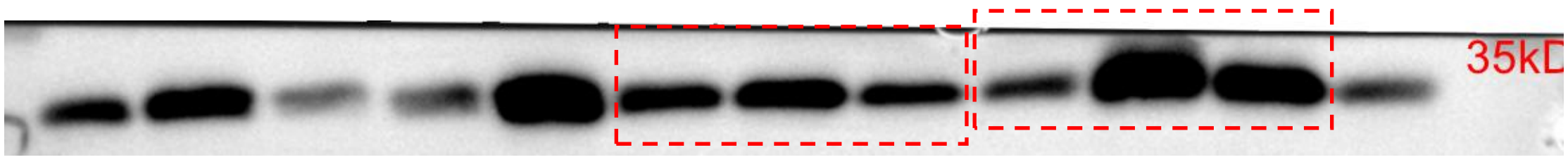

Sample 3+Sample 3 β-Tubulin cortex+Hippo

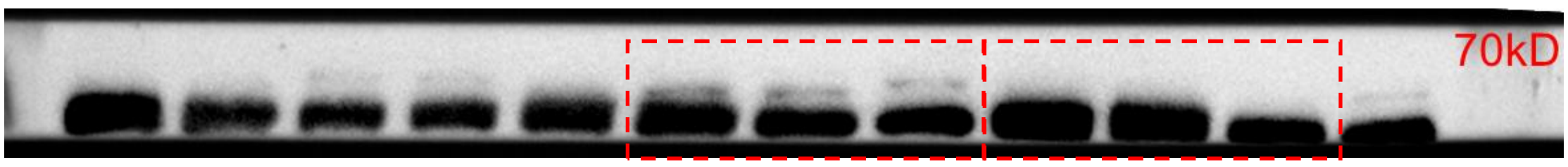

Figure 2.

Lanes within the red dotted boxes, arranged left to right: WT group, Control-5xFAD group, PE-5xFADgroup, RNAi-Tmem9-5xFAD group, PE+OE--5xFAD group.

Figure 2.b Atg5, P62,LC3II/LCI and LAL

Atg5

Sample 1- Atg5-cortex

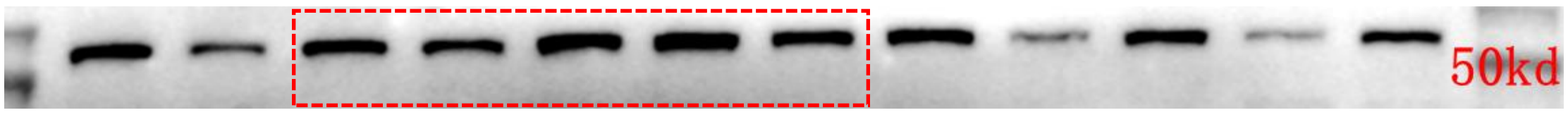

Sample 1- Gapdh-cortex

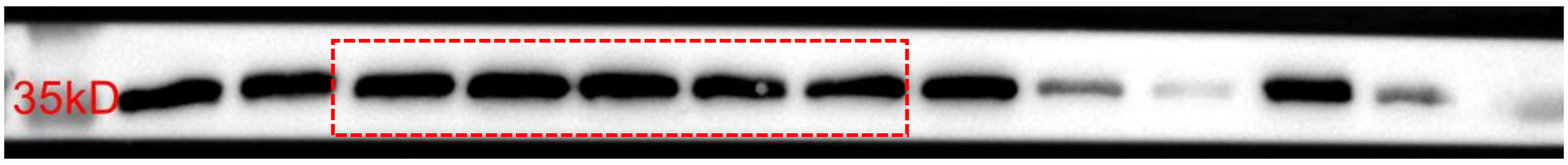

Sample 2- Atg5-cortex

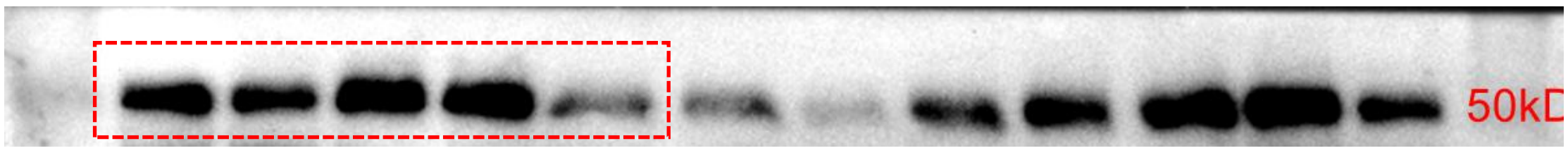

Sample 2- Gapdh-cortex

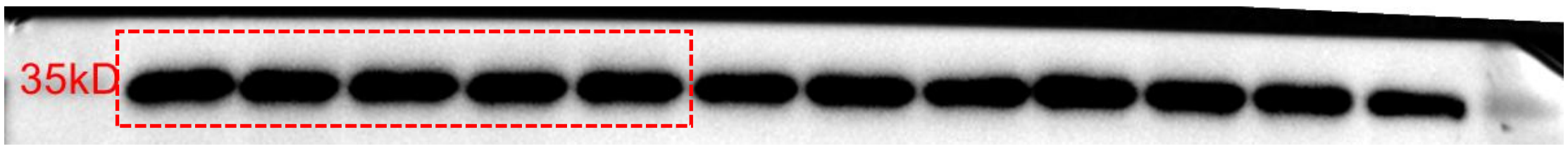

Sample 3- Atg5-cortex

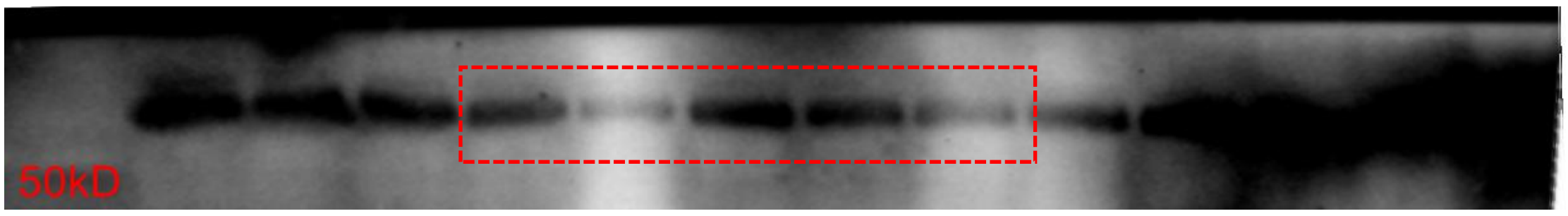

Sample 3- Gapdh-cortex

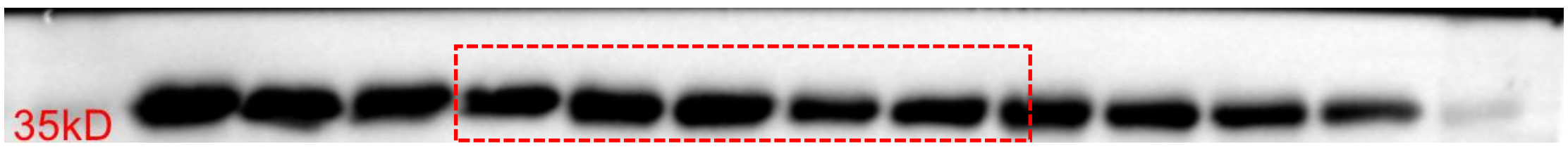

Sample 4- Atg5-cortex

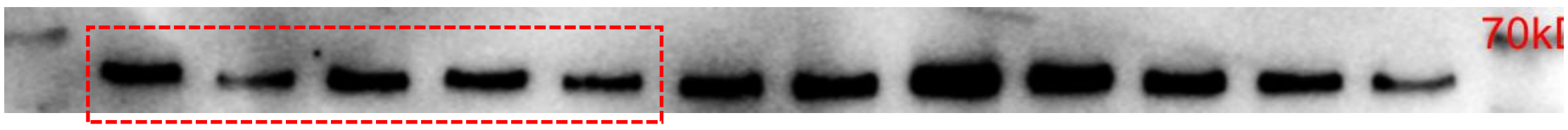

Sample 4- Gapdh-cortex

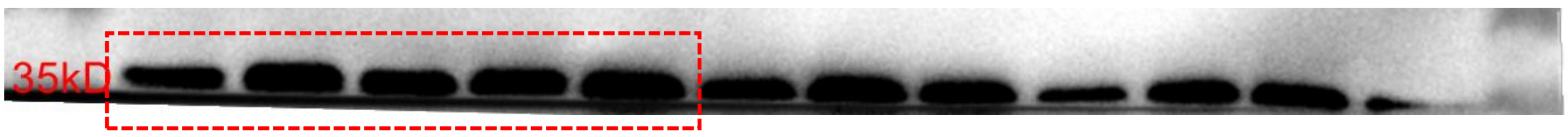

Atg5-Hippocampus

Sample 1- Atg5 Hippo

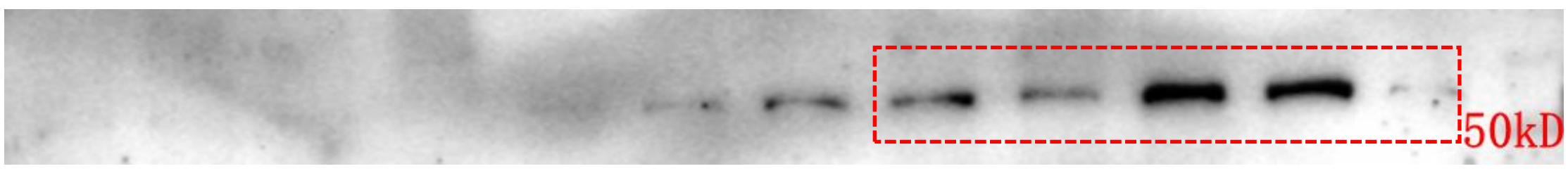

Sample 1-Gapdh-Hippo

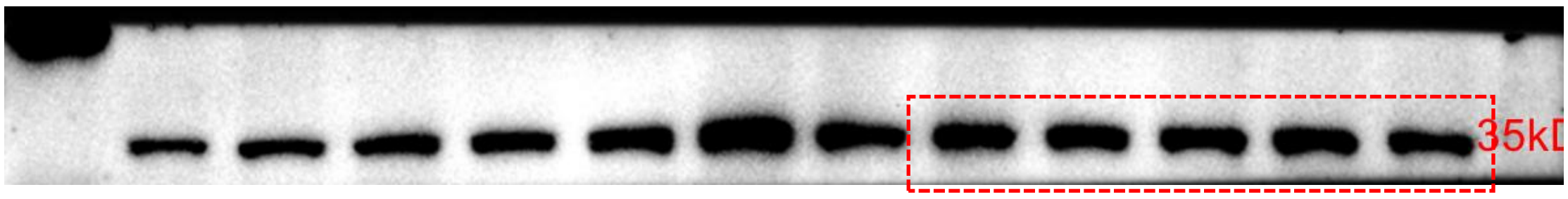

Sample 2- Atg5 Hippo

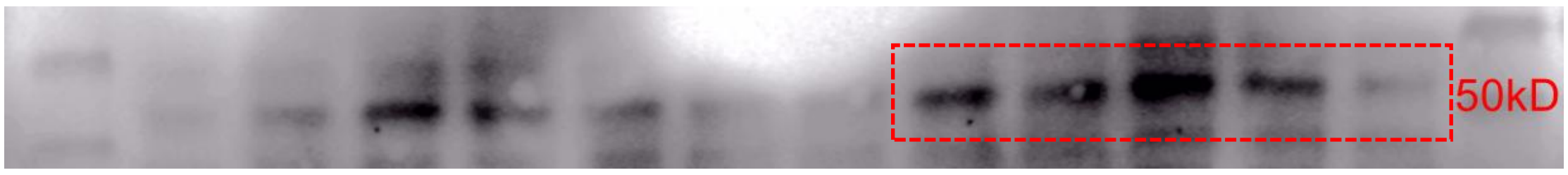

Sample 2- Gapdh Hippo

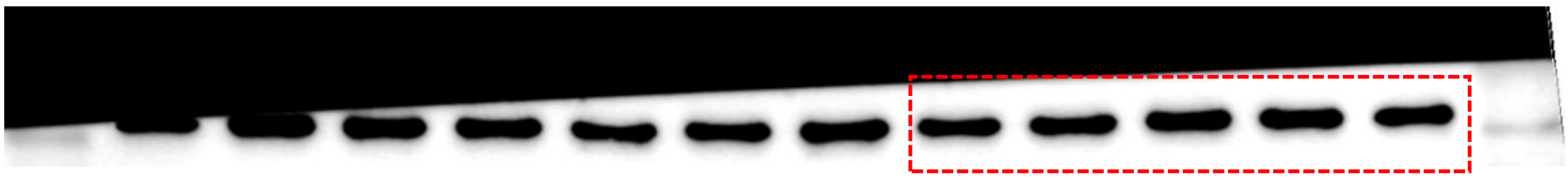

Sample 3- Atg5 Hippo

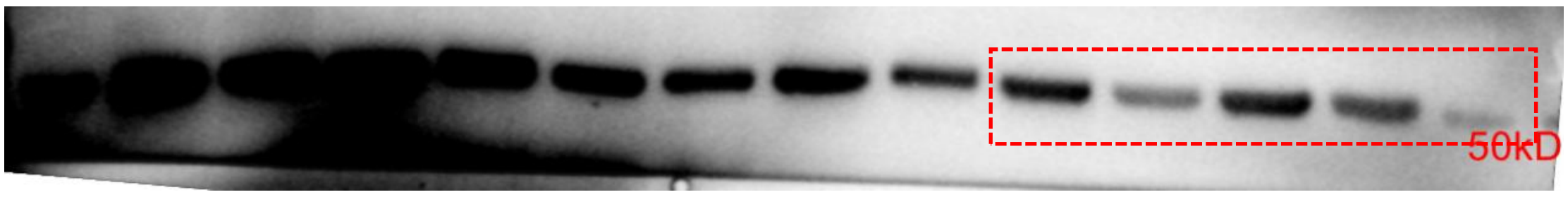

Sample 3- Gapdh Hippo

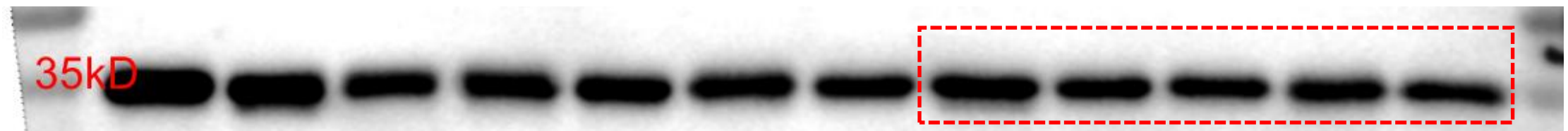

Sample 4- Atg5 Hippo

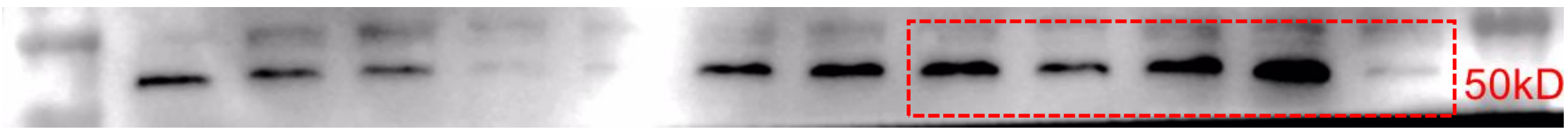

Sample 4- Gapdh Hippo

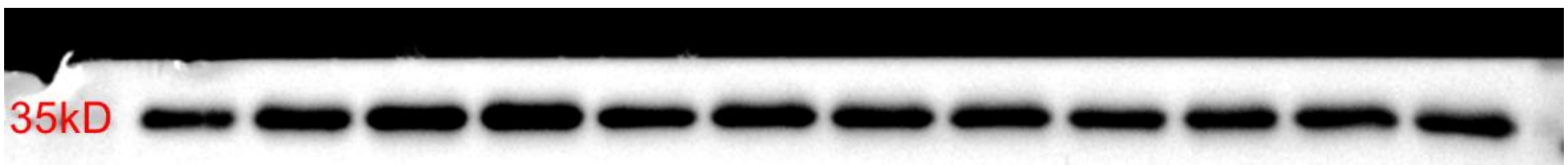

P62

Sample-1 P62 Cortex

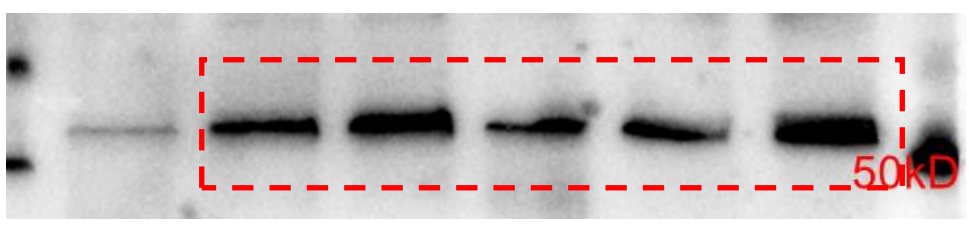

Sample-1 Gapdh Cortex

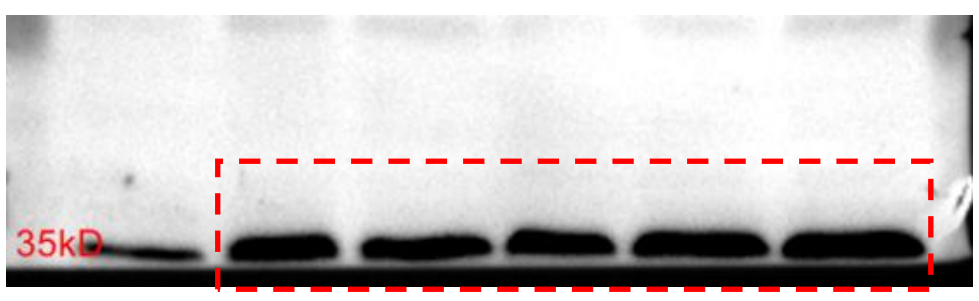

Sample-2 P62 Cortex

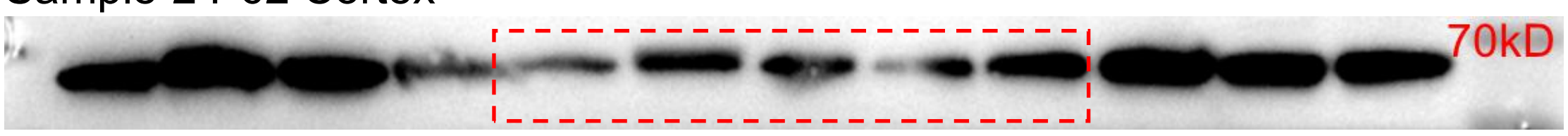

Sample-2 β -actin Cortex

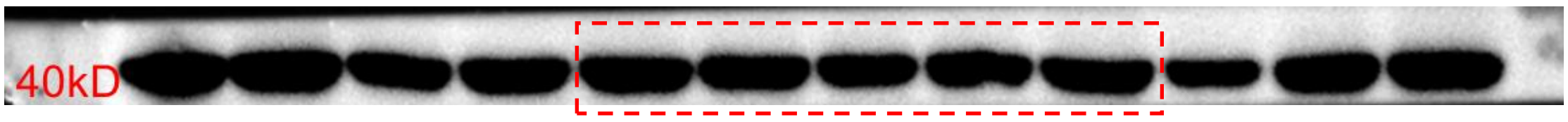

Sample-3+Sample 4 P62 Cortex

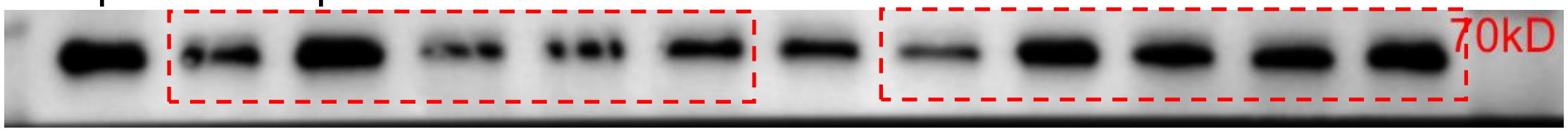

Sample-3+Sample 4 β-actin Cortex

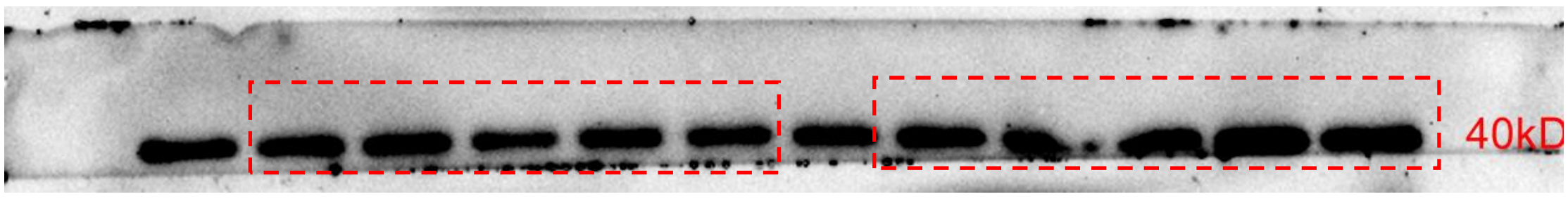

Hippocampus

sample-1 P62 Hippo

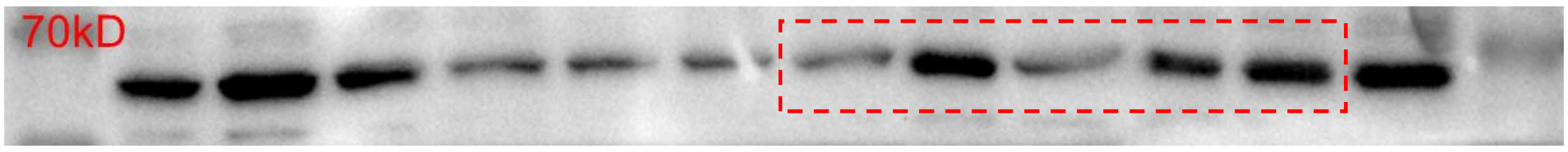

sample-1 GapdhHippo

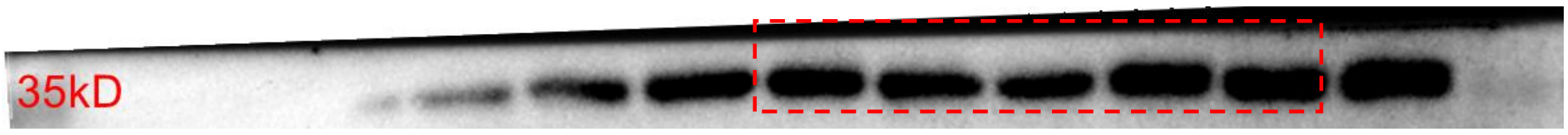

sample-2 P62 Hippo

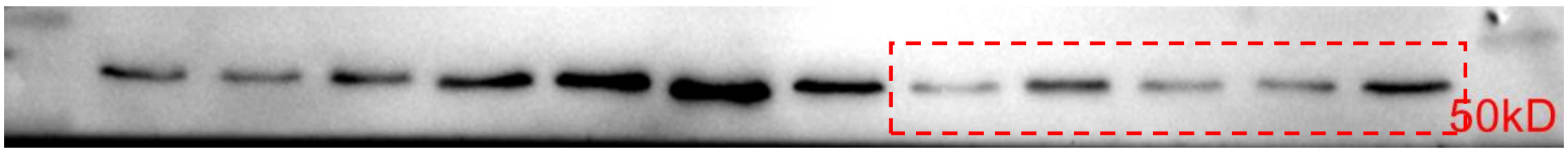

sample-2 β-actin Hippo

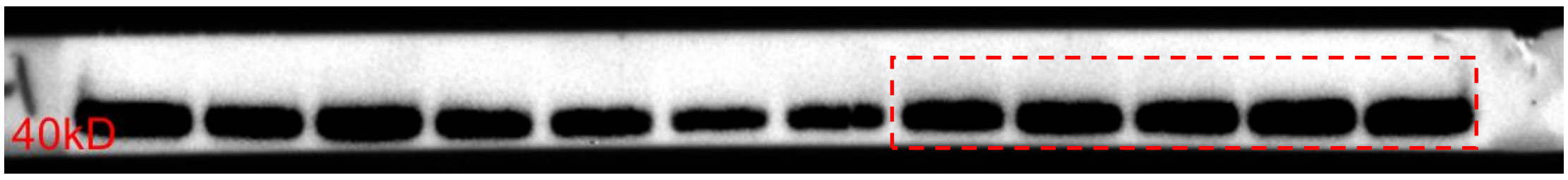

sample-3 P62 Hippo

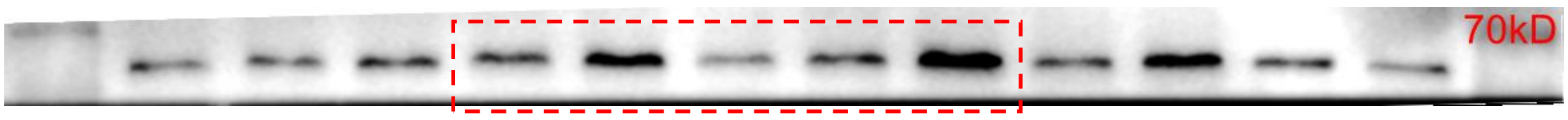

sample-3 β-actin Hippo

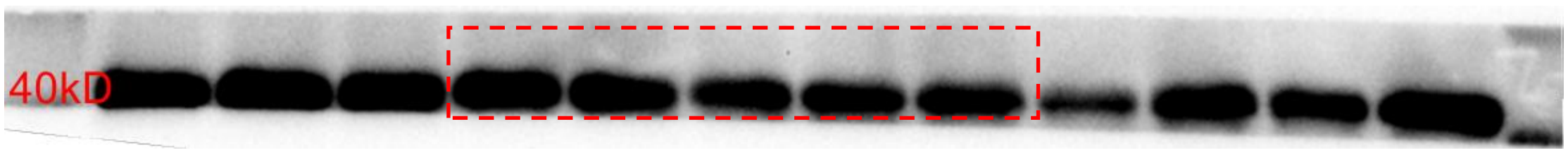

sample-4 P62 Hippo

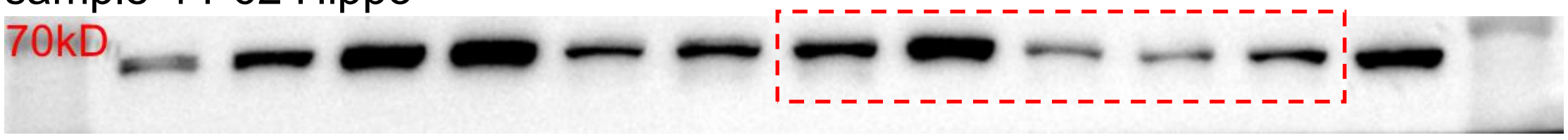

sample-4 β-actin Hippo

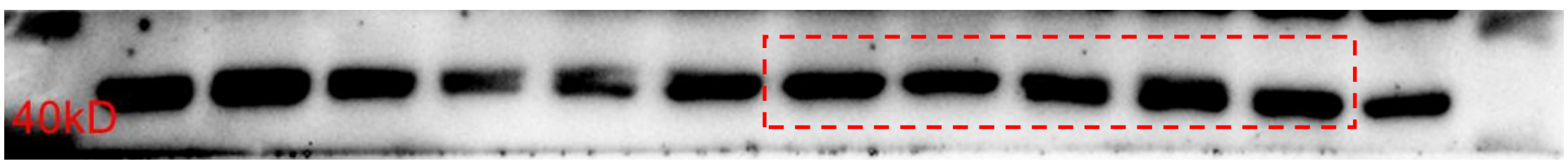

LC3

Sample-1 Lc3II/I. cortex

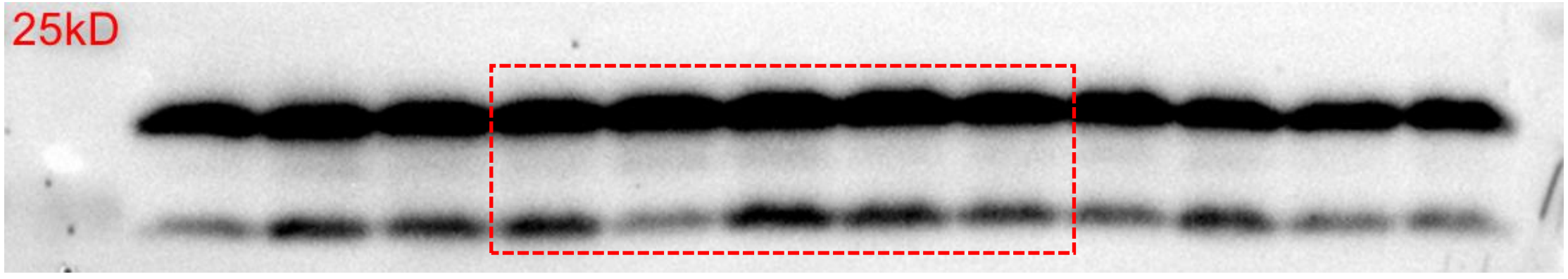

Sample-2 Lc3II/I. cortex

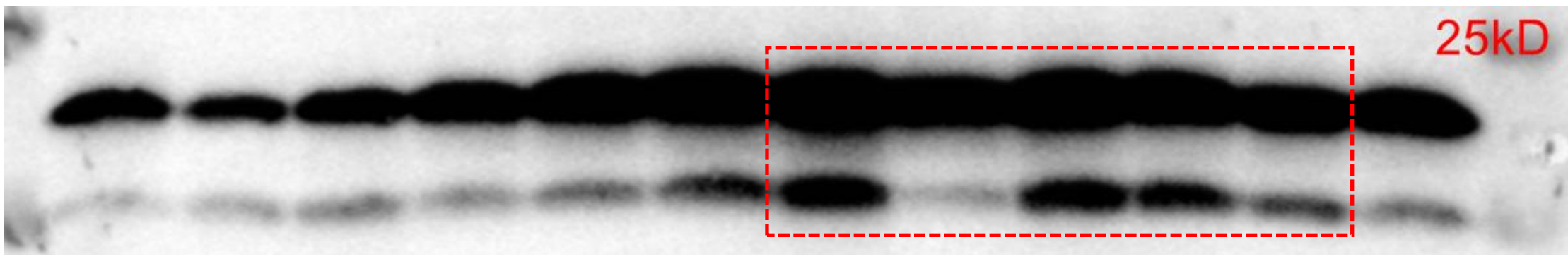

Sample-2 β-actin cortex

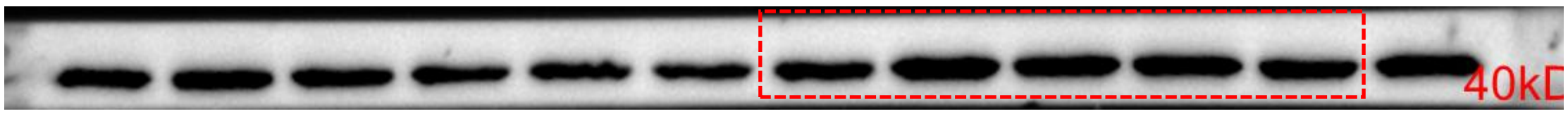

Sample-3 Lc3II/I. cortex

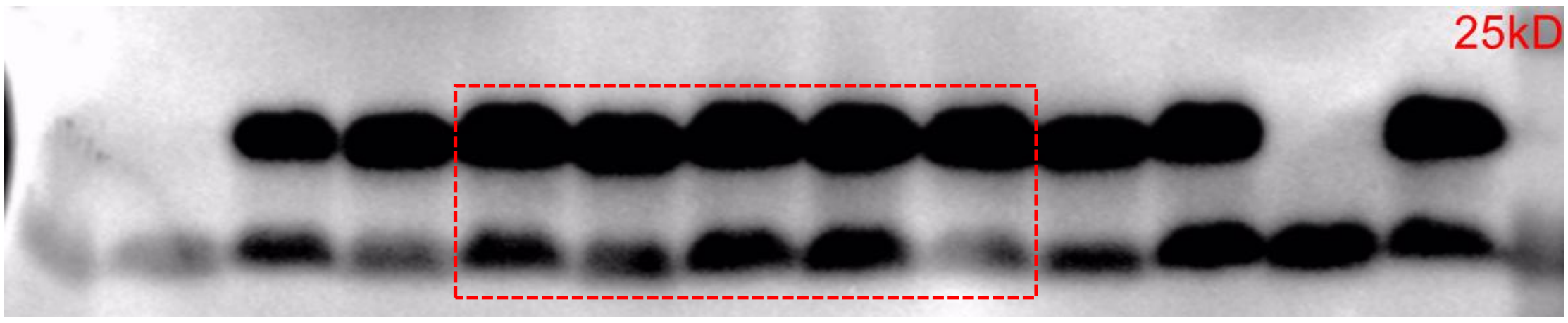

Sample-4 Lc3II/I. cortex

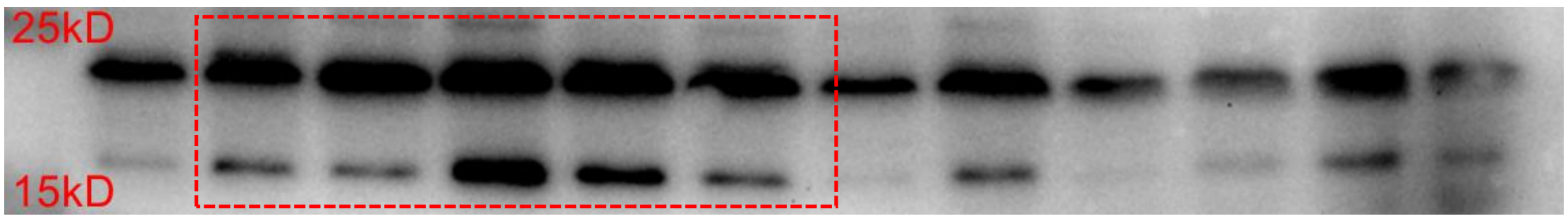

Sample-1 Lc3II/I. Hip

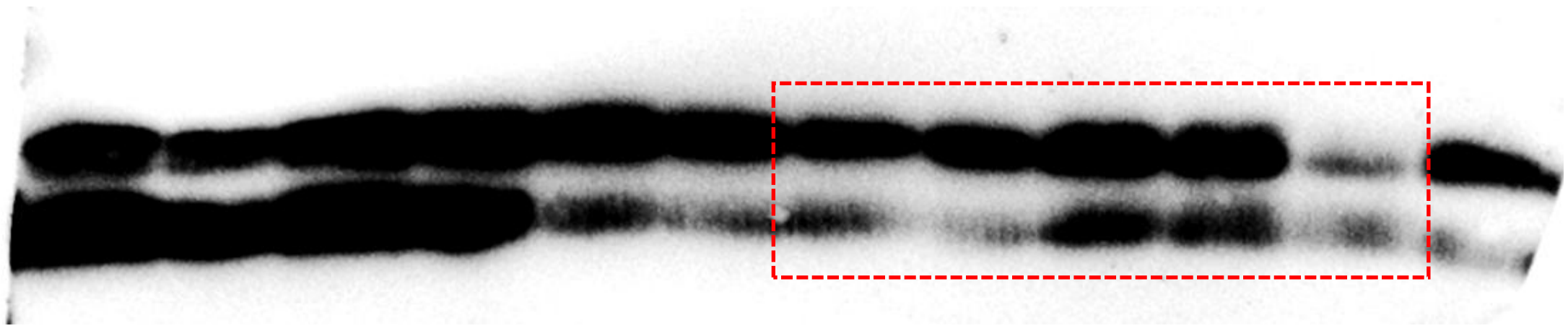

Sample-1 β-actin-Hip

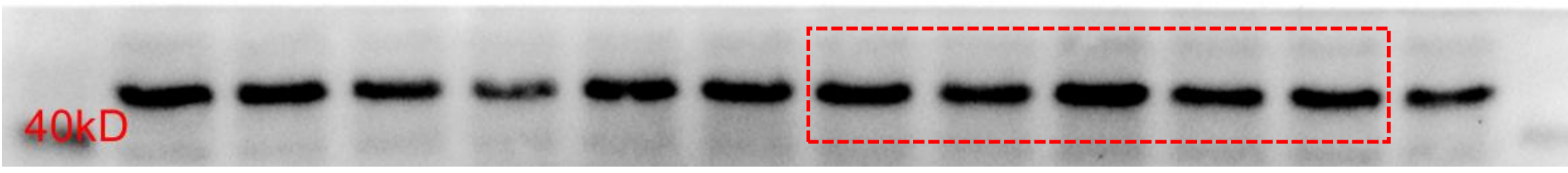

Sample-2 Lc3II/I. Hip

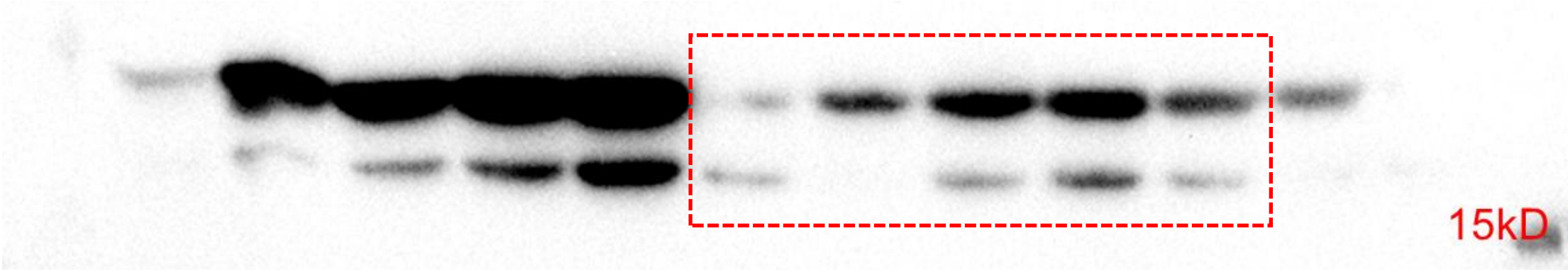

Sample-3 Lc3II/I. Hippo

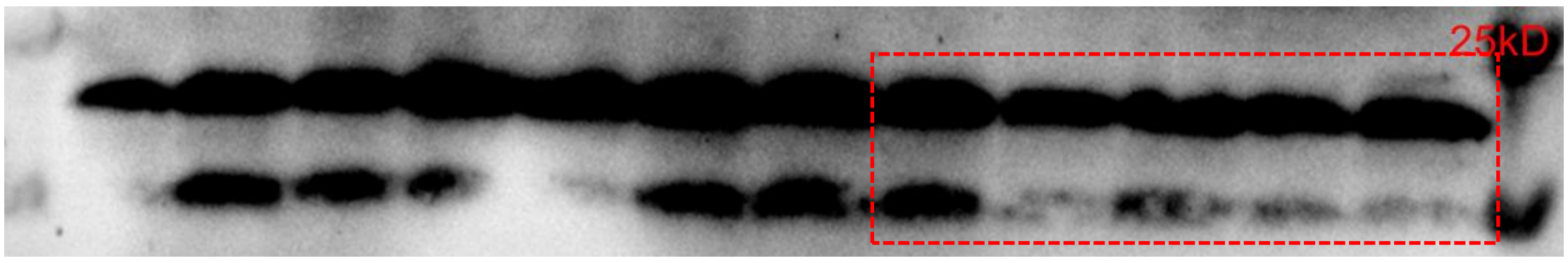

Sample-4 Lc3II/I. Hippo

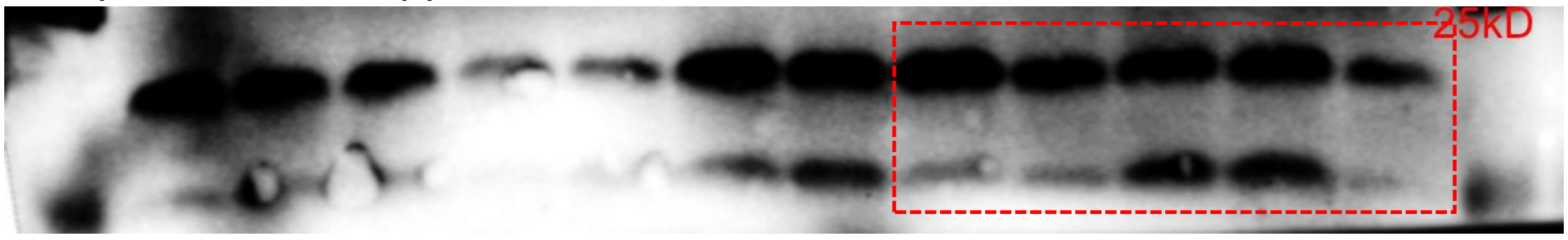

Lal

Sample -1 Lal-Cortex

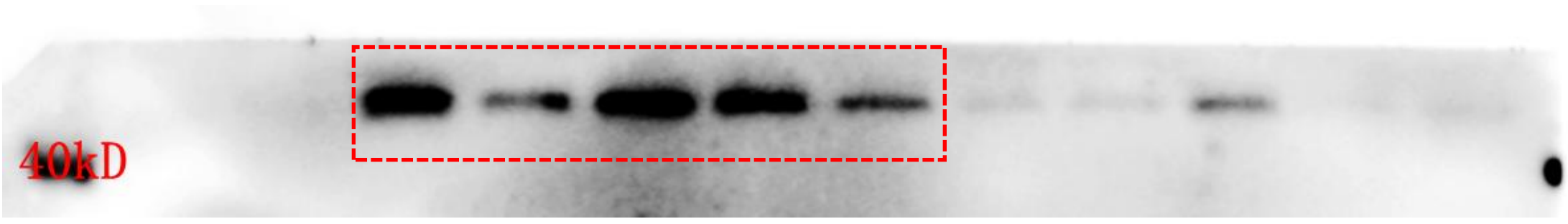

Sample -1 β-Tubulin-cortex

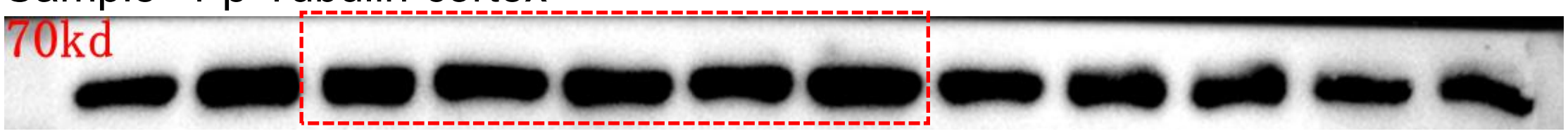

Sample -2 Lal-Cortex

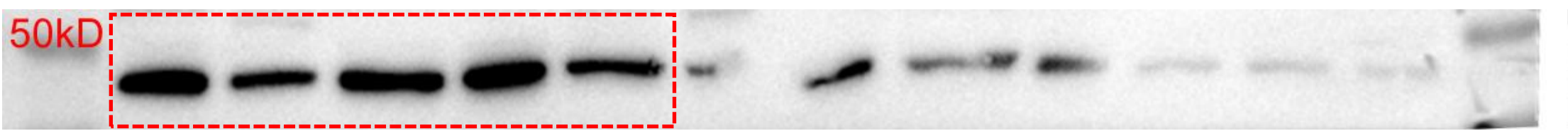

Sample -2 β tubulin-Cortex

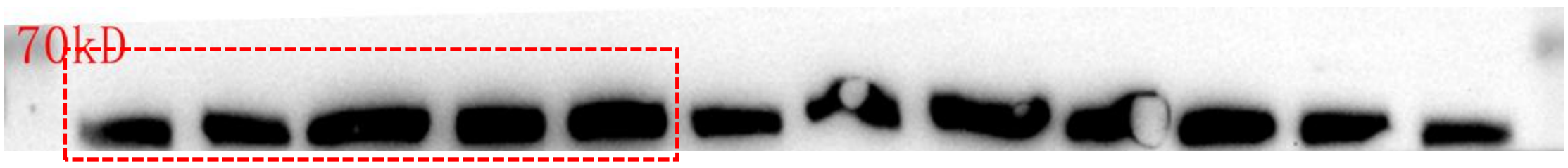

Sample -3 Lal-Cortex

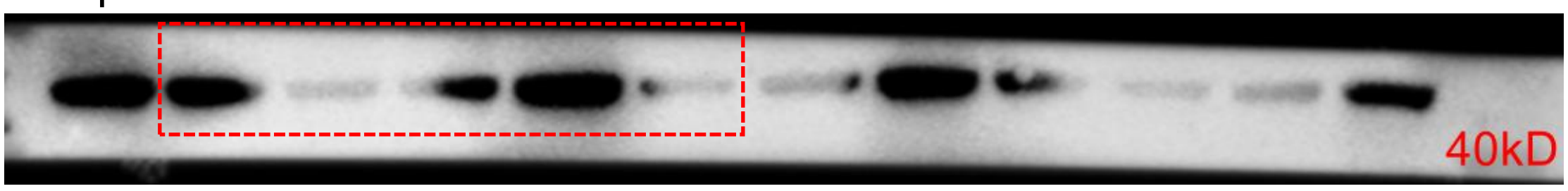

Sample -3 β tubulin-Cortex

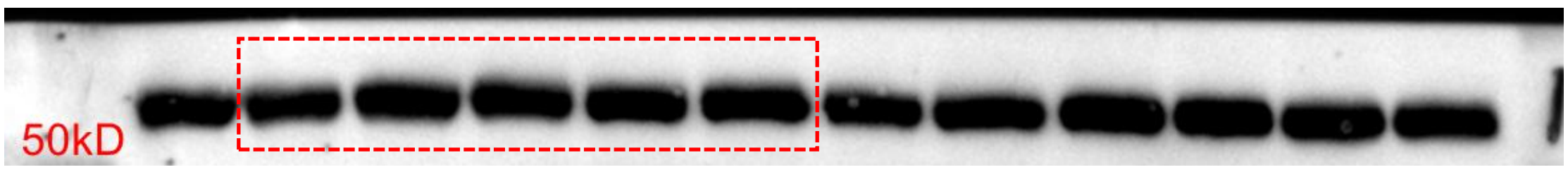

Sample -4 Lal-Cortex

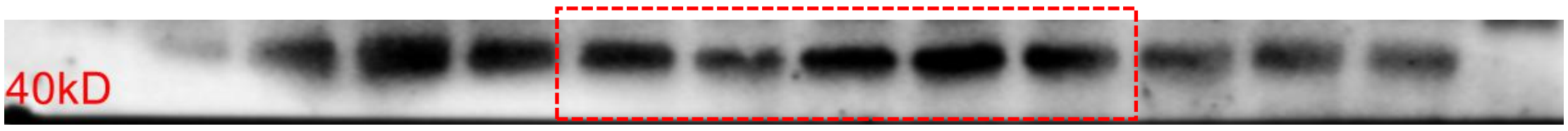

Sample -4 β tubulin-Cortex

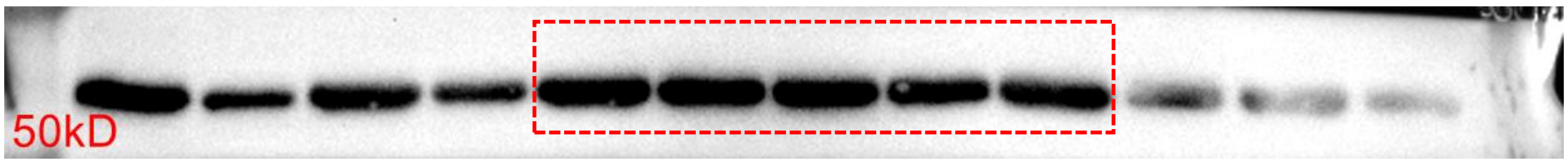

Sample -1 Lal-Hippo

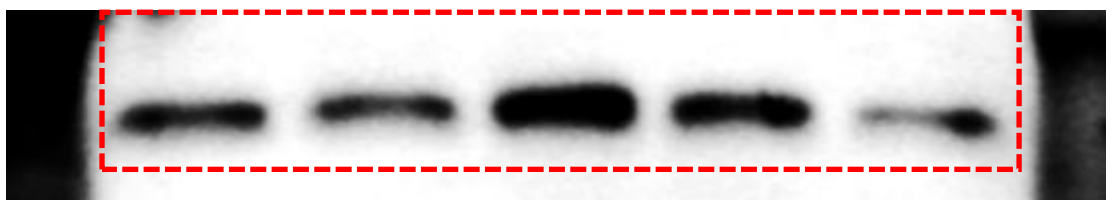

Sample -1 Tubulin-hip

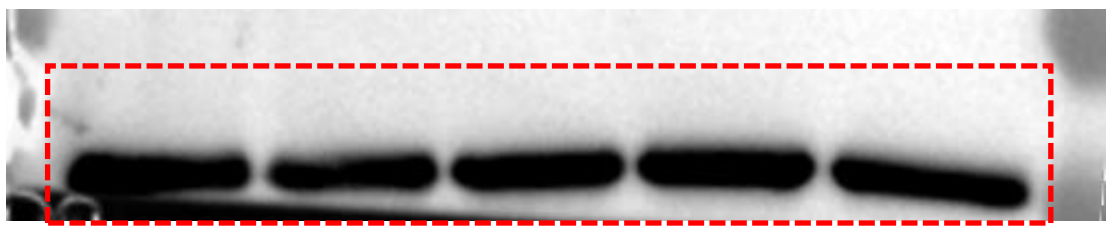

Sample -2 Lal-Hippo

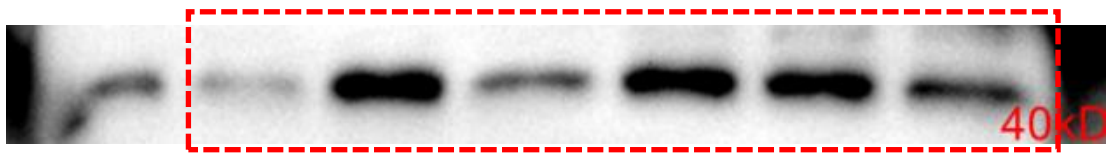

Sample -2 β-Tubulin-Hippo

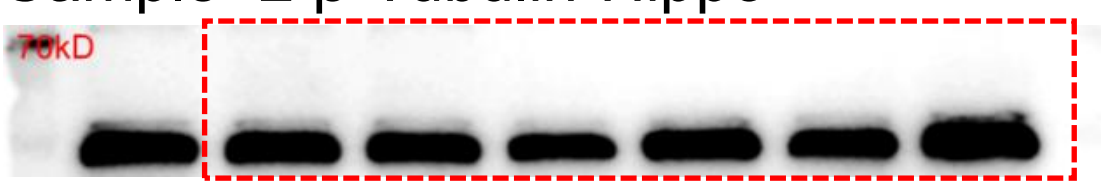

Sample -3 Lal-Hippo

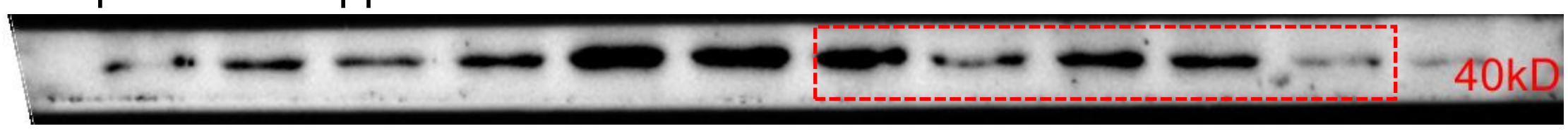

Sample -3 β-Tubulin-Hippo

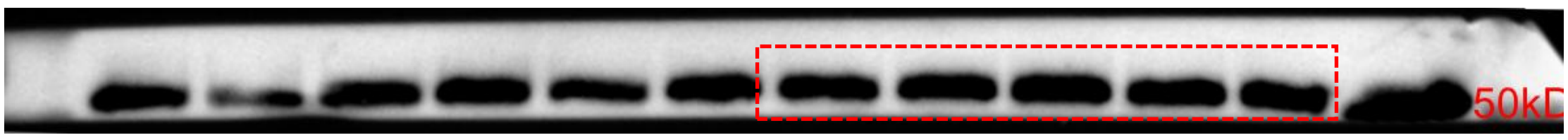

Sample -4 Lal-Hippo

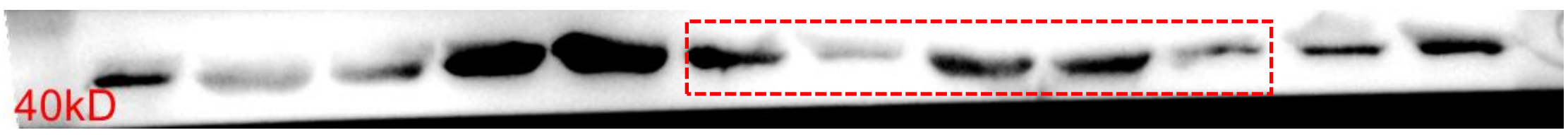

Sample -4 β-Tubulin-Hippo

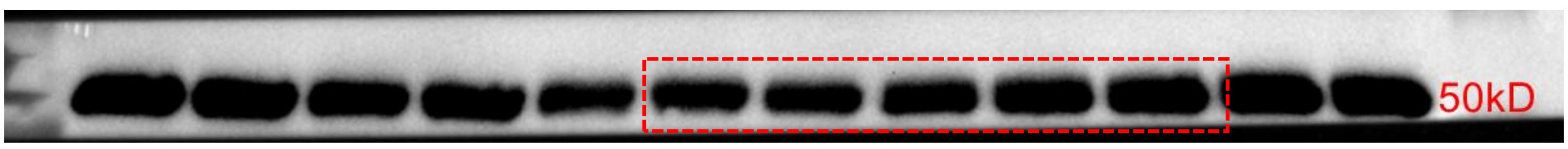

Figure 3.

Lanes within the red dotted boxes, arranged left to right: WT, Control-5xFAD, PE-5xFAD, RNAi-Tmem9, PE+OE-Tmem9.

Figure 3a. Cpt1a, Ndufb10

Cpt1a Cortex

Sample-1 Cpt1a -Cortex

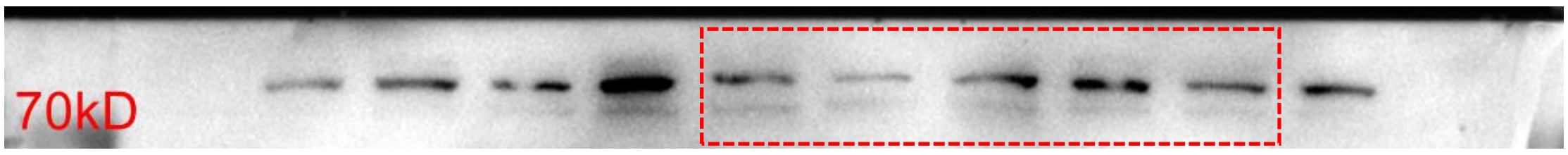

Sample-1 Gapdh-cortex

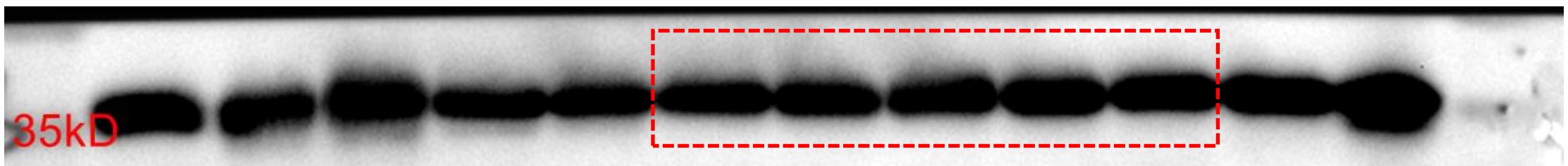

Sample-2 Cpt1a -Cortex

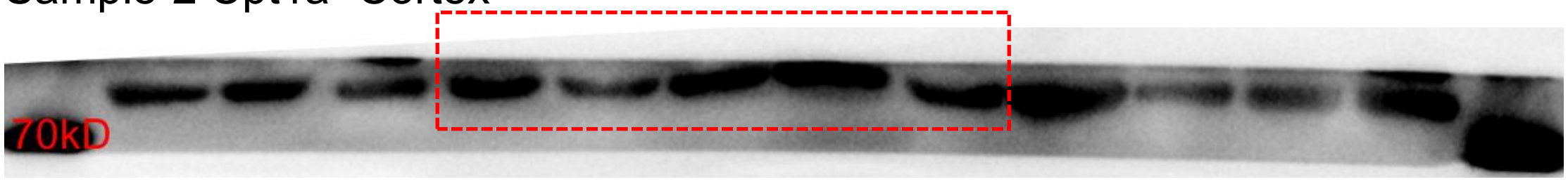

Sample-2 Gapdh-cortex

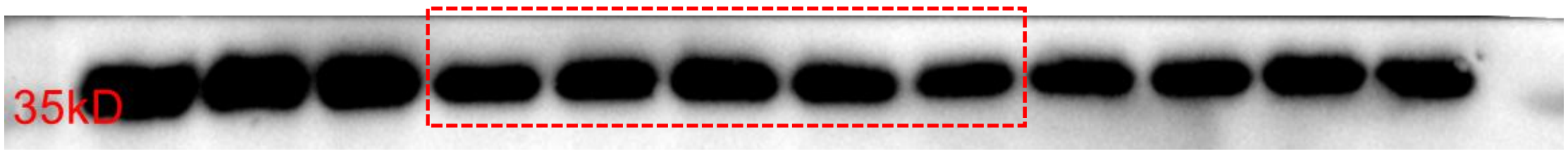

Sample-3 Cpt1a -cortex

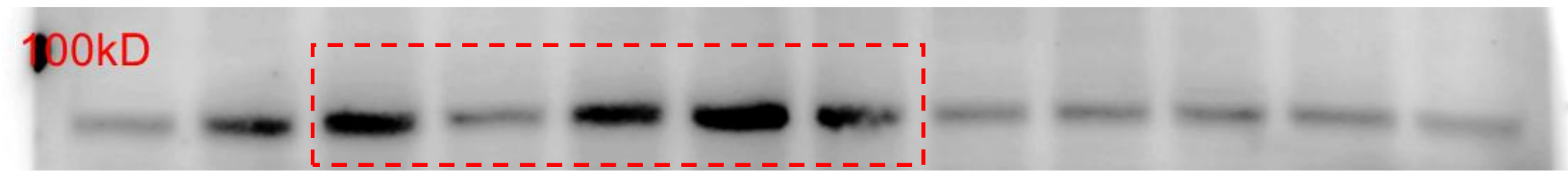

Sample-3 β-actin -cortex

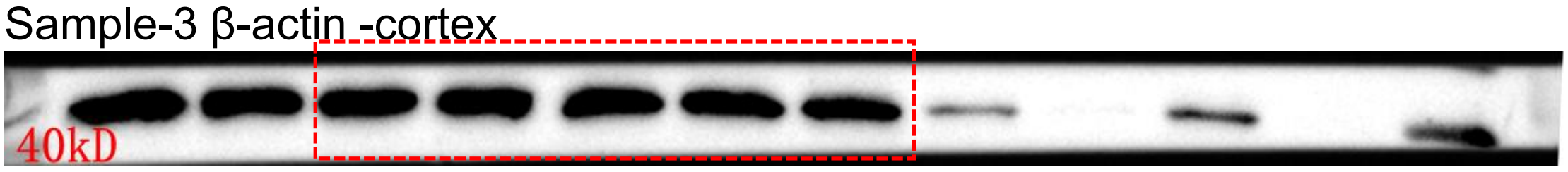

Sample-4 Cpt1a -Cortex

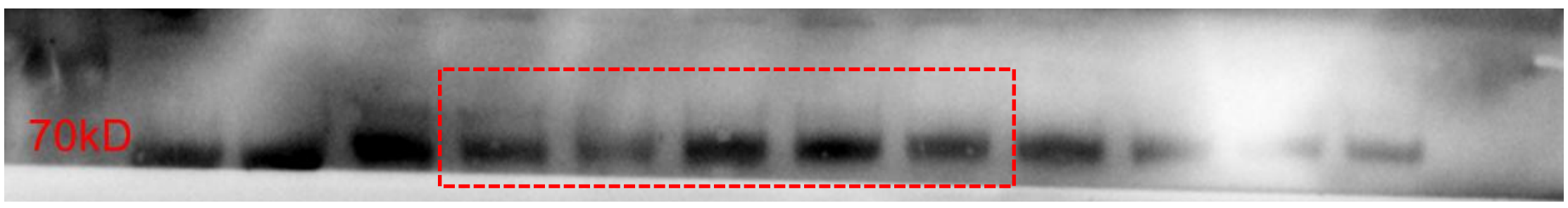

Sample-4 β-actin -cortex

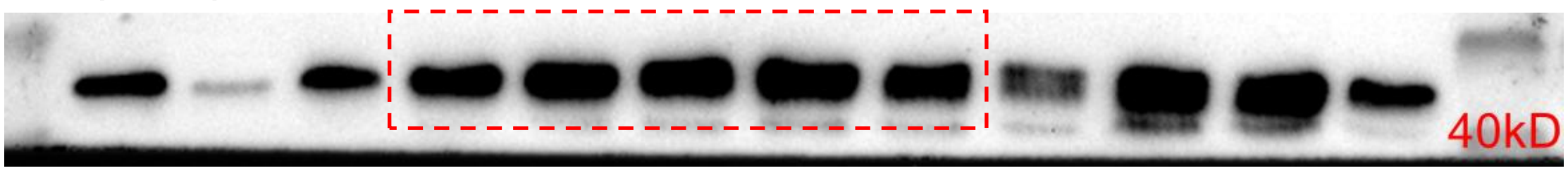

CPT1A Hippocampus

Sample-1 Cpt1a -Hippo

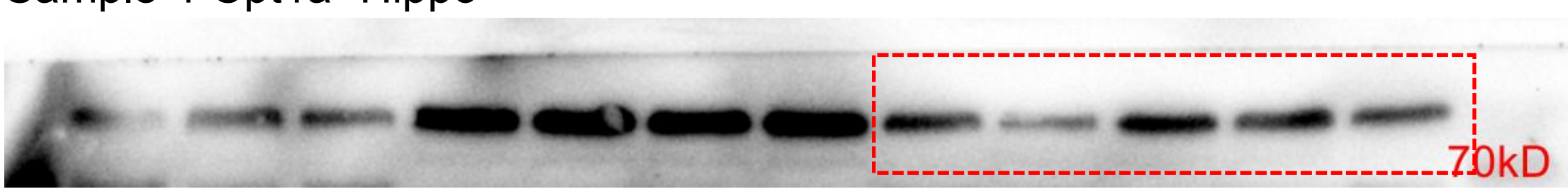

Sample-1 Gapdh-Hippo

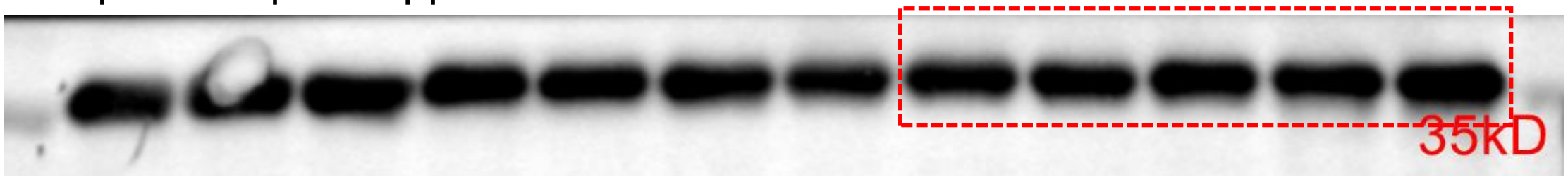

Sample-2 Cpt1a -Hippo

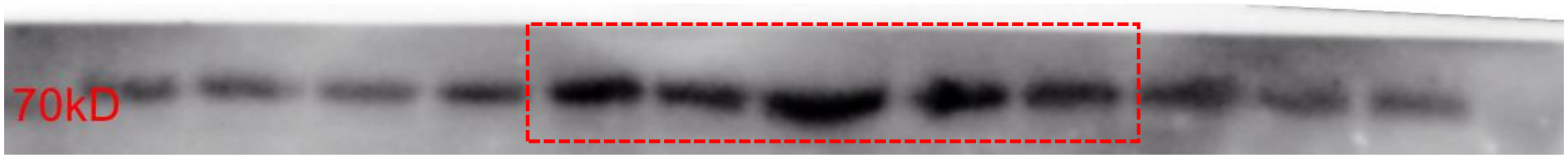

Sample-2 Gapdh-Hippo

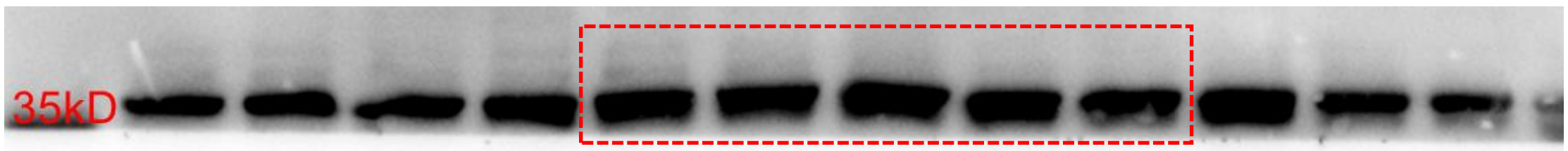

Sample-3 Cpt1a -Hippo

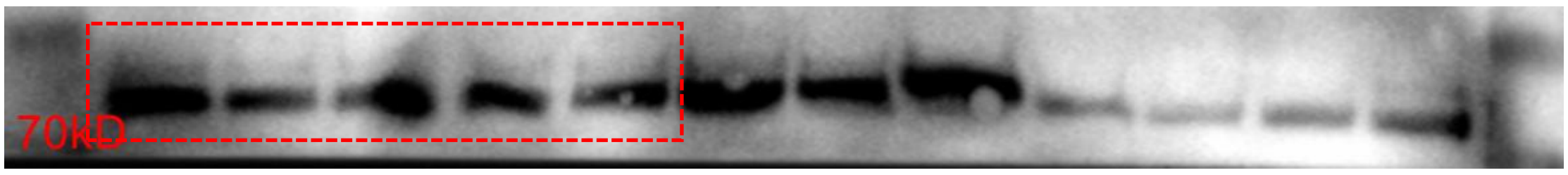

Sample-3 Gapdh-Hippo

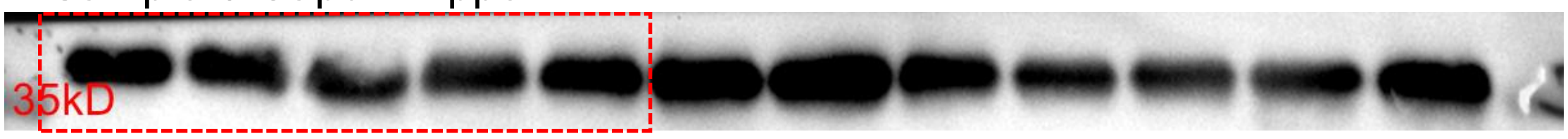

Sample-4 Cpt1a -Hippo

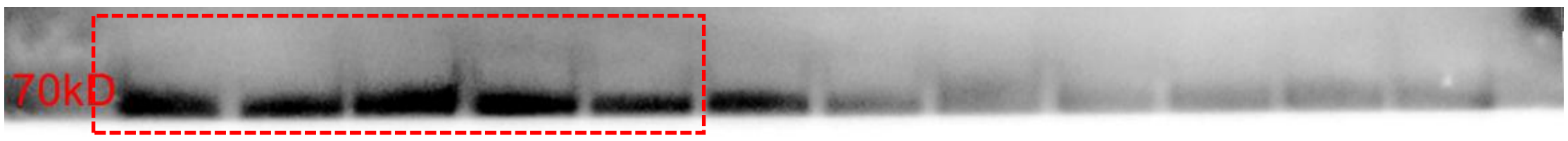

Sample-4 β-actin

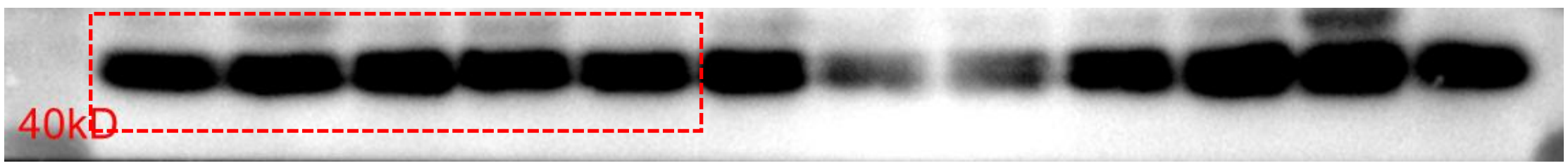

Figure 3a. Ndufb10

Sample-1 Ndufb10 cortex+Hippo

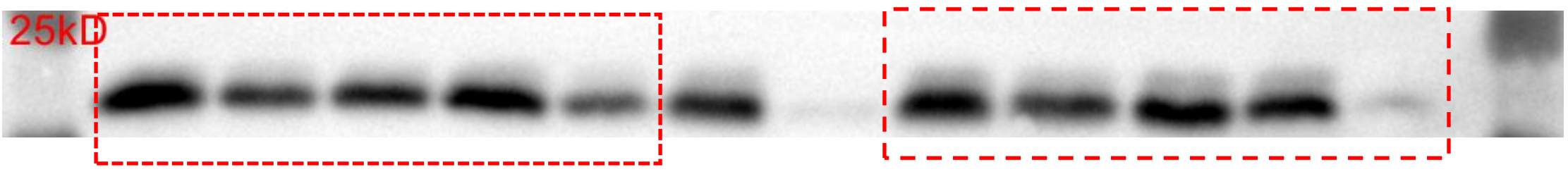

Sample-1 β-Tubulin cortex+Hippo

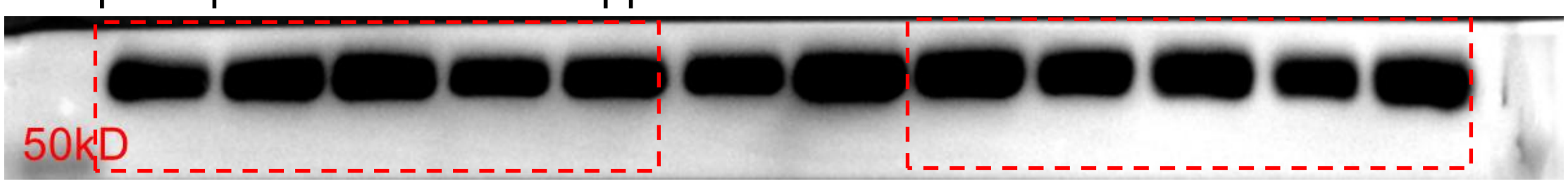

Sample-2 Ndufb10 cortex

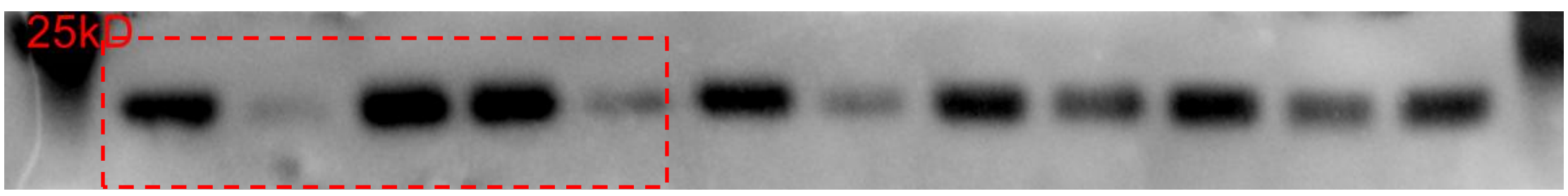

Sample-2 β-tubulin cortex

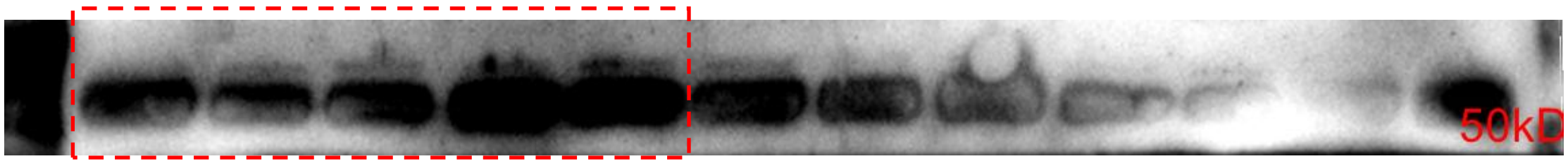

Sample-3 Ndufb10 cortex

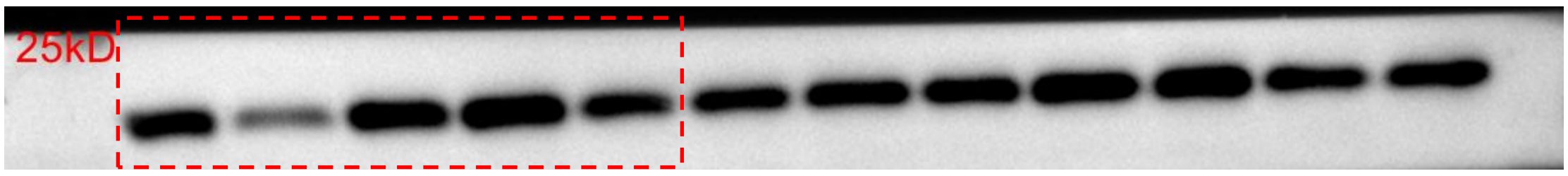

Sample-3 β-tubulin cortex

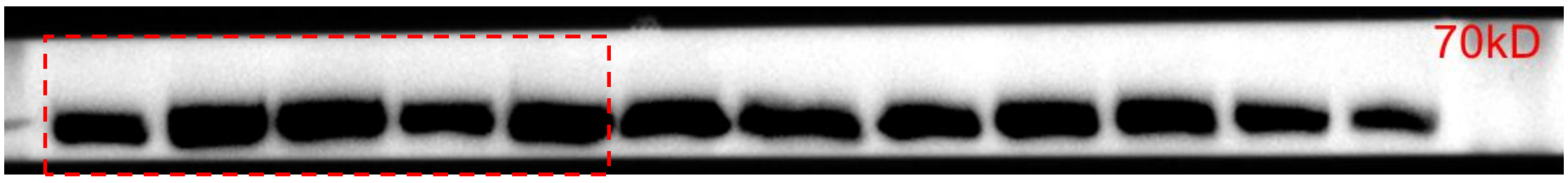

Sample-4 Ndufb10 cortex

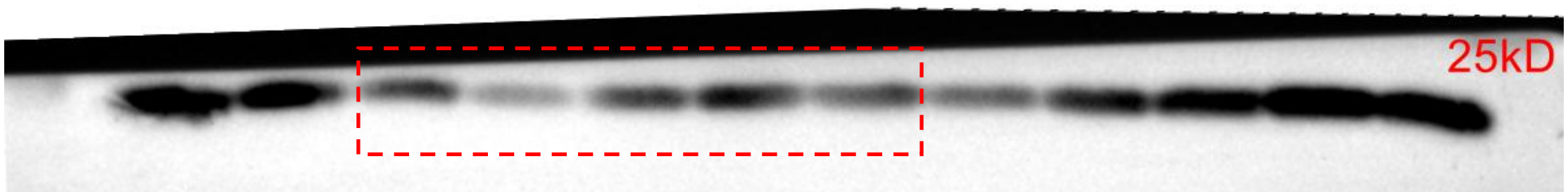

Sample-4 β-tubulin cortex

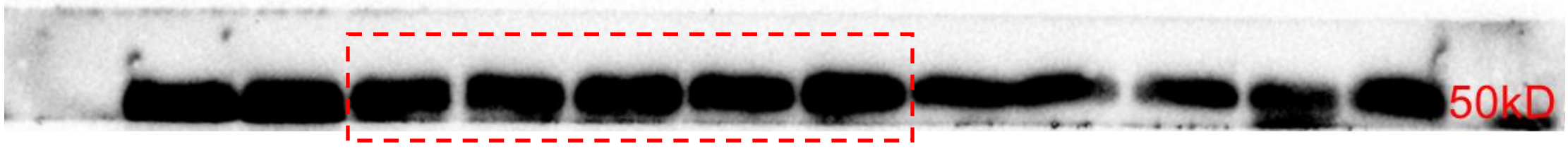

Hippocampus

Sample-2 Ndufb10 hippo

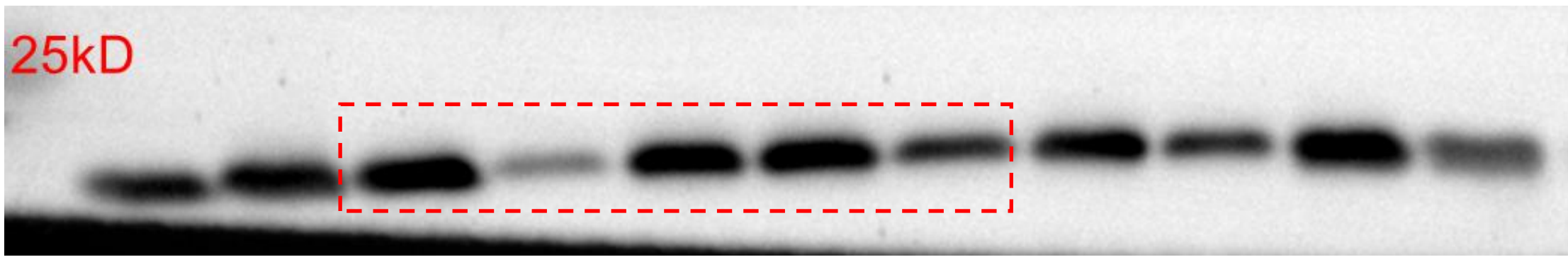

Sample-2 β-Tubulin hippo

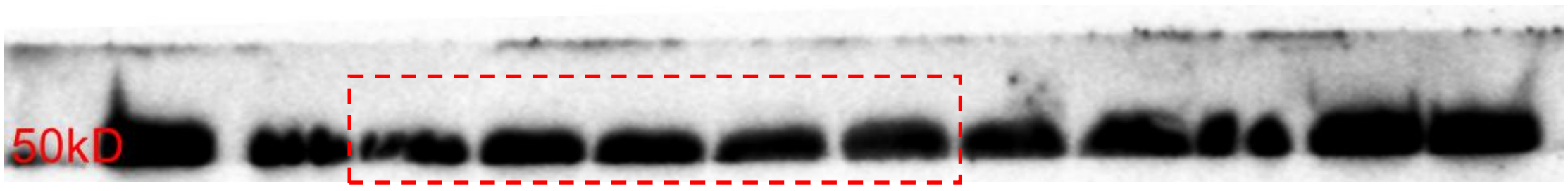

Sample-3 Ndufb10 hippo

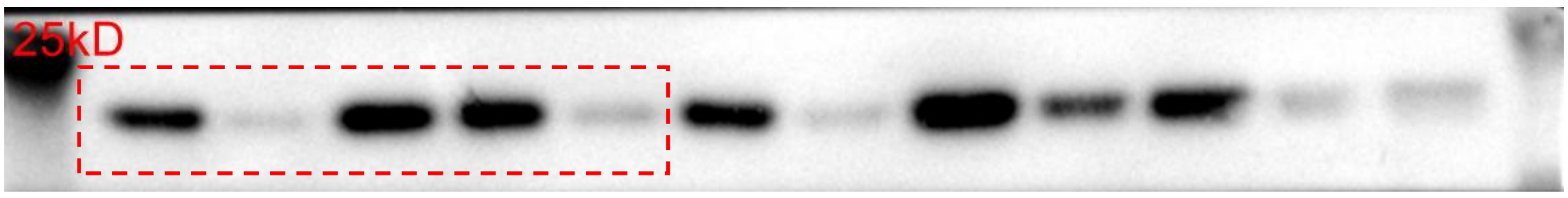

Sample-3 β-Tubulin hippo

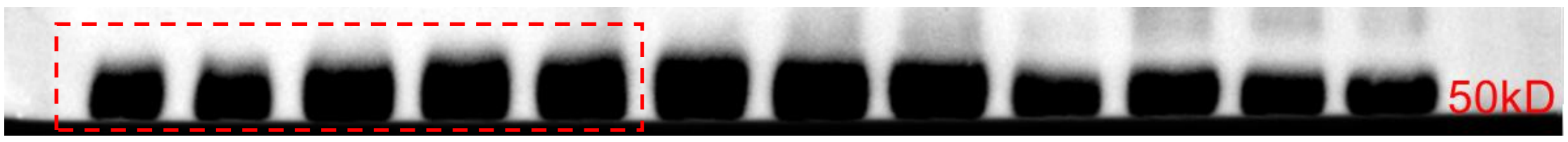

Sample-4 Ndufb10 hippo

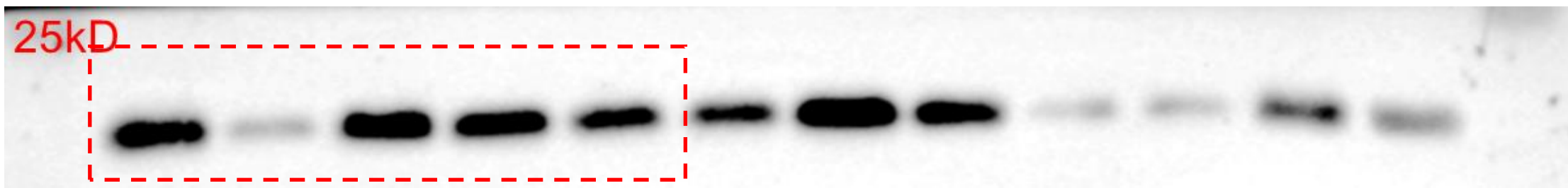

Sample-4 β-Tubulin hippo

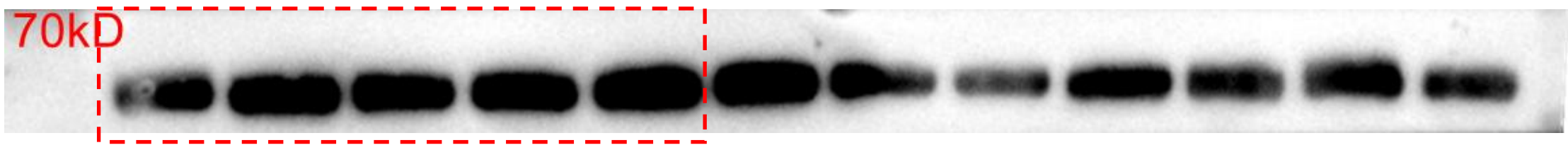

Figure 3a.Sdhb

Sample 1 Sdhb-Cortex

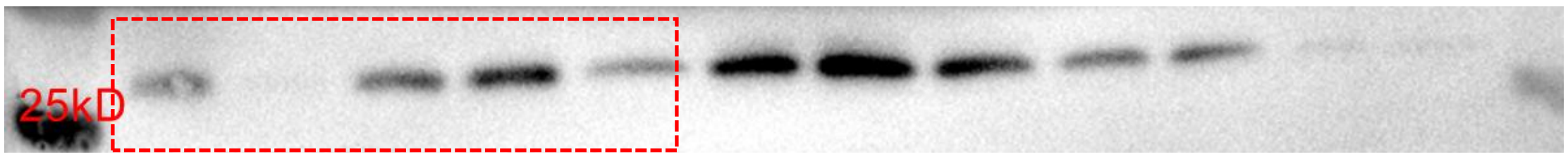

Sample 1 β-Tubulin-cortex

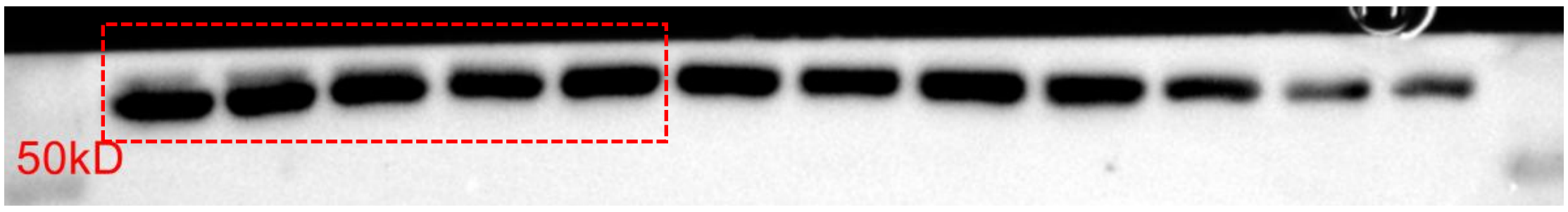

Sample 2 Sdhb-Cortex

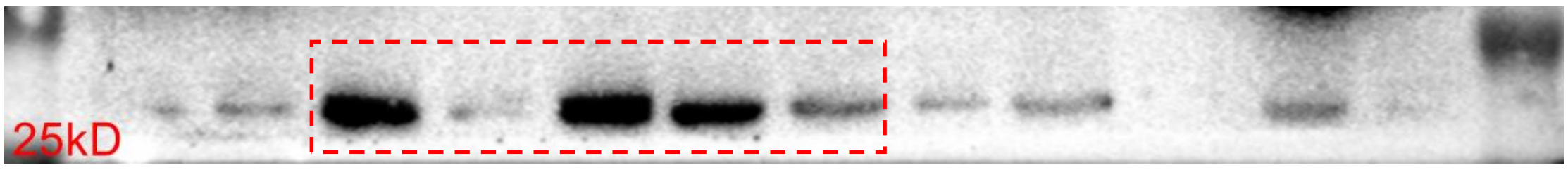

Sample 2 β-Tubulin-Cortex

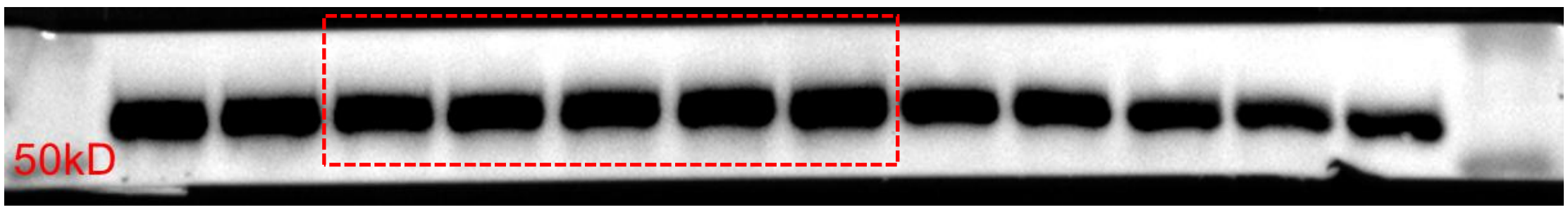

Sample 3 Sdhb-Cortex

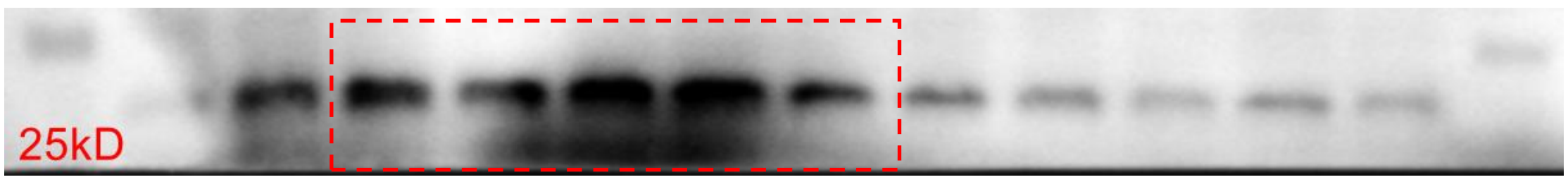

Sample 3 β-Tubulin-Cortex

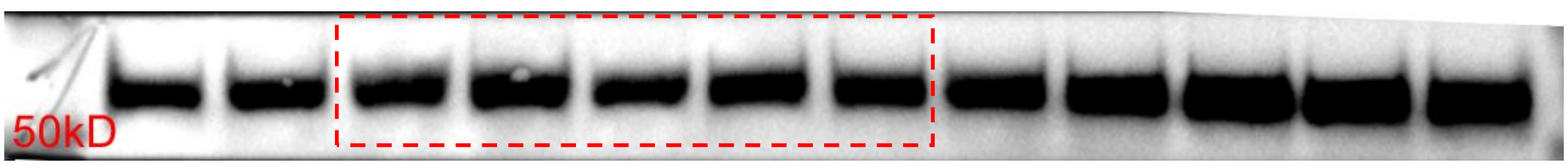

Sample 4 Sdhb-Cortex

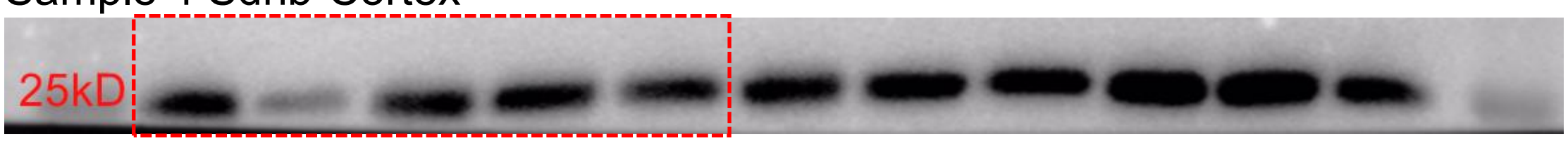

Sample 4 β-Tubulin-cortex

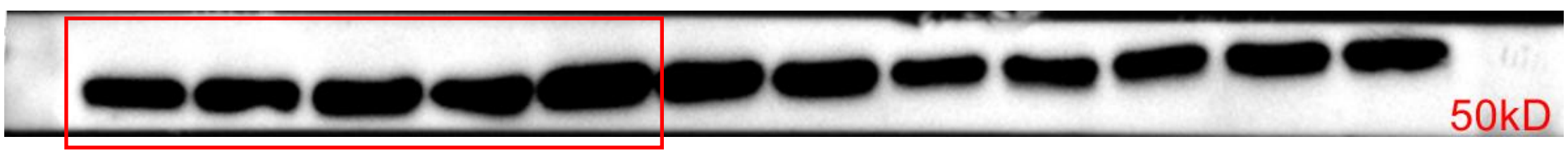

Sample 1 Sdhb-Hippo

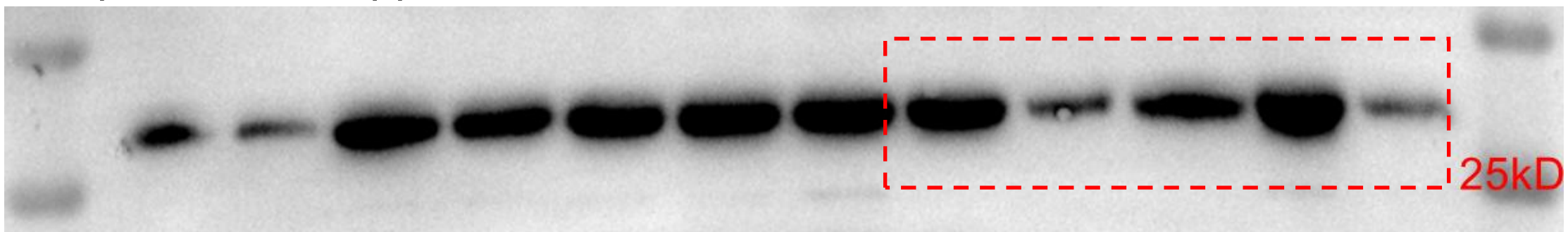

Sample 1 β-Tubulin Hippo

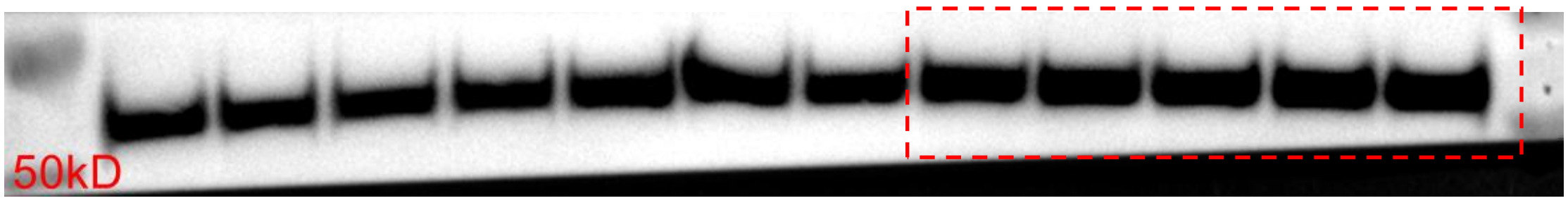

Sample 2 Sdhb-Hippo

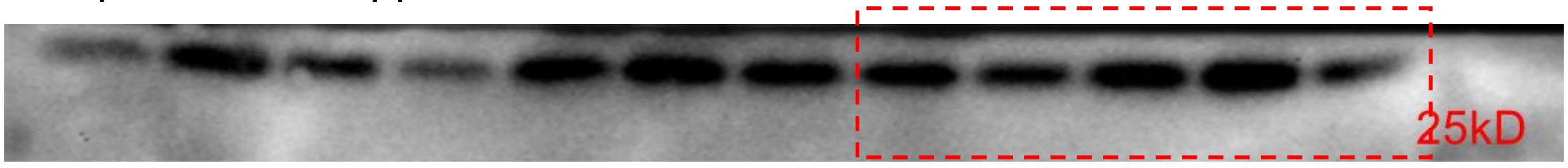

Sample 2 β-Tubulin-Hippo

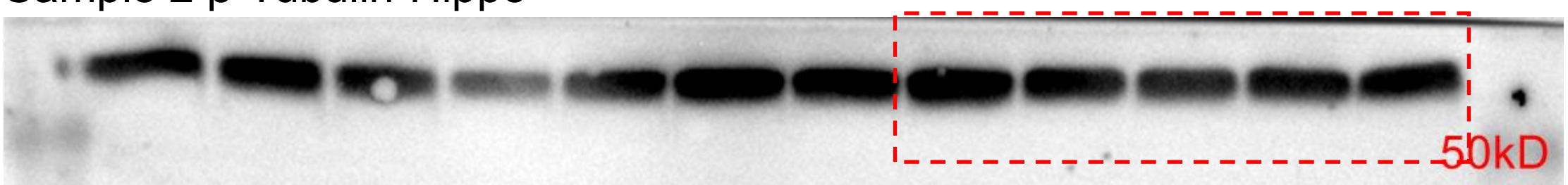

Sample 3 Sdhb-Hippo

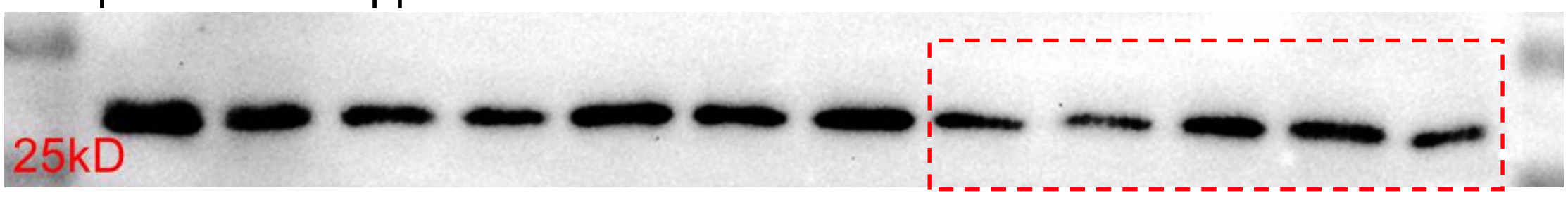

Sample 3 β-Tubulin-Hippo

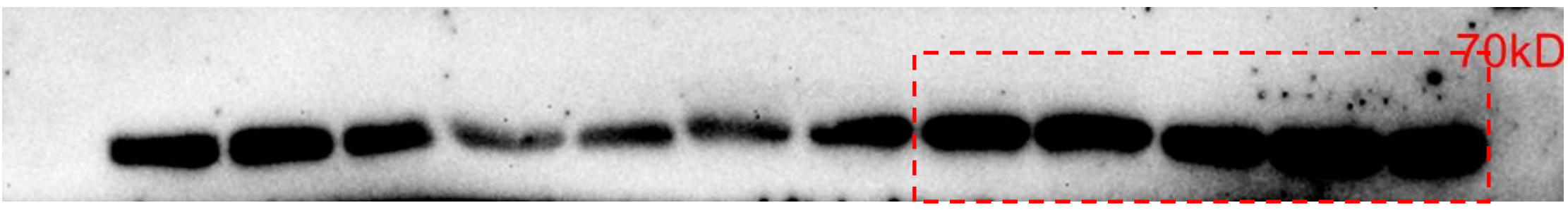

Sample 4 Sdhb-Hippo

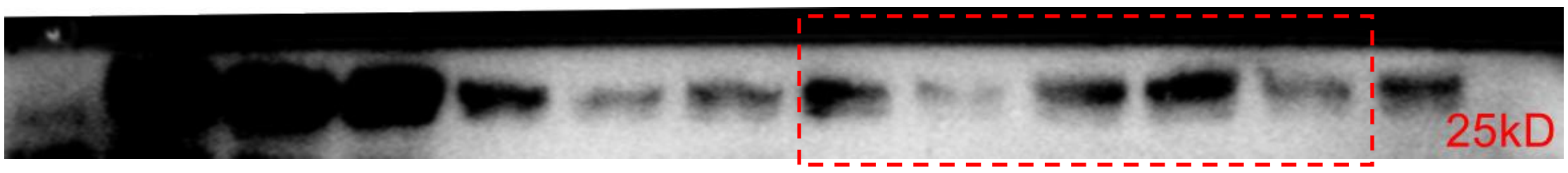

Sample 4 β-Tubulinn-Hippo

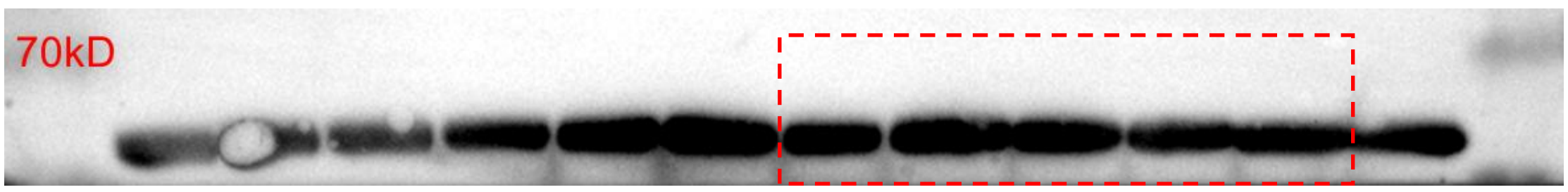

Figure 3a.Uqcrc2

Sample 1 Uqcrc2 cortex

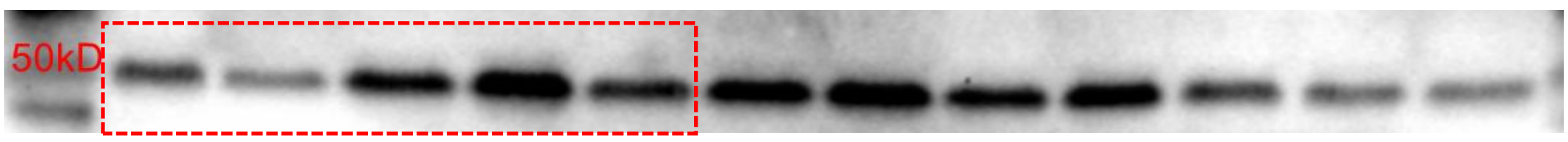

Sample 1 Gapdh cortex

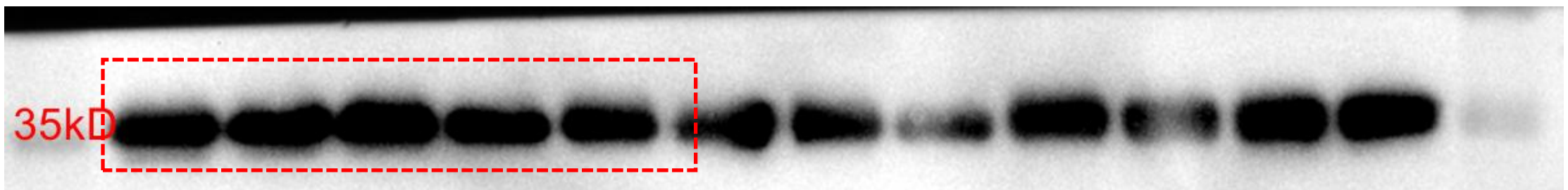

Sample 2 Uqcrc2 cortex

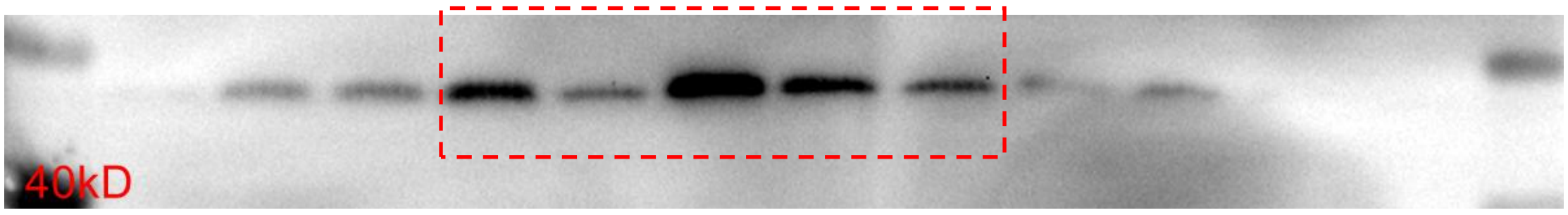

Sample 2 Gapdh cortex

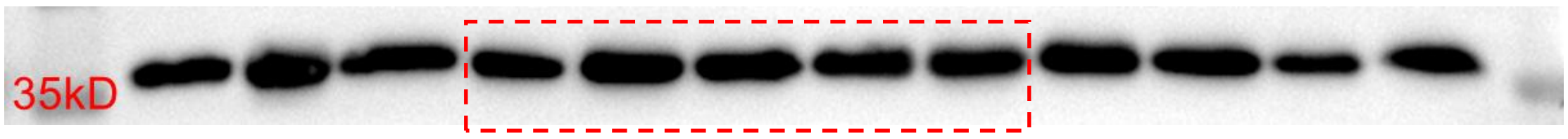

Sample 3 Uqcrc2 cortex

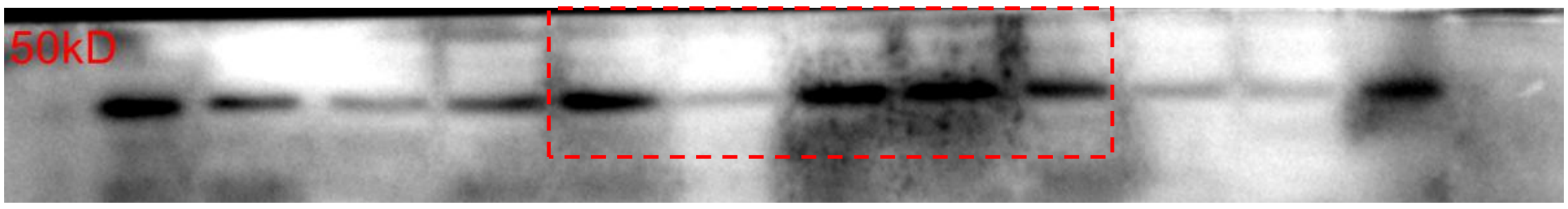

Sample 3 Gapdh cortex

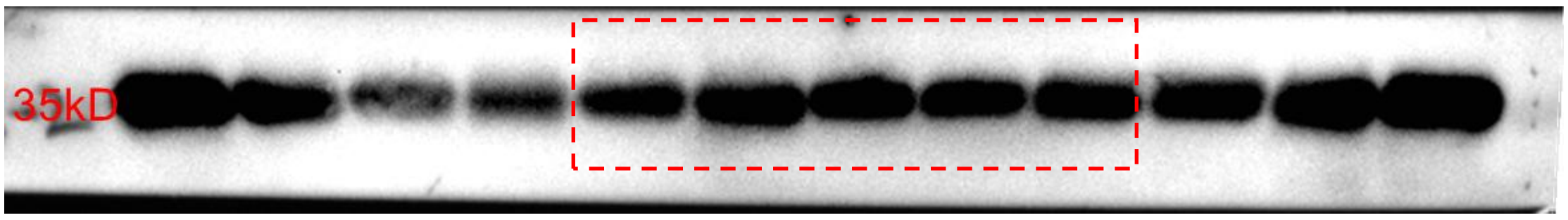

Sample 4 Uqcrc2 cortex

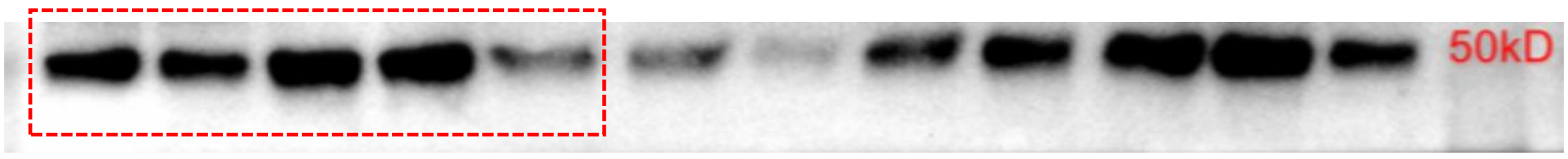

Sample 4 Gapdh cortex

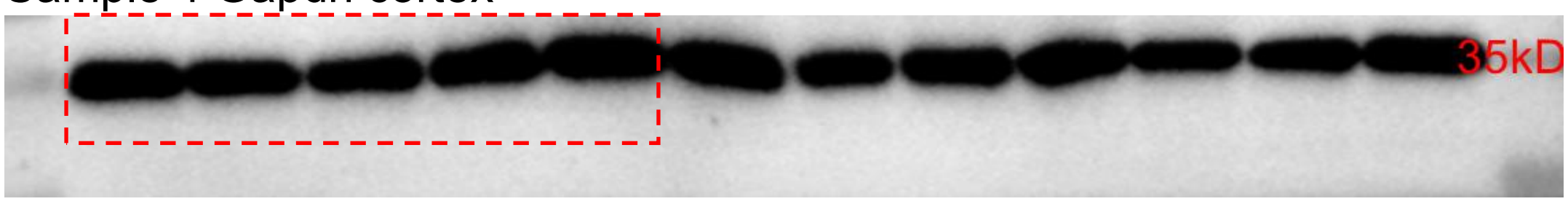

Sample 1 Uqcrc2 Hippo

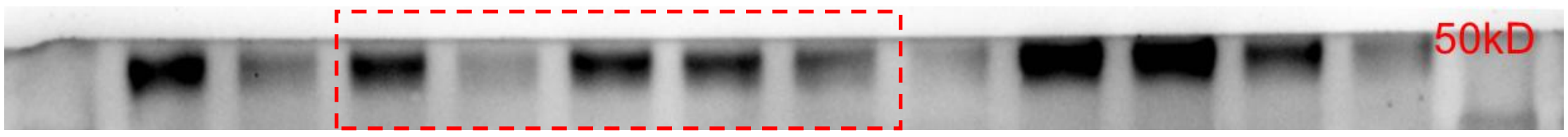

Sample 1 Gapdh Hippo

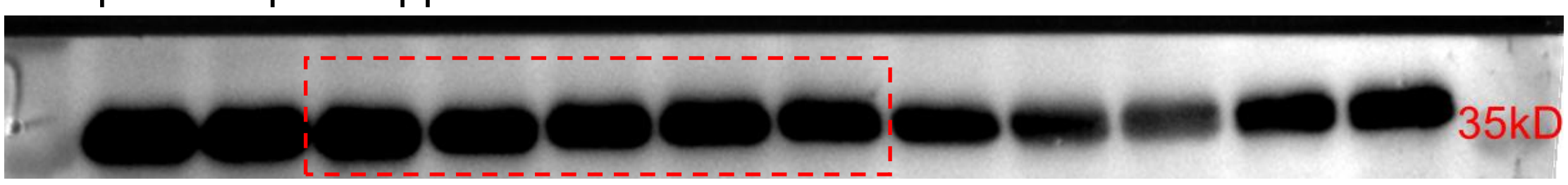

Sample 2 Uqcrc2 hippo

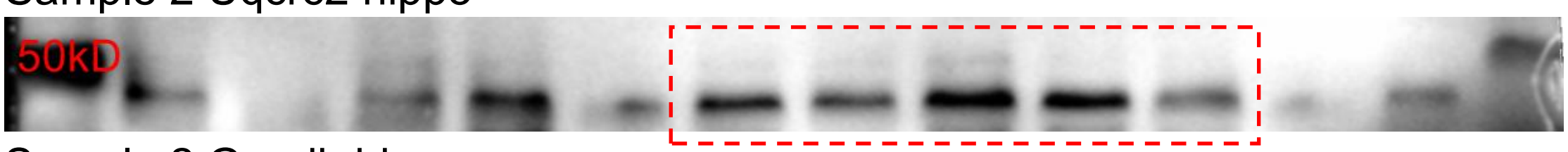

Sample 2 Gapdh hippo

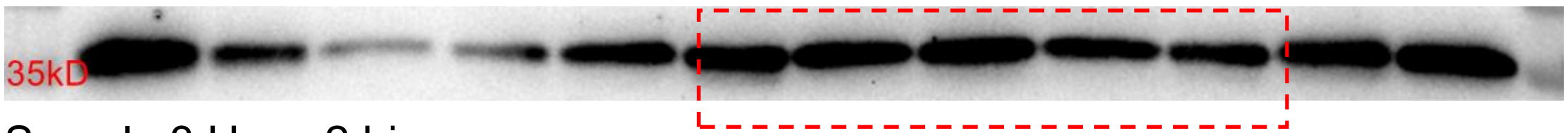

Sample 3 Uqcrc2 hippo

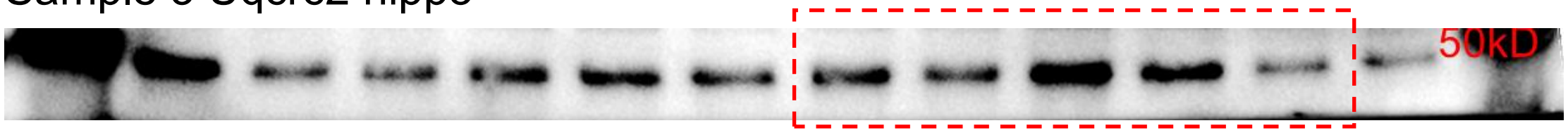

Sample 3 Gapdh hippo

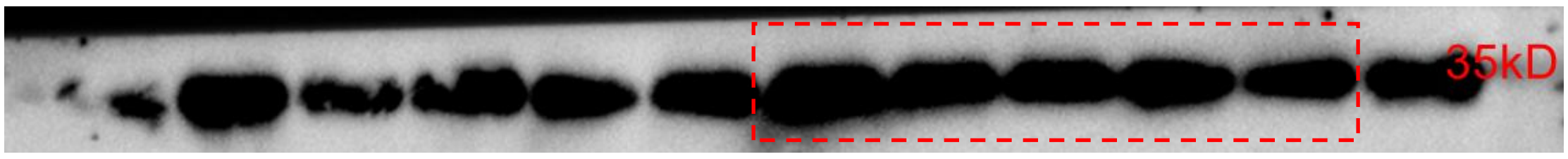

Sample 4 Uqcrc2 hippo

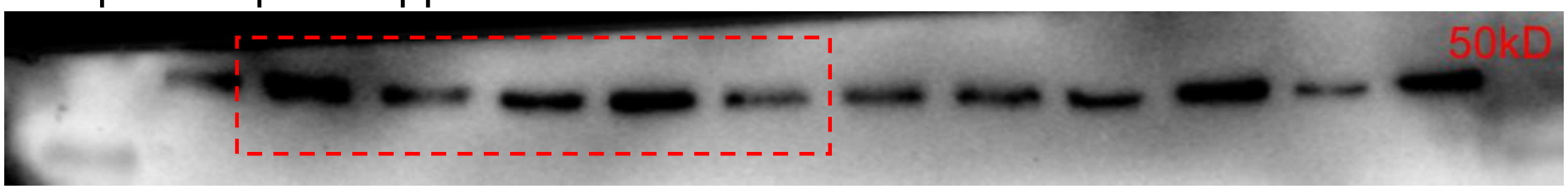

Sample 4 Gapdh hippo

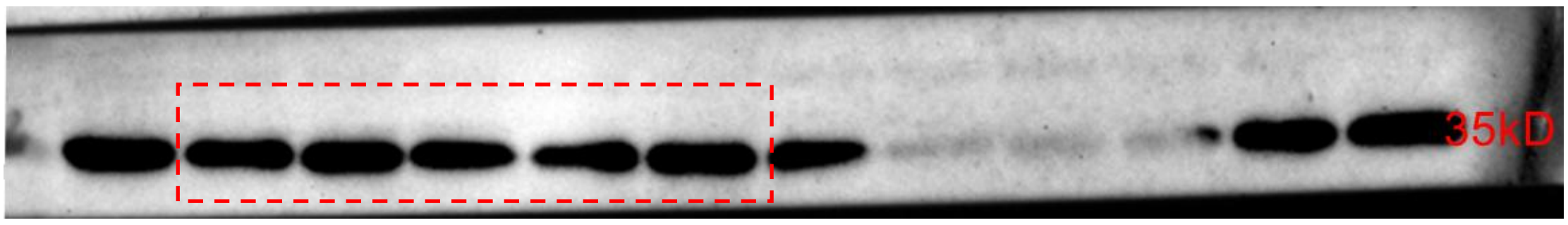

Figure 3a. Mtco2

Sample 1 MtCO2 cortex+Hippo

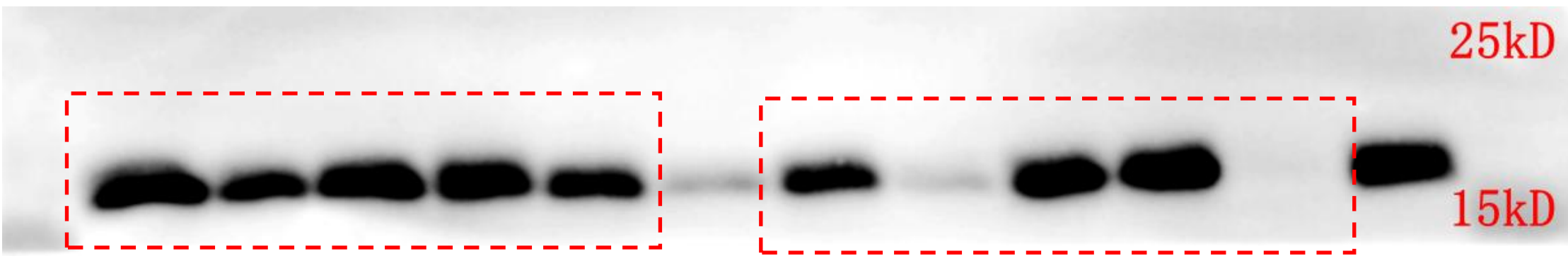

Sample 1 Gapdh Cortex+Hippo

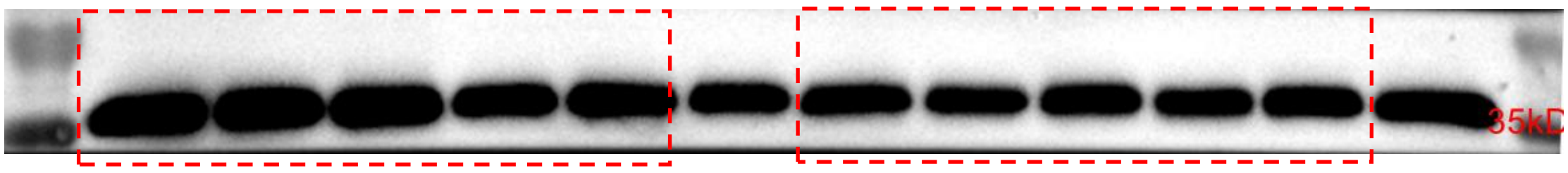

Sample 2 MtCO2 cortex+Hippo

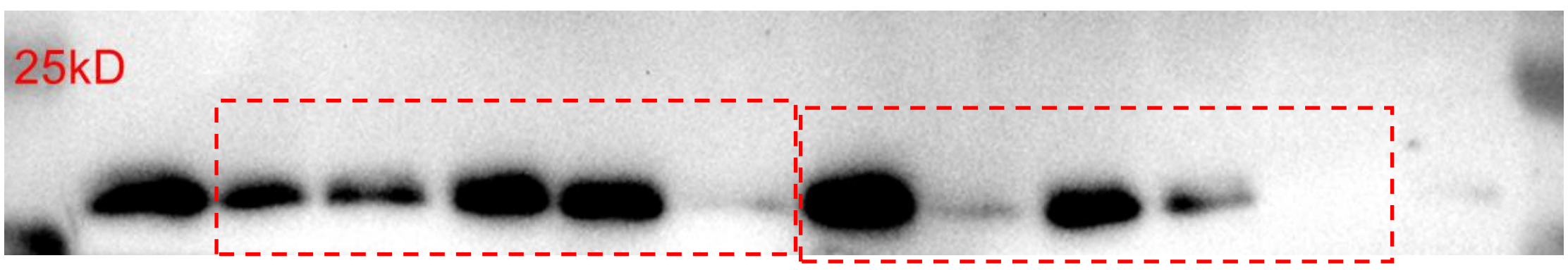

Sample 2 Gapdh cortex+Hippo

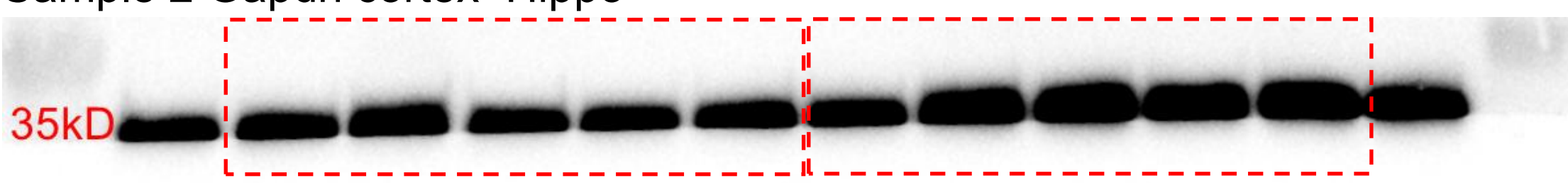

Sample 3 MtCO2 cortex

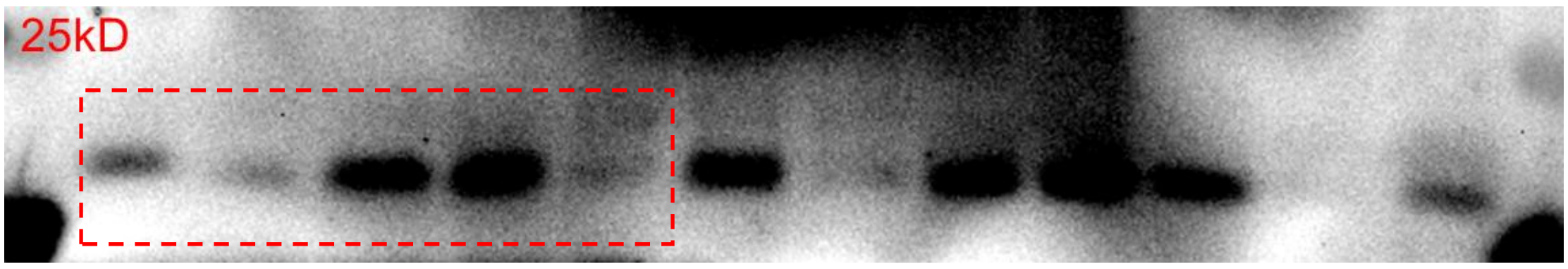

Sample 3 Gapdh cortex

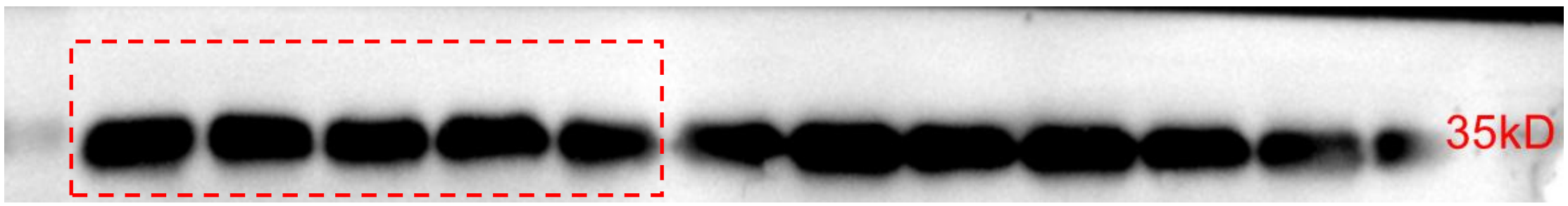

Sample 4 MtCO2 cortex

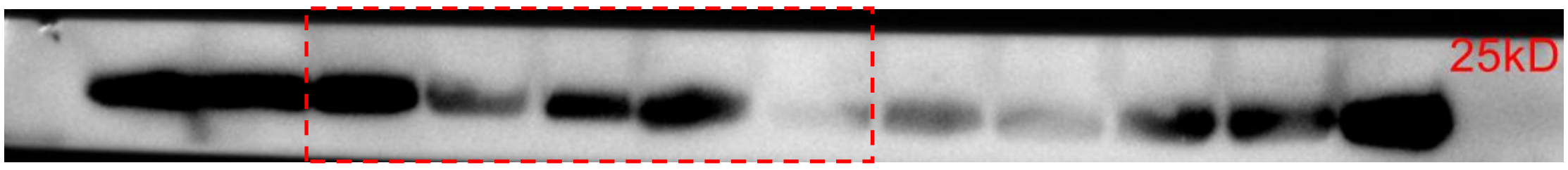

Sample 4 Gapdh cortex

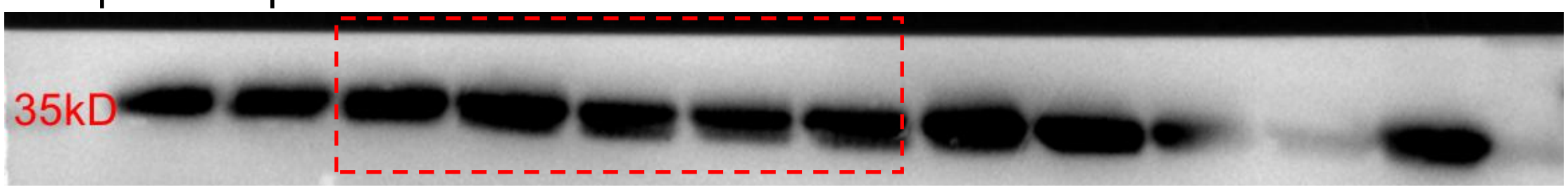

Sample 3 MtCO2 Hippo

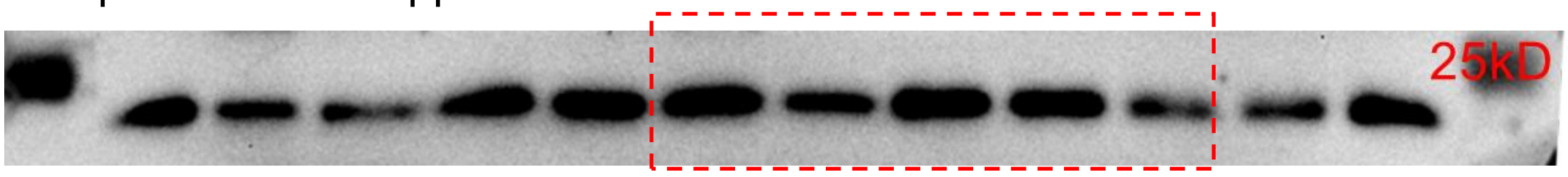

Sample 3 Gapdh Hippo

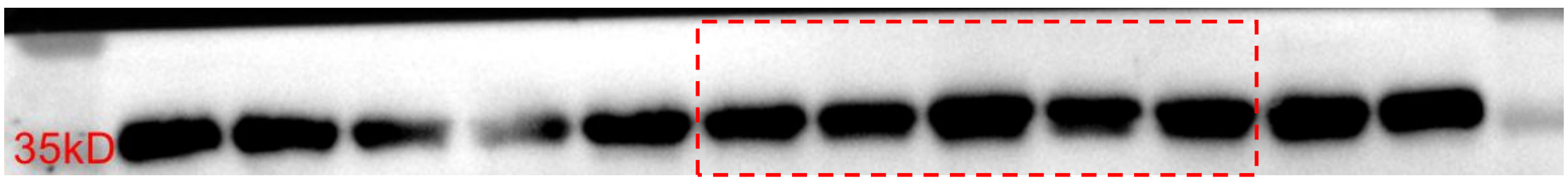

Sample 4 MtCO2 Hippo

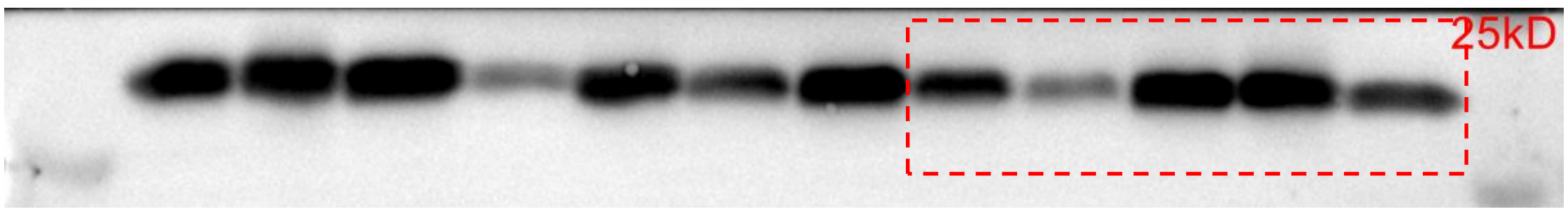

Sample 4 Gapdh hippo

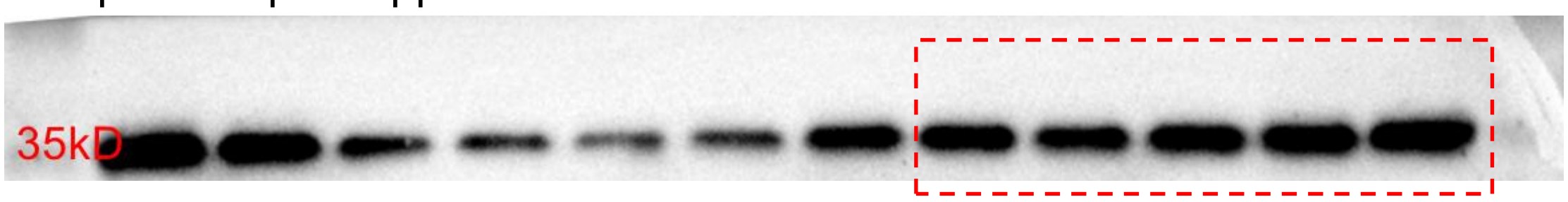

Figure 3a.Atpaf1

Sample -1 Atpaf1-cortex

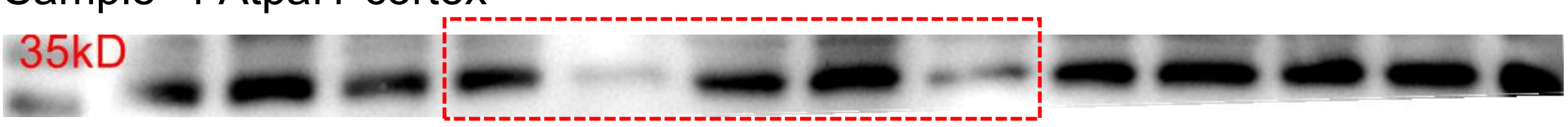

Sample -1 β Actin-cortex

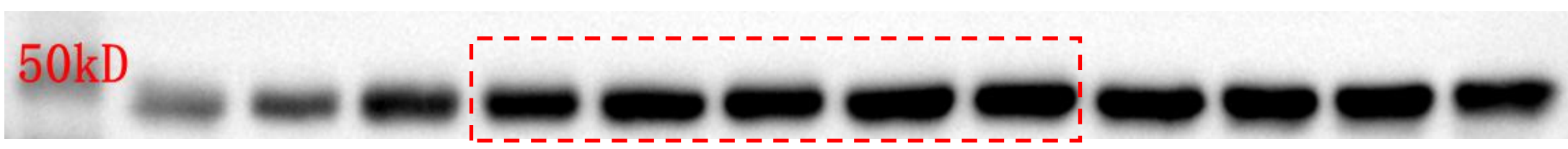

Sample -2 Atpaf1-cortex

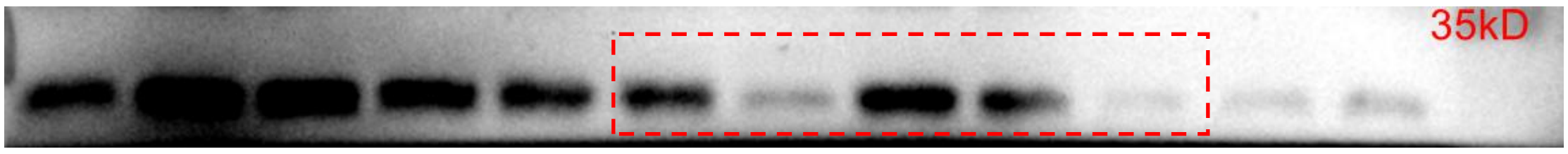

Sample -2 β Actin-cortex

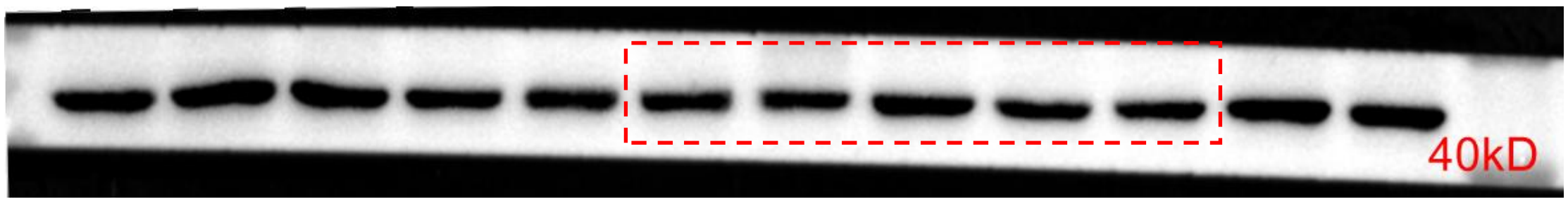

Sample -3 Atpaf1-cortex

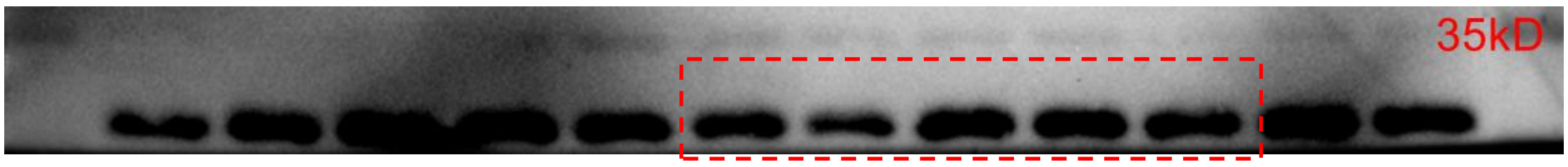

Sample -3 β Actin-cortex

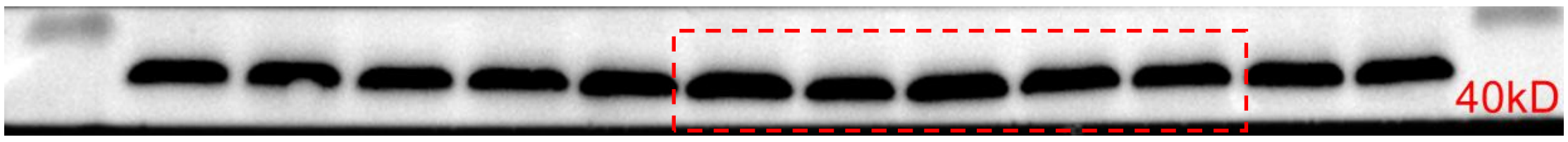

Sample -4 Atpaf1-cortex

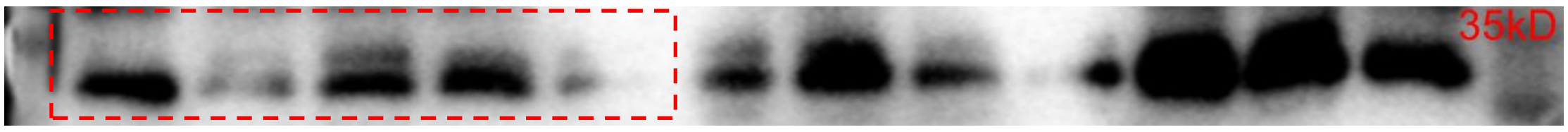

Sample -4 β Actin-cortex

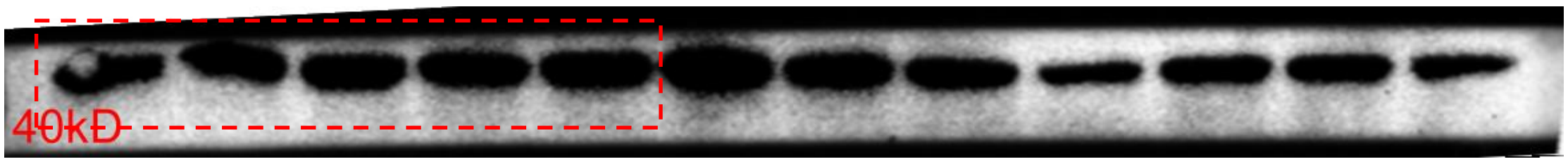

Sample -1 Atpaf1-Hippo

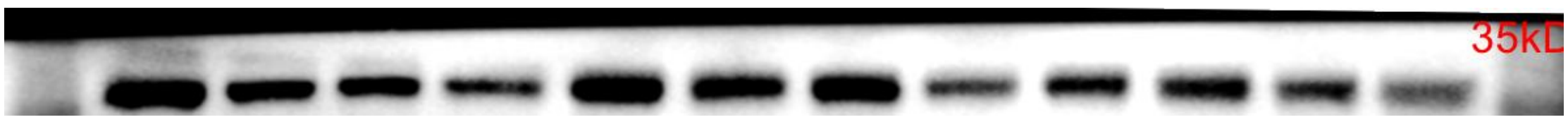

Sample -1 β Actin-Hippo

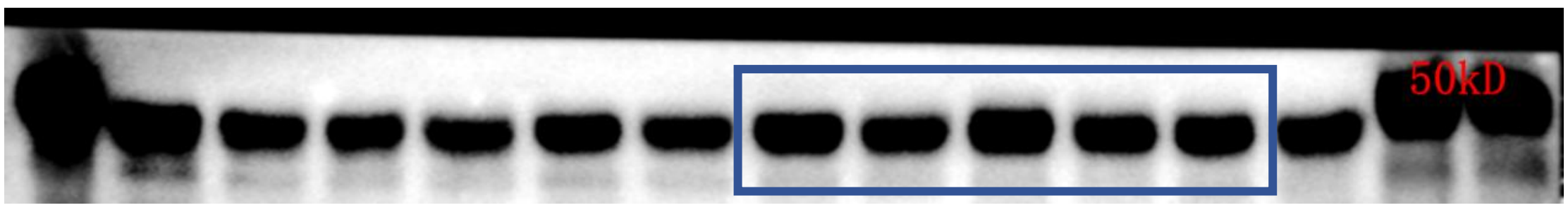

Sample2 Atpaf1-Hippo

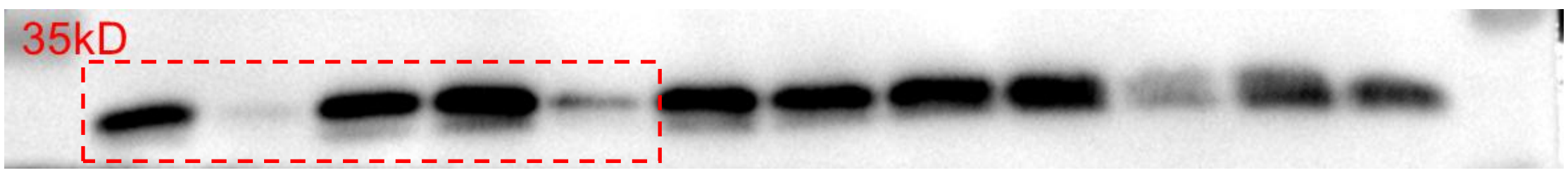

Sample2 β Actin-Hippo

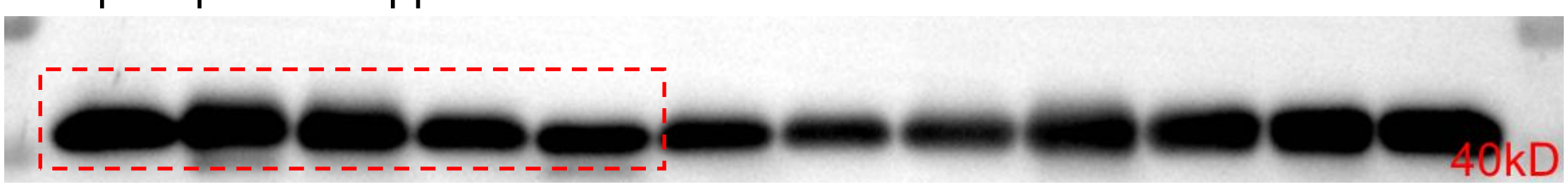

Sample3 Atpaf1-Hippo

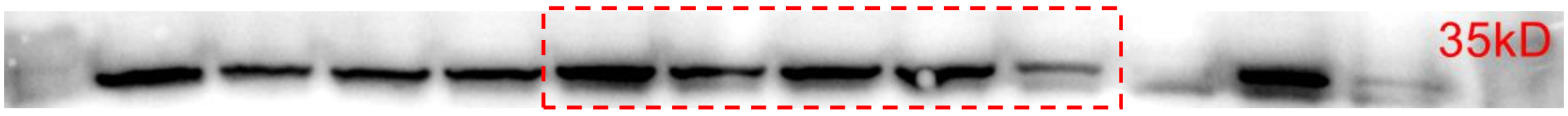

Sample3 β Actin-Hippo

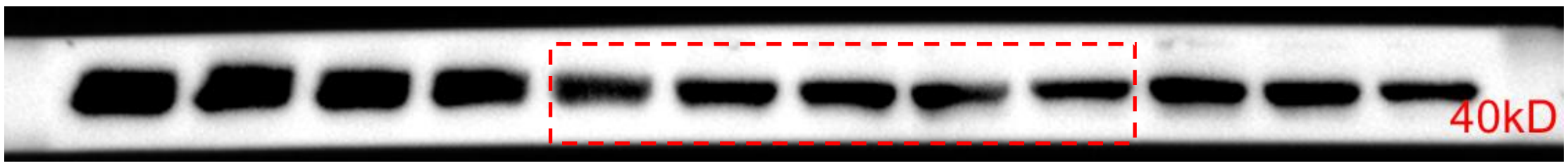

Sample4 Atpaf1-Hippo

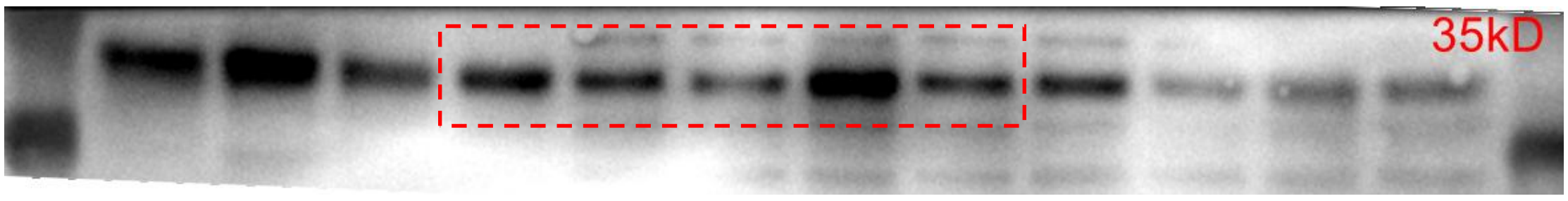

Sample 4 β Actin-Hippo

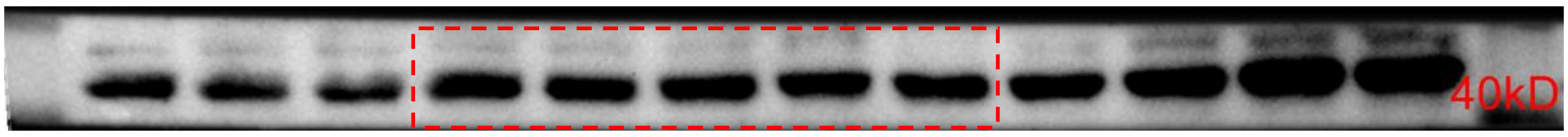

Figure 3c. Nlrp3,Caspase-1/Cleaved caspase-1,Il-18 and Il-10

Figure 3c. Nlrp3

Sample-1 Nlrp3-Cortex

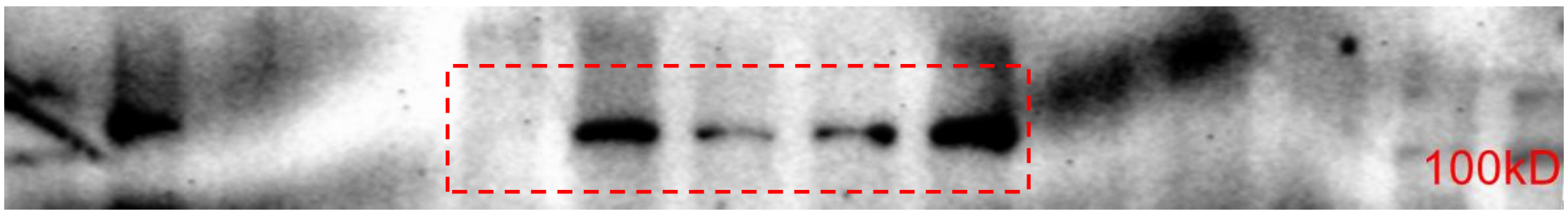

Sample-1 GAPDH-Cortex

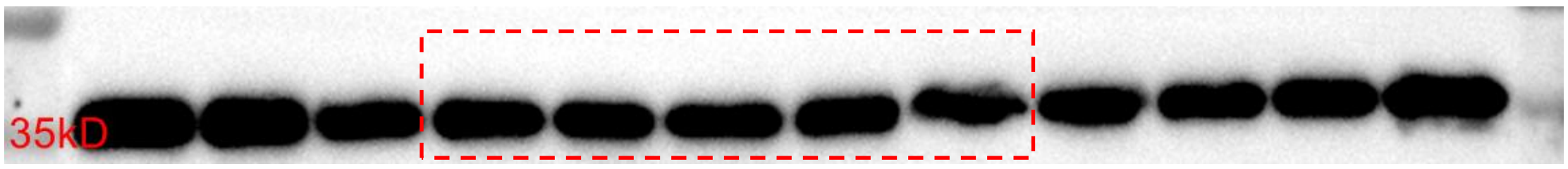

Sample-2 Nlrp3-Cortex

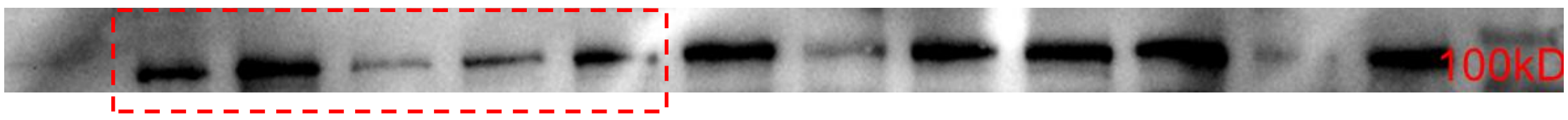

Sample-2 Gapdh-Cortex

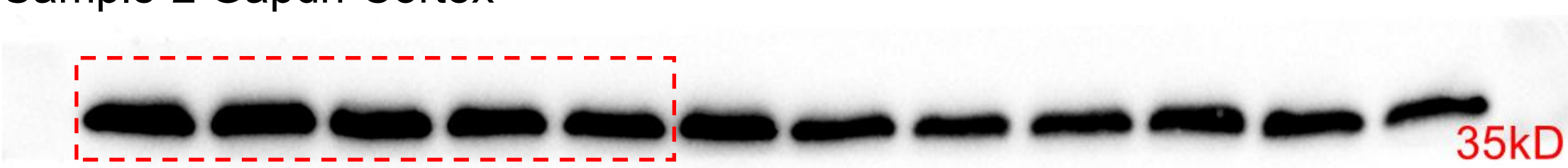

Sample-3 Nlrp3-Cortex

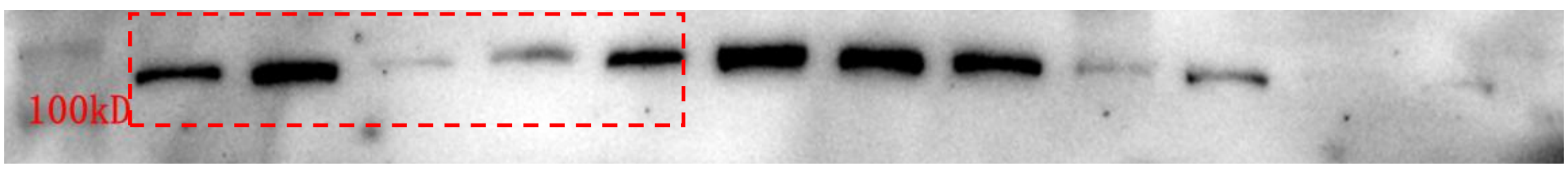

Sample-3 Gapdh-Cortex

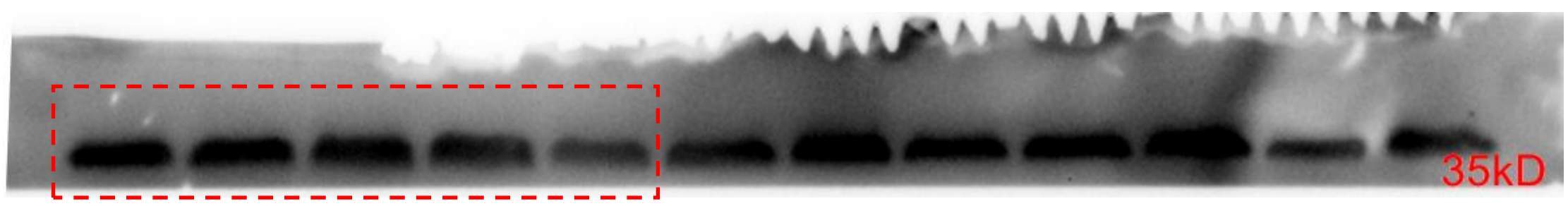

Sample-4 Nlrp3-Cortex

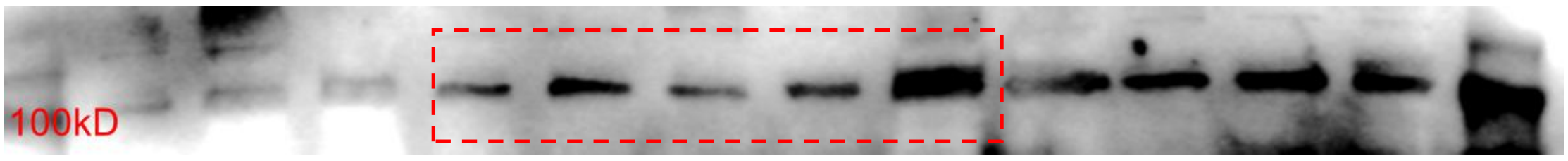

Sample-4 Gapdh-Cortex

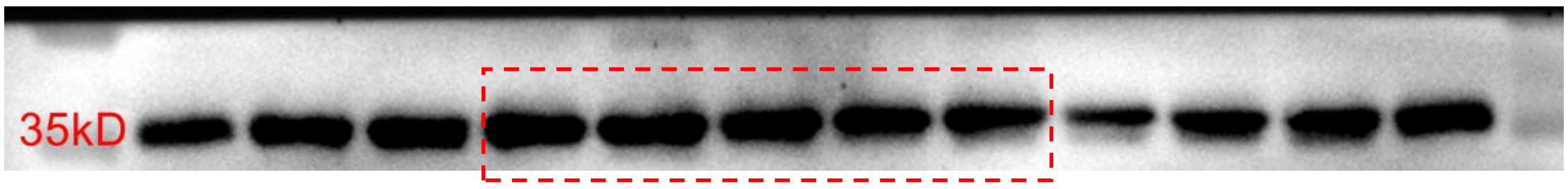

Sample 1-Nlrp3 hippo

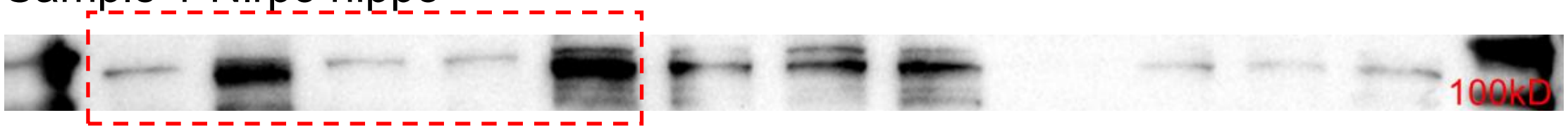

Sample 1-Gapdh hippo

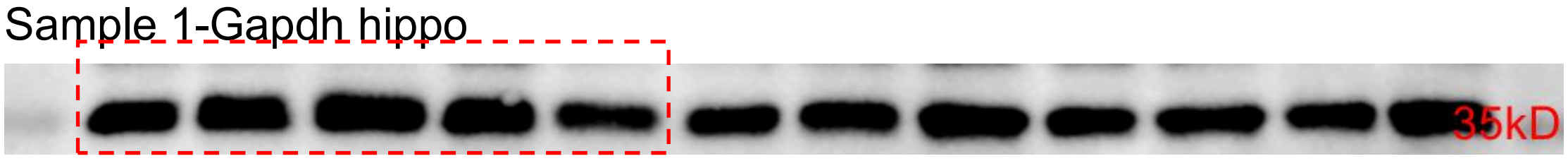

Sample 2-Nlrp3 hippo

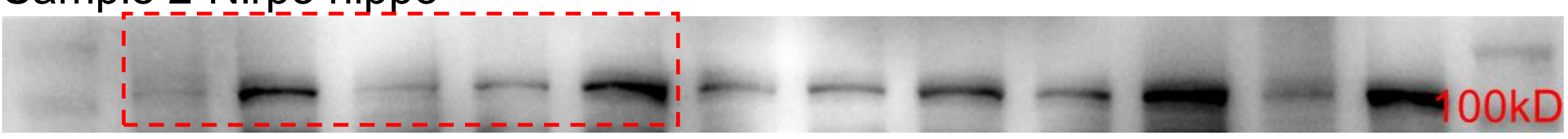

Sample 2-Gapdh hippo

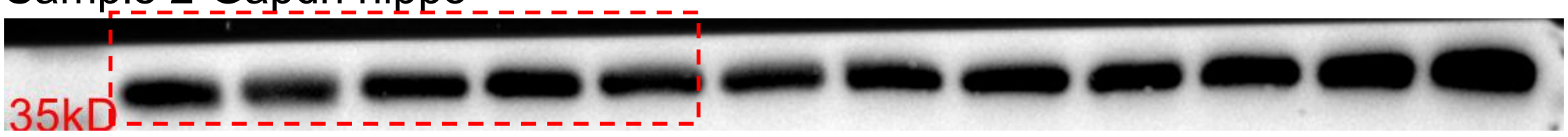

Sample 3 Nlrp3-hippo

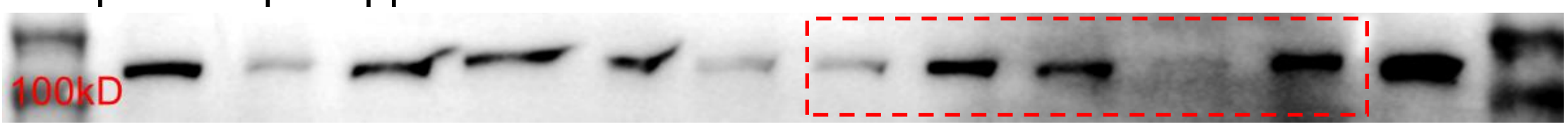

Sample 3 Gapdh-hippo

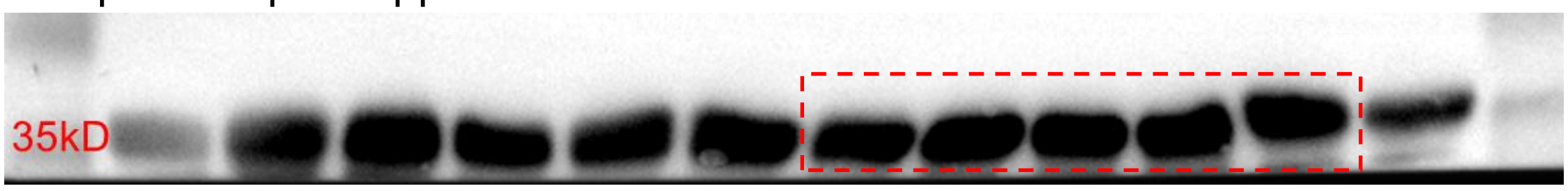

Sample 4-Nlrp3 hippo

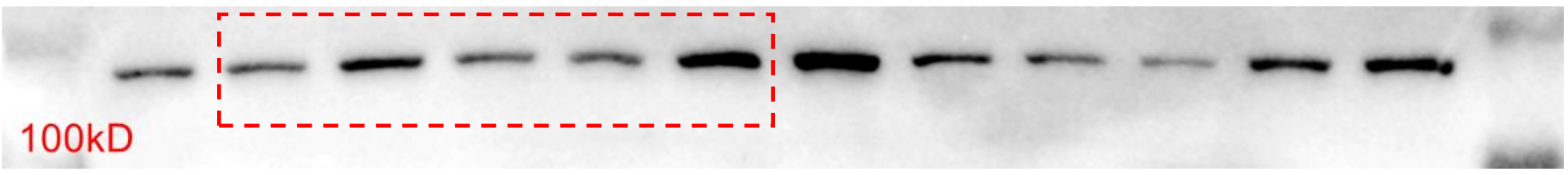

Sample 4-Gapdh hippo

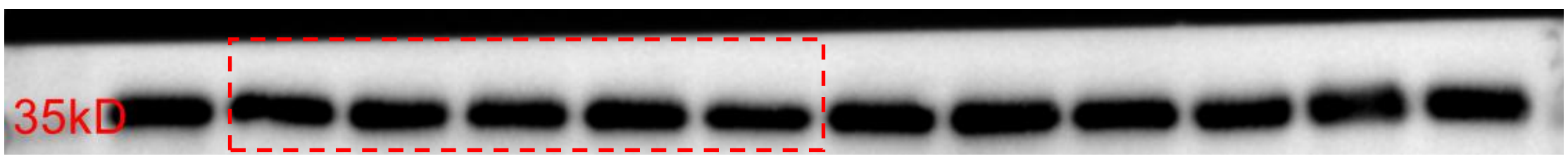

Figure 3c.caspase-1/Cleaved Caspase-1

Sample 1 Caspase-1 cortex

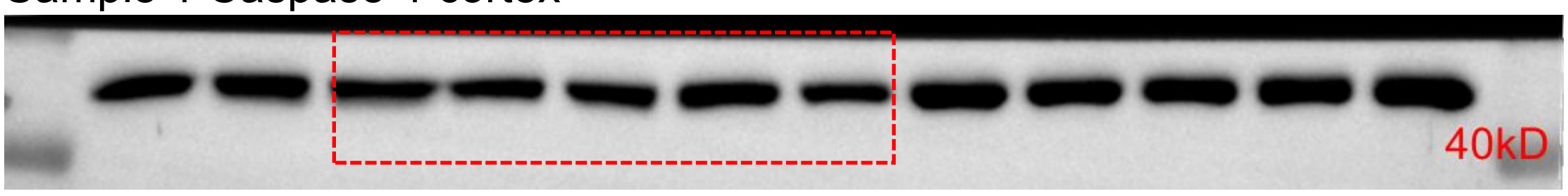

Sample 1 Cleaved Caspase-1 cortex

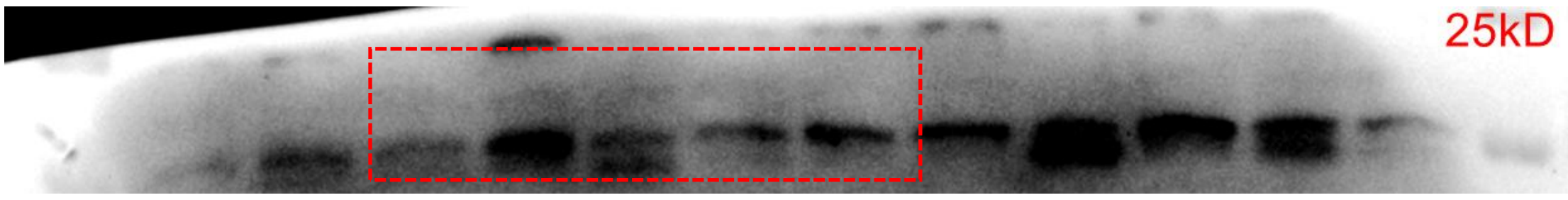

Sample 2 Caspase-1 cortex

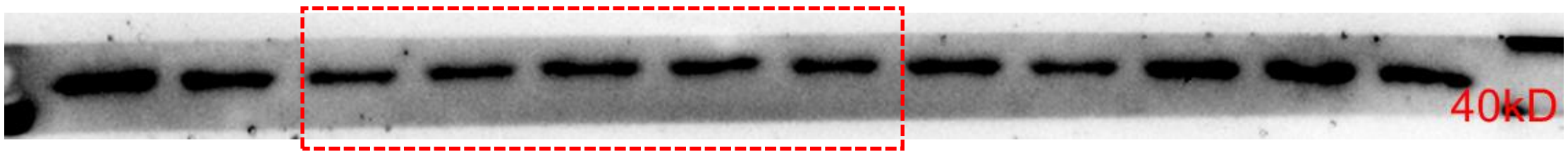

Sample 2 cortex Cleaved Caspase-1

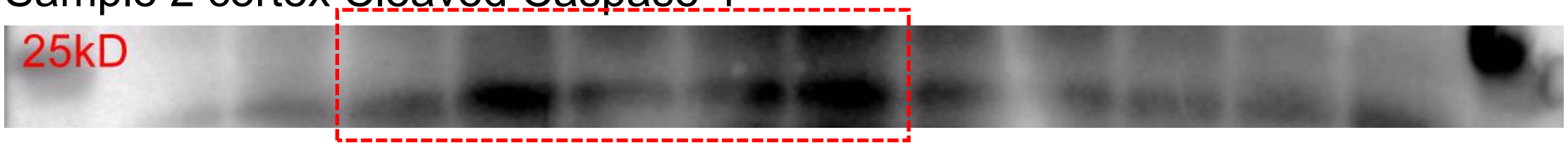

sample 3 Caspase-1 cortex

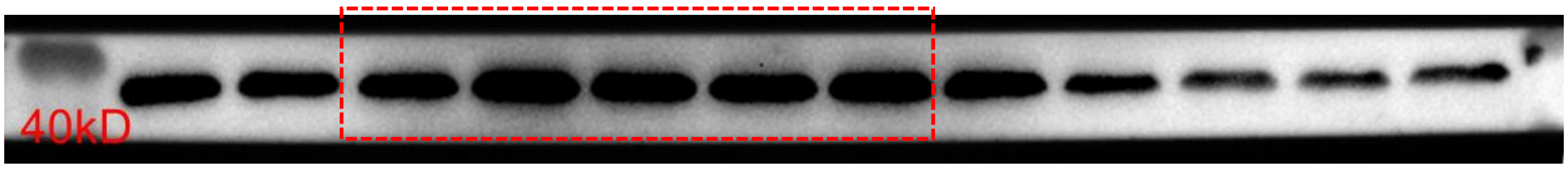

sample 3 cortex clevaed caspase-1

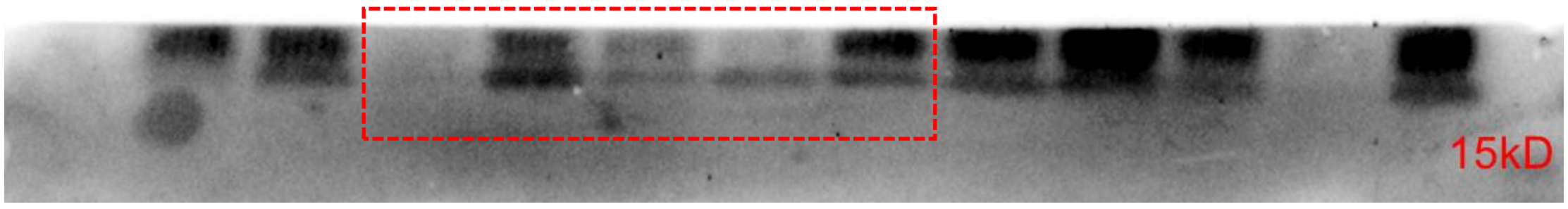

sample 4 Caspase-1 cortex

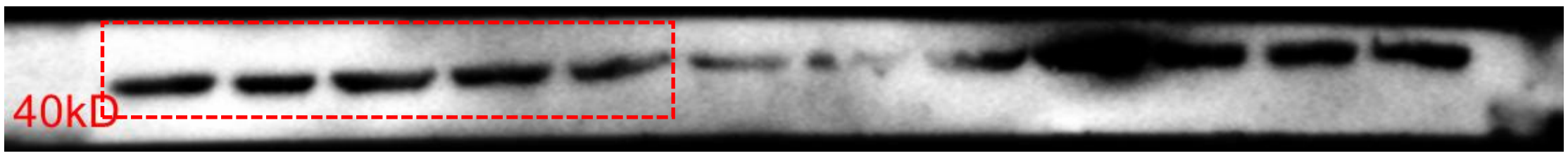

sample 4 cortex clevaed caspase-1

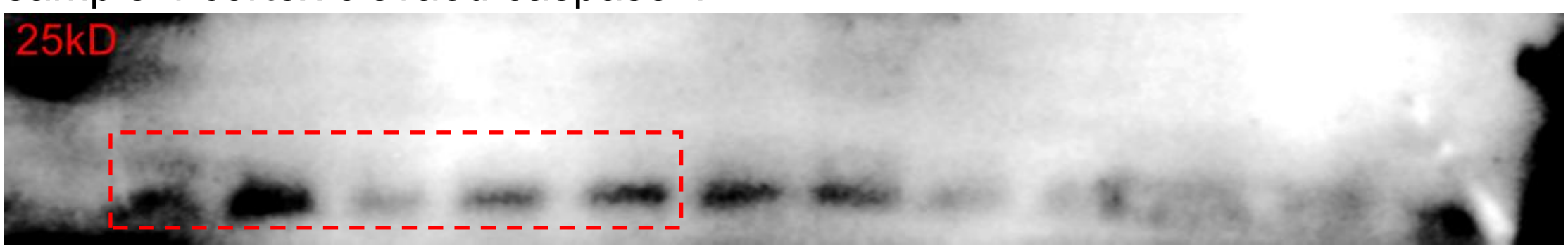

Sample 1 Caspase-1 Hippo

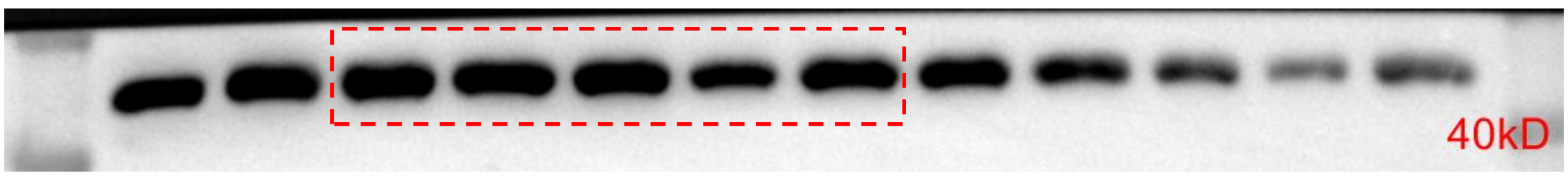

Sample 1 Cleaved Caspase-1 hippo

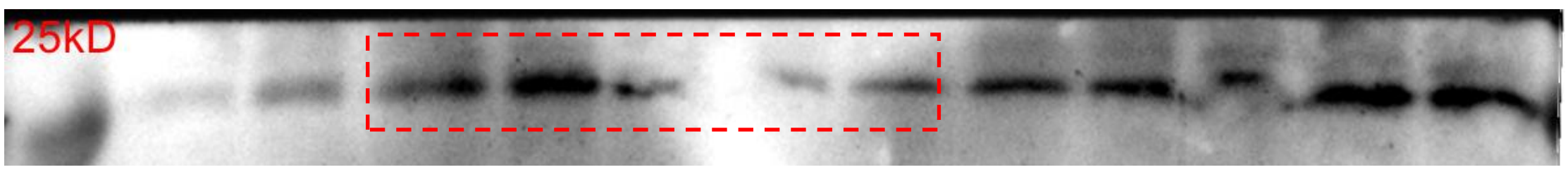

Sample 2 Caspase-1 Hippo

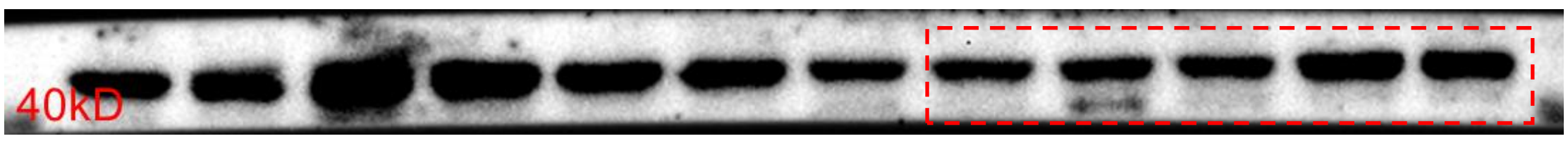

Sample 2 Cleaved Caspase-1 hippo

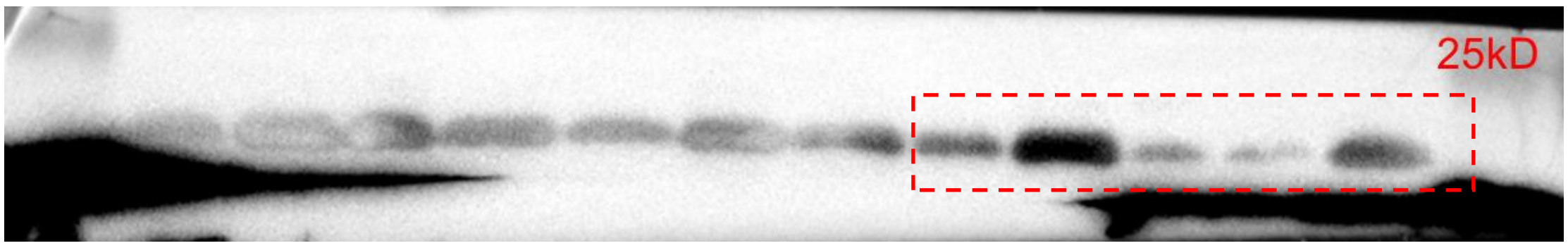

Sample 3 Caspase-1 Hippo

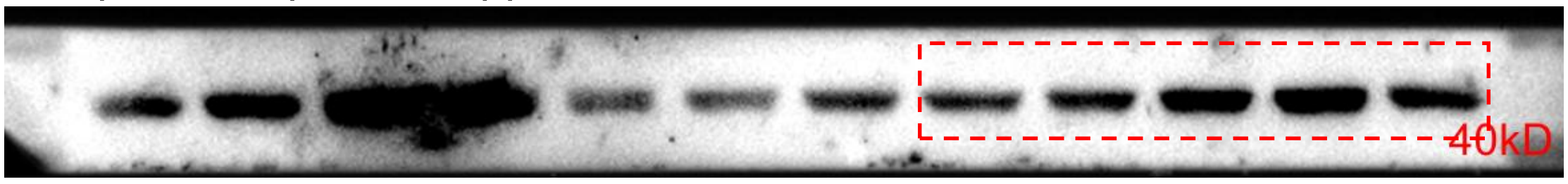

Sample 3 Cleaved Caspase-1 hippo

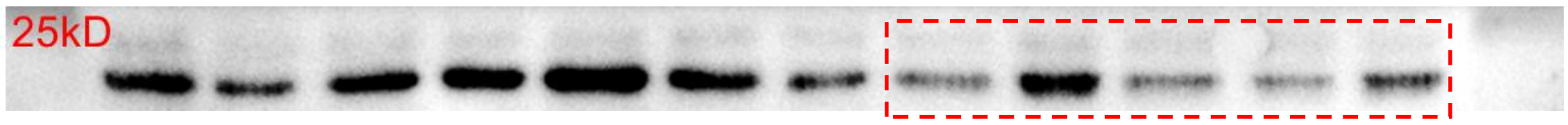

Sample 4 Caspase-1 Hippo

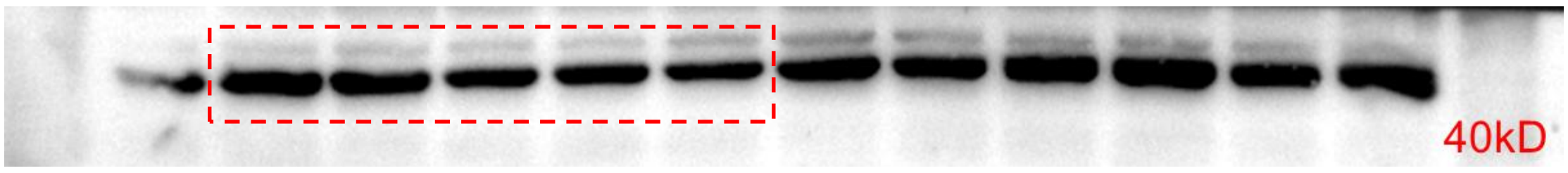

Sample 4 Cleaved Caspase-1 hippo

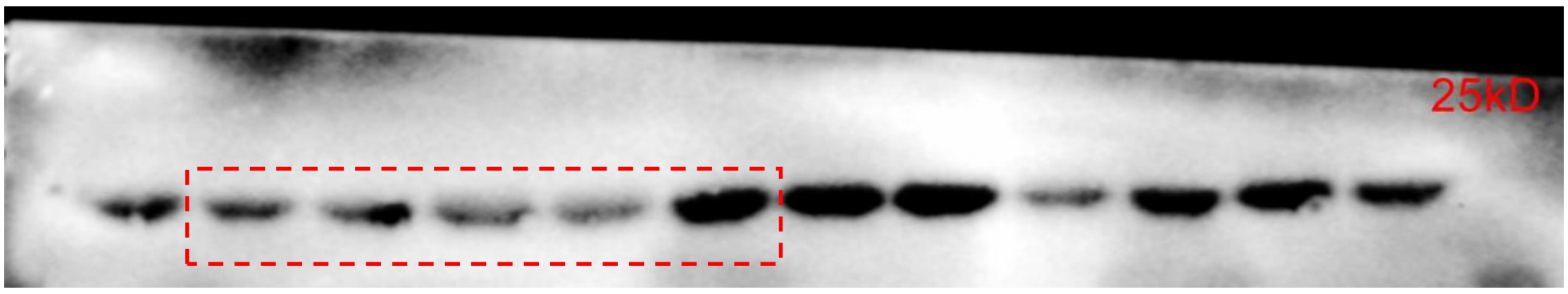

Figure 3c.Il-18

Sample-1 Il-18 cortex

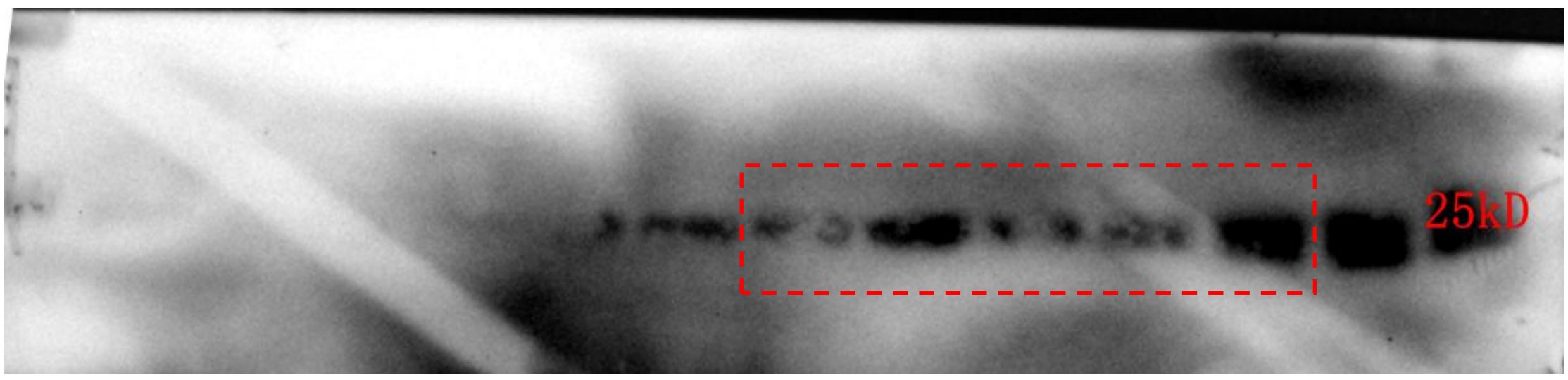

Sample-1 Tubulin-Cortex

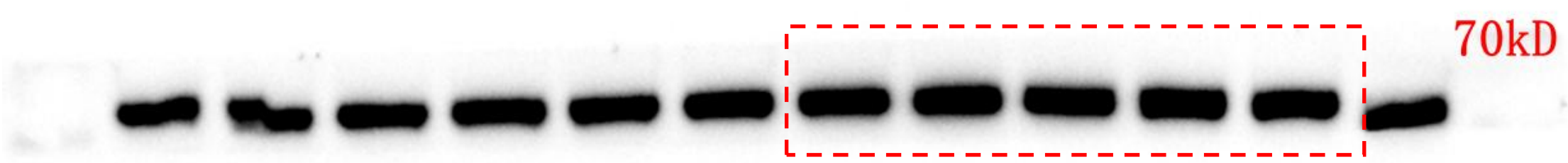

Sample-2 Il-18 cortex

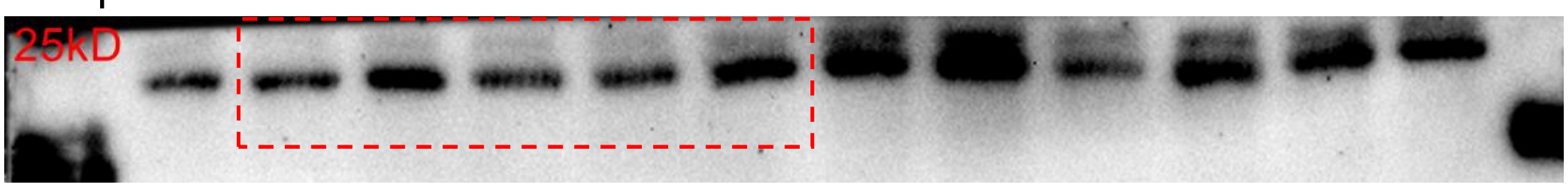

Sample-2 Tubulin-Cortex

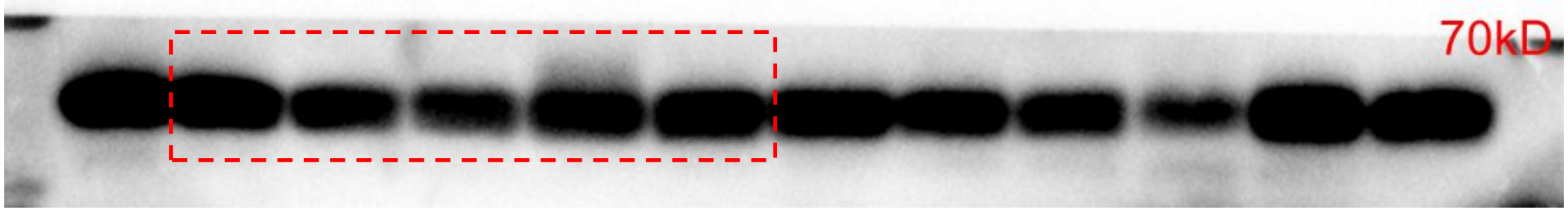

Sample-3 Il-18 cortex

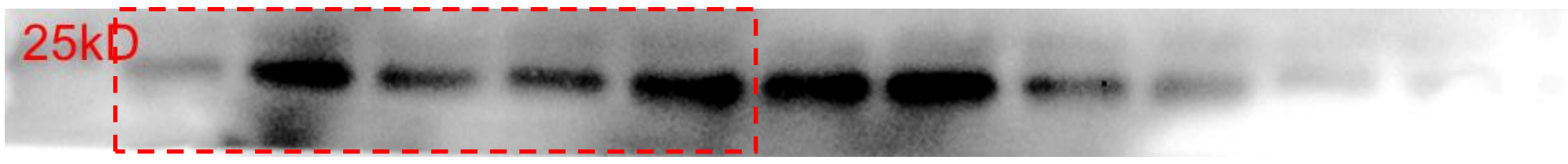

Sample-3Tubulin-Cortex

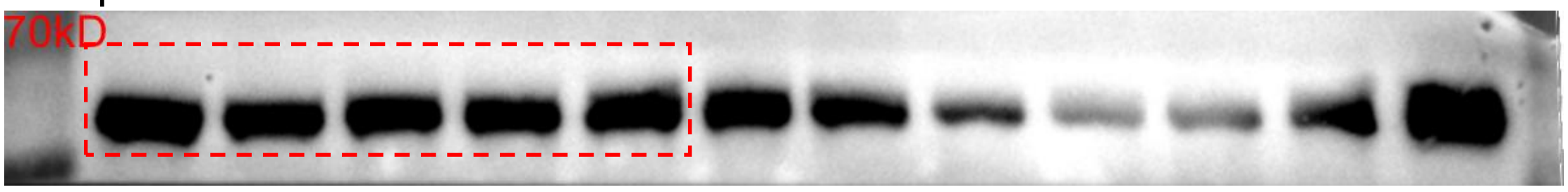

Sample-4 Il-18 cortex

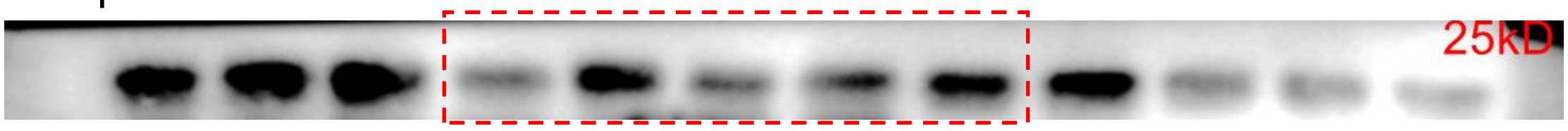

Sample-4 Il-18 tubulin

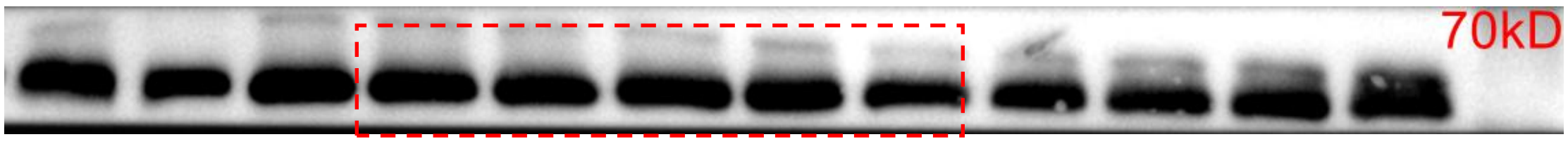

Sample-1 Il-18-Hippocampus

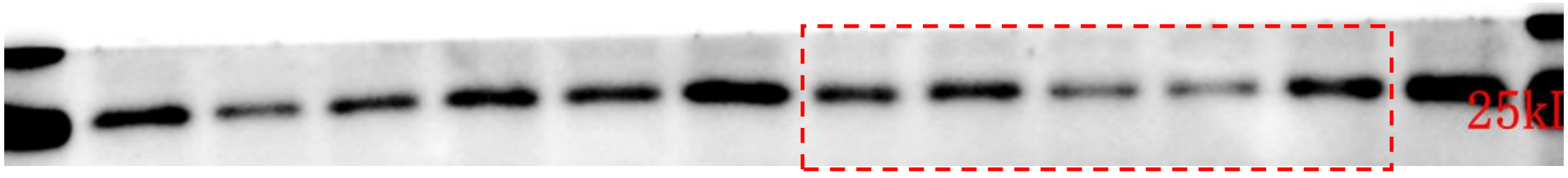

Sample-1 Tubulin-hippocampus

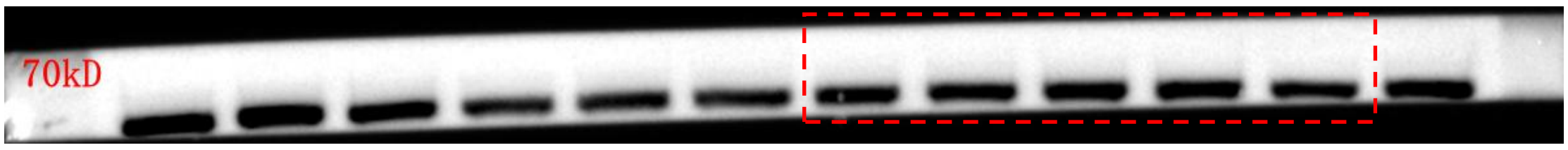

Sample-2 Il-18-Hippocampus

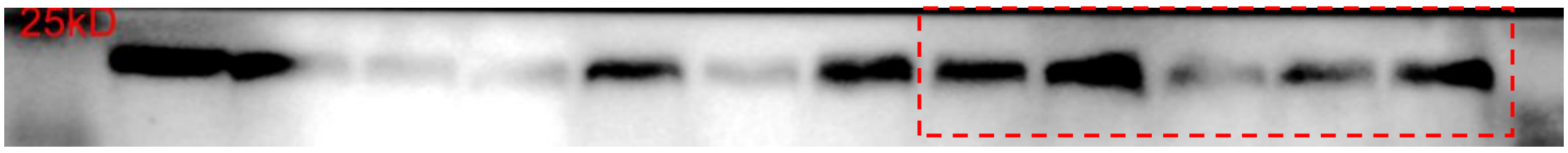

Sample-2 Tubulin-Hippocampus

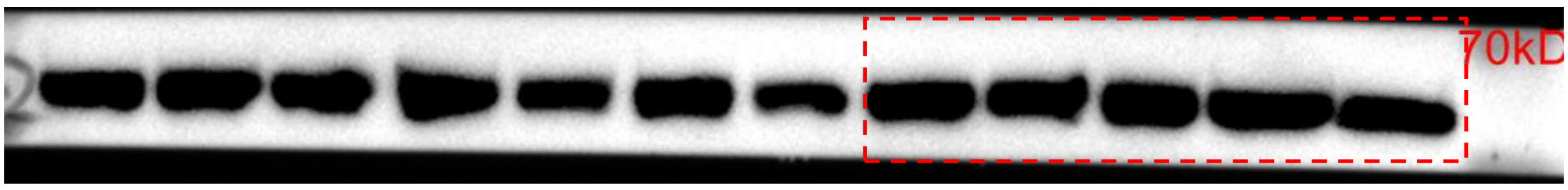

Sample-3 Il-18-Hippocampus

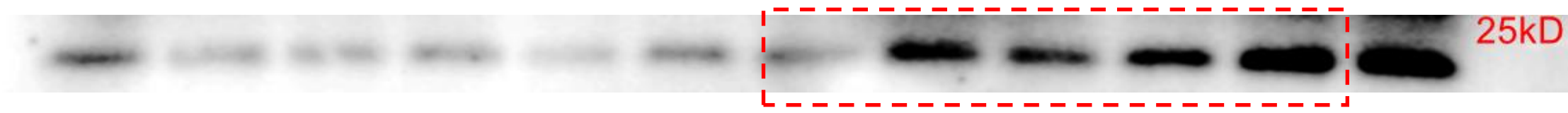

Sample-3 Tubulin-Hippocampus

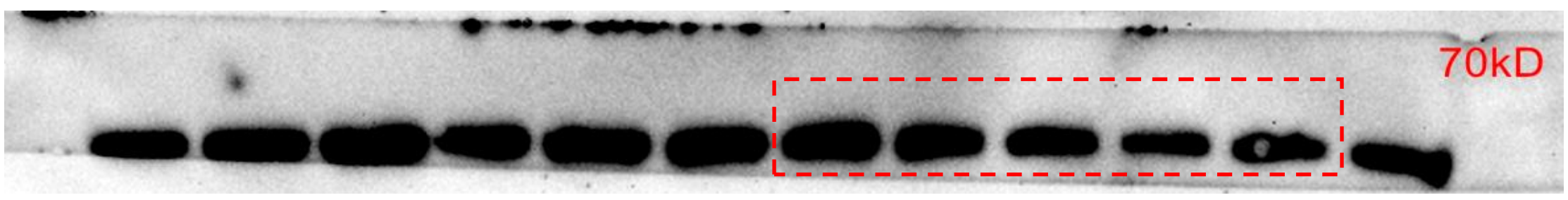

Sample-4 Il-18-Hippocampus

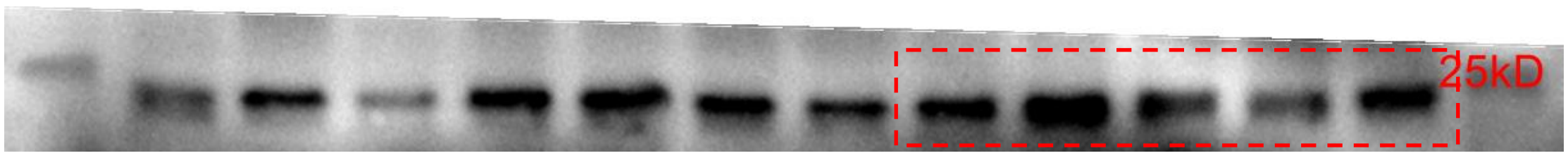

Sample-4 Tubulin-Hippocampus

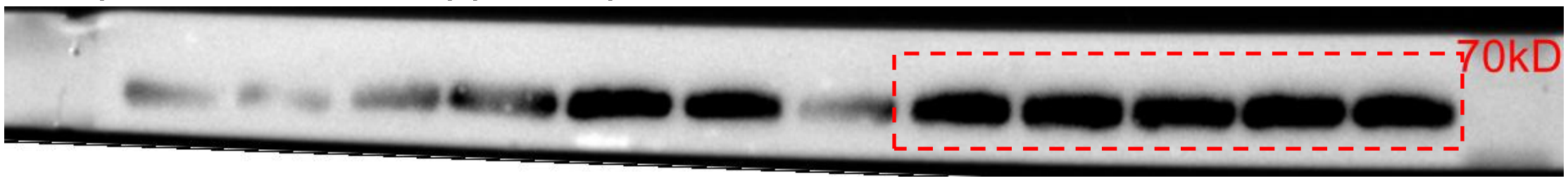

Figure 3c.Il-10

Sample-1 Il-10 Cortex

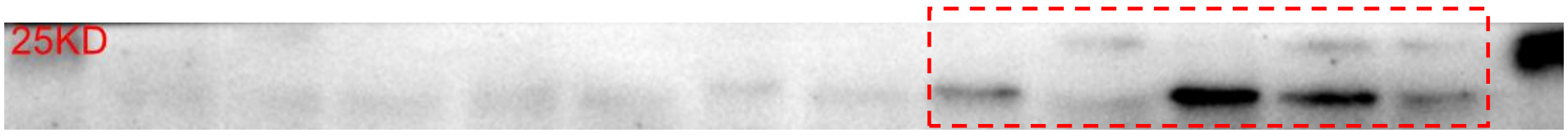

Sample-1 Tubulin-cortex

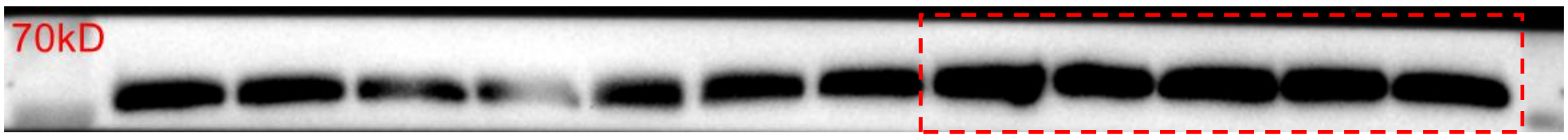

Sample-2 Il-10 Cortex

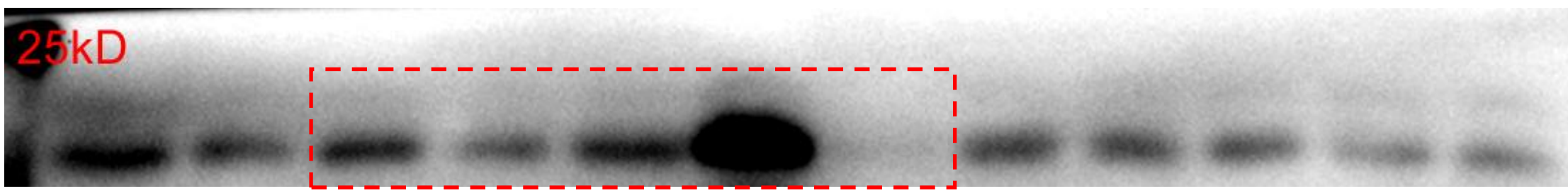

Sample-2 Tubulin Cortex

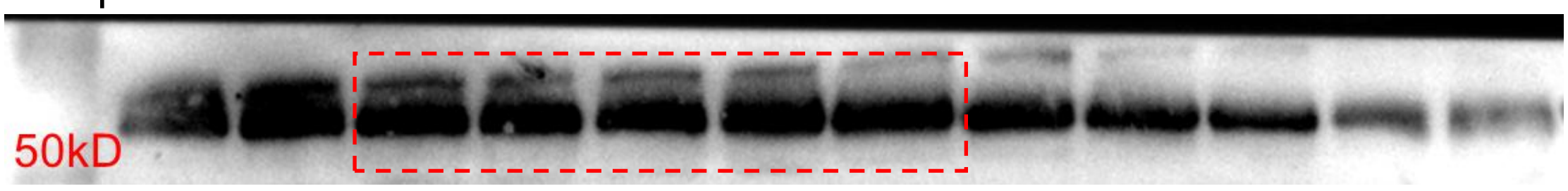

Sample-3 Il-10 Cortex

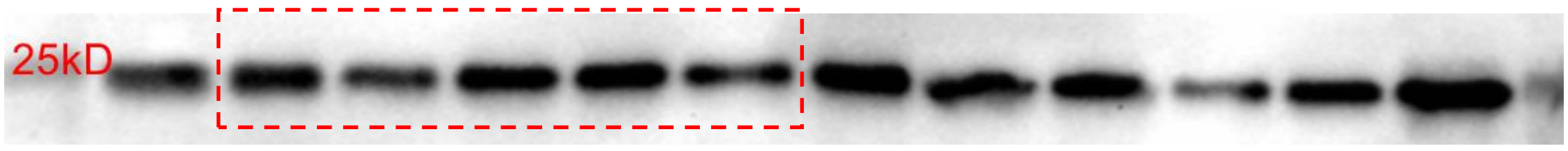

Sample-3 Tubulin Cortex

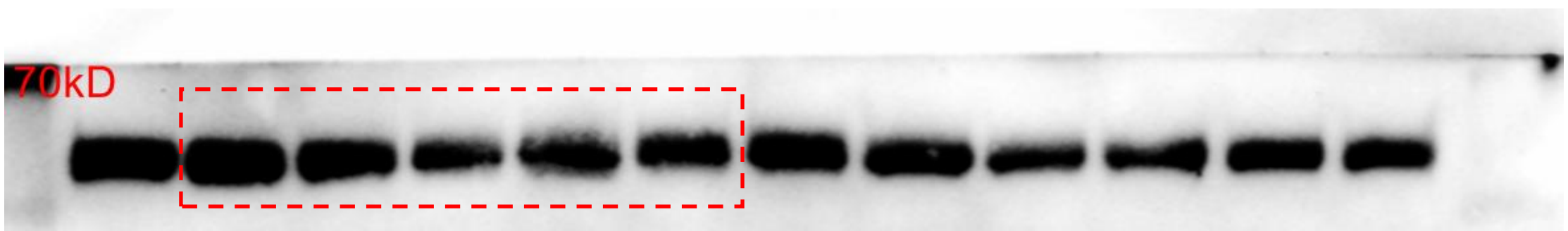

Sample-4 Il-10 Cortex

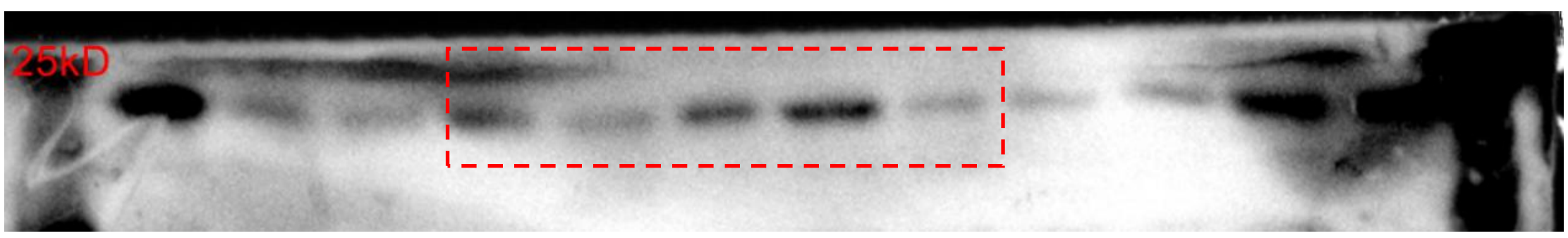

Sample-4 tubulin Cortex

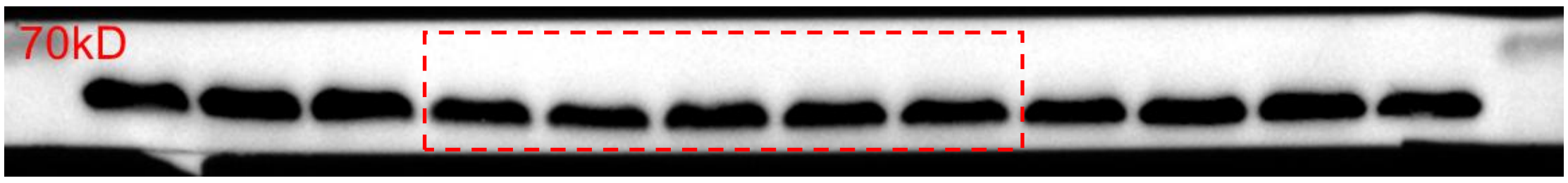

Sample-1 Il-10 hippo

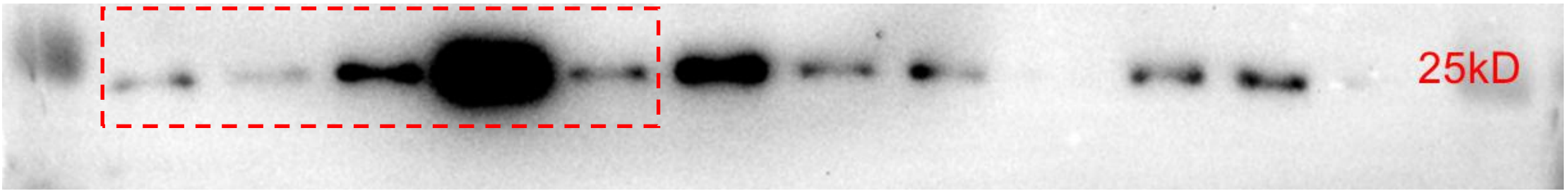

Sample-1 Tubulin hippo

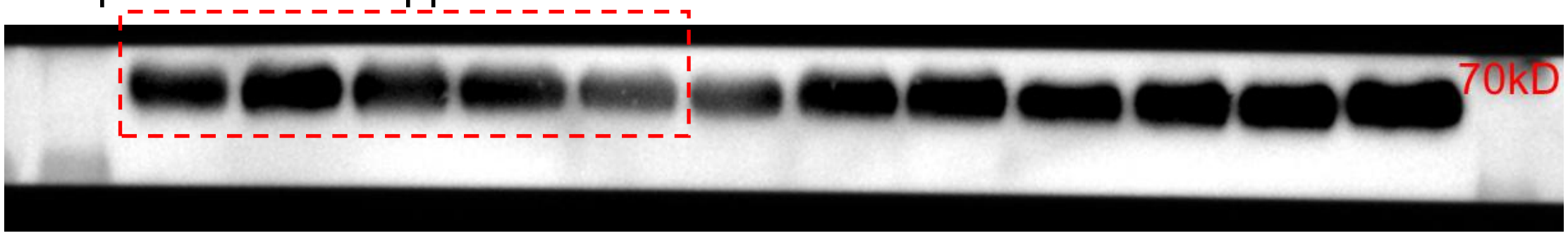

Sample-2 Il-10 hippo

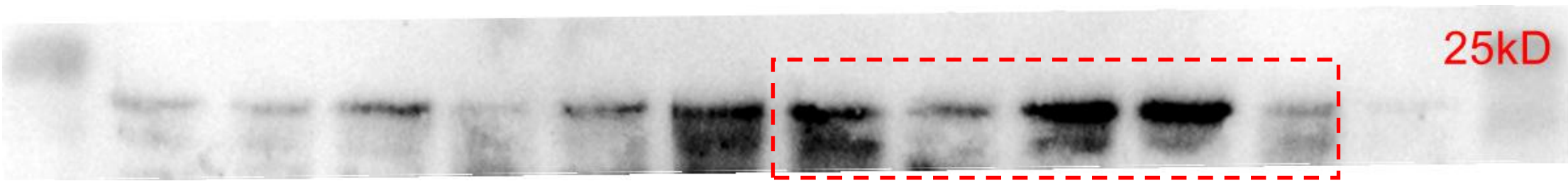

Sample-2 Tubulin hippo

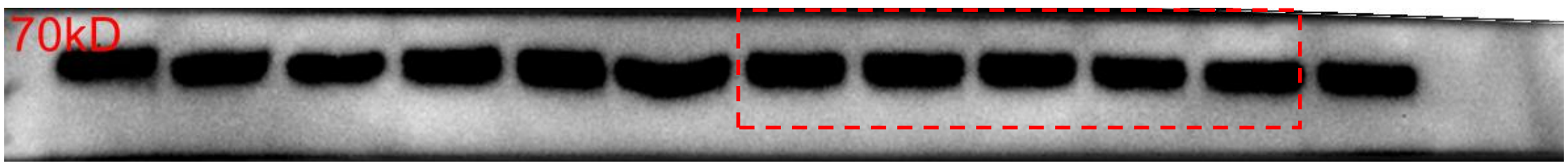

Sample-3 Il-10 hippo

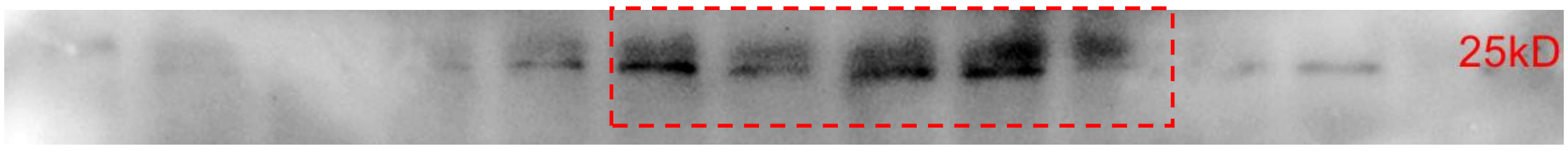

Sample-3 Tubulin hippo

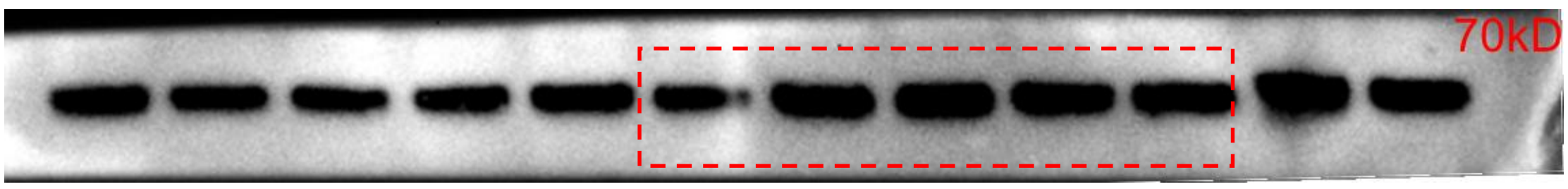

Sample-4 Il-10 hippo

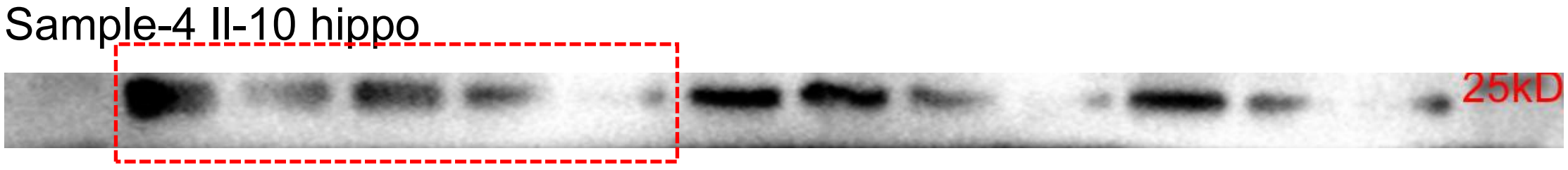

Sample-4 Tubulin hippo

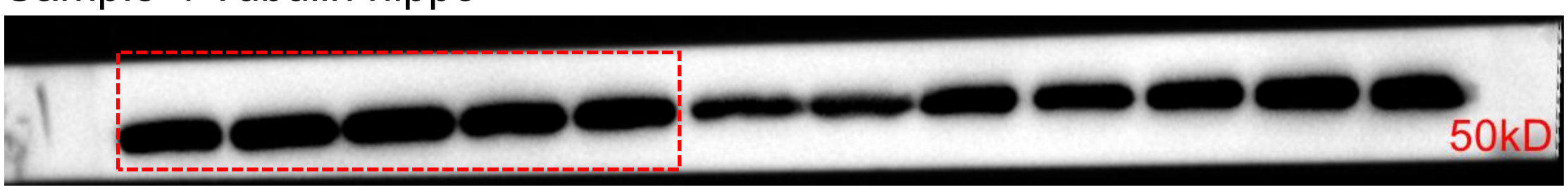

Figure 5.

Lanes within the red dotted boxes, arranged left to right: Sham, oAβ group, Oleic Acid (OA)+oAβ group,Oleic Acid (OA)+oAβ+Rapamycin (Rap) group, Oleic Acid (OA)+oAβ+Chloroquine(CQ) group.

Figure 5d. Atg5

Sample-1 Atg5

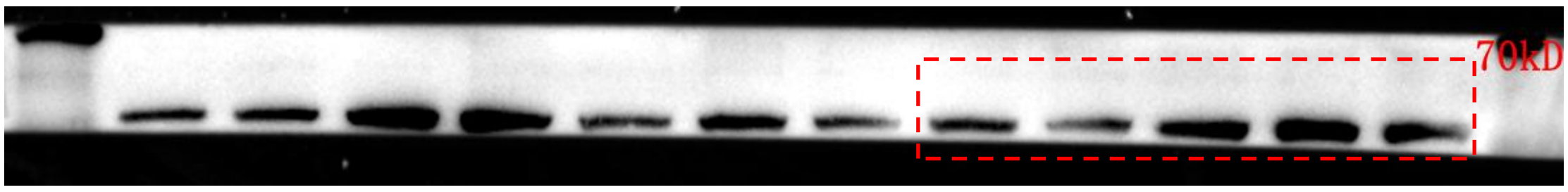

Sample-1 β Actin

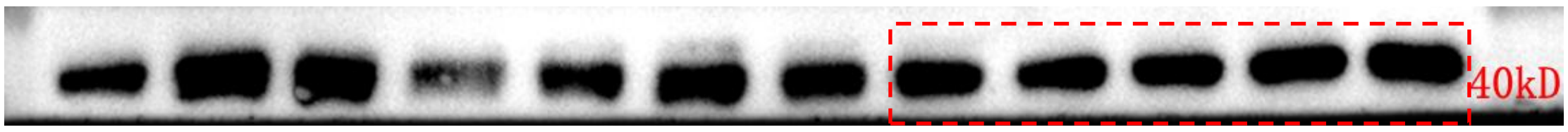

Sample-2 Atg5

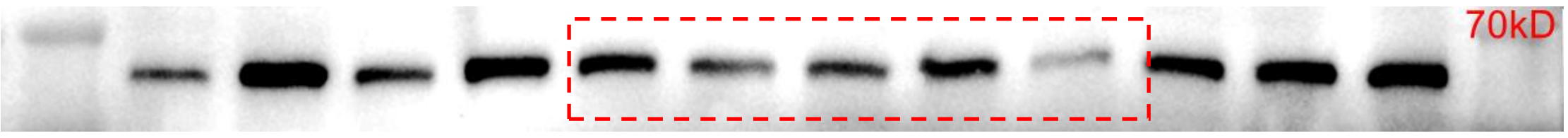

Sample-2 β Actin

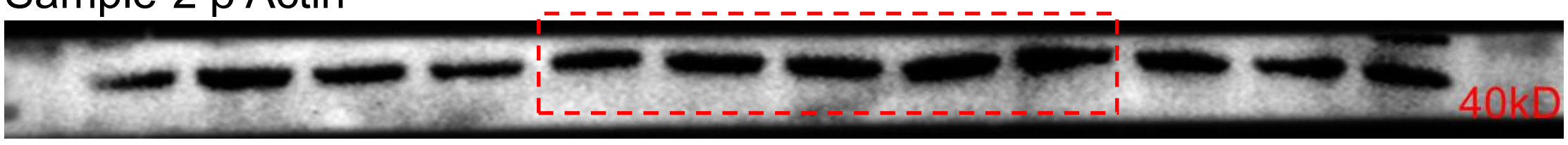

Sample-3 Atg5

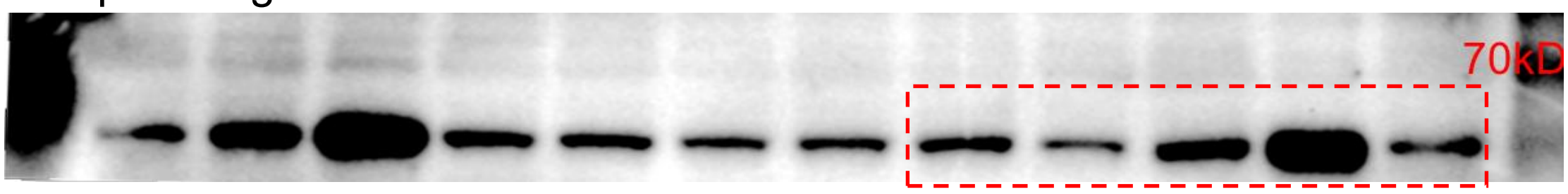

Sample-3 β Actin

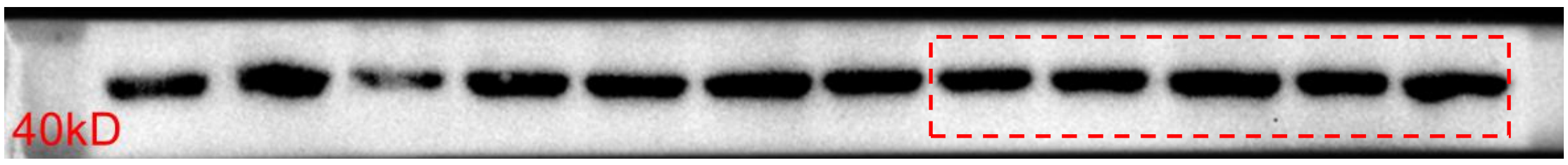

Figure 5f. Lc3II/Lc3I

Sample 1-Lc3II/Lc3I

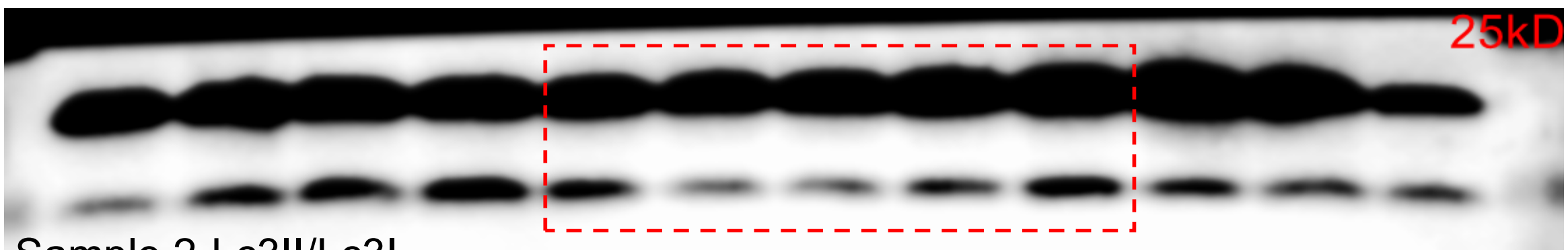

Sample 2-Lc3II/Lc3I

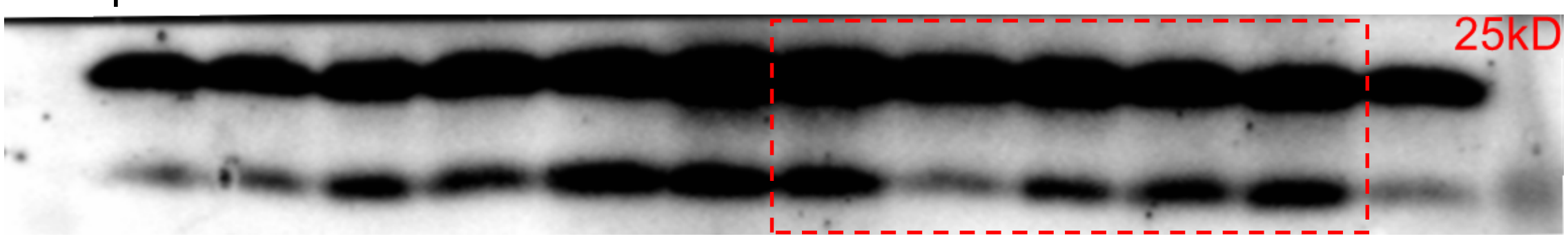

Sample 3-Lc3II/Lc3I

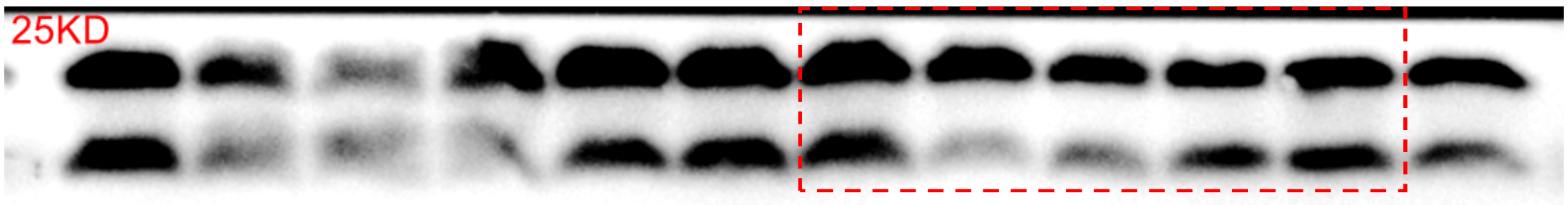

Sample 3-β-actin

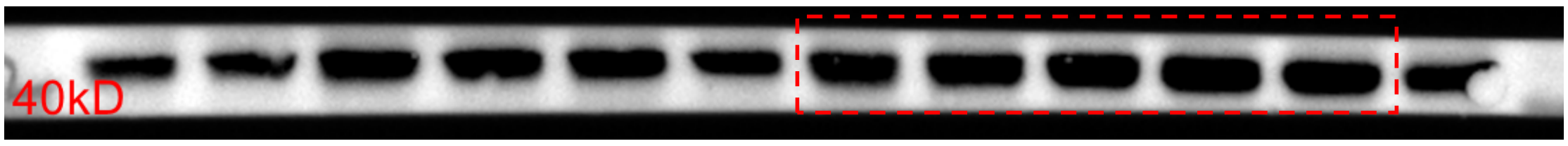

Figure 5h. Cpt1a

Sample 1-Cpt1a

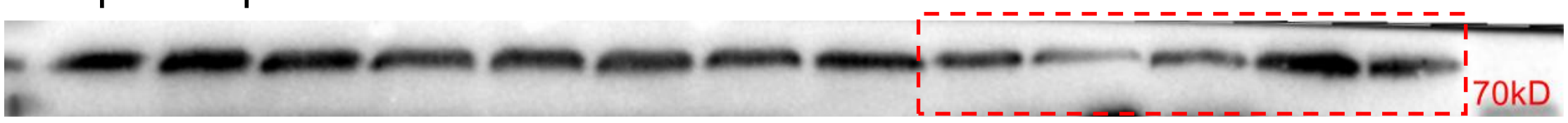

Sample 1-Gapdh

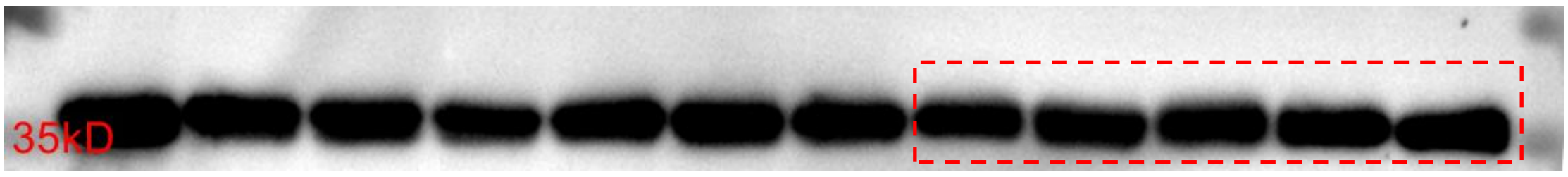

Sample 2-Cpt1a

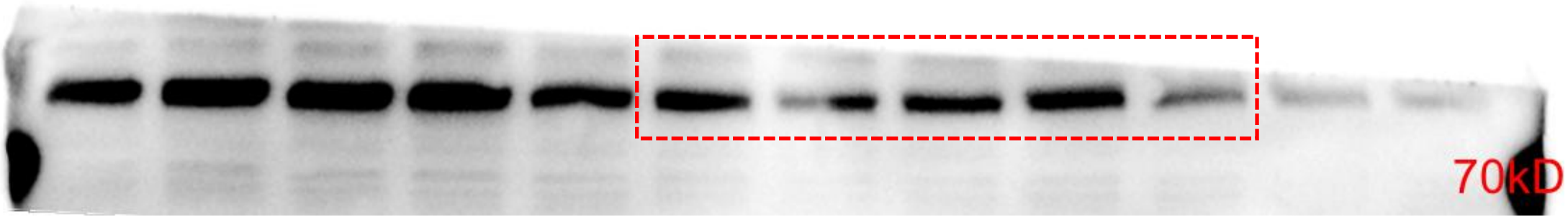

Sample 2-Gapdh

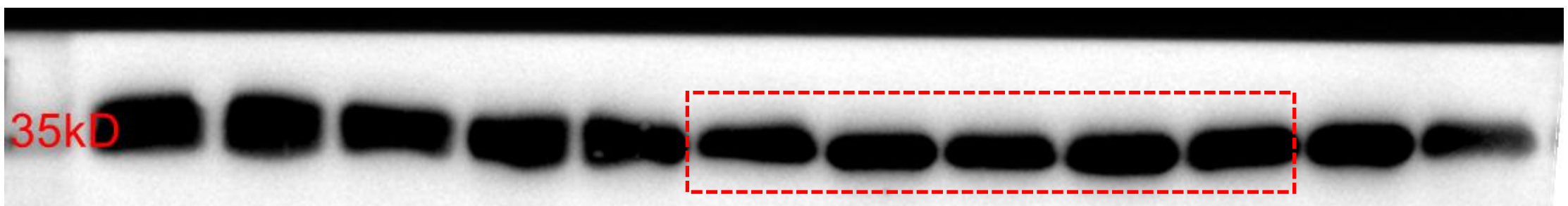

Sample 3-Cpt1a

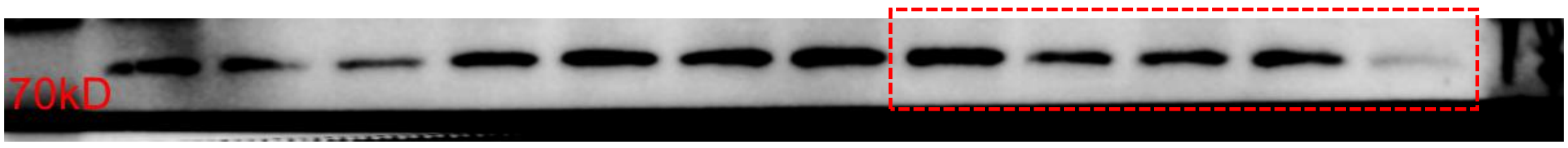

Sample 3-Gapdh

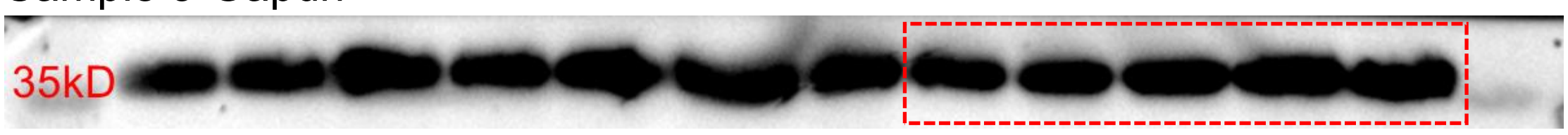

Figure 5j. Ndufb10

Sample 1-Ndufb10

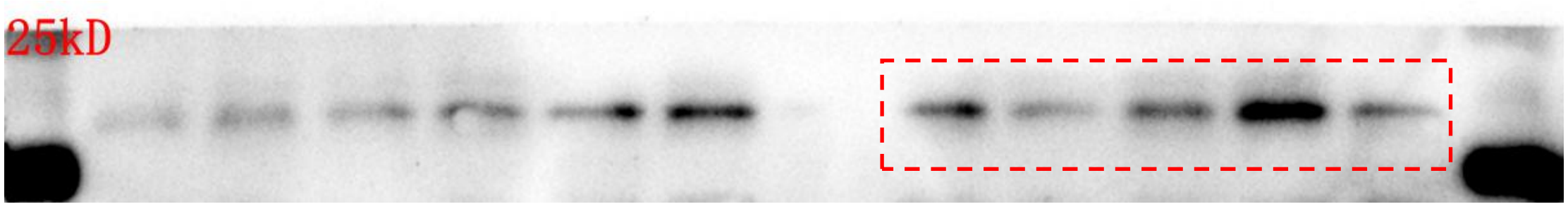

Sample 1-β tubulin

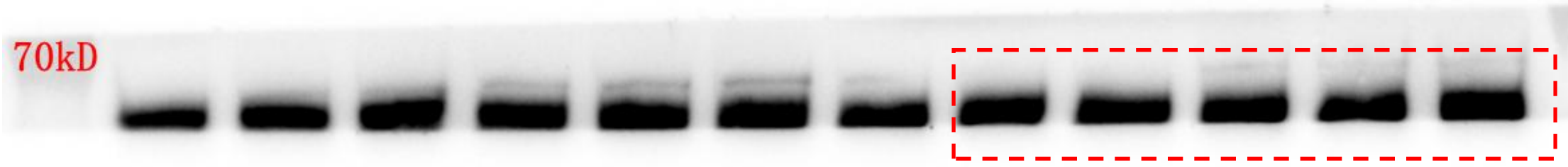

Sample 2-Ndufb10

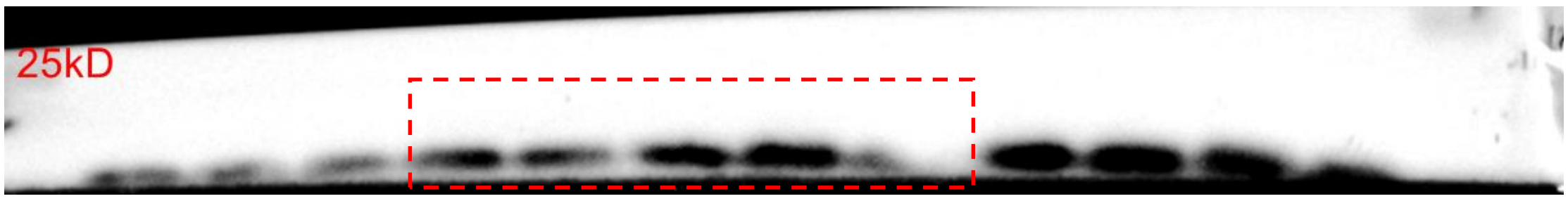

Sample 2-β tubulin

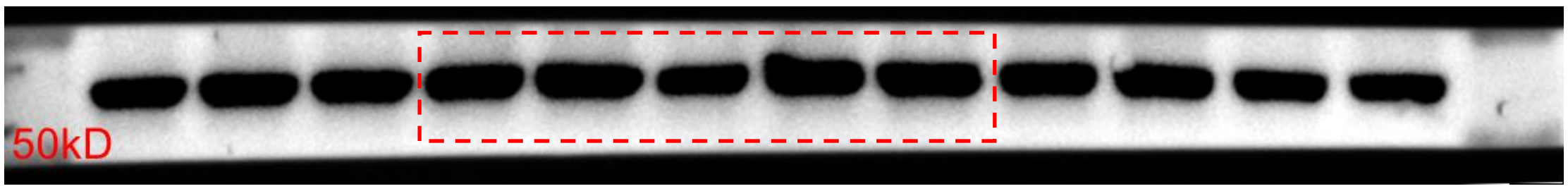

Sample 3-Ndufb10

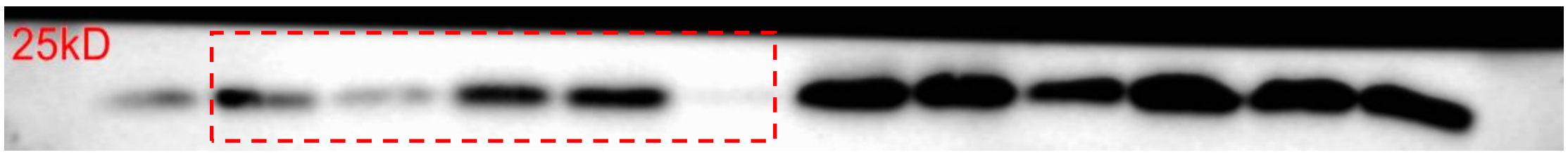

Sample 3-β tubulin

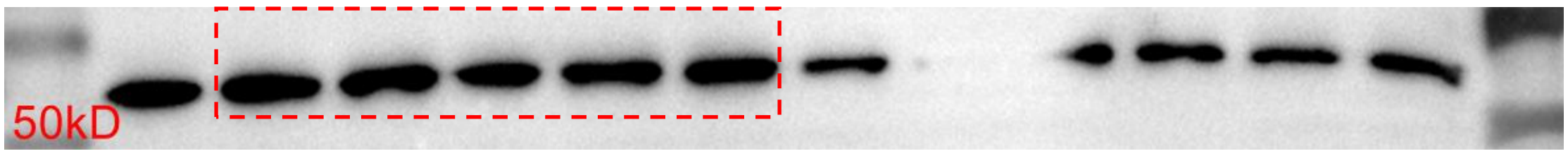

Figure 5l. Mtco2

Sample -1 MtCO2

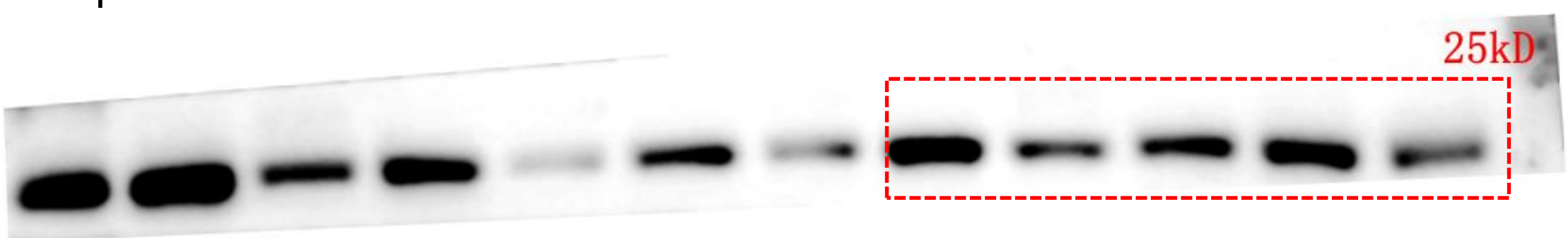

Sample -1 β actin

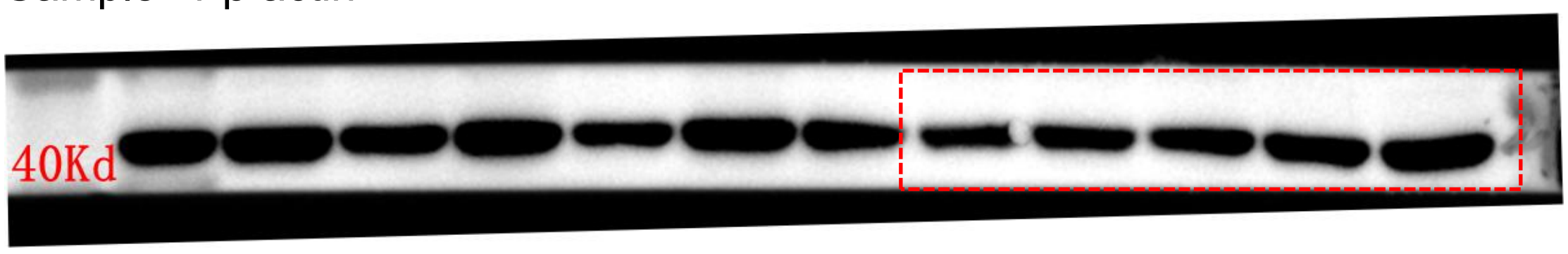

Sample -2 MtCO2

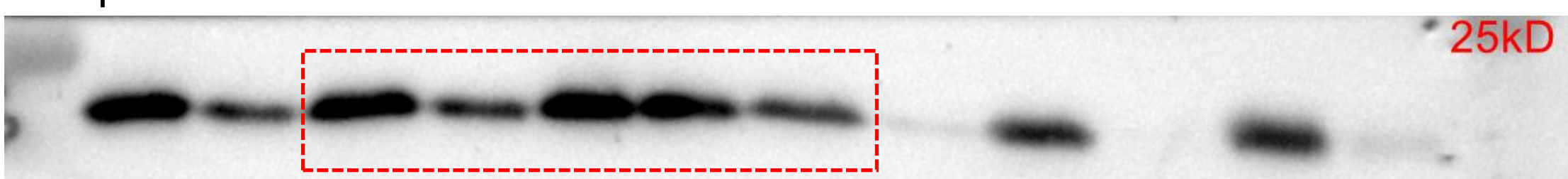

Sample -2 β actin

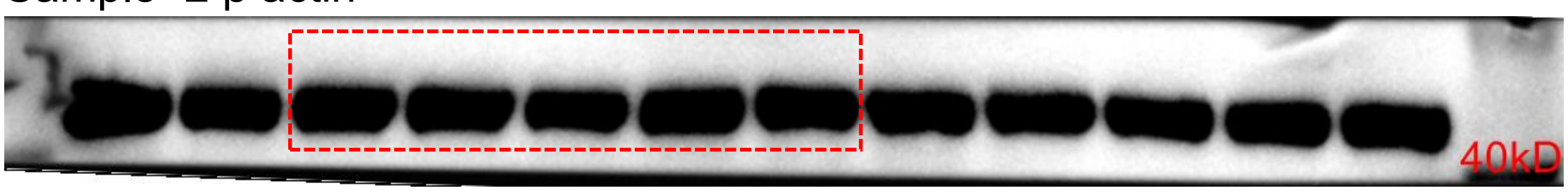

Sample -3 MtCO2

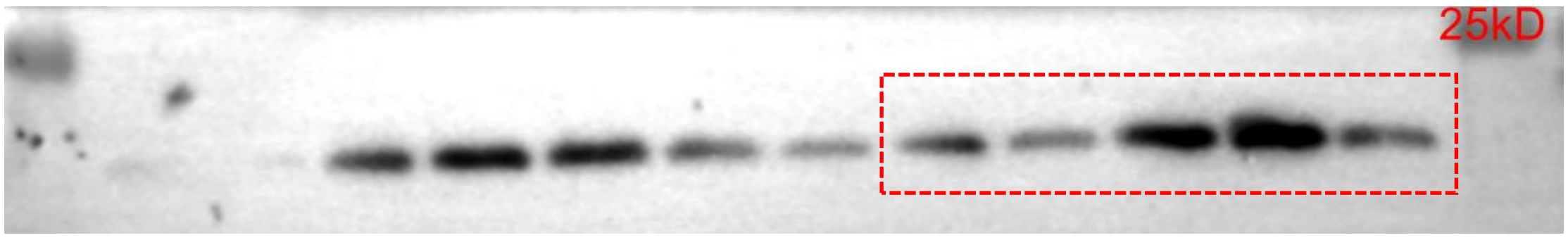

Sample -3 β actin

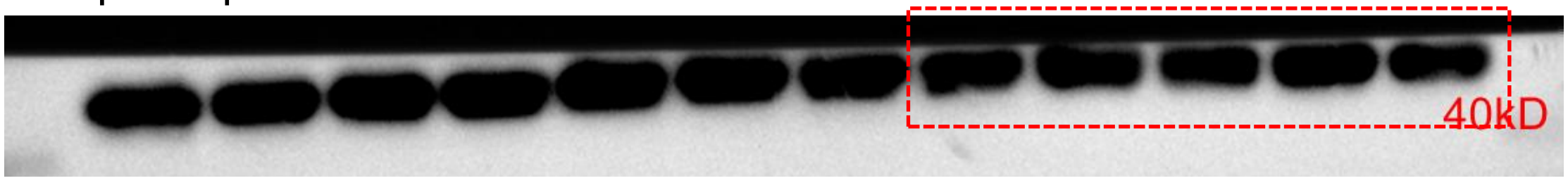

Figure 5n. Atpaf1,Sdhb,Nlrp3, Inos, Cleaved Il-1 β ,Il-10

Figure 5n. Atpaf1

Sample 1 Atpaf1

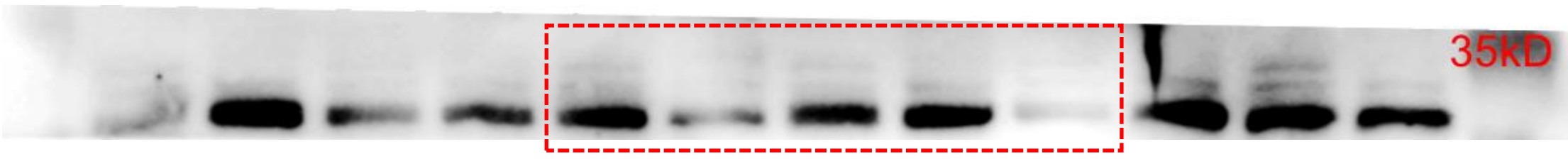

Sample 1 β-Tubulin

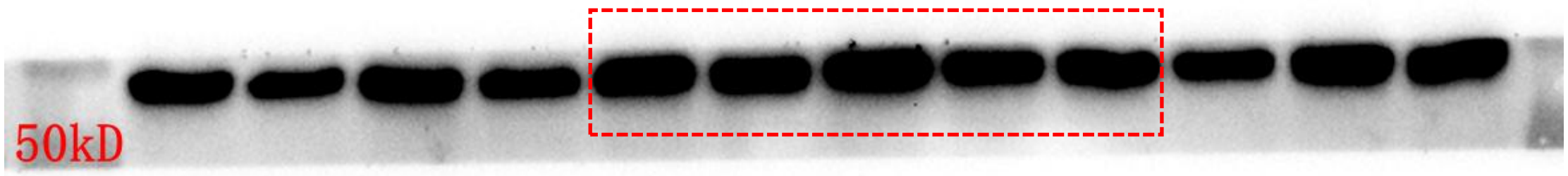

Sample 2-Atpaf1

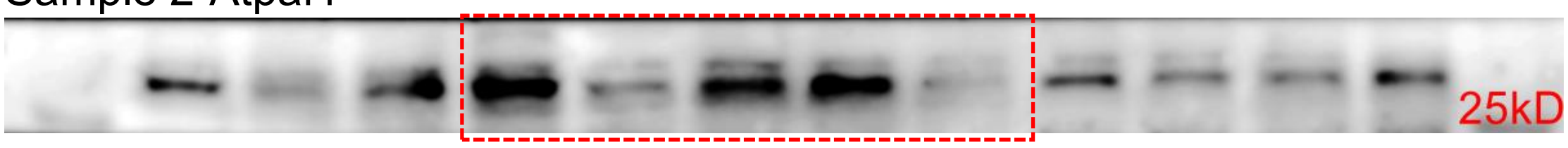

Sample 2 β-Tubulin

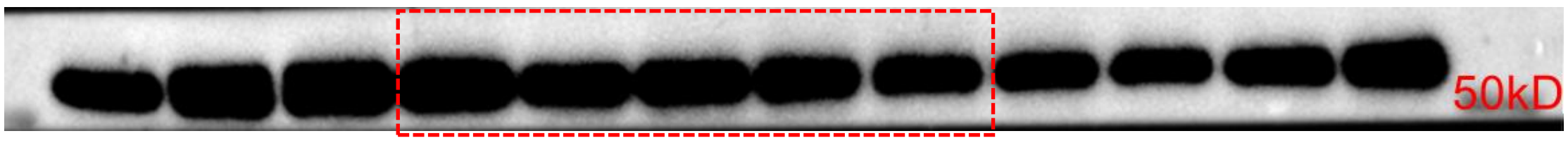

Sample 3 Atpaf1

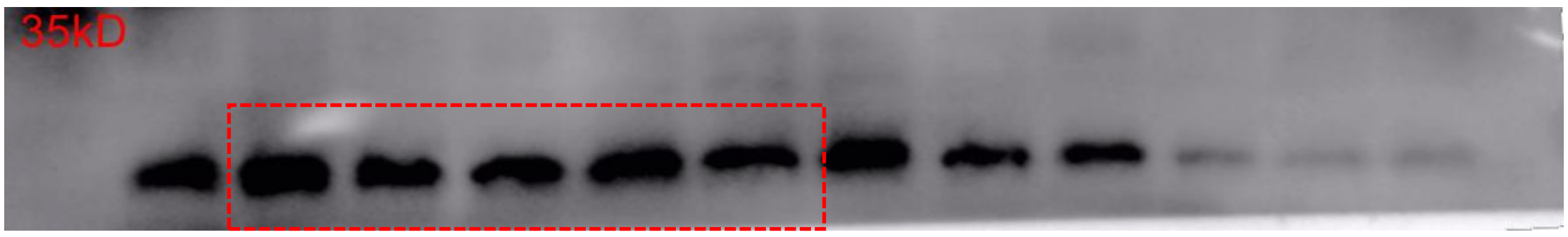

Sample 3 β-Tubulin

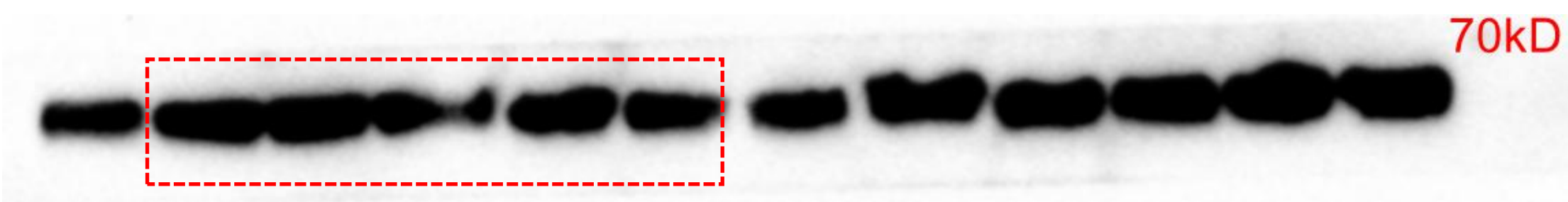

Figure 5n. Sdhb

Smple 1 Sdhb

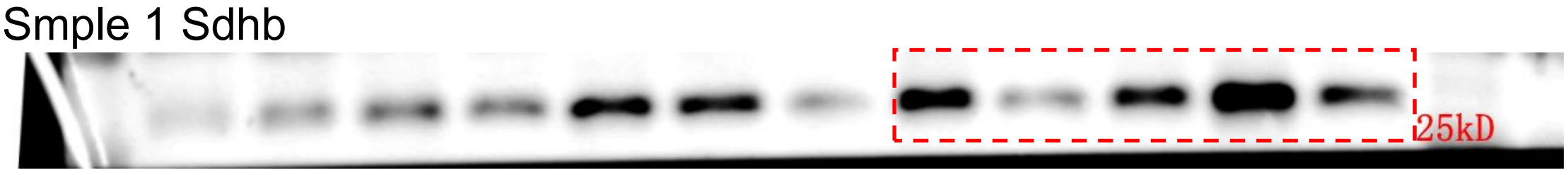

Smple 1 β Actin

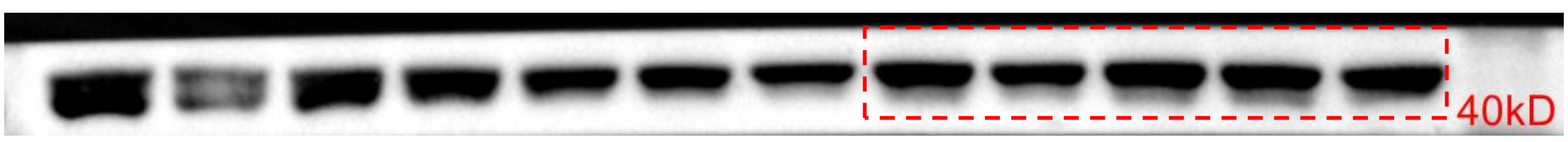

Smple 2 Sdhb

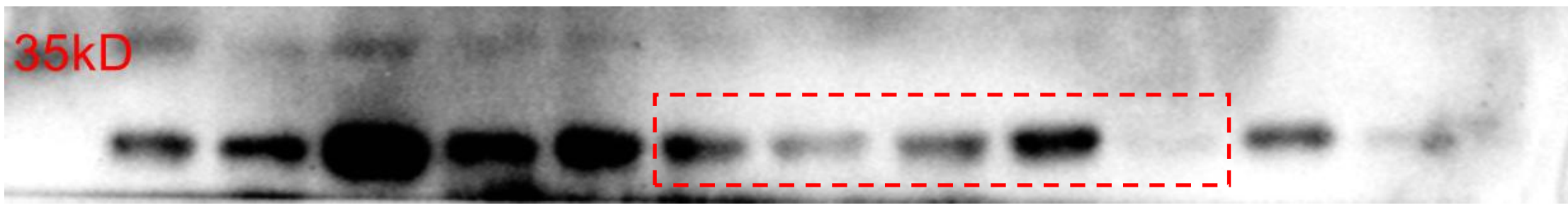

Smple 2 β Actin

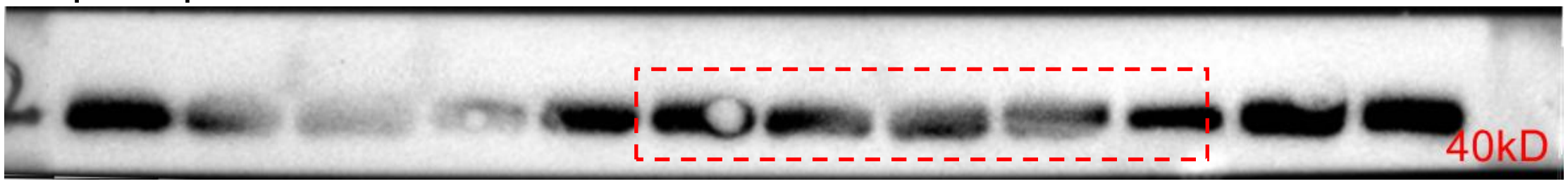

Smple 3 Sdhb

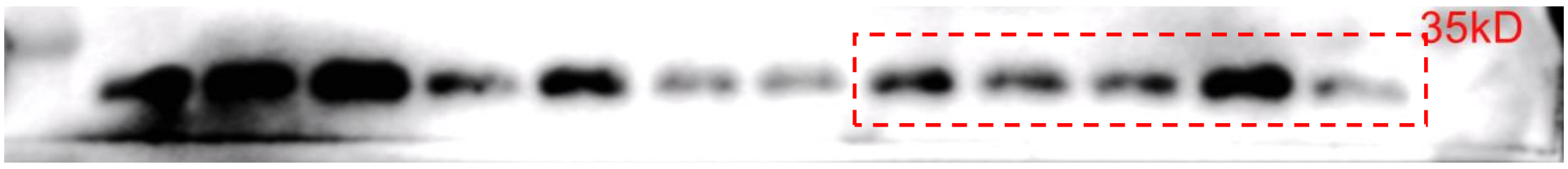

Smple 3 β Actin

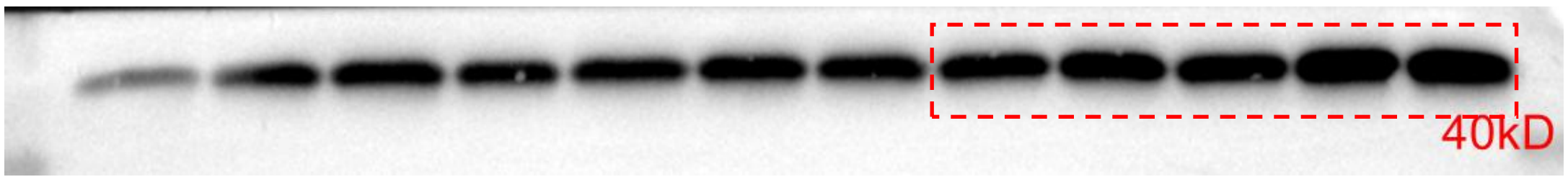

Figure 5n. Nlrp3

Sample 1 Nlrp3

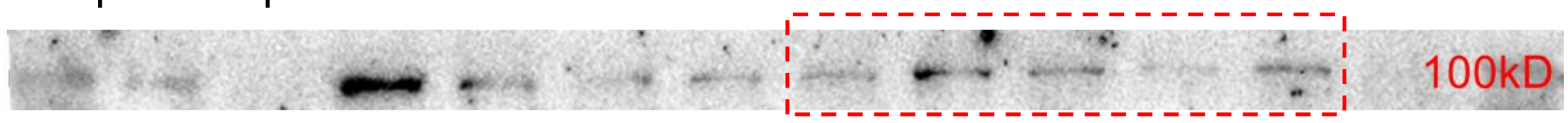

Sample 1 β-Actin

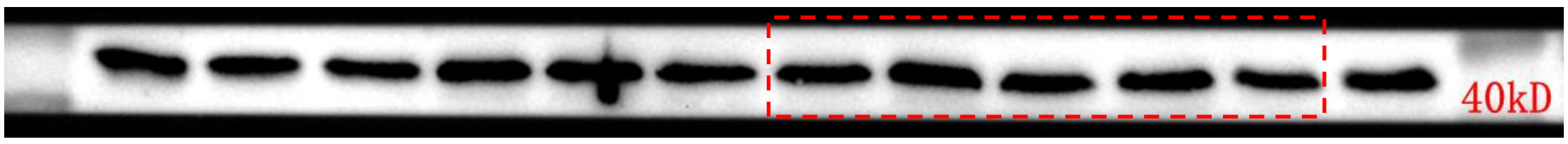

Sample 2 Nlrp3

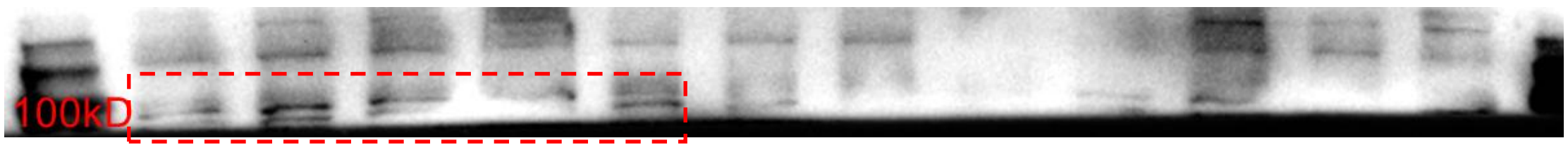

Sample 2 β-Actin

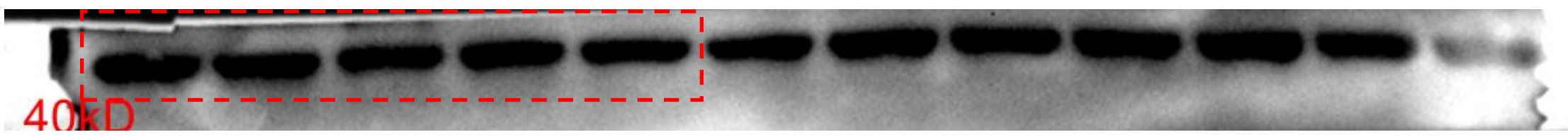

Sample 3 Nlrp3

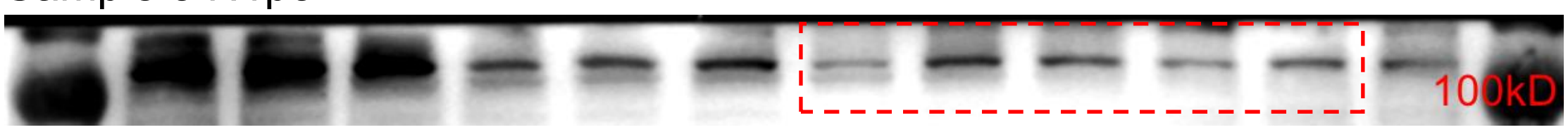

Sample 3 β-Actin

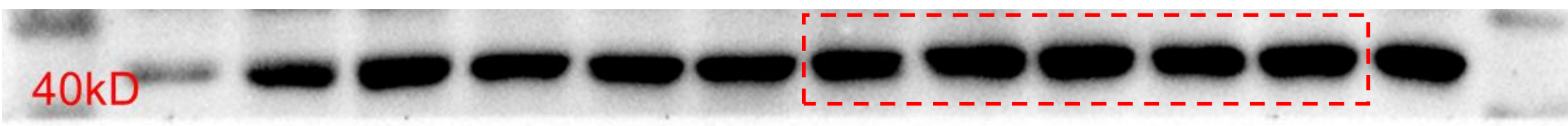

Figure 5n. Inos,Cleaved Il-1 β

Sample 1 Inos

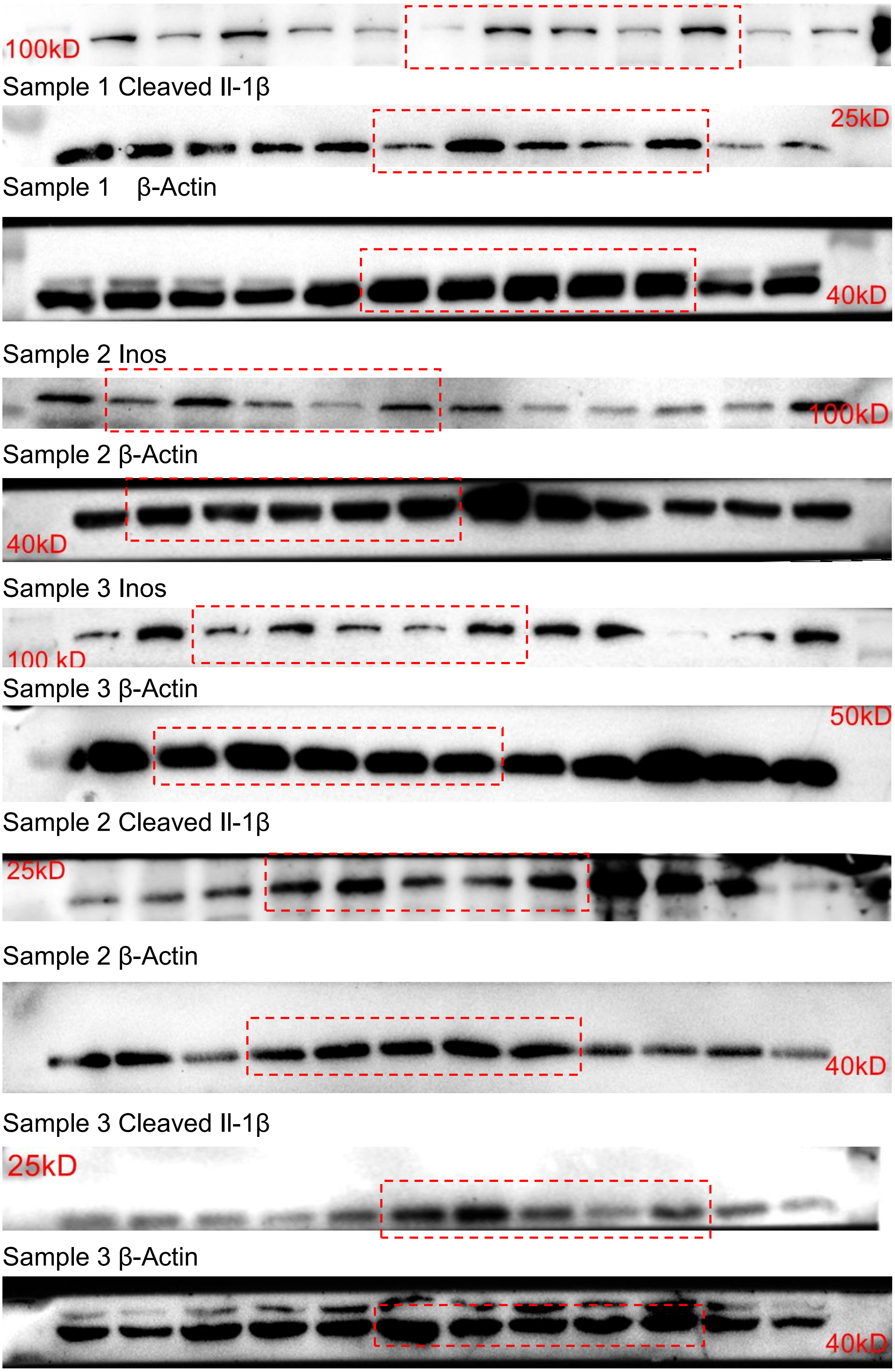

100kD
Sample 1 Cleaved Il-1β
25kD
Sample 1 β-Actin
40kD
Sample 2 Inos
100kD
Sample 2 β-Actin
40kD
Sample 3 Inos
100 kD
Sample 3 β-Actin
50kD
Sample 2 Cleaved Il-1β
25kD
Sample 2 β-Actin
40kD
Sample 3 Cleaved Il-1β
25kD
Sample 3 β-Actin
40kD

Figure 5n. Il-10

Sample 1 Il-10

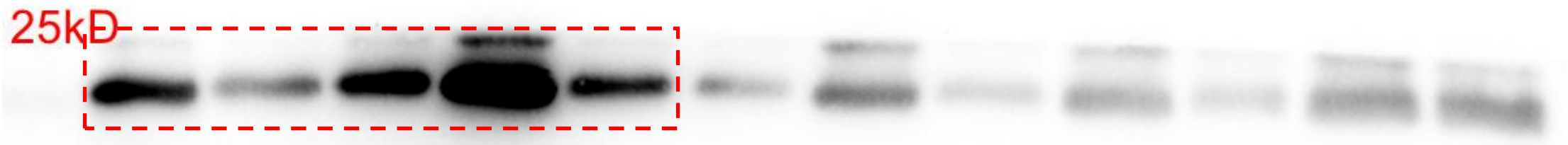

Sample 1 β-tubulin

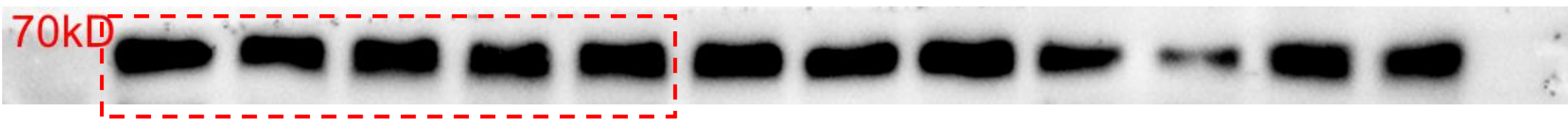

Sample 2 Il-10

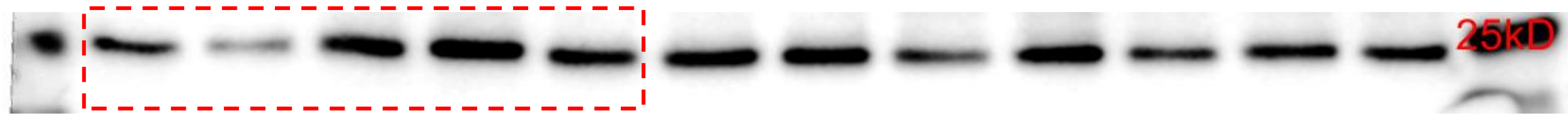

Sample 2 β-tubulin

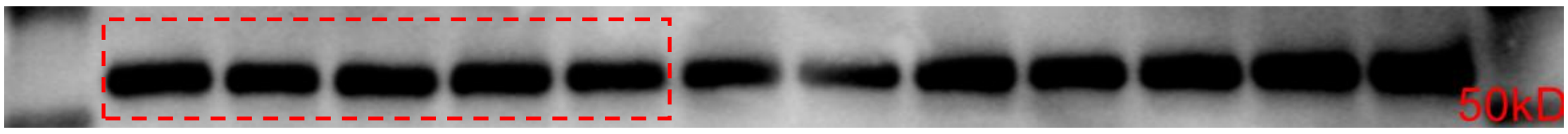

Sample 3 Il-10

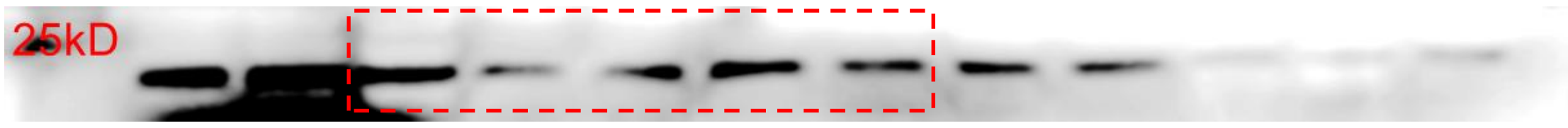

Sample 3 β-tubulin

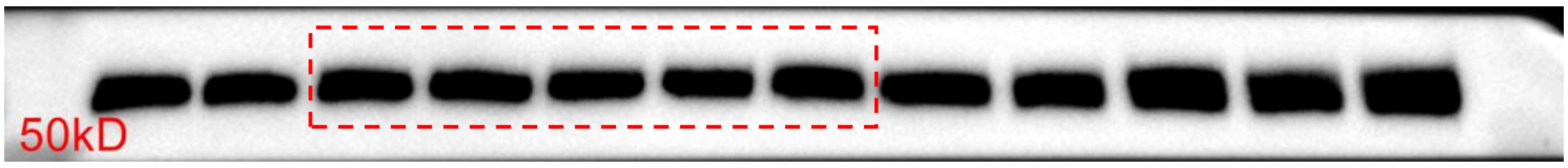

Figure 6a. p-Ampkα/Ampkα for mice brain.

Lanes within the red dotted boxes, arranged left to right: WT group, Control-5xFAD group, PE-5xFADgroup, RNAi-Tmem9-5xFAD group, PE+OE--5xFAD group.

Sample-1 Ampkα-Cortex

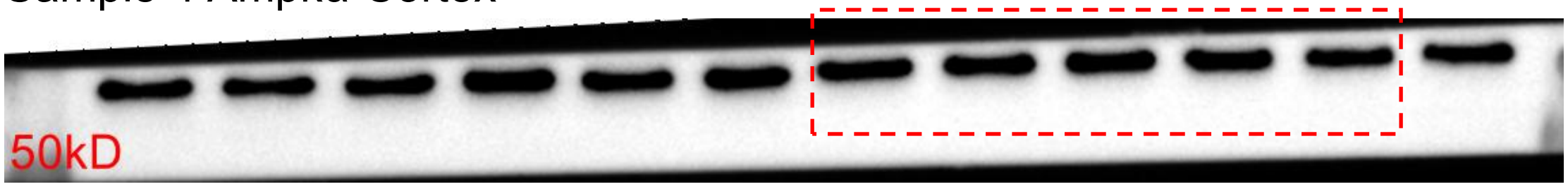

Sample-1 p-Ampkα-Cortex

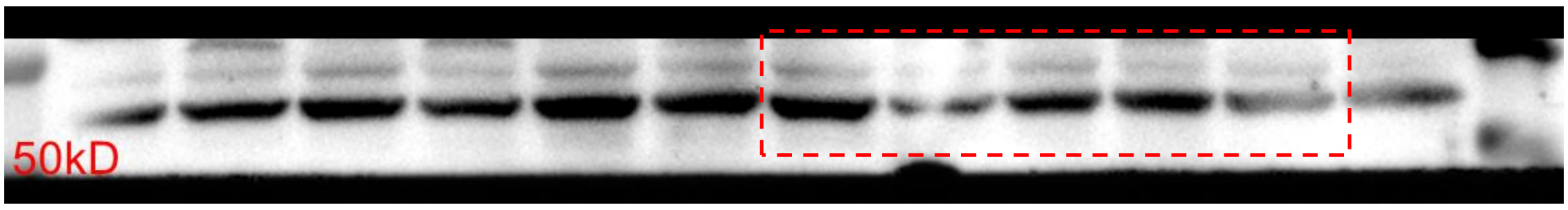

Sample-1 Gapdh-Cortex

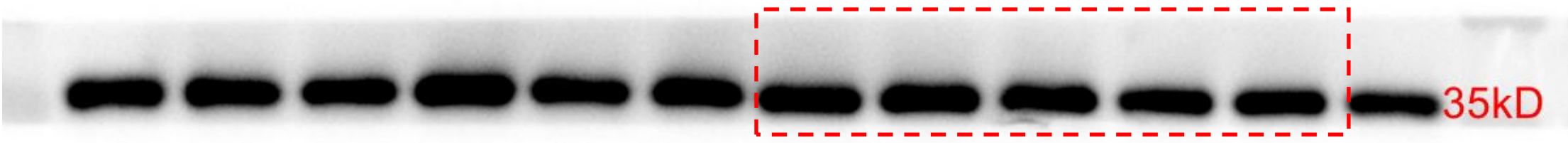

Sample-2 Ampkα-Cortex

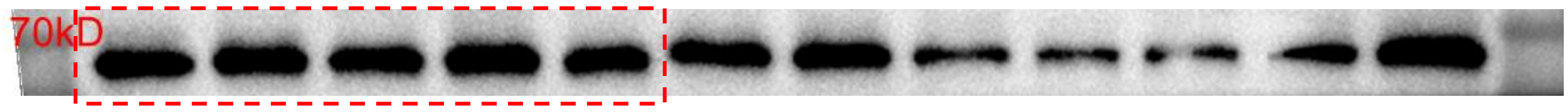

Sample-2 p-Ampkα-Cortex

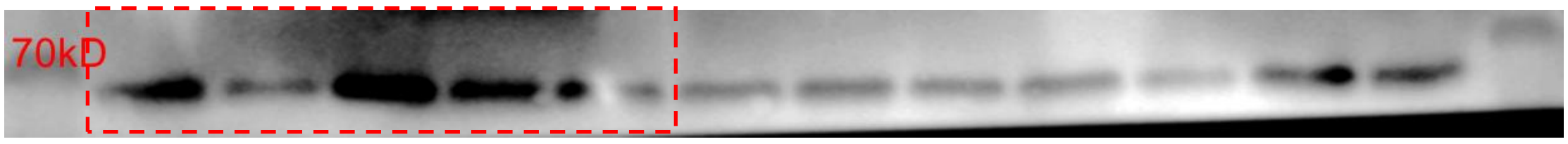

Sample-3 Ampkα-Cortex

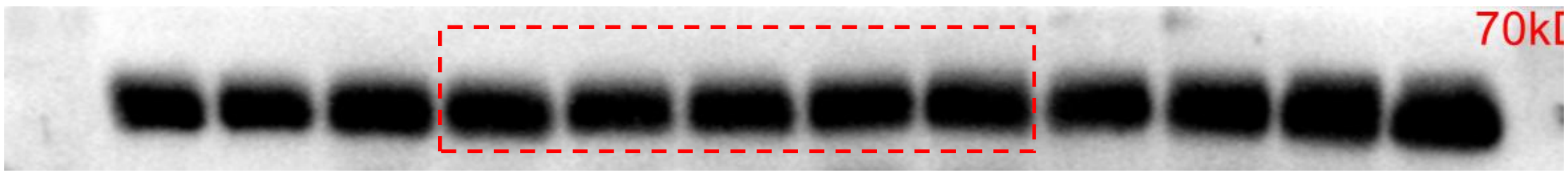

Sample-3 p-Ampkα-Cortex

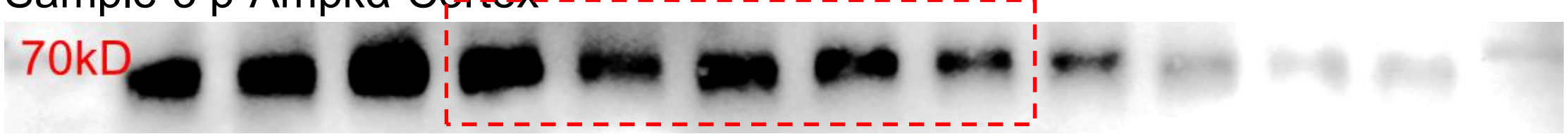

Sample-4 Ampkα-Cortex

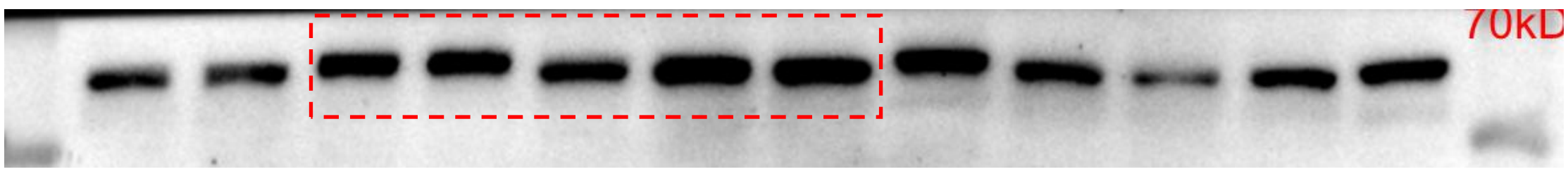

Sample-4 p-Ampkα-Cortex

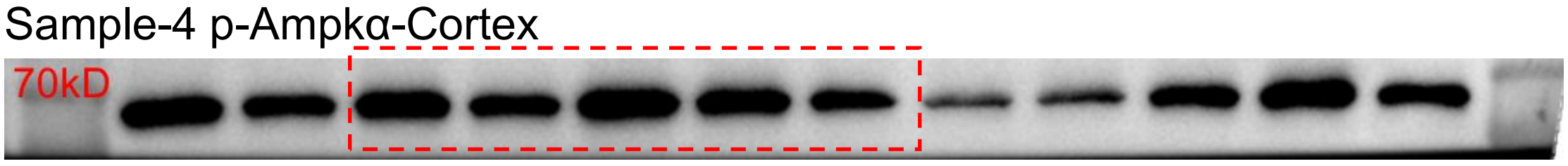

Sample-1 Ampkα-Hippo

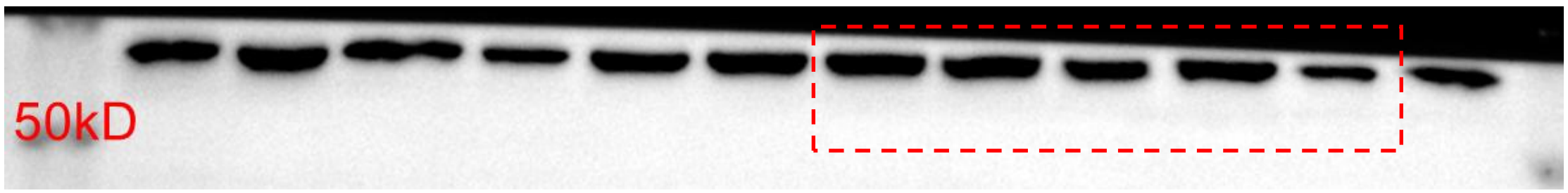

Sample-1 P-Ampkα-Hippo

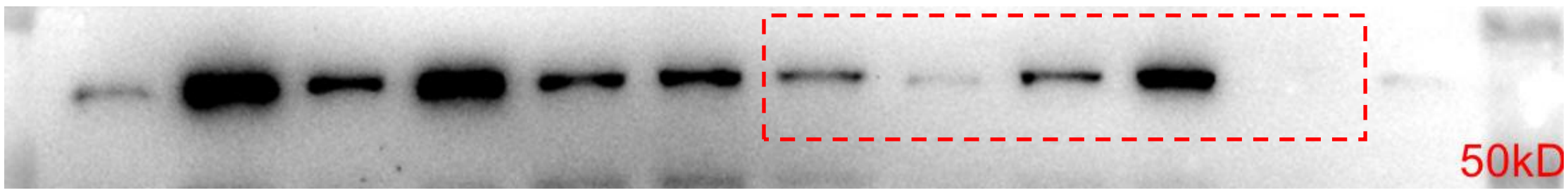

Sample-1 Gapdh-Hippo

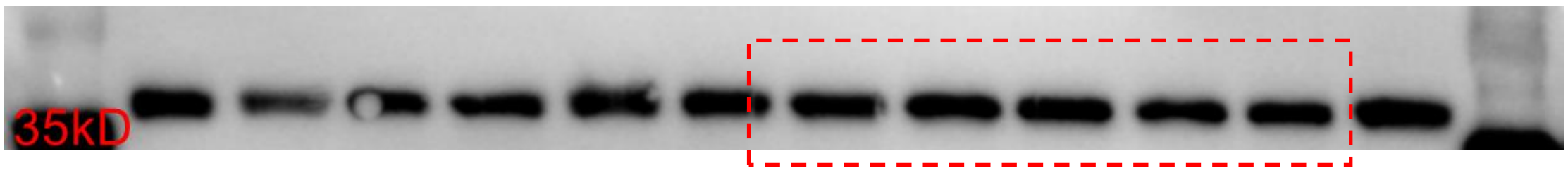

Sample-2 Ampkα-Hippo

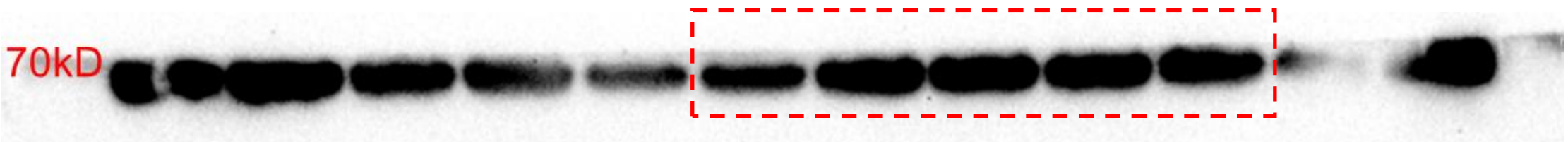

Sample-2 p-Ampkα-Hippo

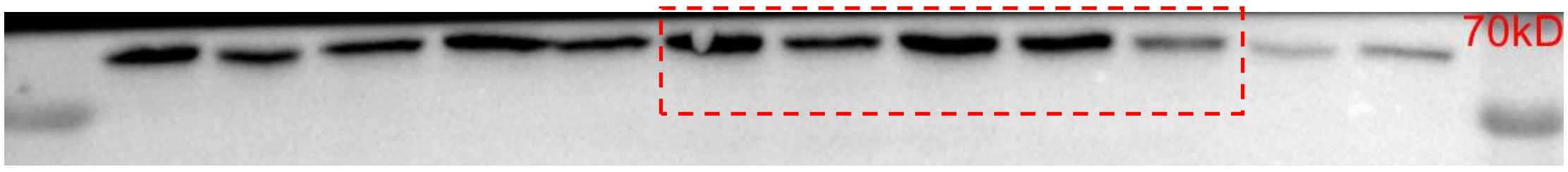

Sample-3 Ampkα-Hippo

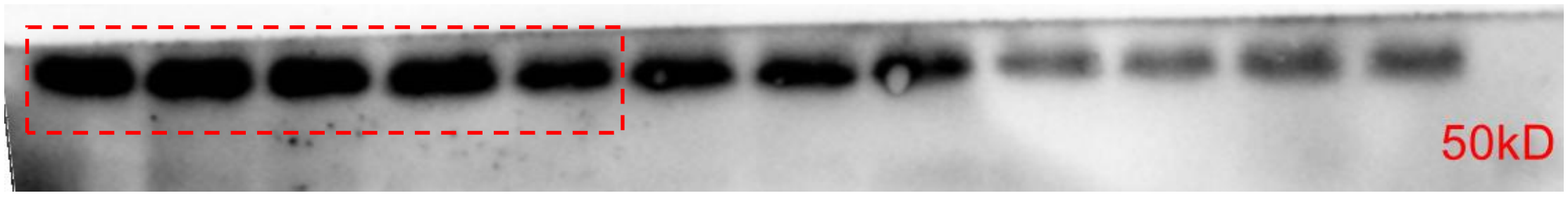

Sample-3 p-Ampkα-Hippo

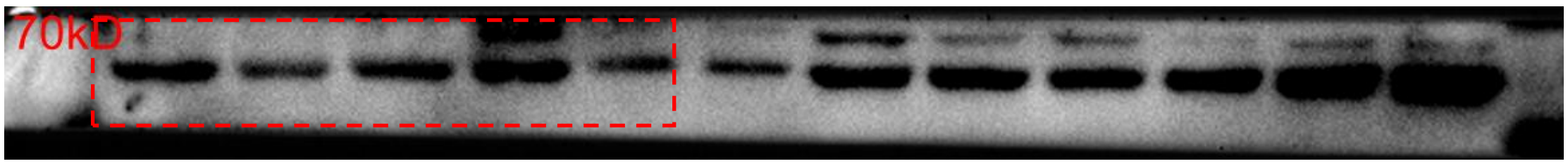

Sample-4 Ampkα-Hippo

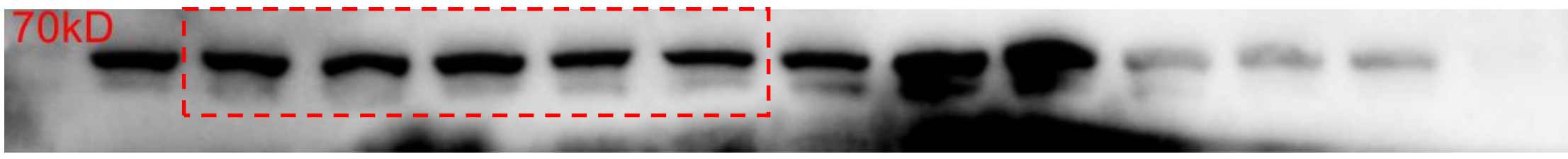

Sample-4 p-Ampkα-Hippo

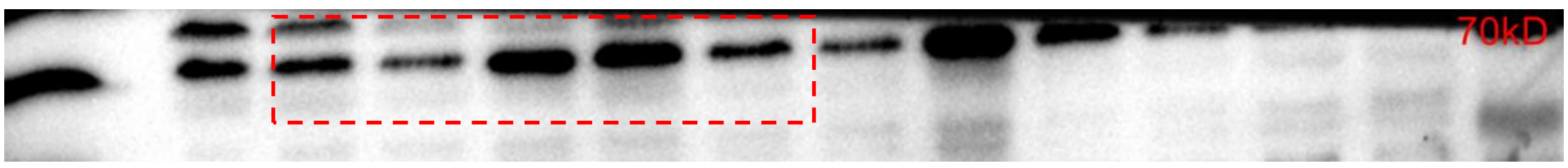

Figure 6c. **p-Ampkα/Ampkα for BV2 cells with OA and oAβ treatment.**

Lanes within the red dotted boxes, arranged left to right: Scramble, ShRNA-Tmem9, Negative control and Tmem9 overexpression groups.

Sample 1 AMPKα

Sample 1 AMPKα

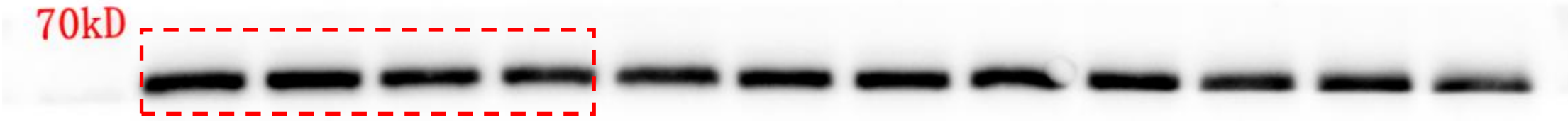

Sample 1 p-AMPKα

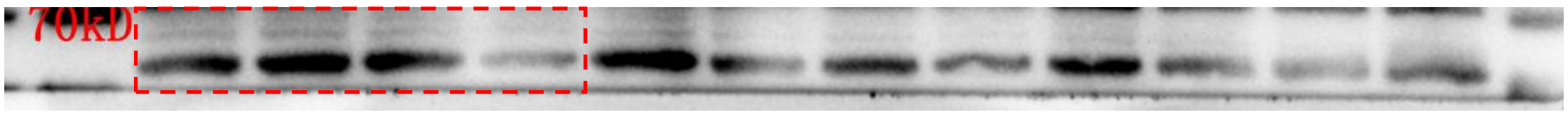

Sample 2+Sample-3 AMPKα

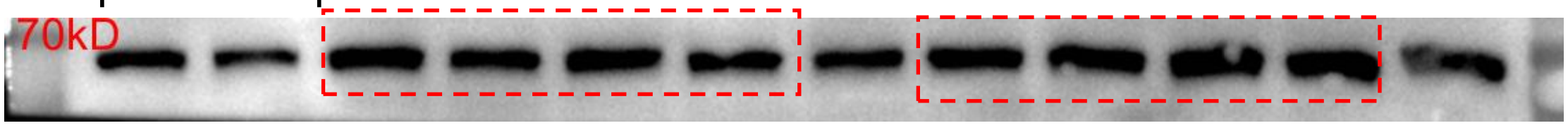

Sample 2+Sample-3 p-AMPKα

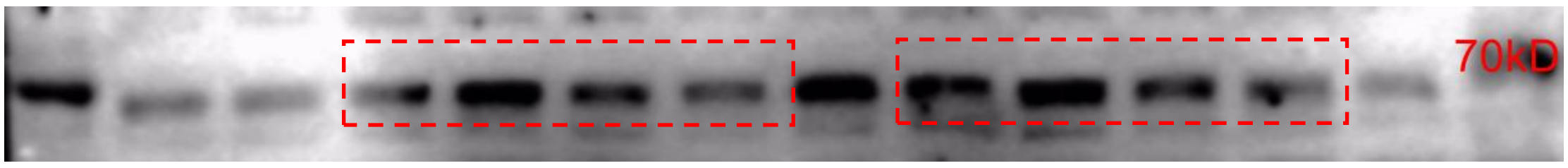

Figure 6e-q. BV2 microglial cells transfected with Tmem9-specific shRNA and subsequently treated with oleic acid (OA), dimethyl sulfoxide (DMSO), or Compound C prior to oligomeric amyloid-beta (oAβ) exposure. Lanes within the red dotted boxes, arranged left to right: DMSO group, Compound C (CC) group.

Figure 6e. p-Ampkα/Ampkα

Sample 1+Sample 2 AMPKα

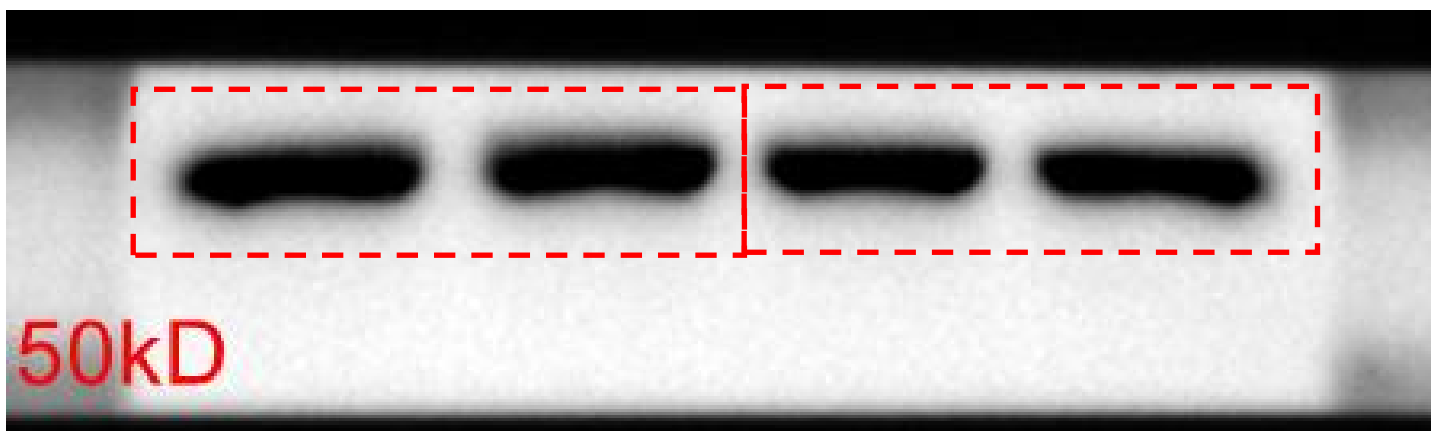

Sample 1+Sample 2 P-AMPKα

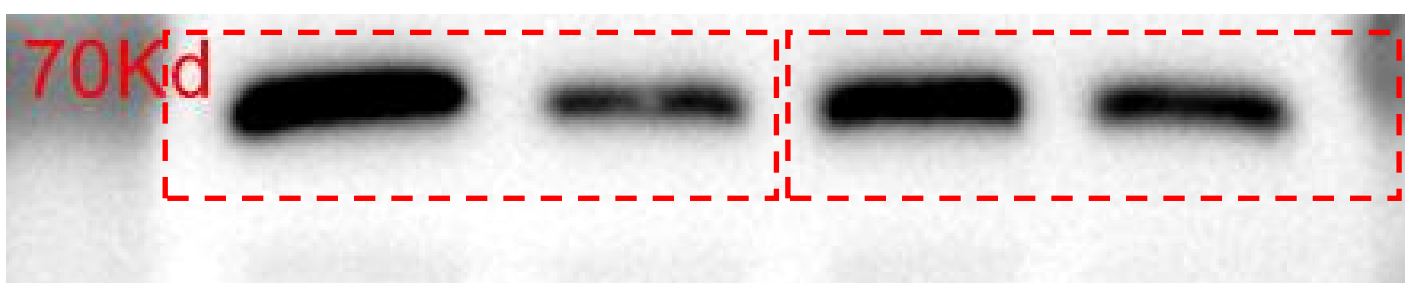

Sample 3 AMPKα

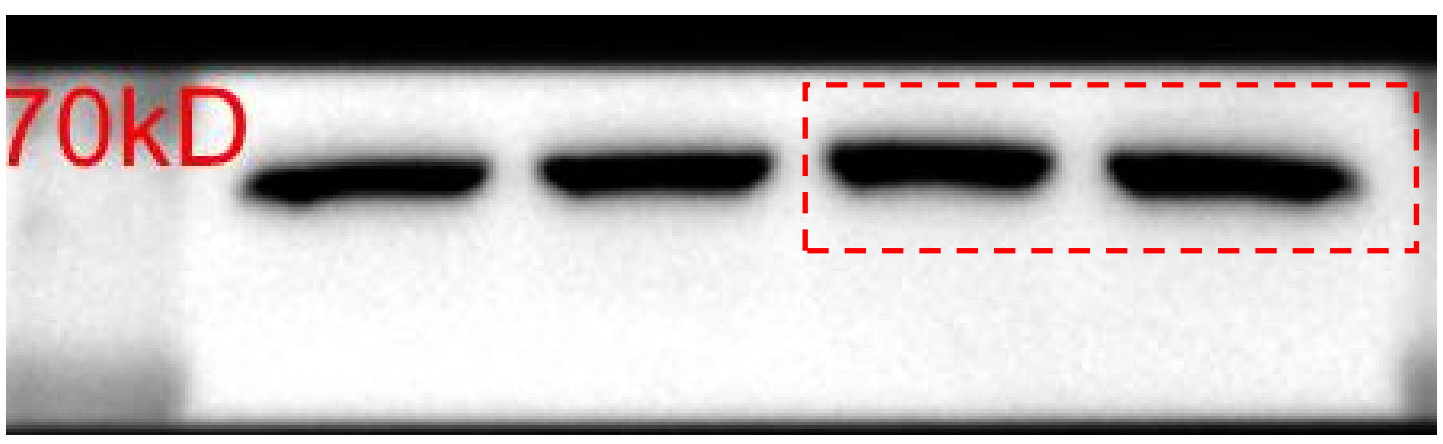

Sample 3 p-AMPKα

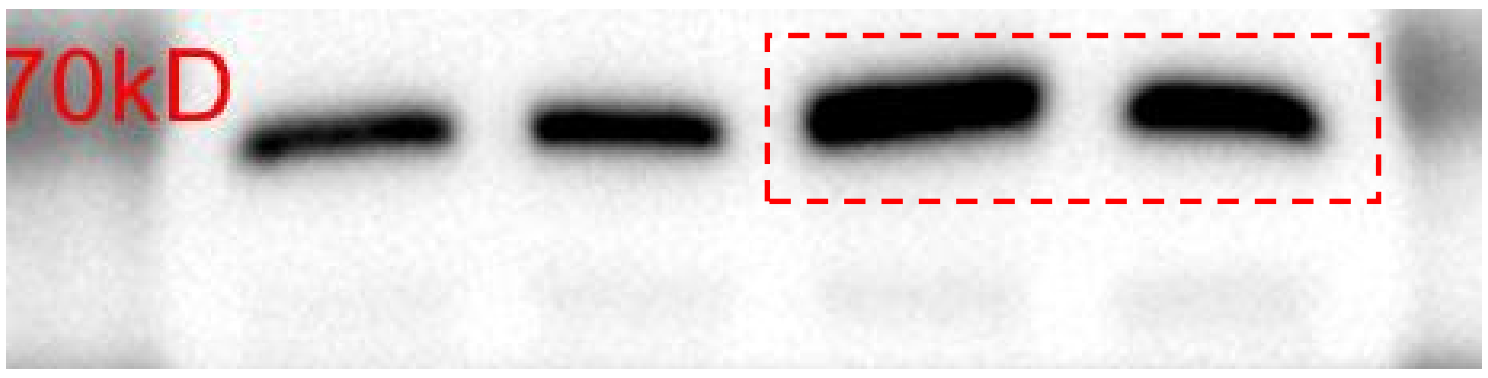

Figure 6 f. Atg5,P62, Lc3II/Lc3I

Atg5

Smaple 1+ Sample 2 Atg5

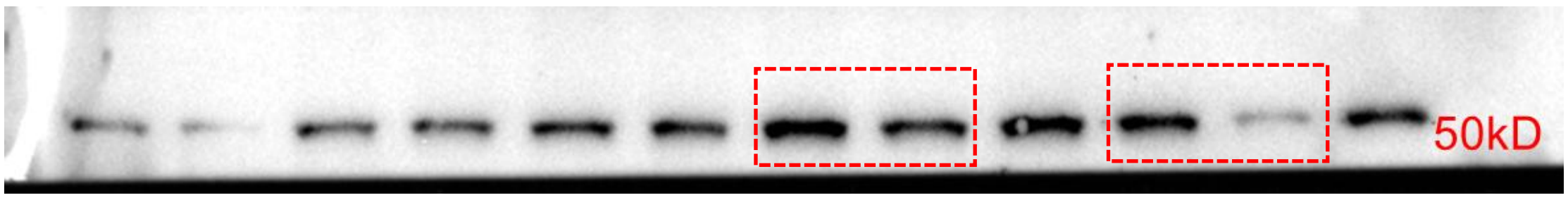

Smaple 1+ Sample 2 Gapdh

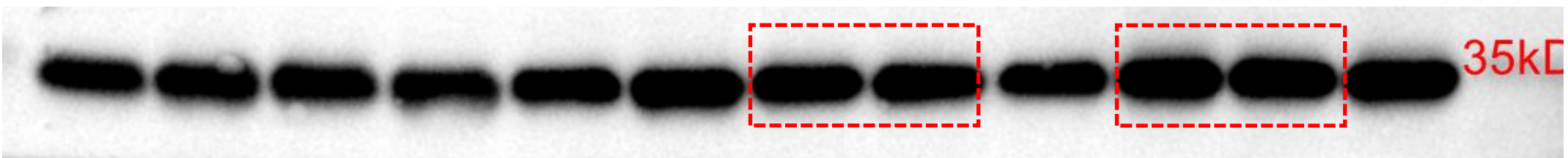

Sample 3 Atg5

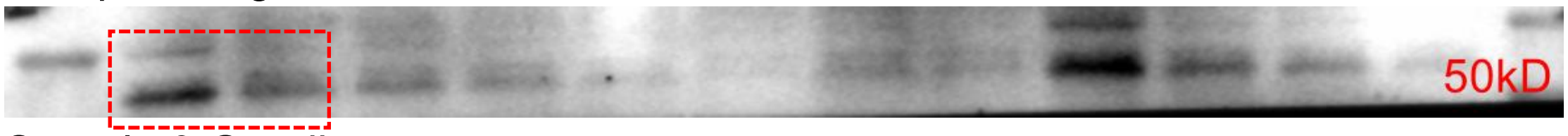

Sample 3 Gapdh

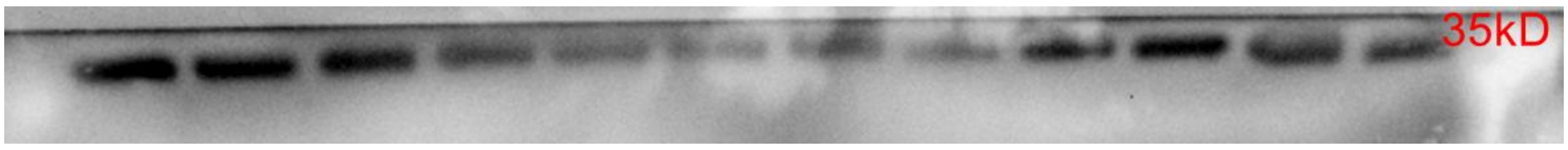

Sample 1+Sample 2+Sample 3 P62

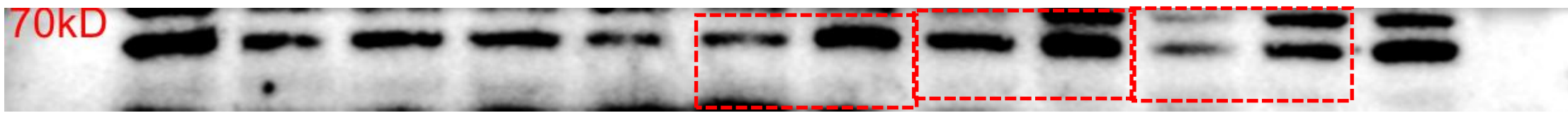

Sample 1+Sample 2+Sample 3 Gapdh

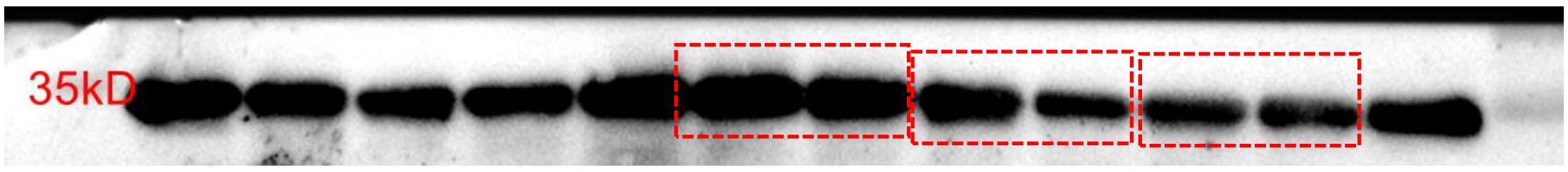

Lc3II/Lc3I

Smaple 1+Sample 2 LC3

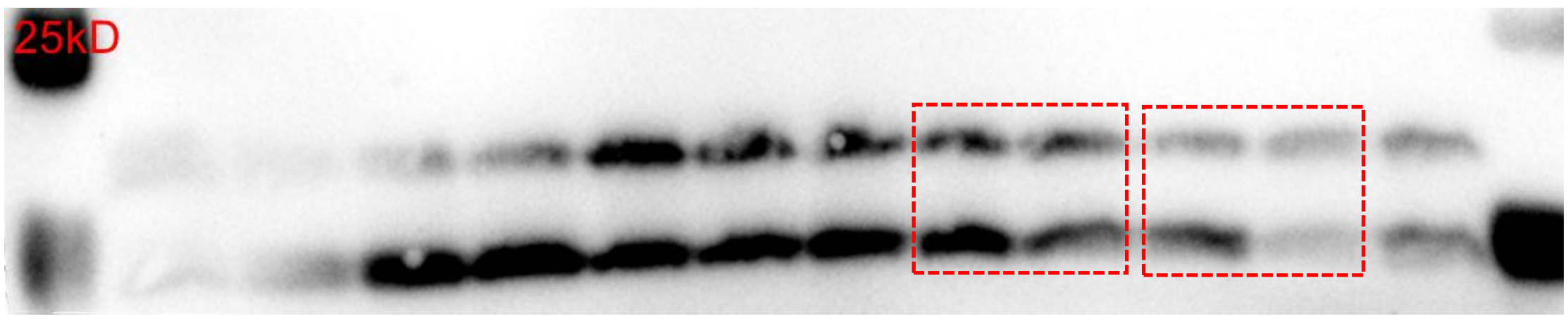

Sample 3 LC3

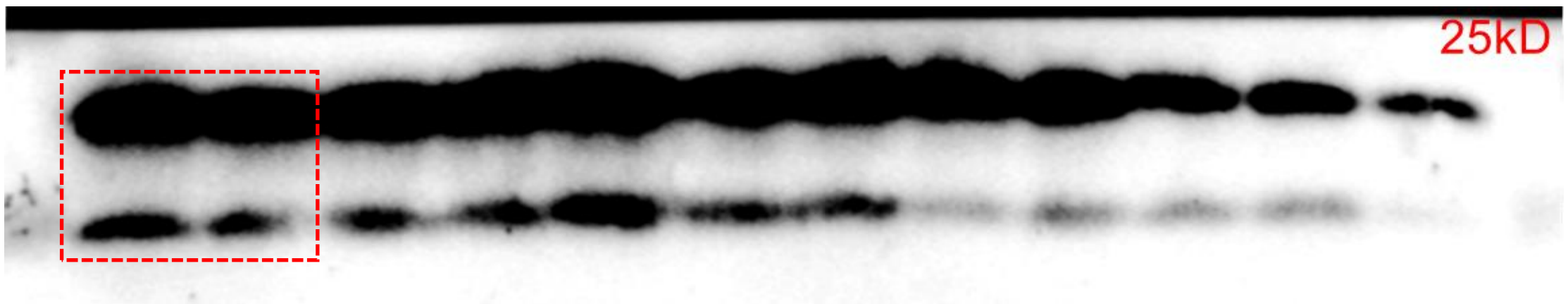

Figure 6 h.

Sample 1+ Sample 2 Ndufb10

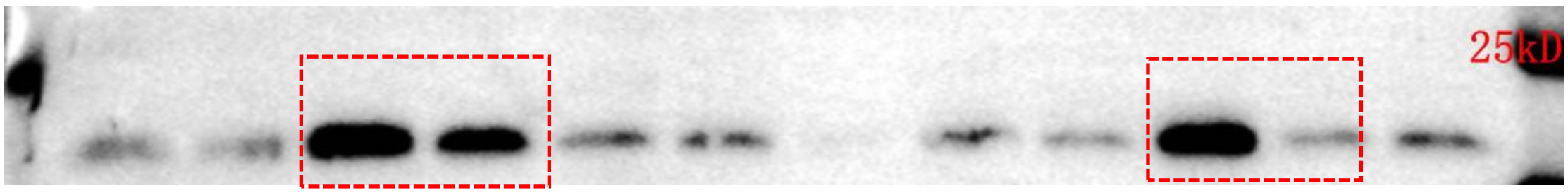

Sample 1+ Sample 2 β Tubulin

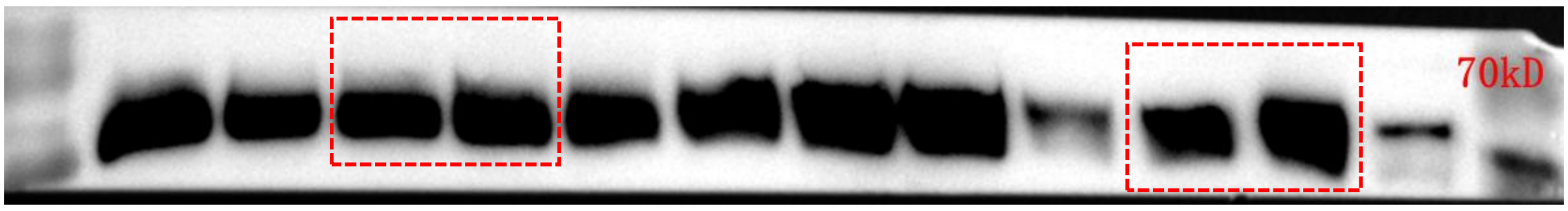

Sample 3 Ndufb10

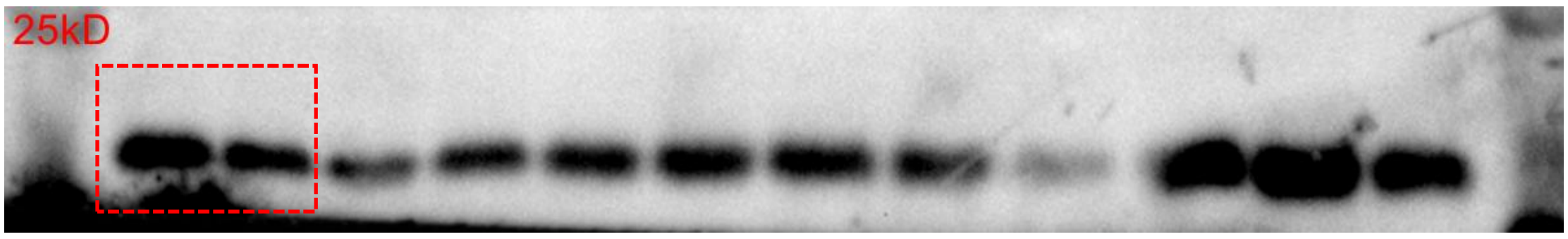

Sample 3 β Tubulin

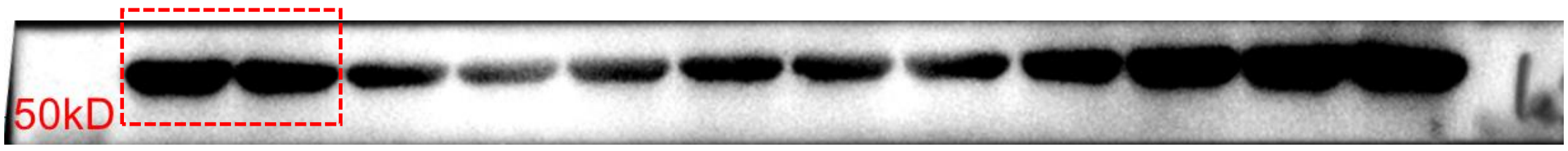

Figure 6.i

Sample 1+ Sample 2+Sample 3 Uqcrc2

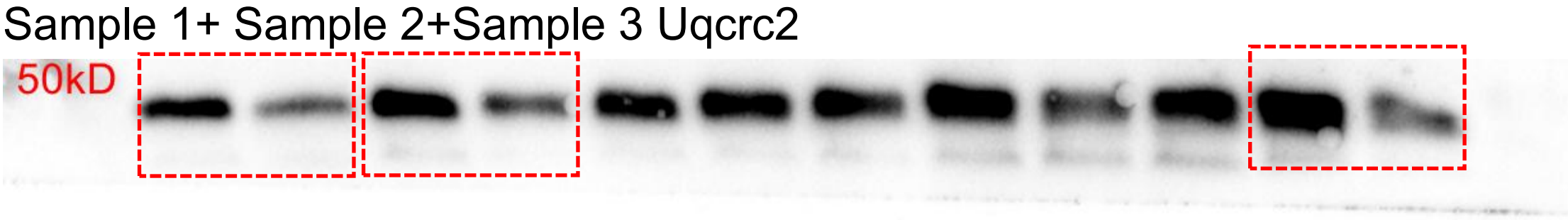

Sample 1+ Sample 2+Sample 3 Gapdh

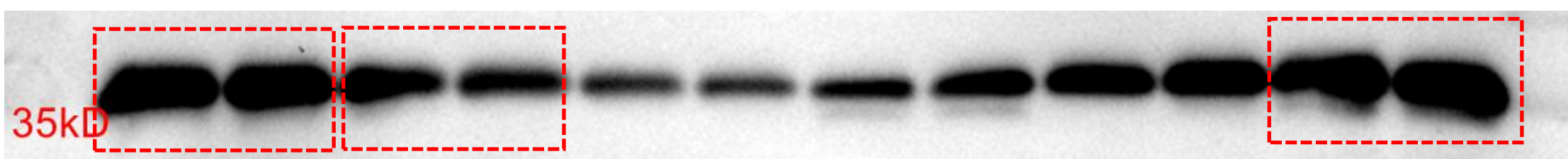

Figure 6.j

Sample 1 Sdhb

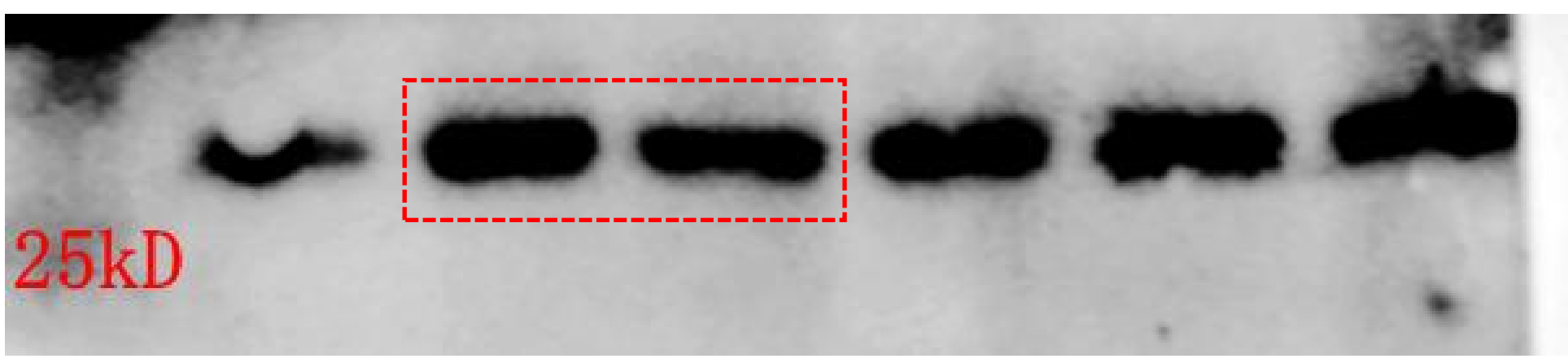

Sample 1 β actin

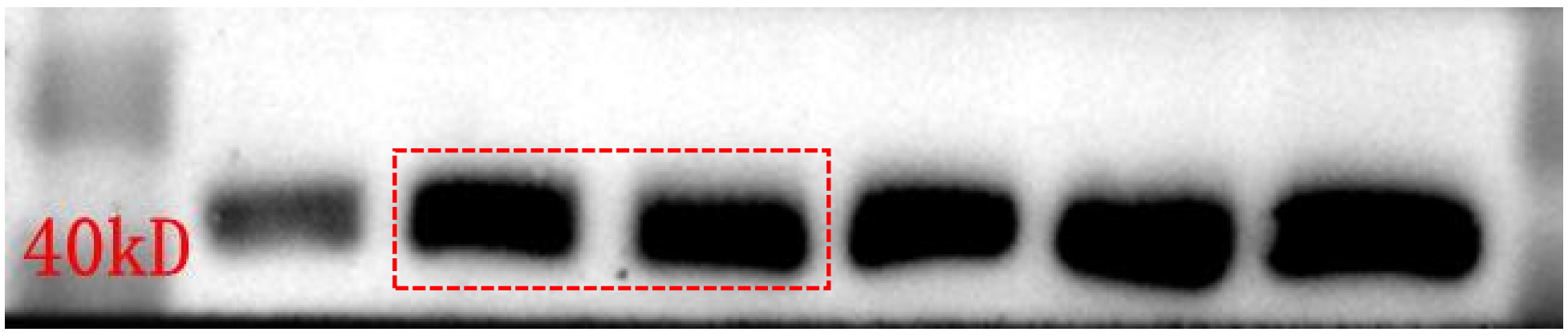

Sample 2+Sample 3 Sdhb

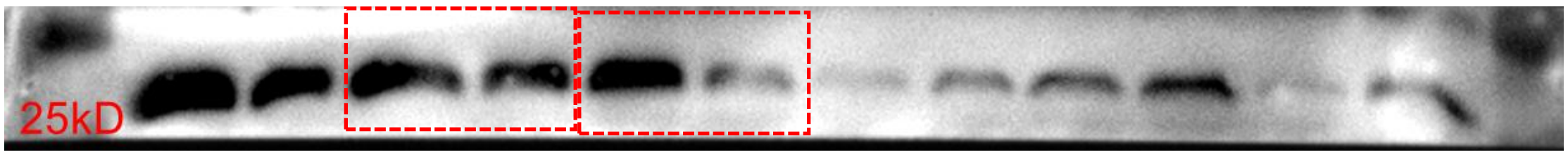

Sample 2+Sample 3 β actin

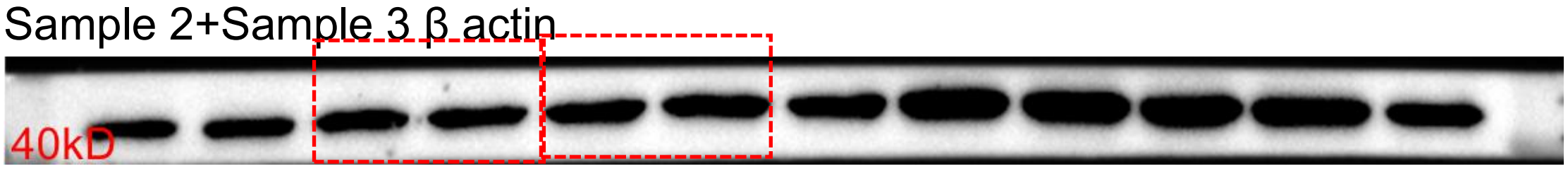

Figure 6.k

Sample 1 MtCO2

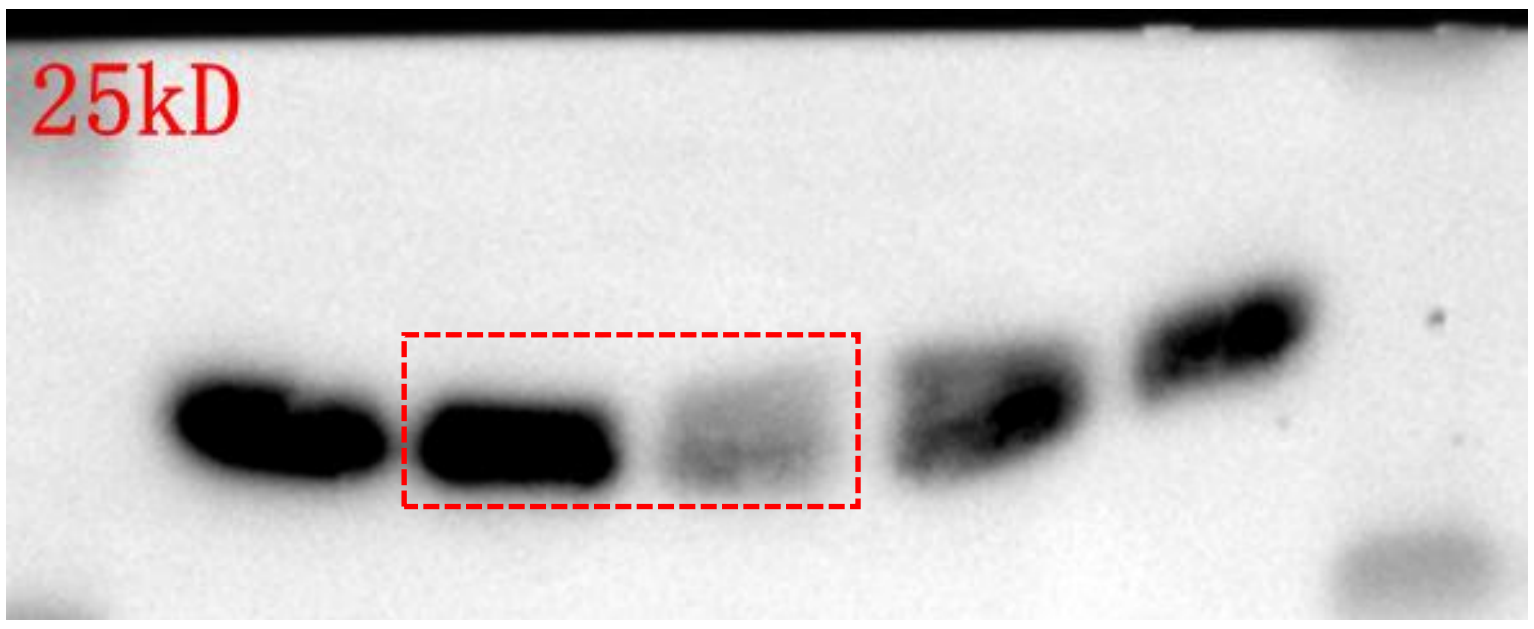

Sample 1 β-Tubulin

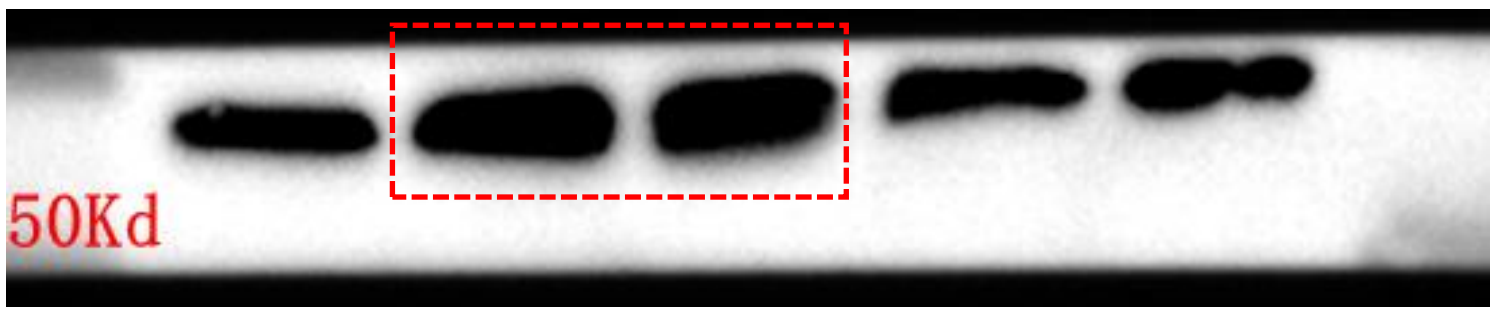

Sample 2+ Sample 2MtCO2

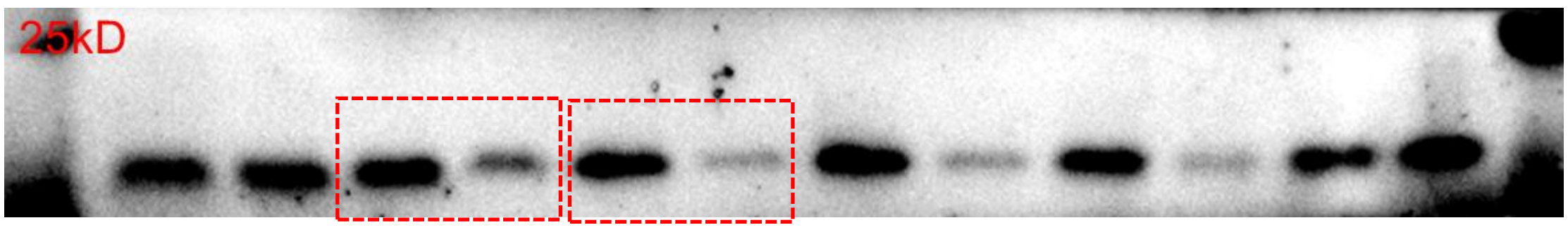

Sample 1+Sample 2 β-Tubulin

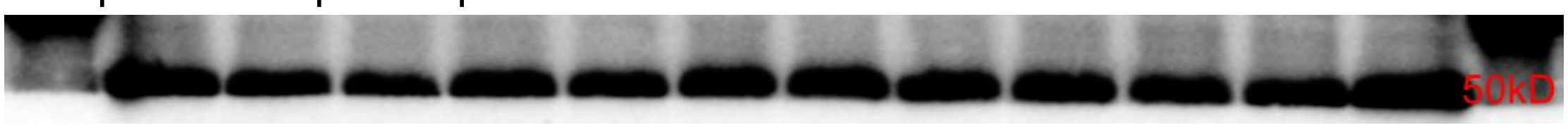

Figure 6.l Atpaf1

Sample 1+Sample 2 Atpaf1

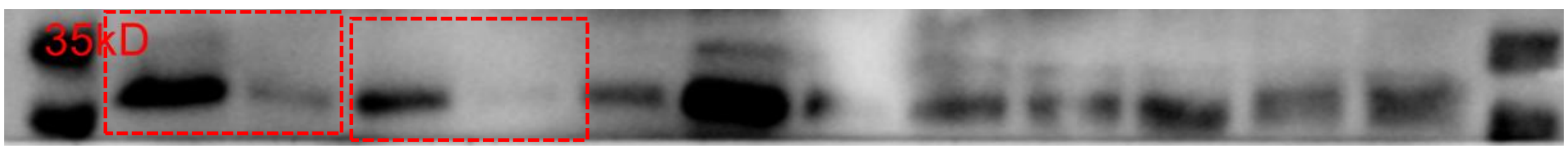

Sample 1+Sample 2 β-Tubulin

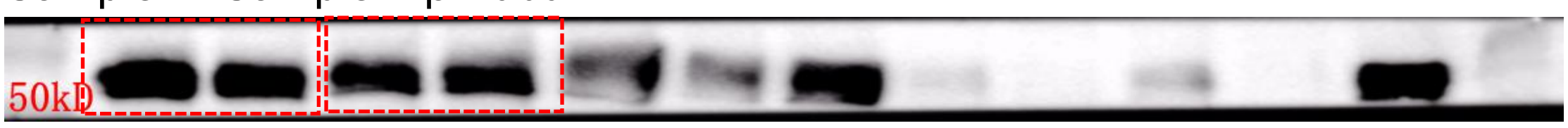

Sample 3 Atpaf1

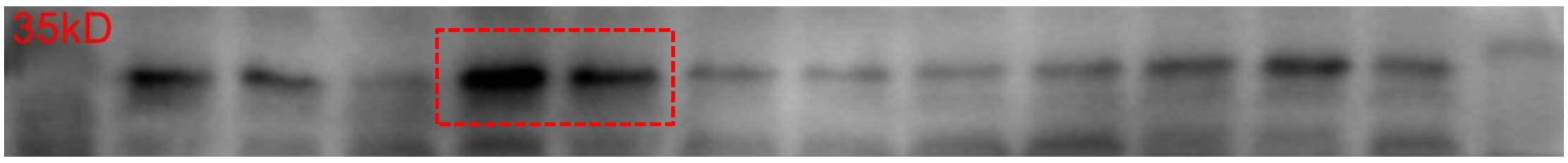

Sample 3 β-Tubulin

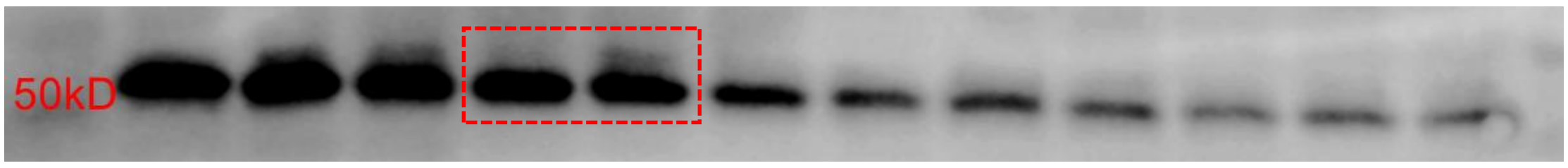

Figure 6m. CPT1A

Sample 1+Sample 2+Sample 3 CPT1A

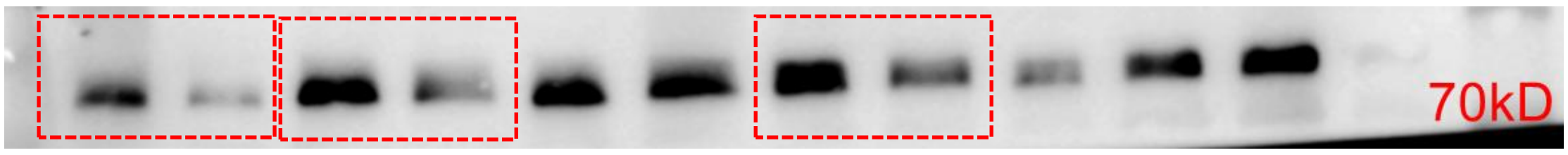

Sample 1+Sample 2+Sample 3 β-Actin

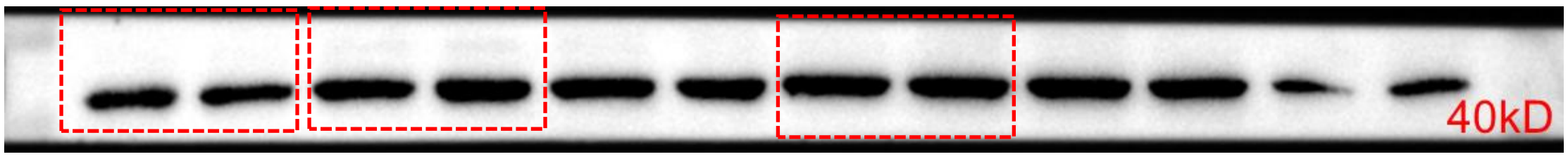

Figure 6.n: Il-10

Sample 1. Il-10

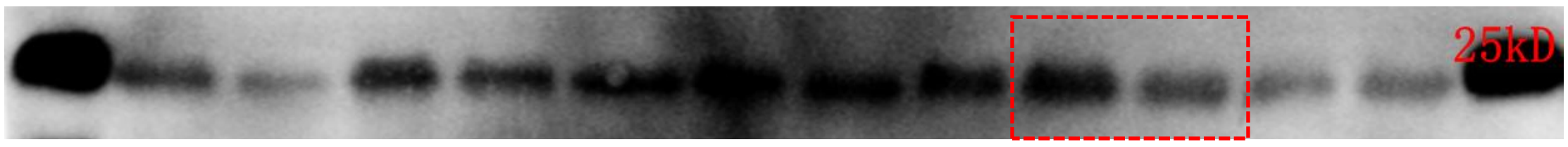

Sample 1. β-Tubulin

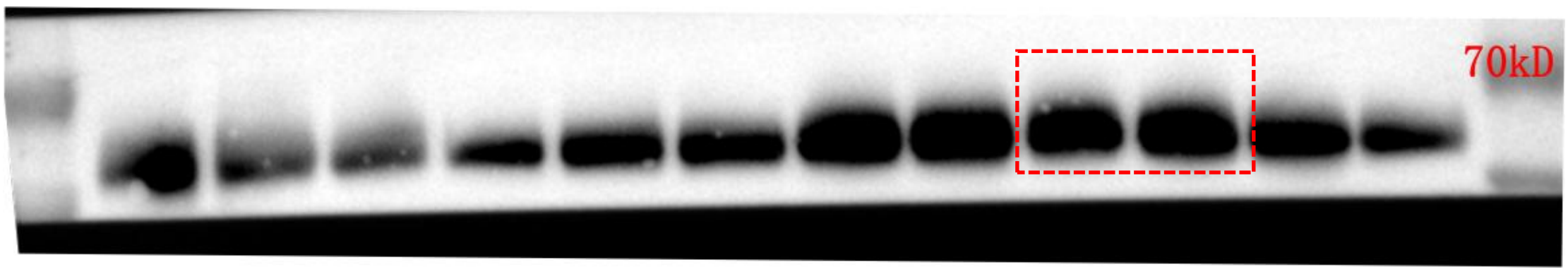

Sample 2+3. IL-10

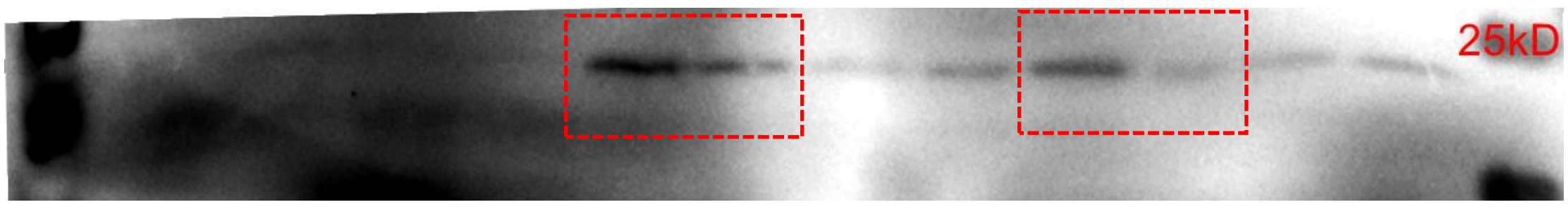

Sample 2+3. β-Tubulin

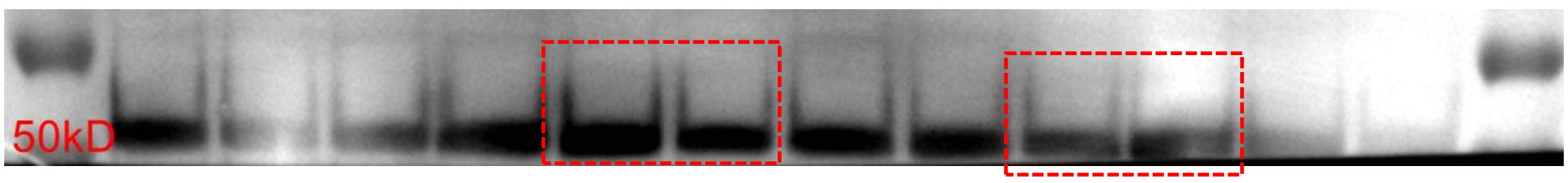

Figure 6.o

Sample 1+Sample 2 Inos

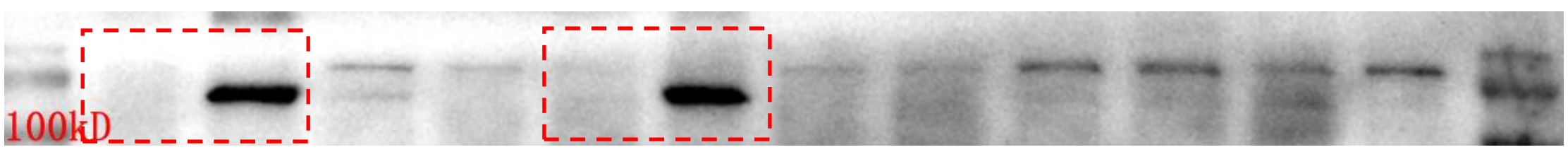

Sample 1+Sample 2 β-Tubulin

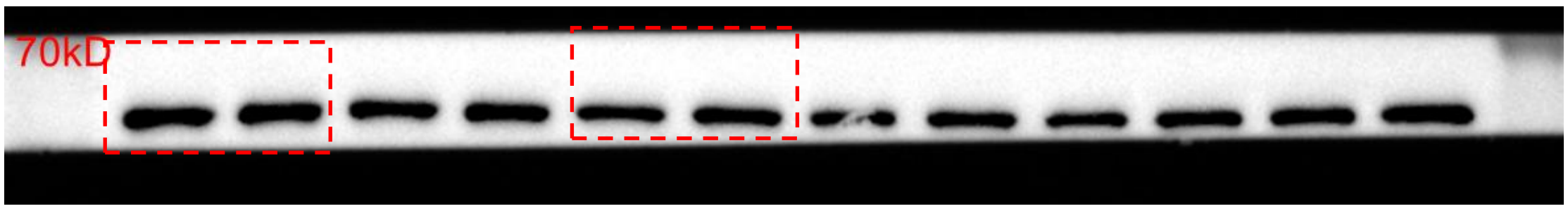

Sample 3 Inos

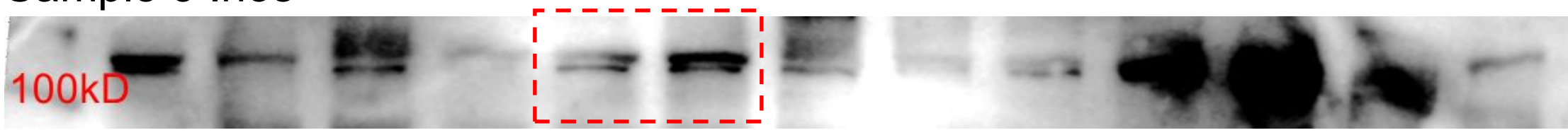

Sample 3 β-Tubulin

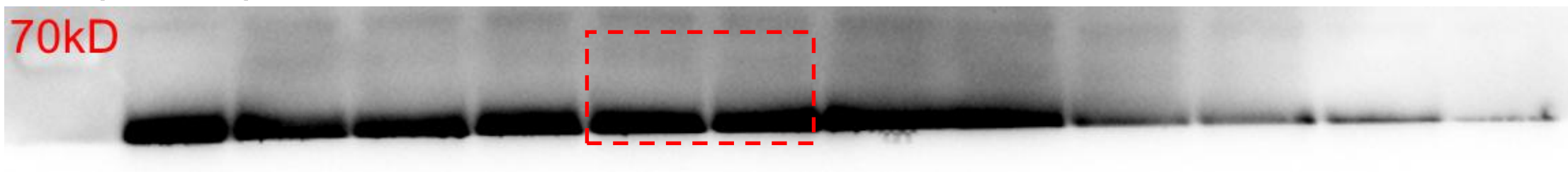

Figure 6.p

Sample 1. Nlrp3

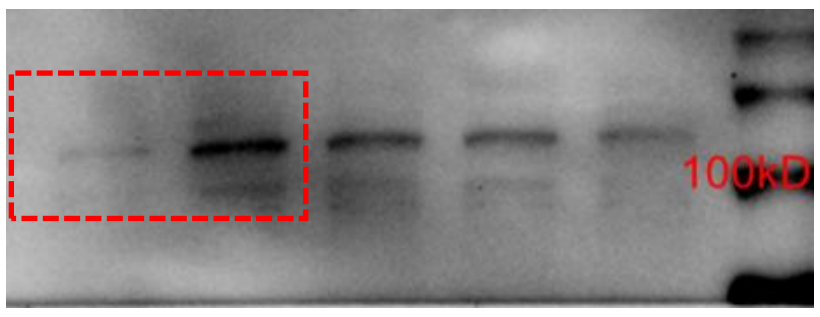

Sample 1. IL-18

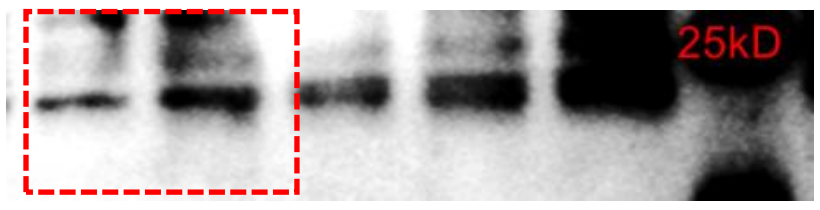

Sample 1. β-actin

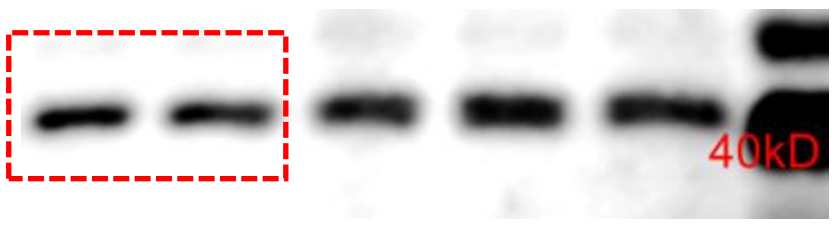

Sample 2+Sample 3. Nlrp3

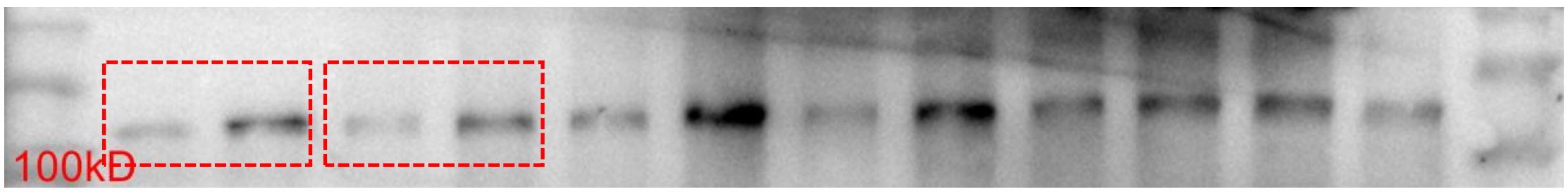

Sample 2+Sample 3. β-actin

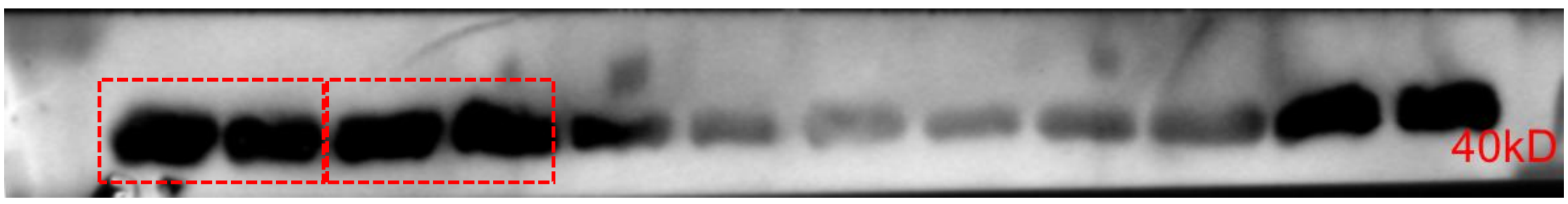

Figure 6.q

Sample 1. Cleaved Il-1β

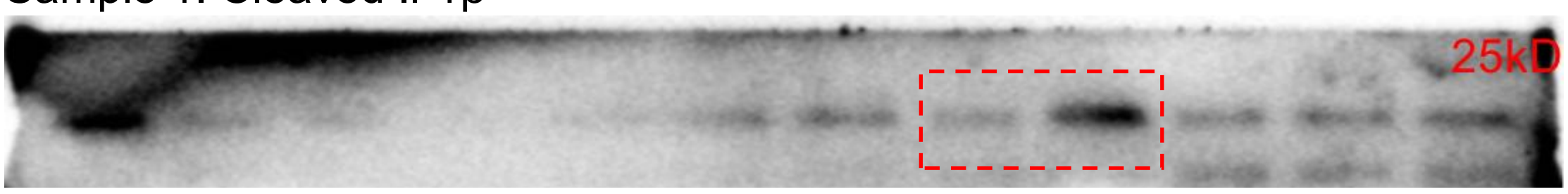

Sample 1. Gapdh

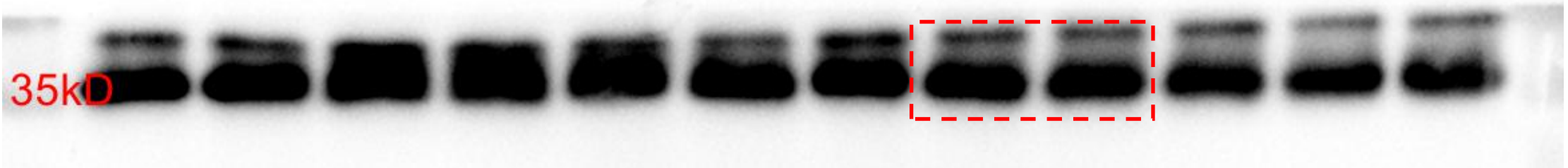

Sample 2+Sample 3. Cleaved Il-1β

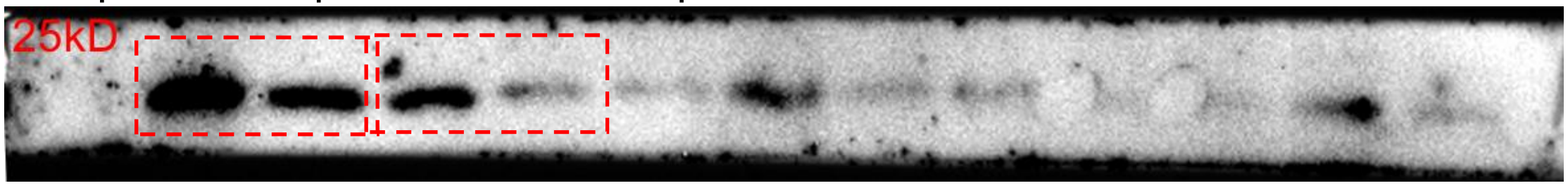

Sample 2+Sample 3. Gapdh

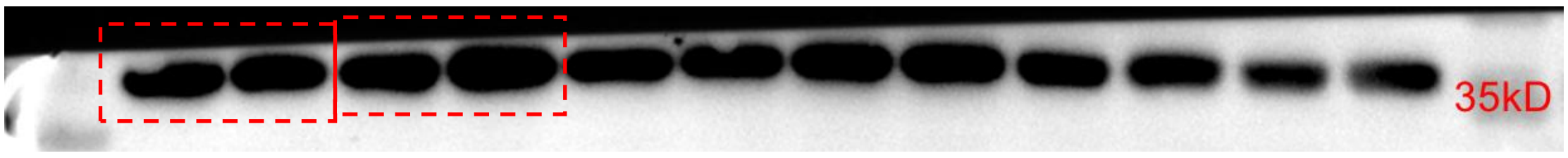

Figure 8. p-Tau,Tau

Lanes within the red dotted boxes, arranged left to right: WT group, Control-5xFAD group, PE-5xFAD group, RNAi-Tmem9-5xFAD group, PE+OE--5xFAD group.

Cortex

Sample 1+Sample 2 p-Tau 217 cortex

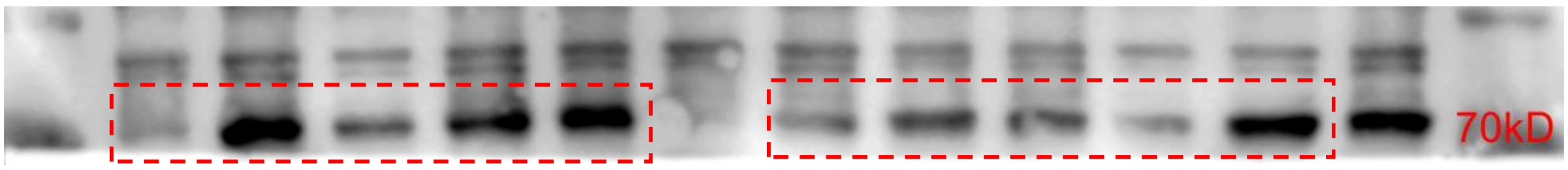

Sample 1+Sample 2 Tau cortex

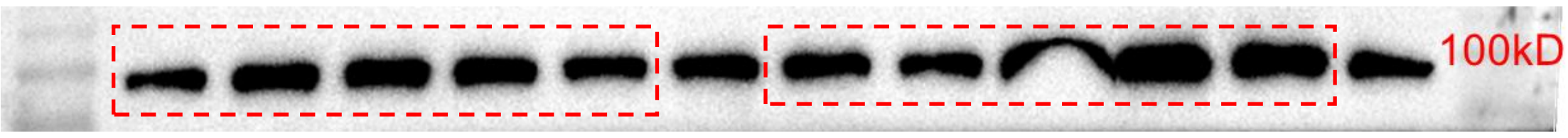

Sample 3 p-Tau 217 cortex

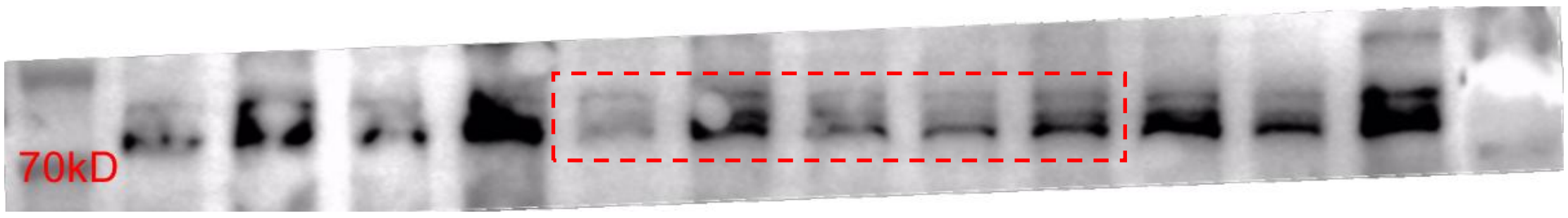

Sample 3 Tau cortex

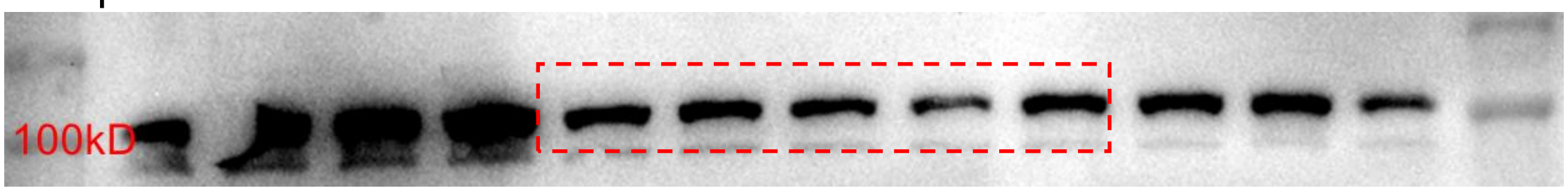

Sample 4 p-Tau 217 cortex

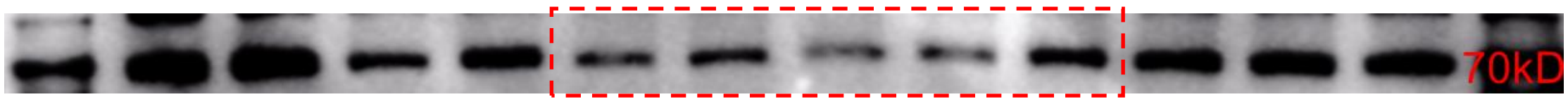

Sample 4 Tau cortex

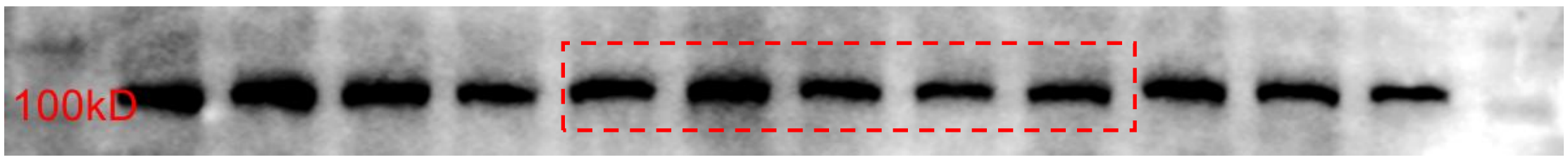

Hippocampus

Sample 1 p-Tau 217 Hippo

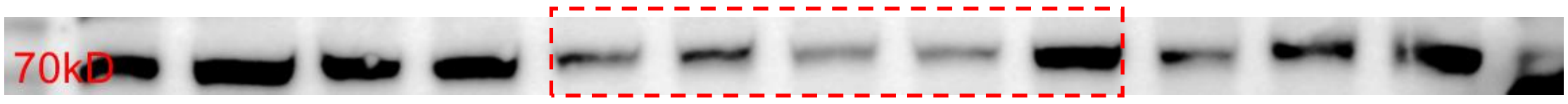

Sample 1 Tau Hippo

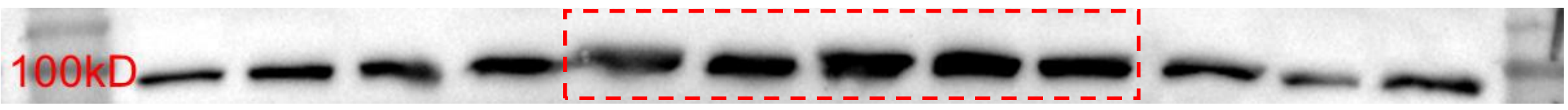

Sample 2 p-Tau 217 Hippo

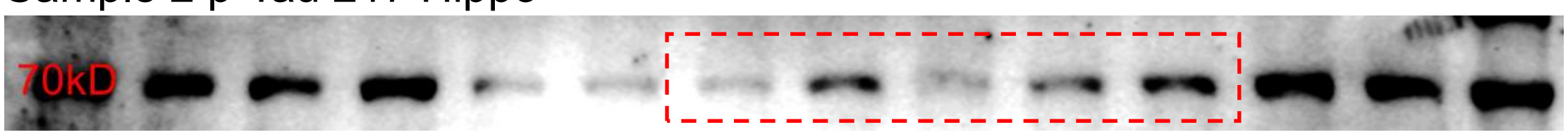

Sample 2 Tau Hippo

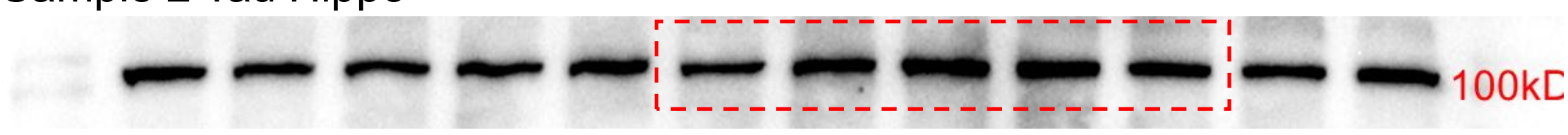

Sample 3 p-Tau 217 Hippo

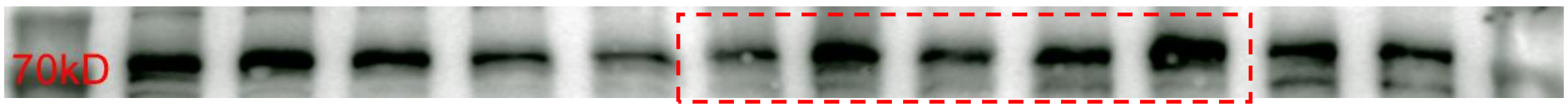

Sample 3 Tau Hippo

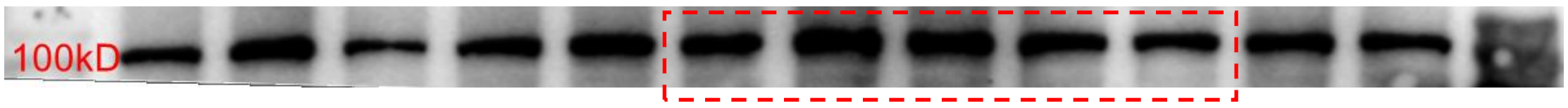

Sample 4 p-Tau 217 Hippo

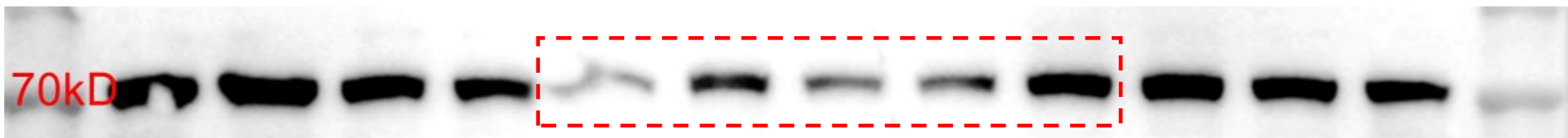

Sample 4 Tau Hippo

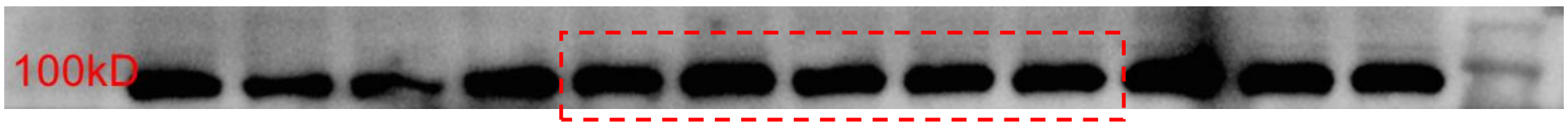

Supplementary figure 1d.

Lanes within the red dotted boxes, arranged left to right: WT group, Control-5xFAD group, PE-5xFAD group.

Atgl

Sample 1 Atgl cortex+Hippo

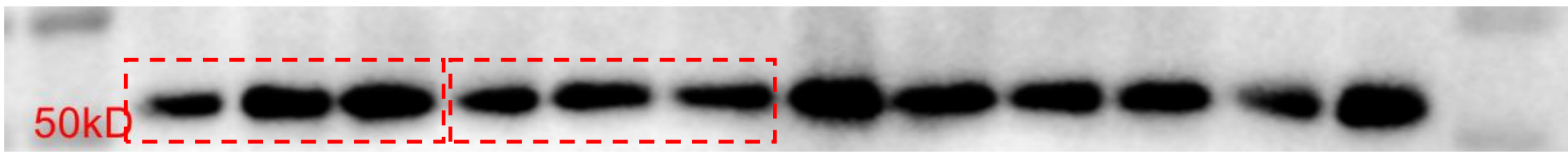

Sample 1 Gapdh cortex+Hippo

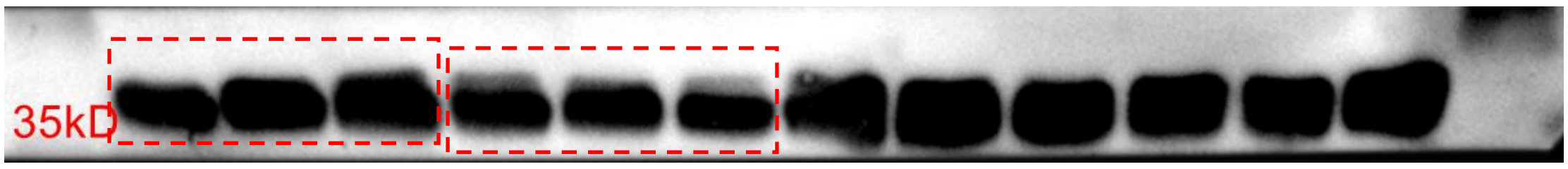

Sample 2+sample 3 Atgl cortex+Hippo

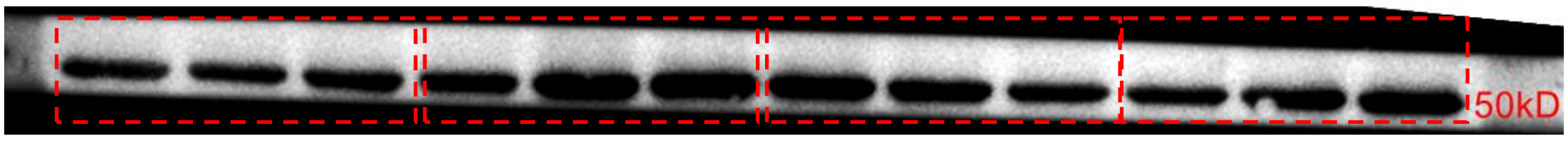

Sample 2+sample 3 Gapdh cortex+Hippo

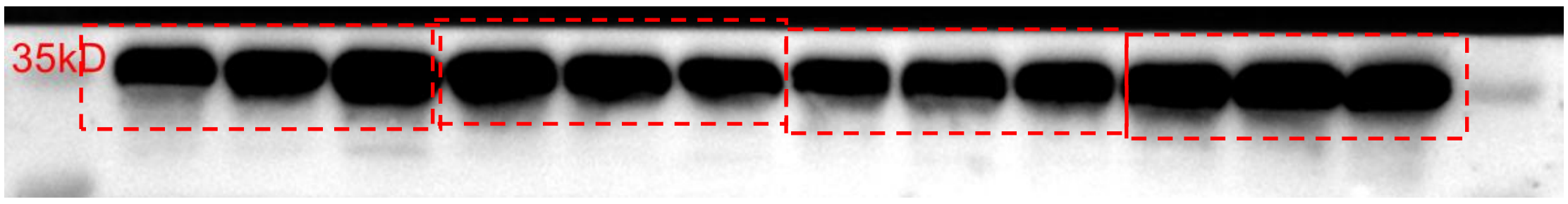

Hsl

Sample 1 Hsl Cortex+Hippo

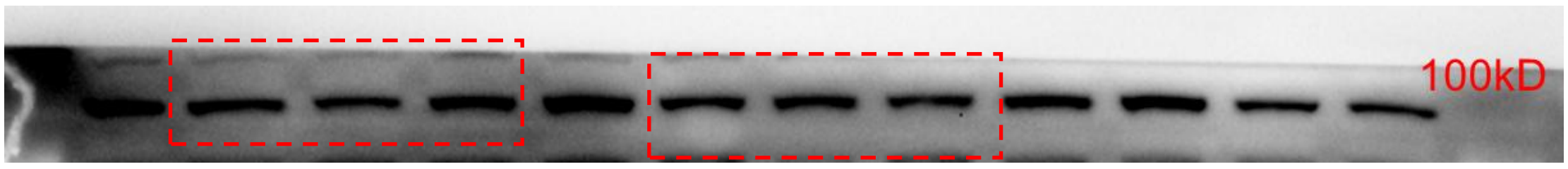

Sample 1 β-actin Cortex+Hippo

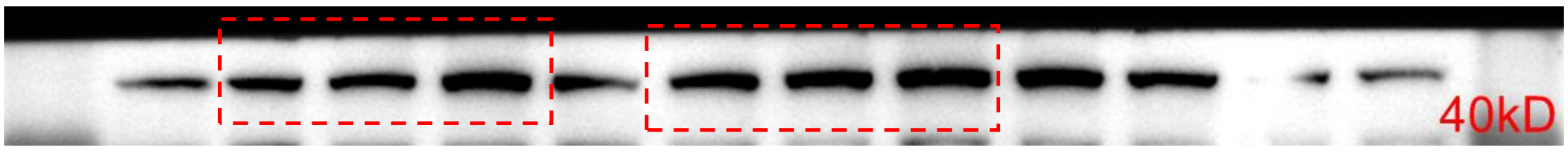

Sample 2 Hsl Cortex+Hippo

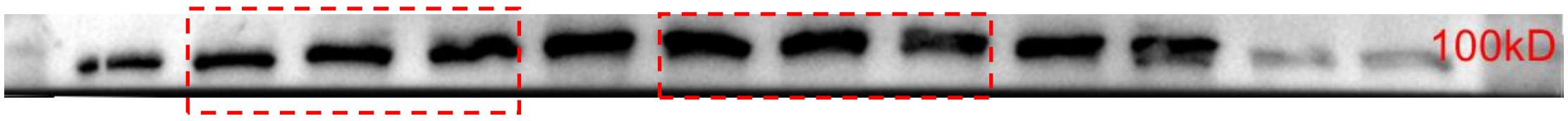

Sample 2 β-actin Cortex+Hippo

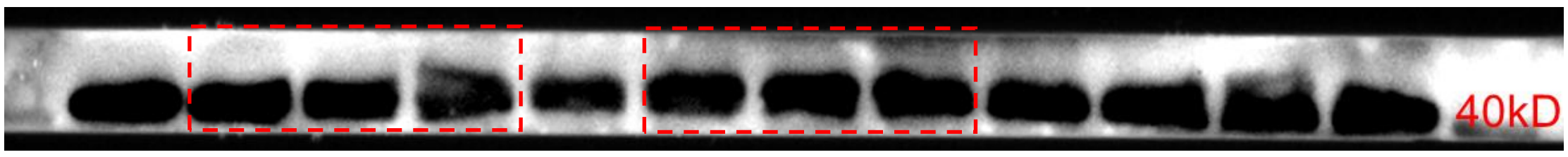

Sample 3 Hsl Cortex+Hippo

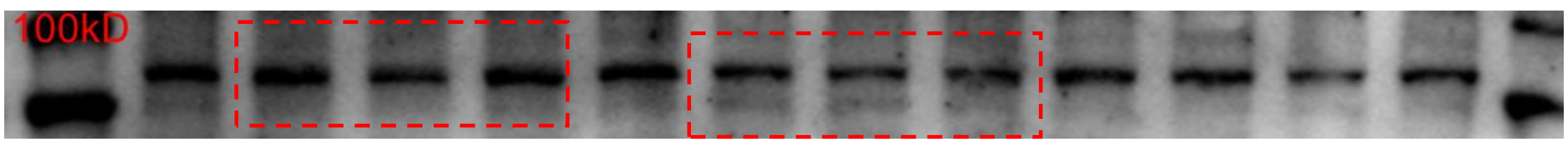

Sample 3 β-actin Cortex+Hippo

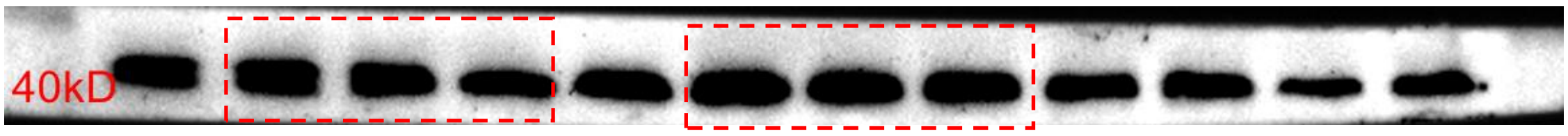
40kD

Supplementary Figure 2b. Lanes within the red dotted boxes, arranged left to right: WT, Control-5xFAD, PE-5xFAD, RNAi-Tmem9, PE+OE-Tmem9.

Cortex

Sample 1 Tmem9-Cortex

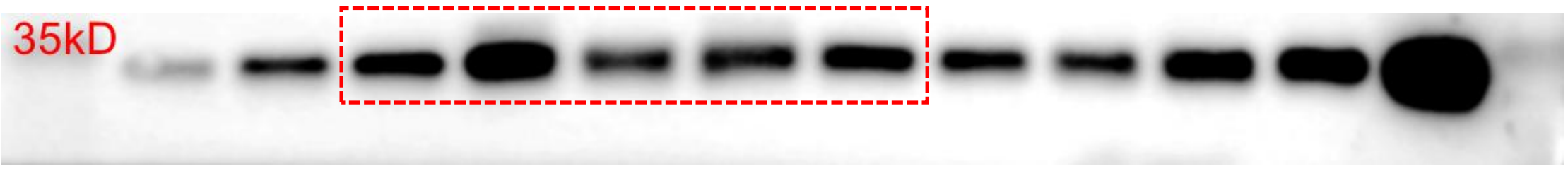

Sample 1 β-Tubulin-Cortex

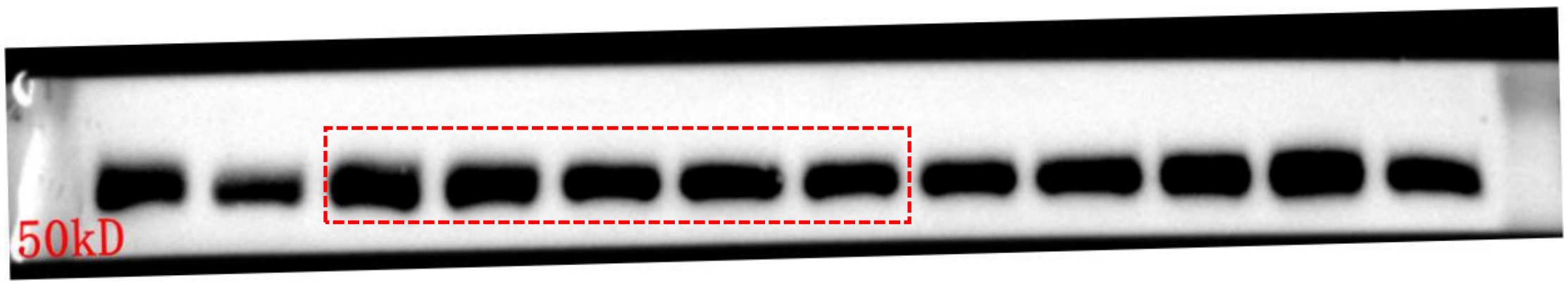

Sample 2 Tmem9-Cortex

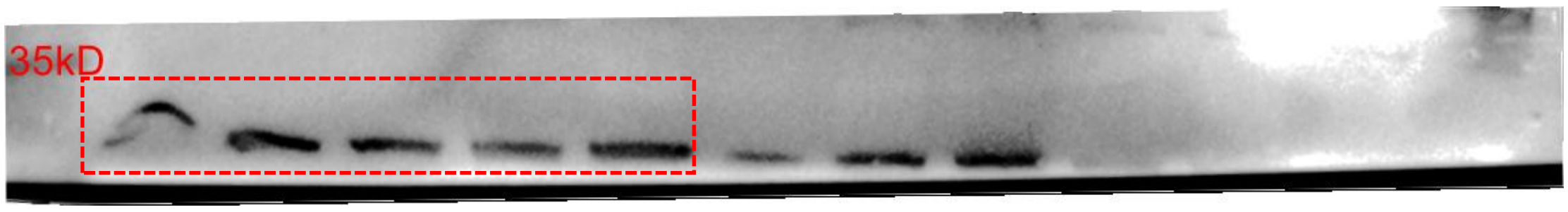

Sample 2 β-Tubulin-Cortex

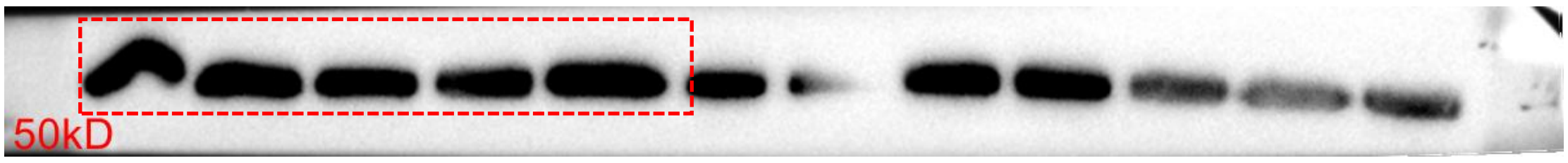

Sample 3 Tmem9-Cortex

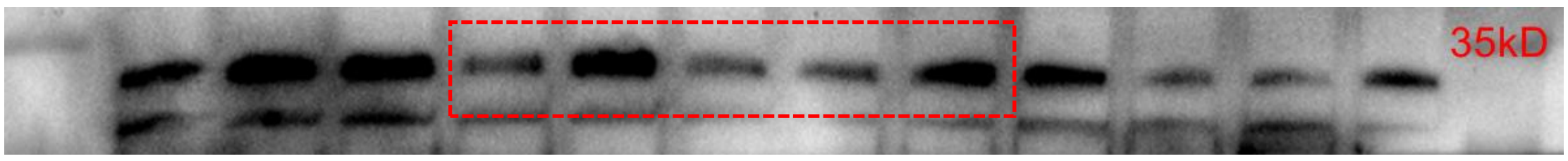

Sample 3 β-Tubulin-Cortex

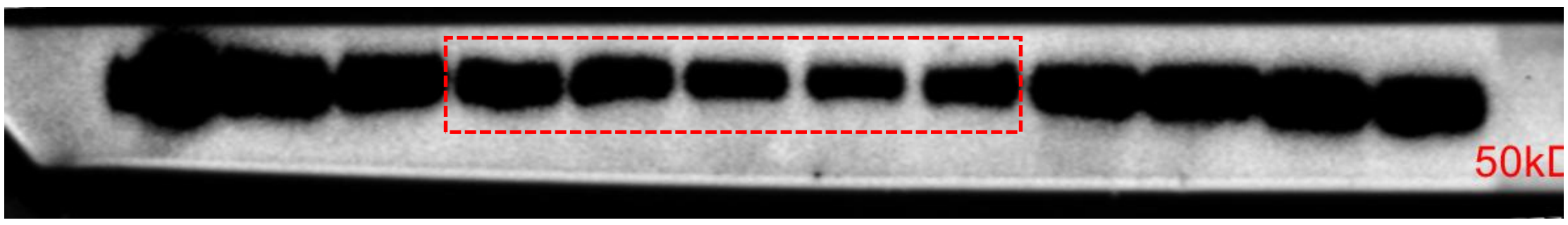

Sample 4 Tmem9-Cortex

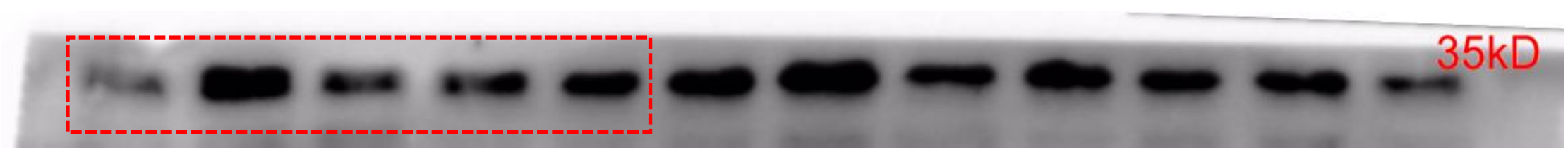

Sample 4 β-Tubulin-Cortex

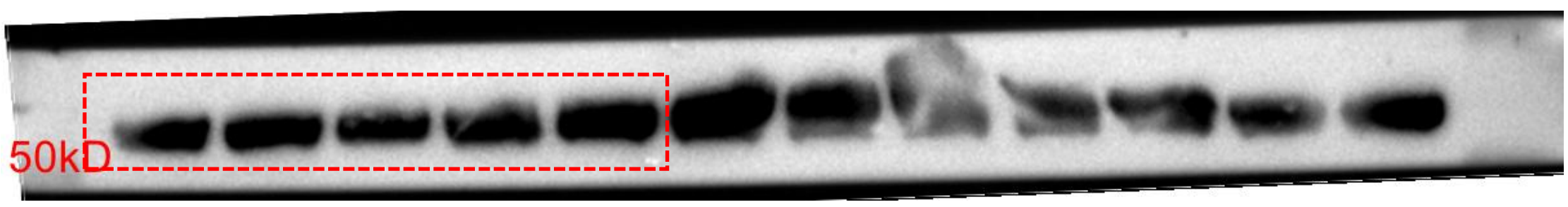

Hippocampus

Sample 1 Tmem9-Hippo

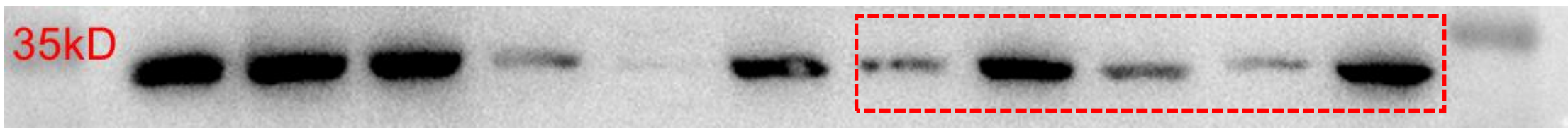

Sample 1 β-Tubulin-Hippo

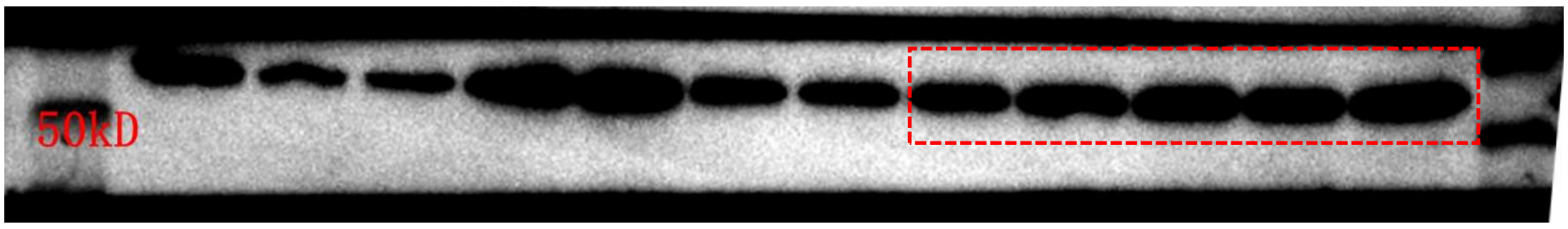

Sample 2 Tmem9-Hippo

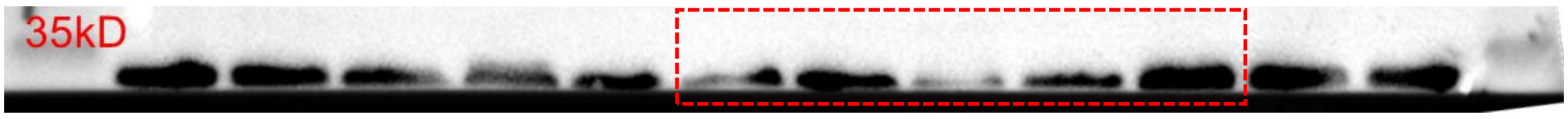

Sample 2 β-Tubulin-Hippo

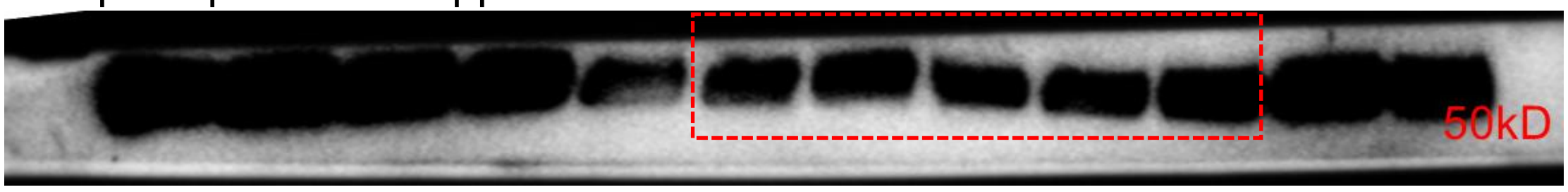

Sample 3+Sample 4 Tmem9-Hippo

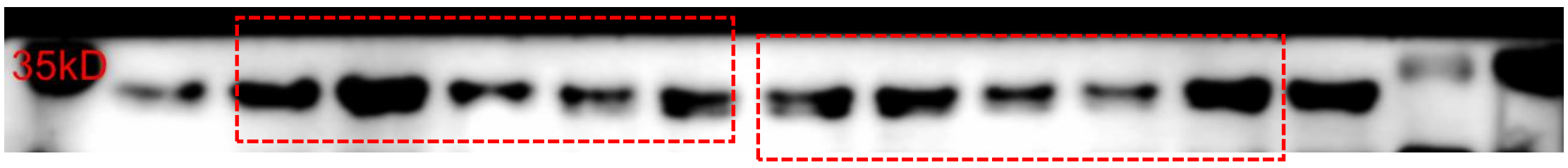

Sample 3+Sample 4 β-Tubulin-Hippo

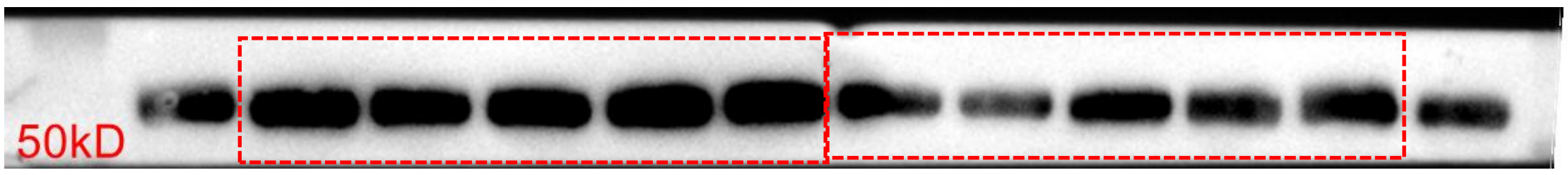

Supplementary Figure 4b. Lanes within the red dotted boxes, arranged left to right: BV2 cells transfected wit scrambe, ShRNA-Tmem9, negative control (NC) and OE-Tmem9 lentivirus.

Sample 1. Tmem9

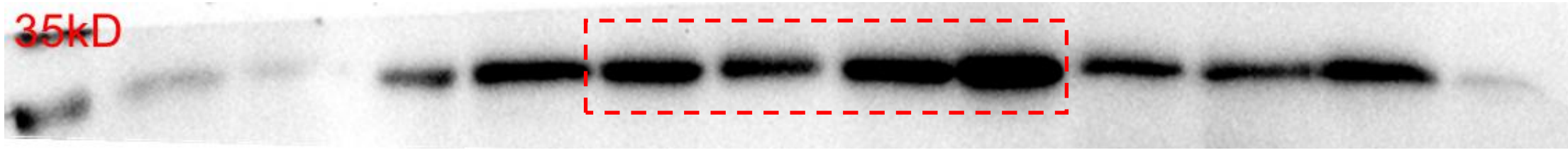

Sample 1. β-Tubulin

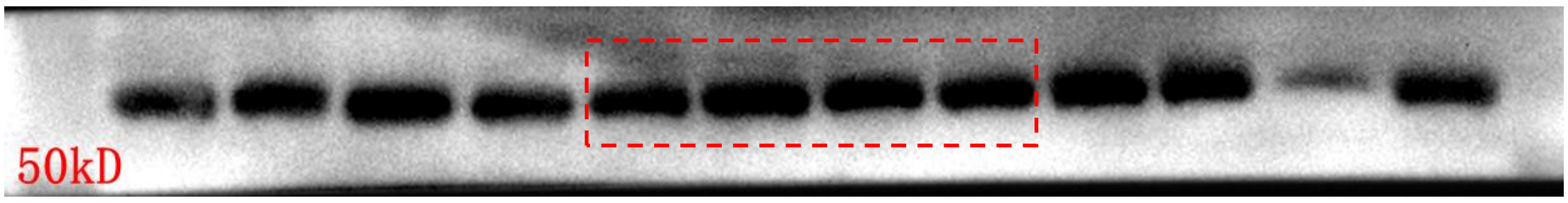

Sample 2. Tmem9

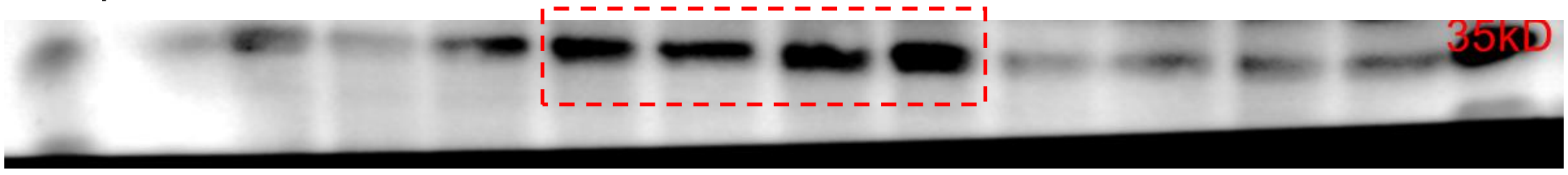

Sample 2. β-Tubulin

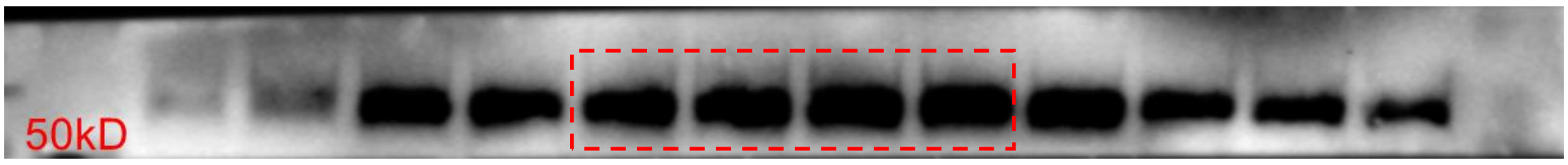

Sample 3. Tmem9

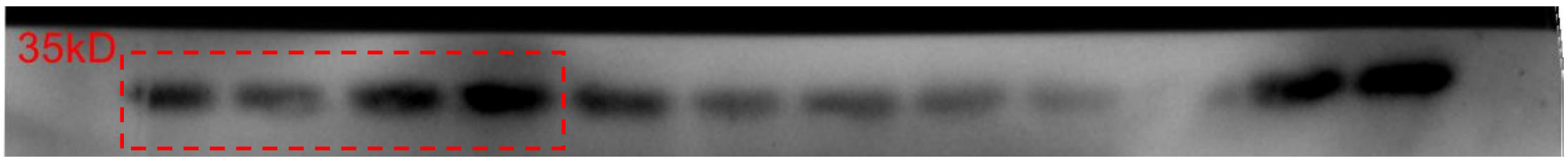

Sample 3. β-Tubulin

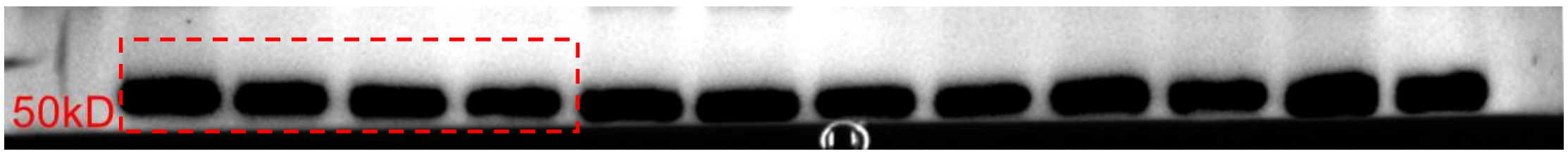

Supplementary figure 5.

Lanes within the red dotted boxes, arranged left to right: Scramble, shRNA-Tmem9, Negative Control (NC), and Overexpression (OE)-Tmem9.

b.Atg5, P62, Lc3II/Lc3I

Atg5

Sample -1 Atg5

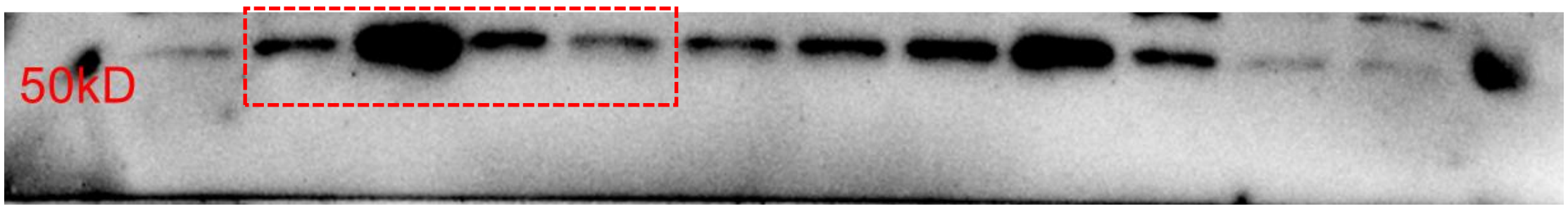

Sample -1 Gapdh

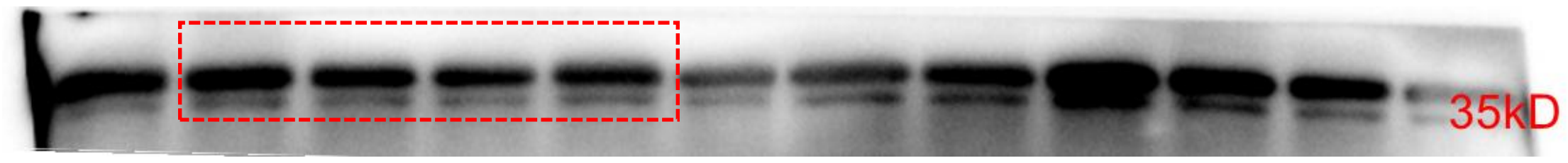

Sample -2+Sample 3 Atg5

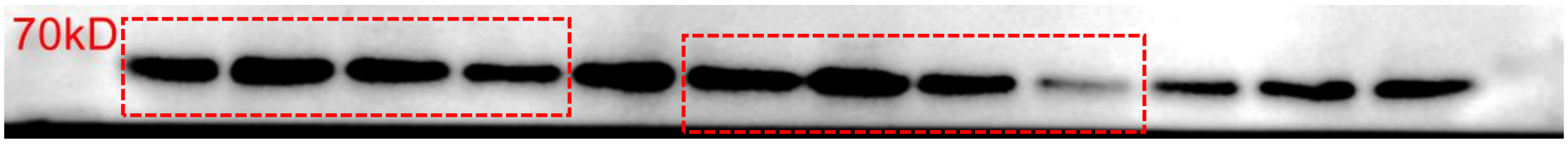

Sample -2+Sample 3 Gapdh

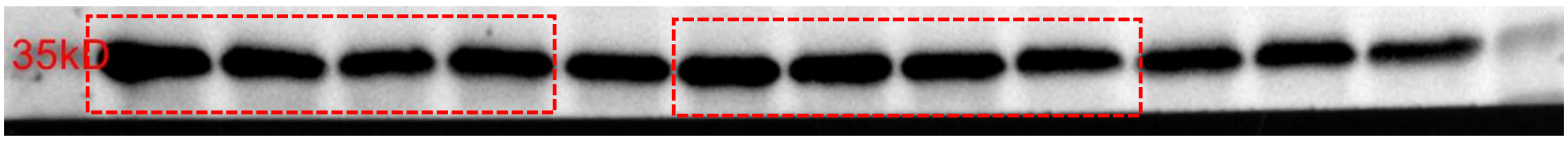

P62

Sample-1. P62

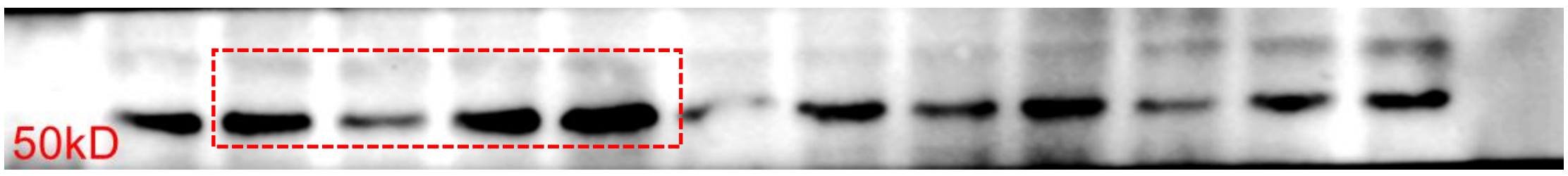

Sample-1. Gapdh

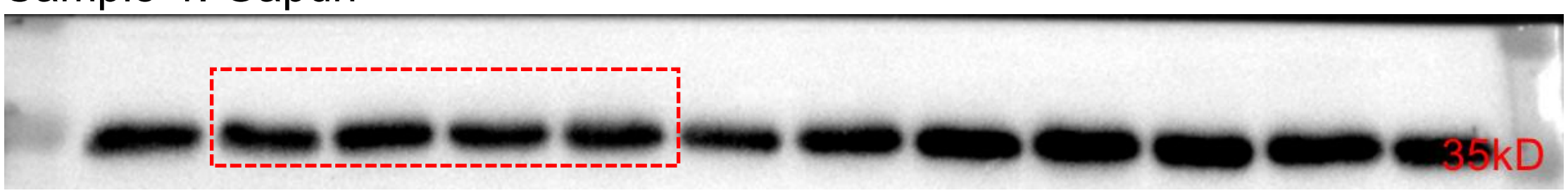

Sample-2. P62

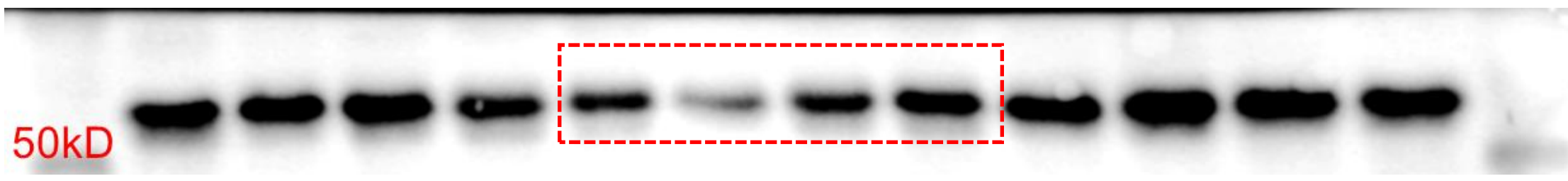

Sample-2. Gapdh

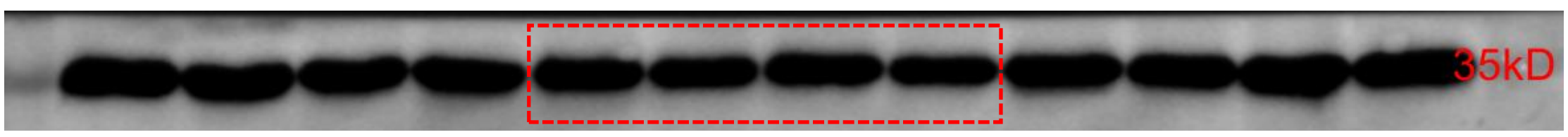

Sample-3. P62

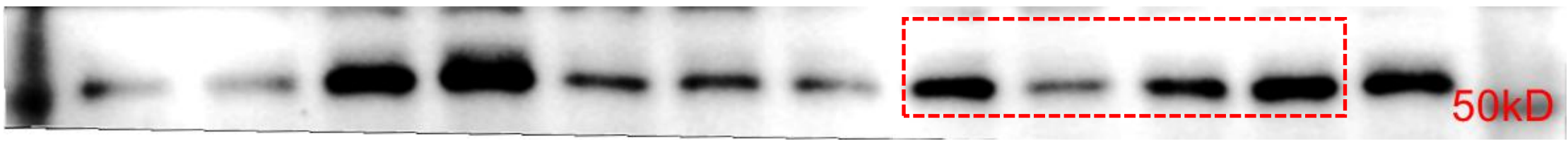

Sample-3. Gapdh

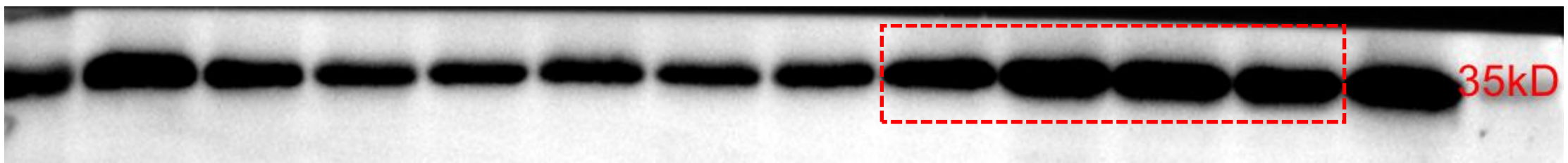

Lc3II/Lc3I

Sample 1. Lc3

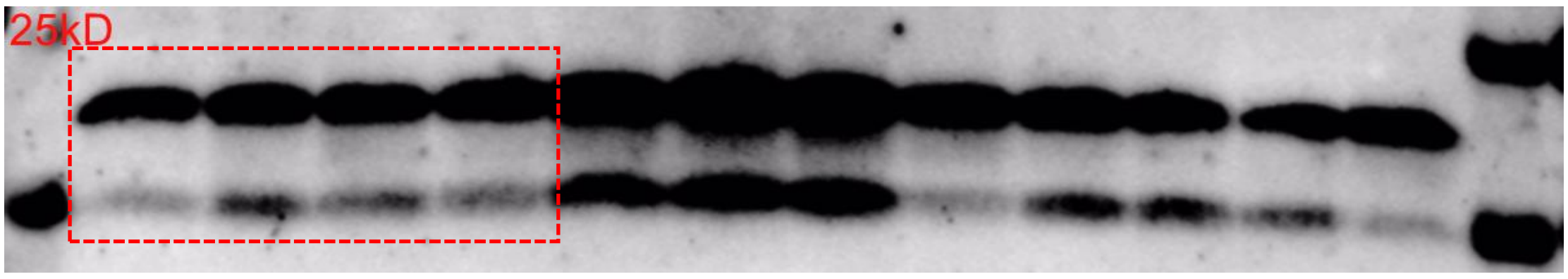

Sample 2. Lc3

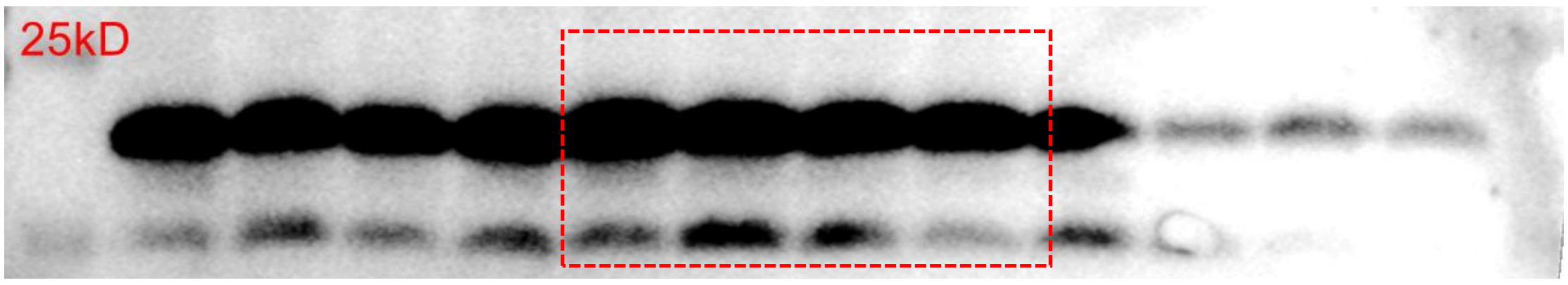

Sample 3. Lc3

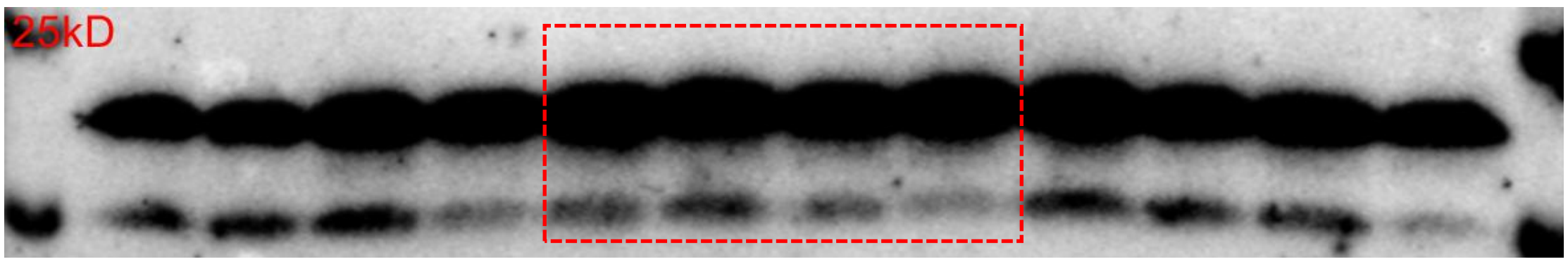

e. Ndufb10, Sdhb, Uqcrc2, Mtco2, Atpaf1

Ndufb10

Sample 1. Ndufb10

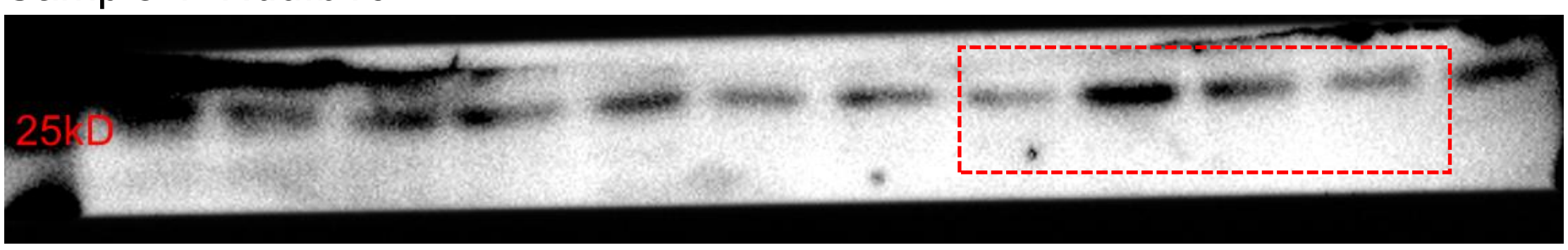

Sample 1. Gapdh

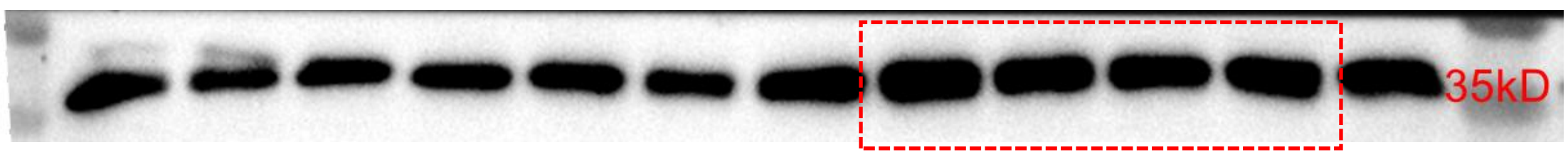

Sample 2. Ndufb10

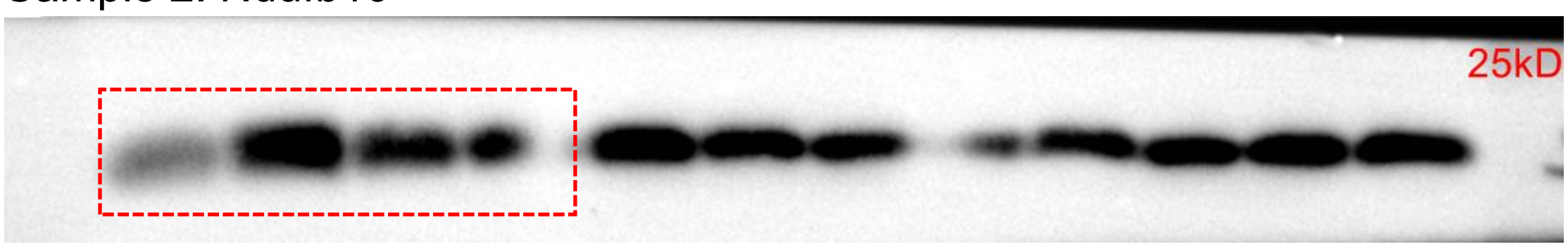

Sample 2. Gapdh

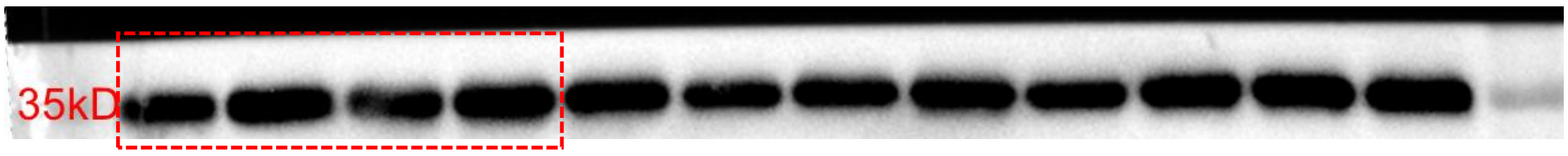

Sample 3. Ndufb10

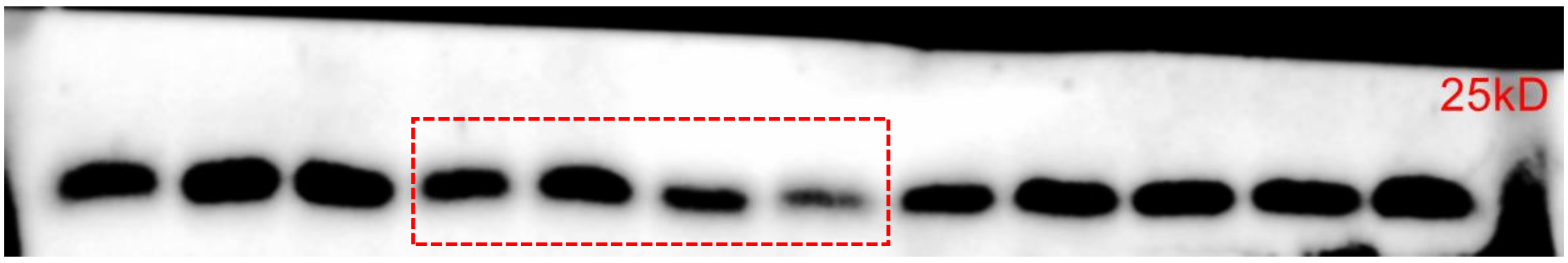

Sample 3. Gapdh

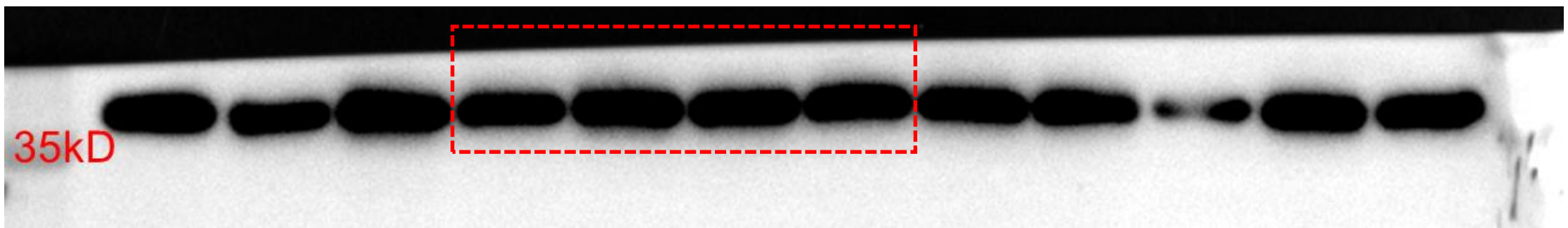

Shdb

Sample 1. Sdhb

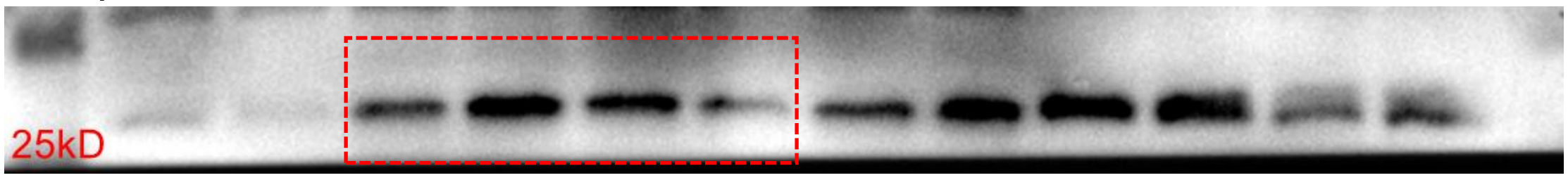

Sample 1. β-Tubulin

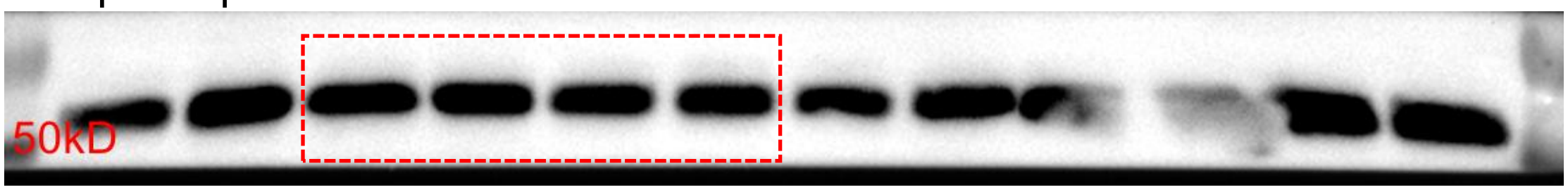

Sample 2. Sdhb

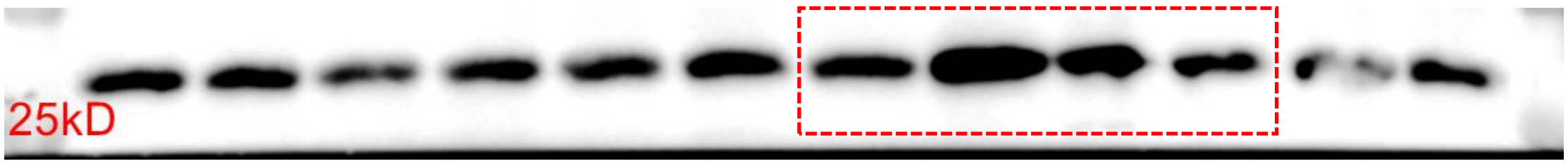

Sample 2. β-Tubulin

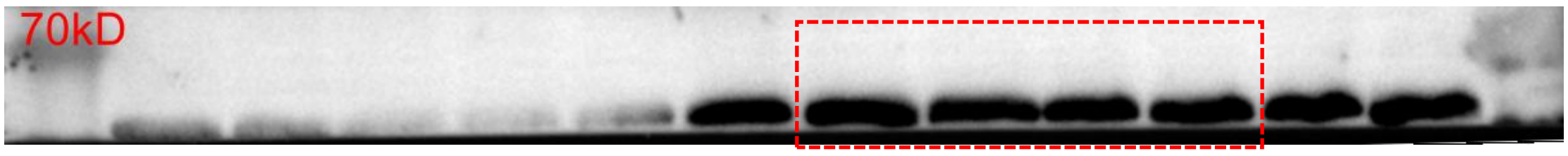

Sample 3. Sdhb

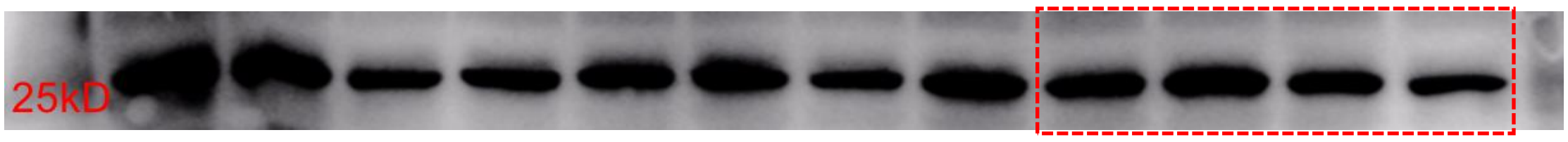

Sample 3. β-Tubulin

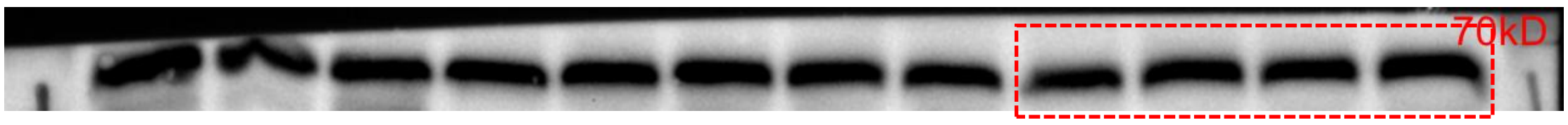

Uqcrc2

Sample 1. Uqcrc2

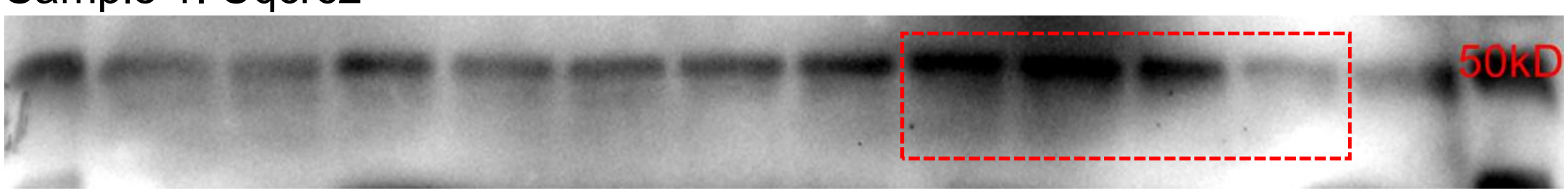

Sample 1. Gapdh

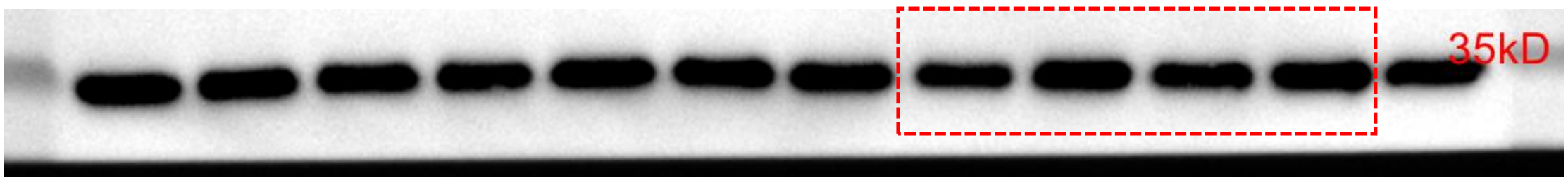

Sample 2. Uqcrc2

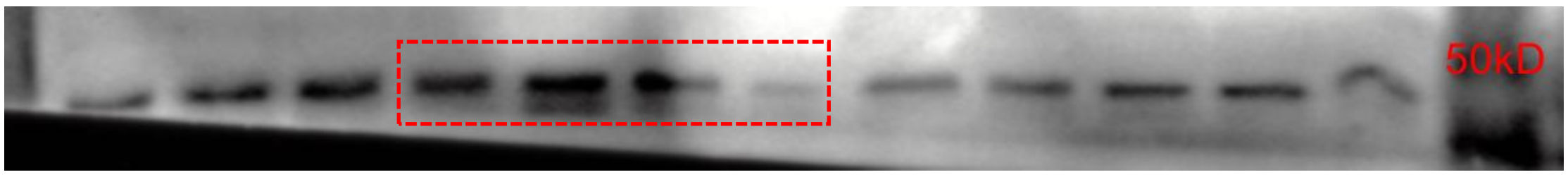

Sample 2. Gapdh

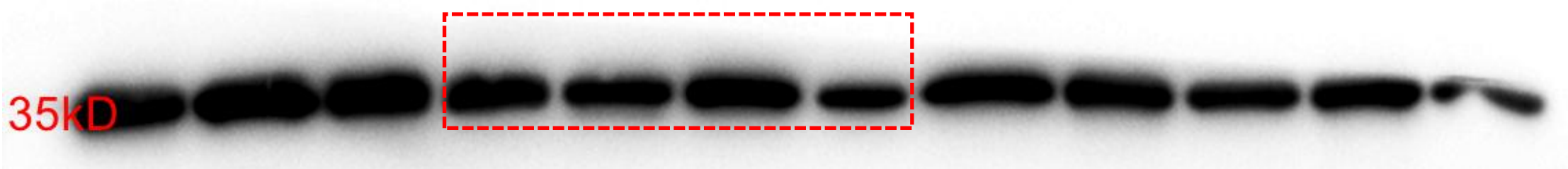

Sample 3. Uqcrc2

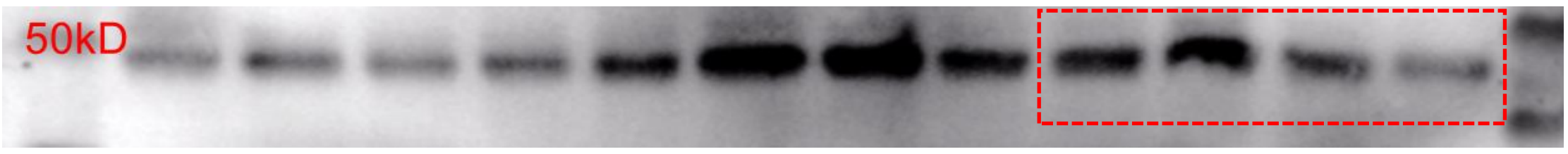

Sample 3. Gapdh

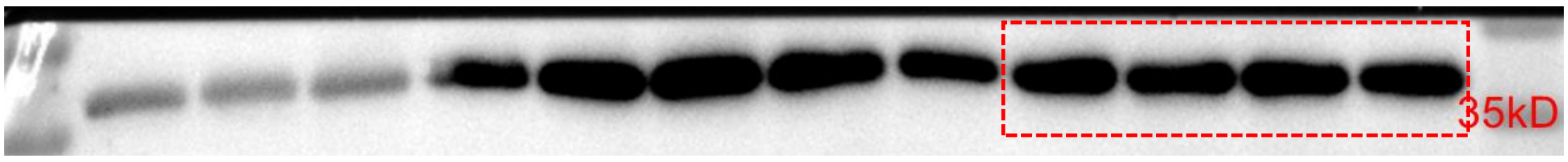

MtCO2

Sample 1. MtCO2

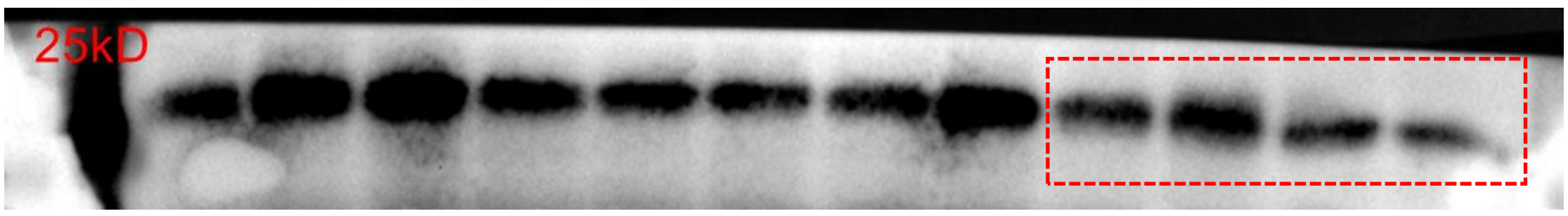

Sample 1. Gapdh

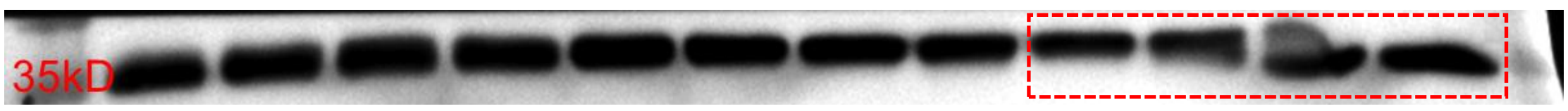

Sample 2. MtCO2

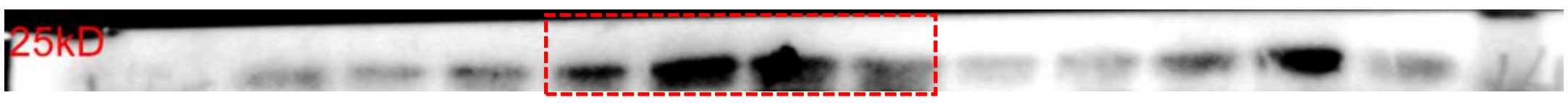

Sample 2. Gapdh

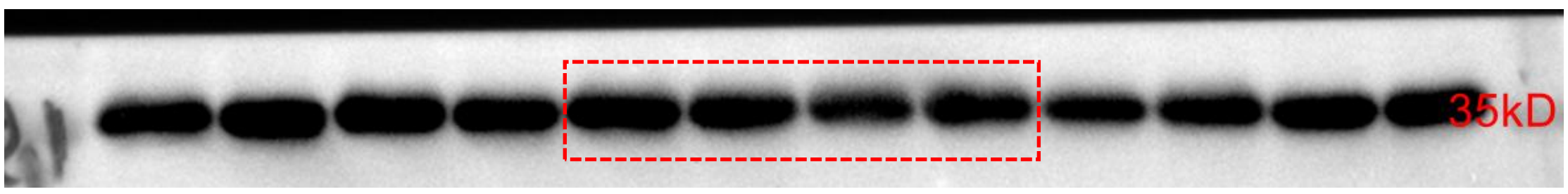

Sample 3. MtCO2

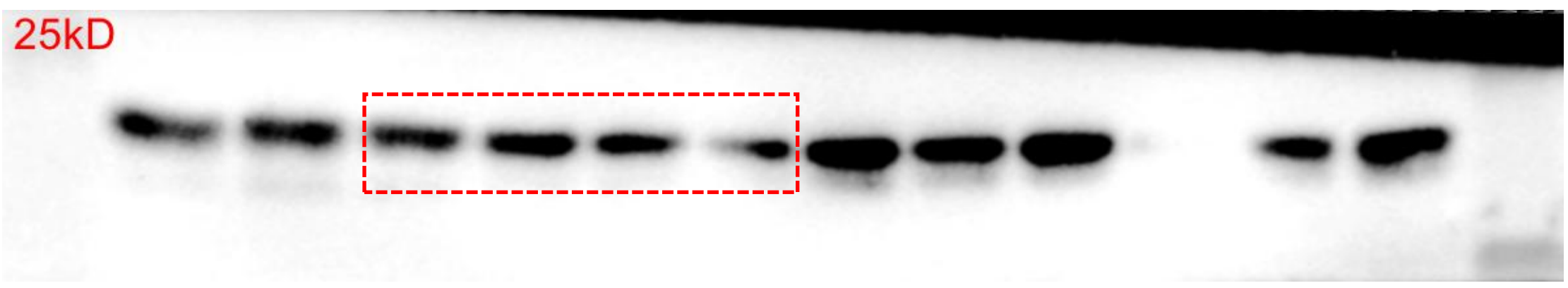

Sample 3. Gapdh

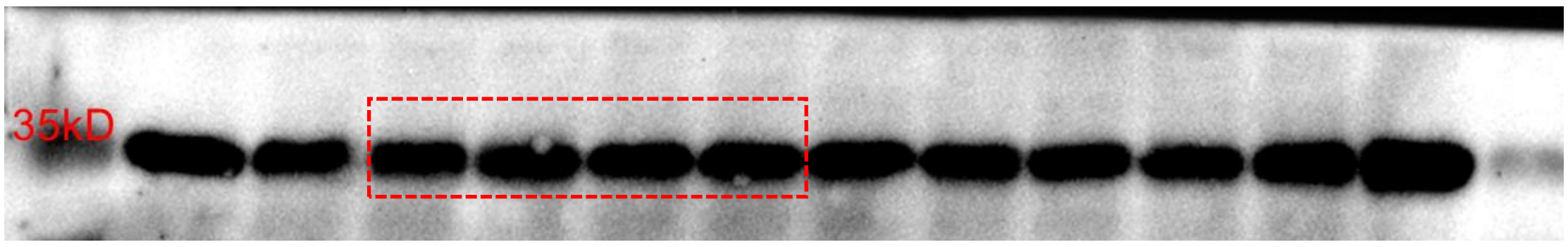

Atpaf1

Sample 1. Atpaf1

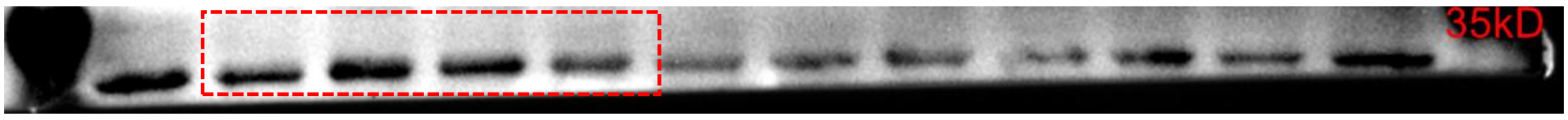

Sample 1. Tubulin

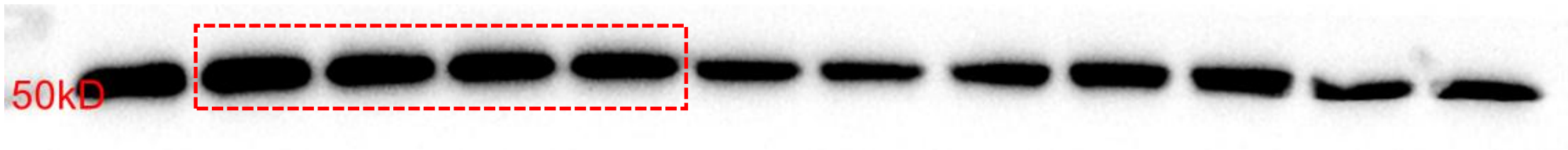

Sample 2. Atpaf1

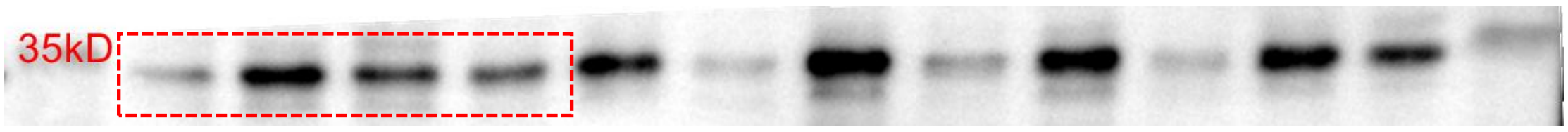

Sample 2. β-Tubulin

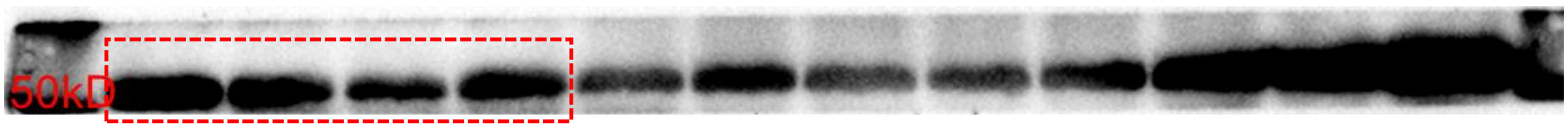

Sample 3. Atpaf1

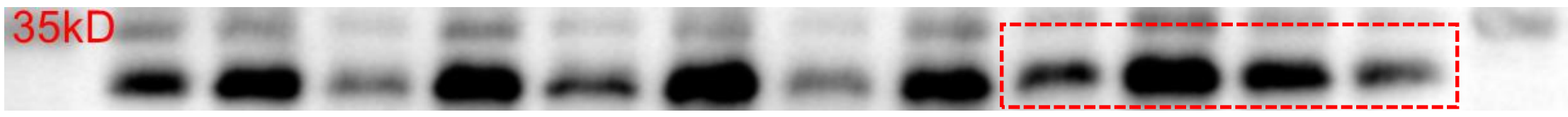

Sample 3. β-Tubulin

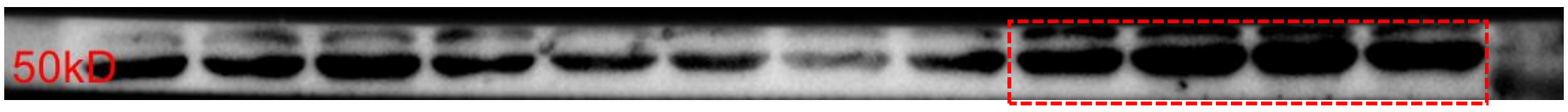

h. Nlrp3, Inos, Cleaved IL-1β, IL-10

Nlrp3

Sample 1. Nlrp3

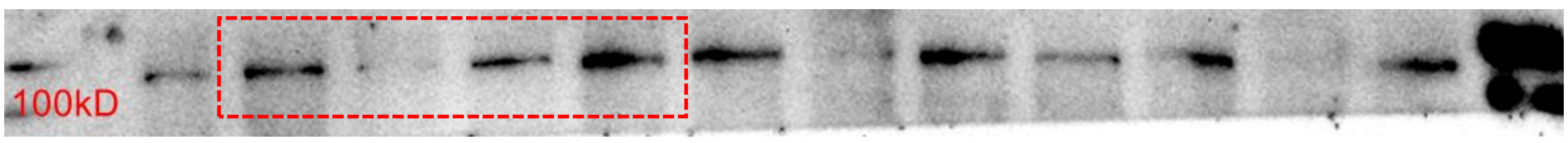

Sample 1. β-Tubulin

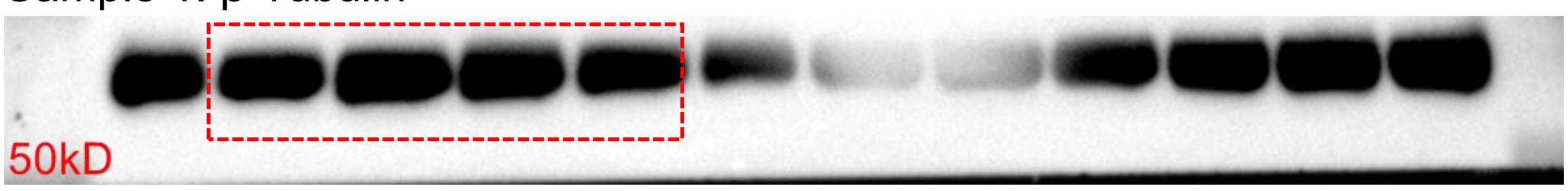

Sample 2. Nlrp3

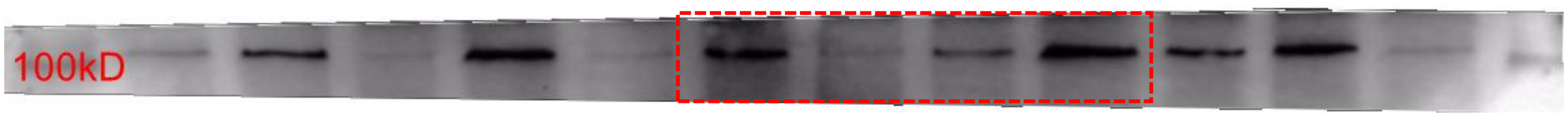

Sample 2. β-Tubulin

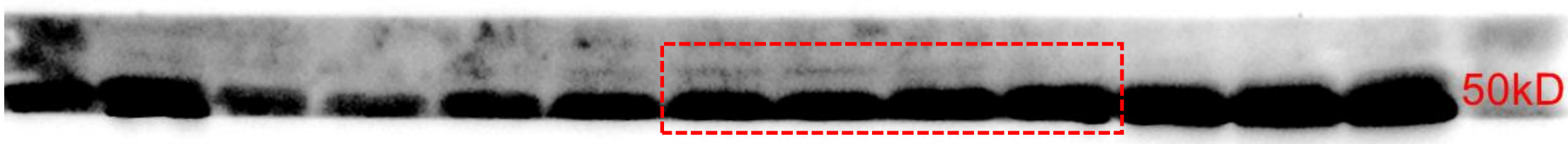

Sample 3. Nlrp3

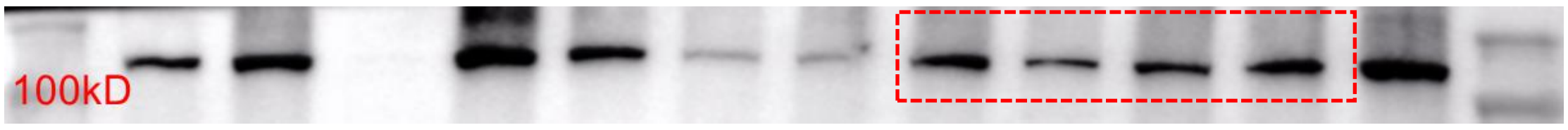

Sample 3. Tubulin

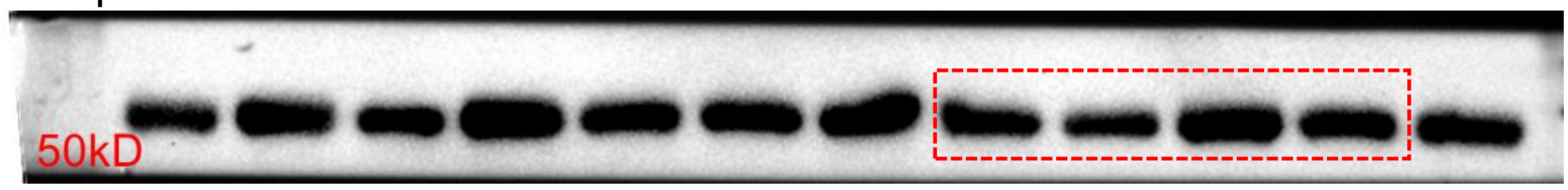

Inos

Sample 1. Inos

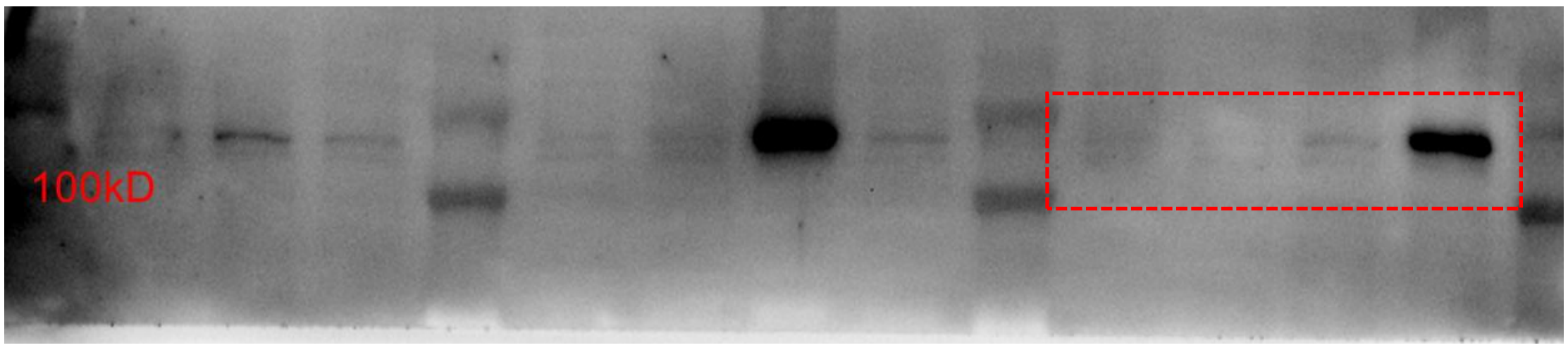

Sample 1.Gapdh

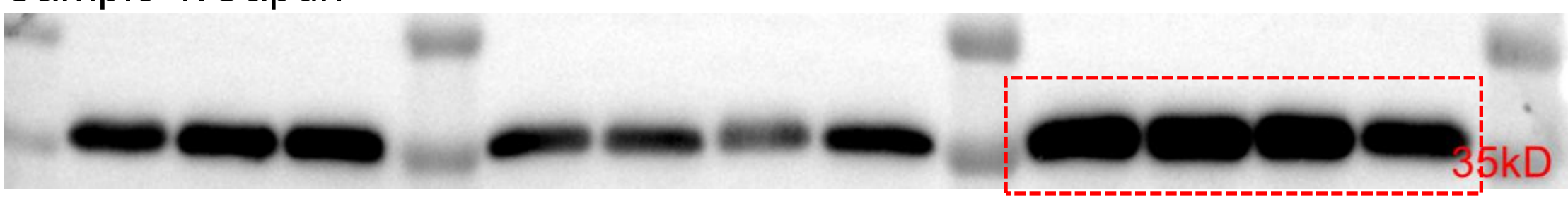

Sample 2. Inos

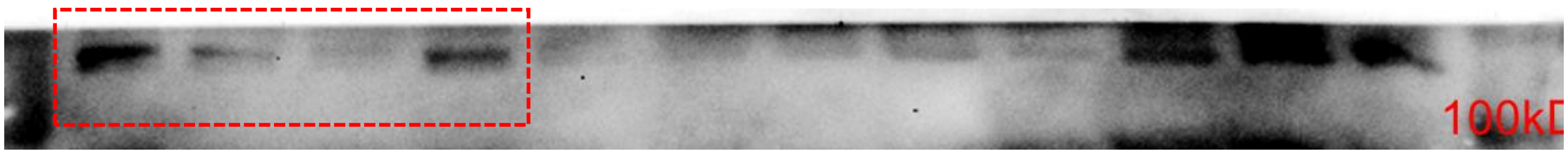

Sample 2.Gapdh

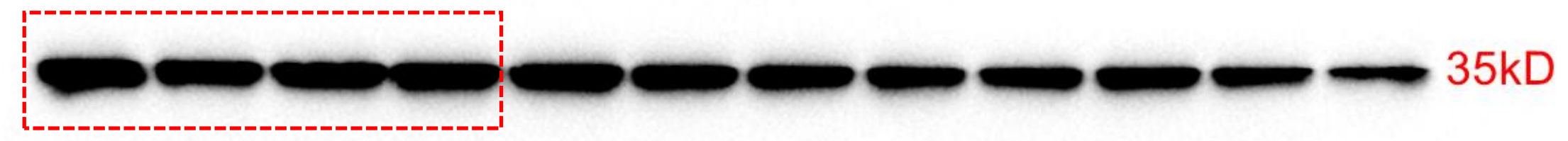

Sample 3. Inos

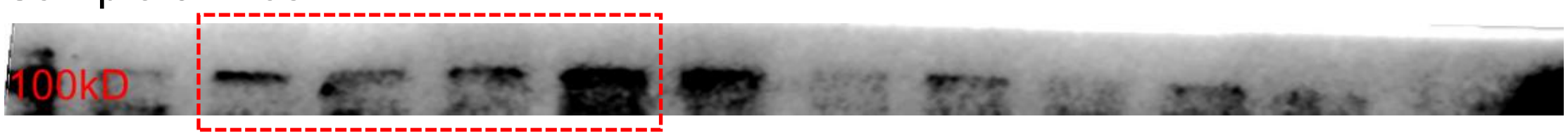

Sample 3.Gapdh

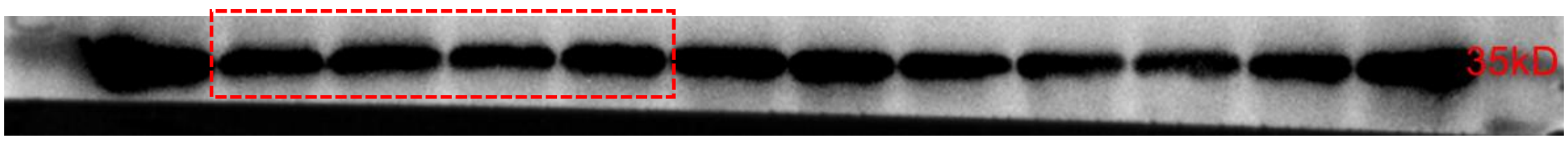

Cleaved Il-1β

Sample 1. Cleaved Il-1β

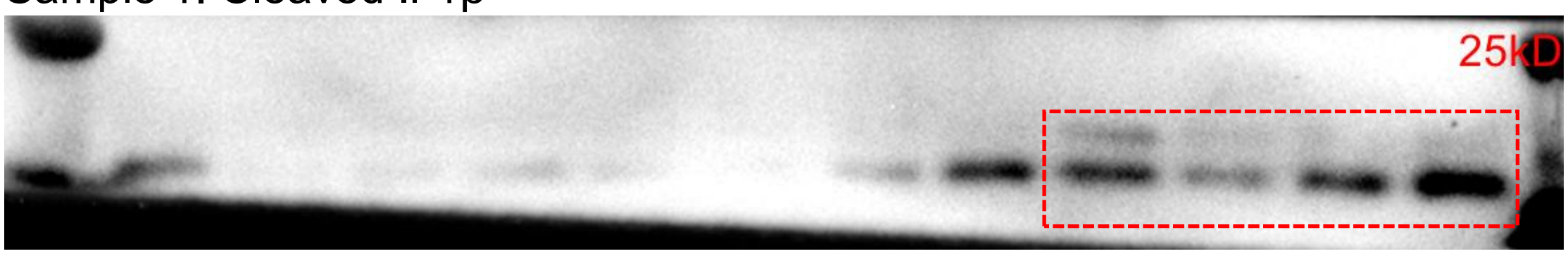

Sample 1. β-actin

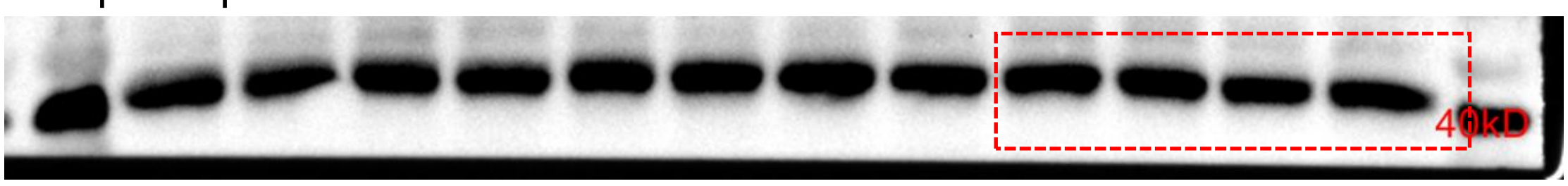

Sample 2. Cleaved Il-1β

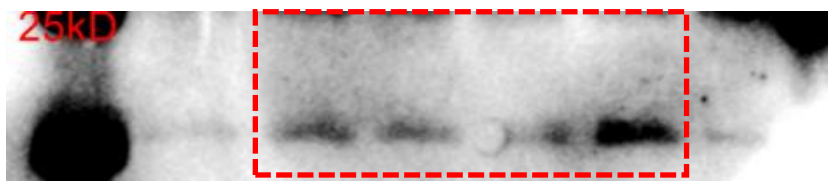

Sample 2. β-actin

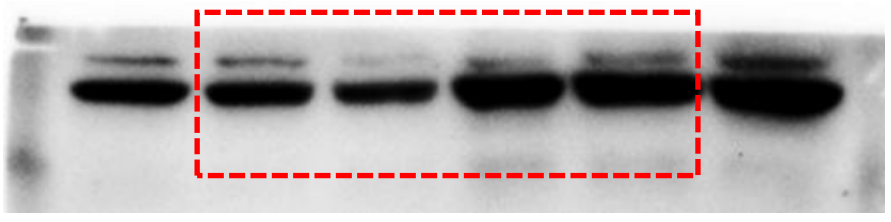

Sample 3. cleaved Il-1β

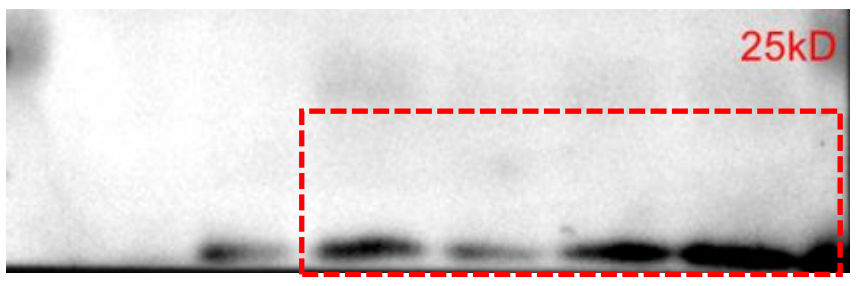

Sample 3. β-actin

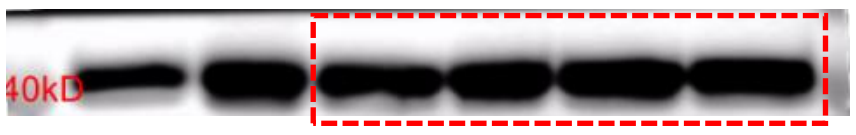

Il-10

Sample 1. Il-10

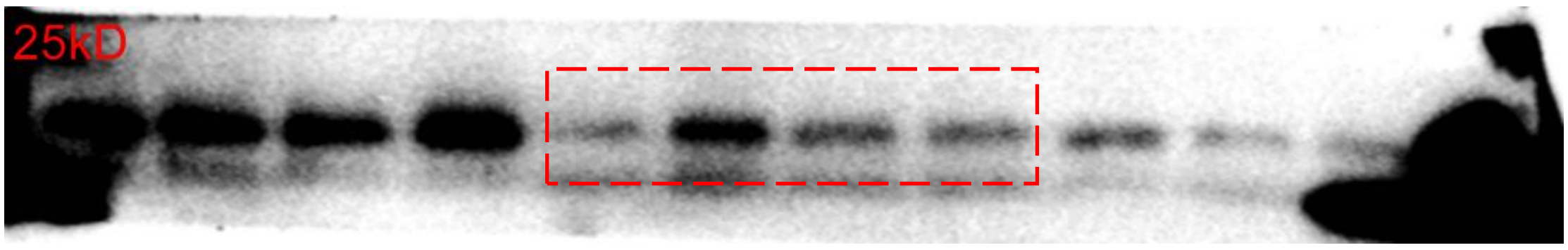

Sample 1. Gapdh

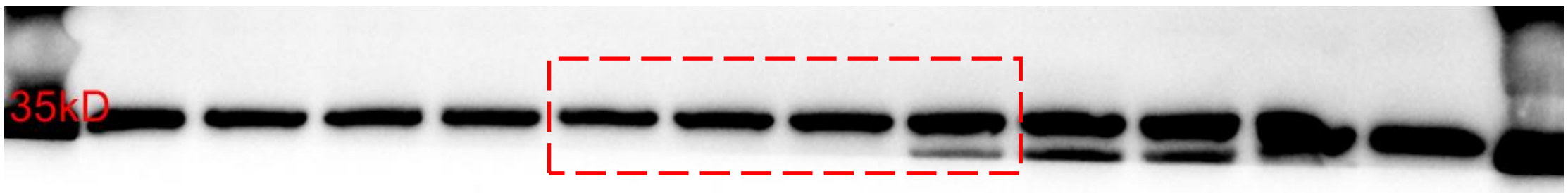

Sample 2. Il-10

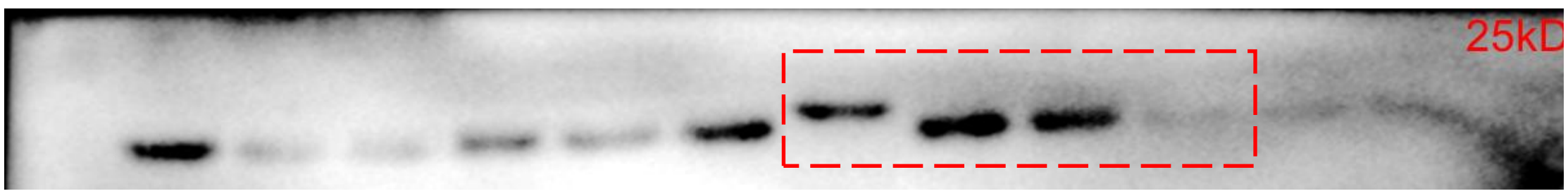

Sample 2. Gapdh

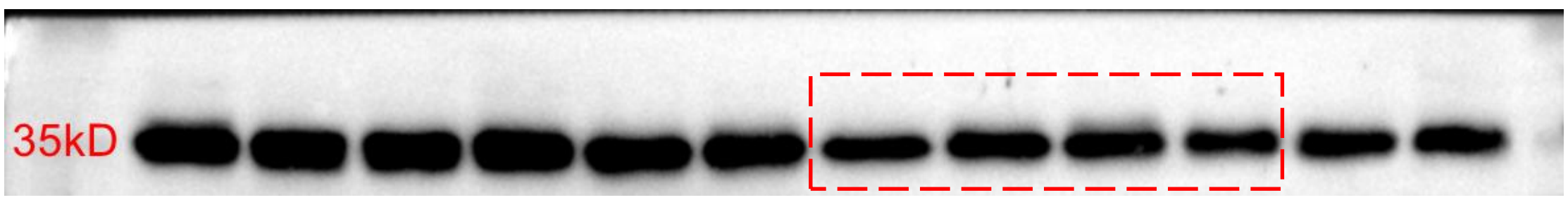

Sample 3. Il-10

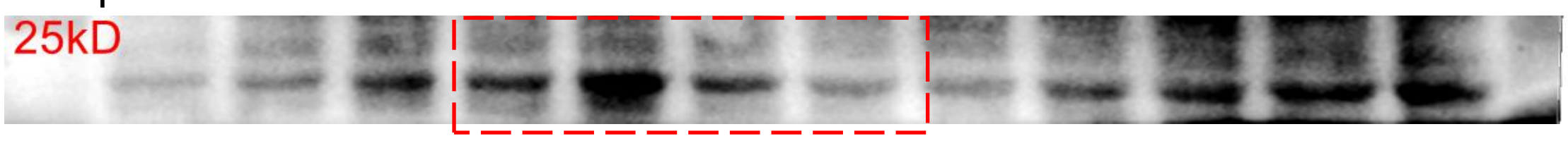

Sample 3. Gapdh

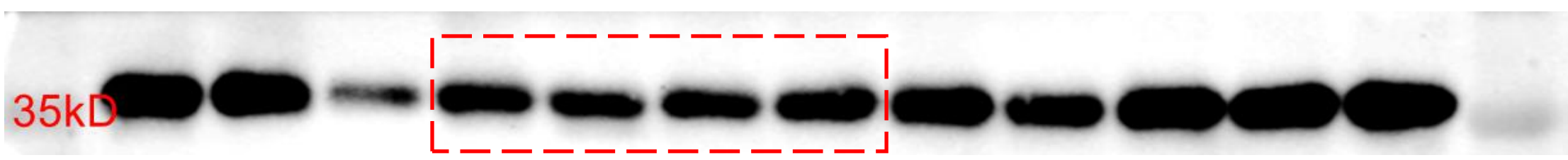

Supplementary figure 6.

Lanes within the red dotted boxes, arranged left to right: Scramble + DMSO (Scramble-DMSO), Scramble+Chloroquine (Scramble-CQ), ShRNA-Tmem9+ DMSO(ShRNA-DMSO), ShRNA-Tmem9+Chloroquine (ShRNA-CQ).

Supplementary figure 6b: Atg5, P62, Lc3II/Lc3I.

Atg5

Sample 1 Atg5

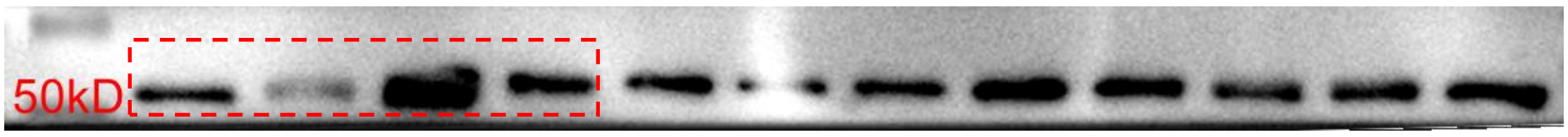

Sample 1 Gapdh

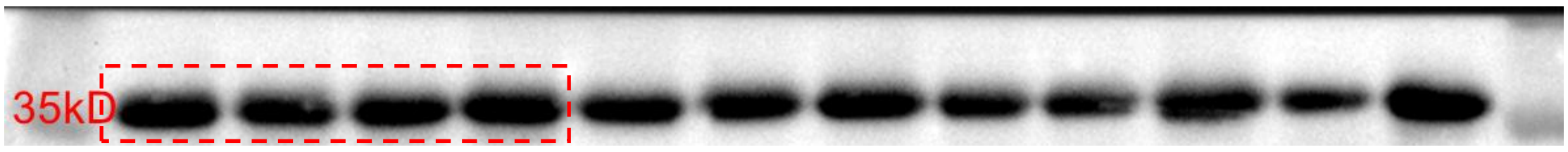

Sample 2 Atg5

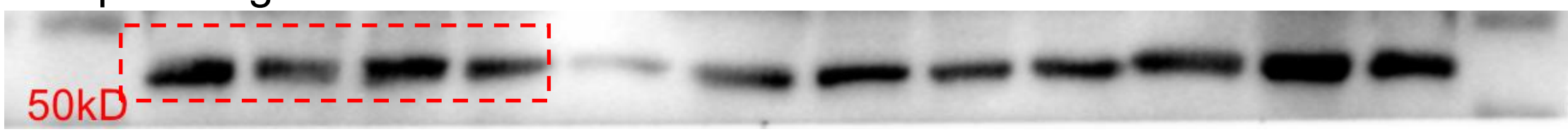

Sample 2 Gapdh

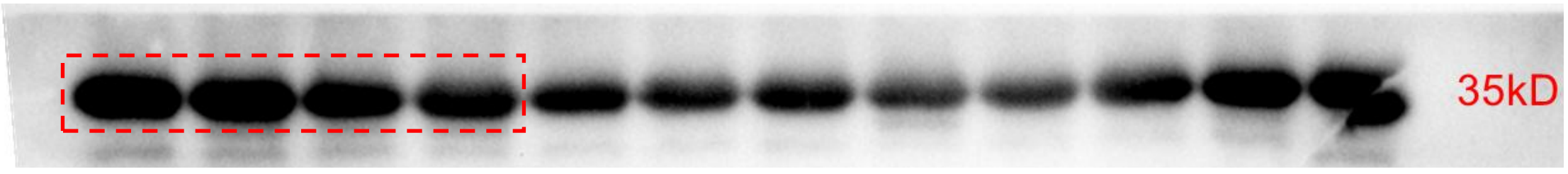

Sample 3 Atg5

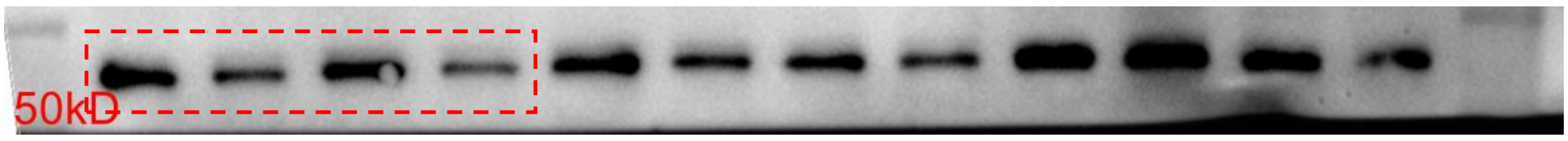

Sample 3 Gapdh

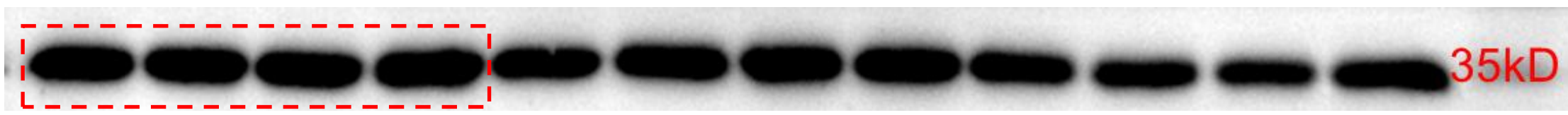

P62

Sample 1 P62

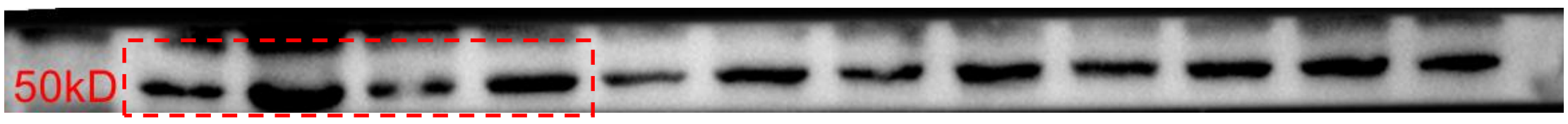

Sample 1 Gapdh

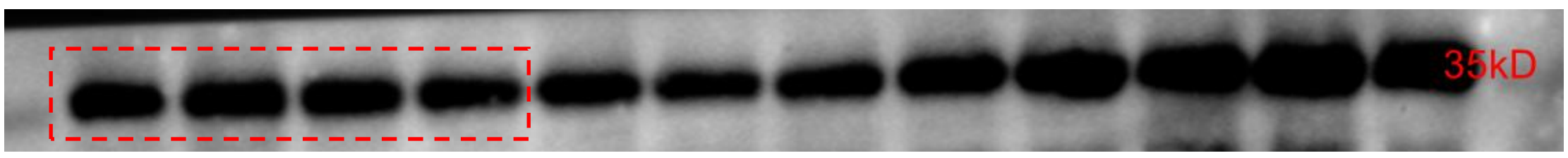

Sample 2 P62

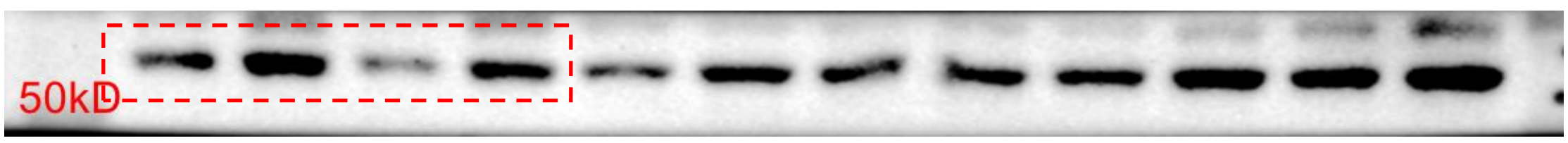

Sample 2 Gapdh

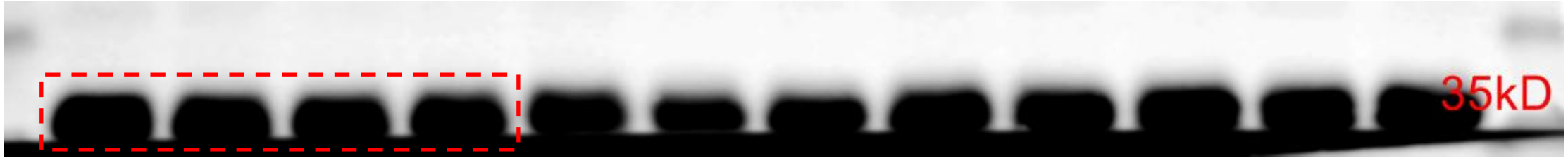

Sample 3 P62

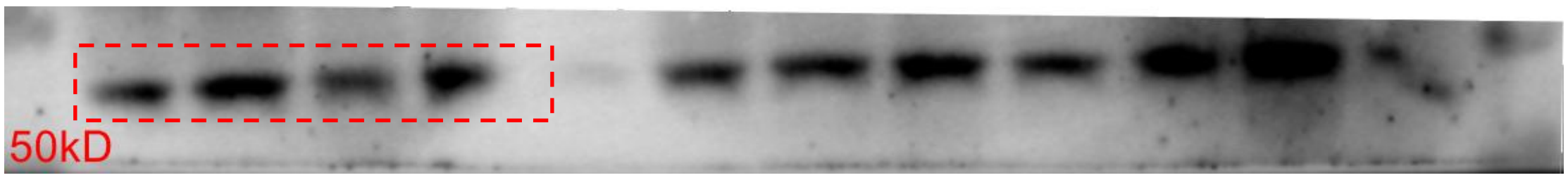

Sample 3 Gapdh

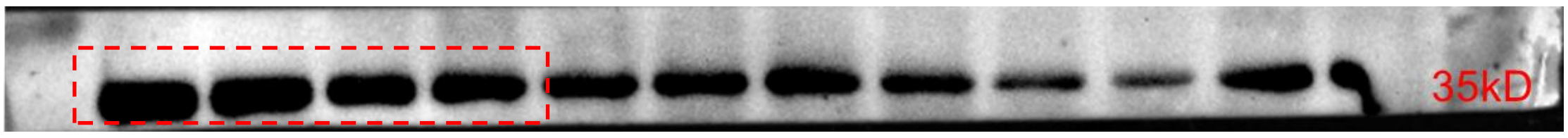

Sample 1 Lc3II/LC3I

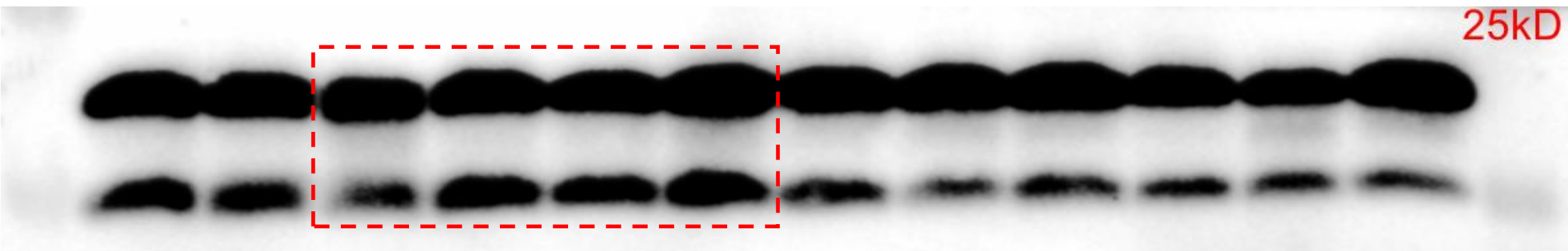

Sample 2 Lc3II/LC3I

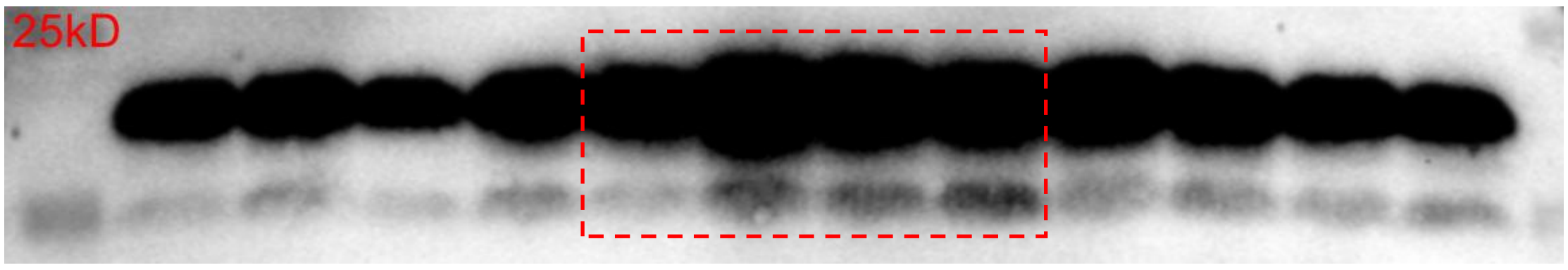

Sample 3 Lc3II/LC3I

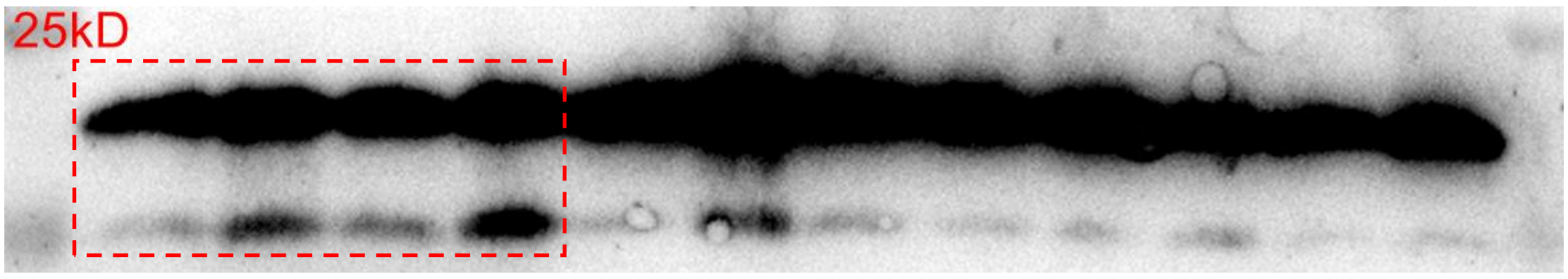

Supplementary figure 6e: Uqcrc2, Sdhb, Atpaf1,Ndufb10, Mtco2

Uqcrc2

Sample 1 Uqcrc2

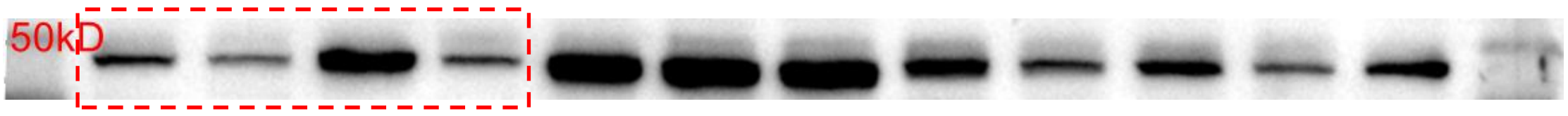

Sample 1 Gapdh

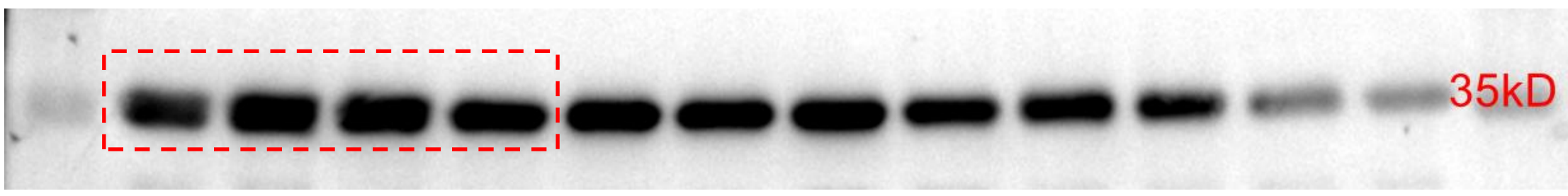

Sample 2 Uqcrc2

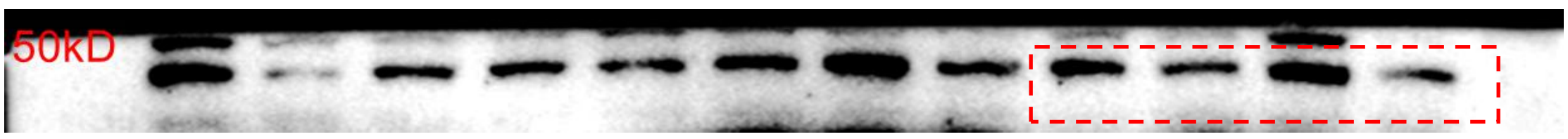

Sample 2 Gapdh

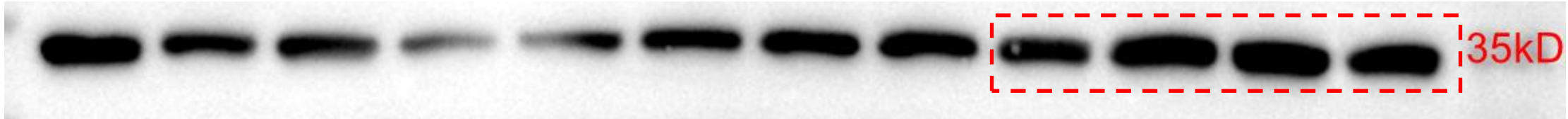

Sample 3 Uqcrc2

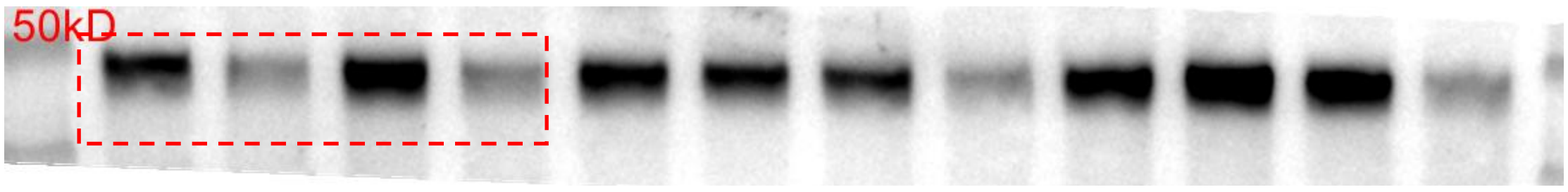

Sample 3 Gapdh

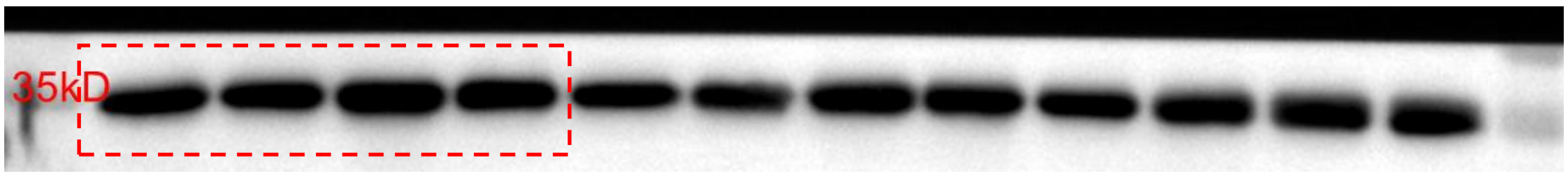

Ndufb10

Sample 1 Ndufb10

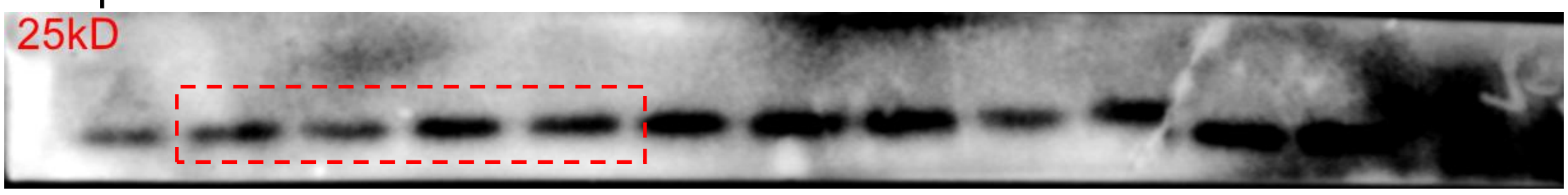

Sample 1 β-Tubulin

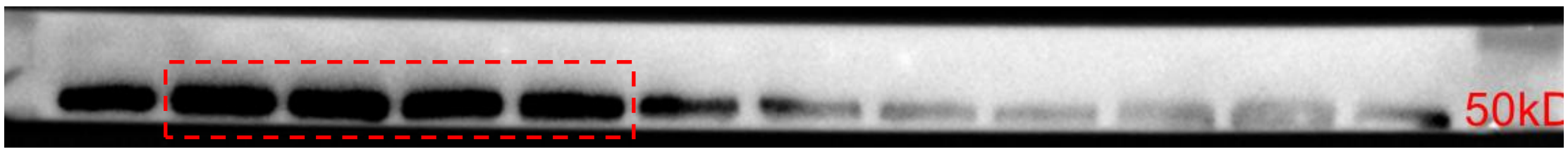

Sample 2 Ndufb10

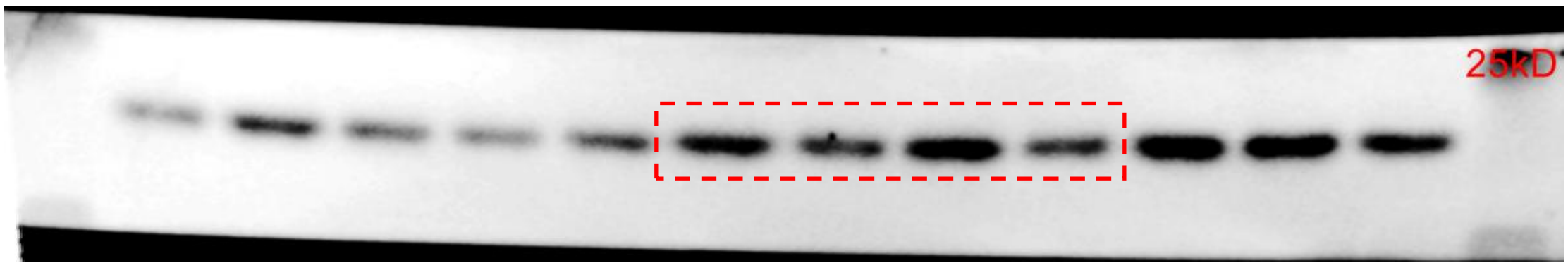

Sample 2 β-Tubulin

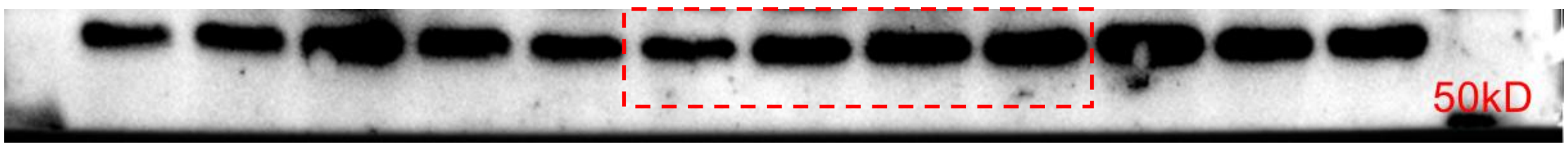

Sample 3 Ndufb10

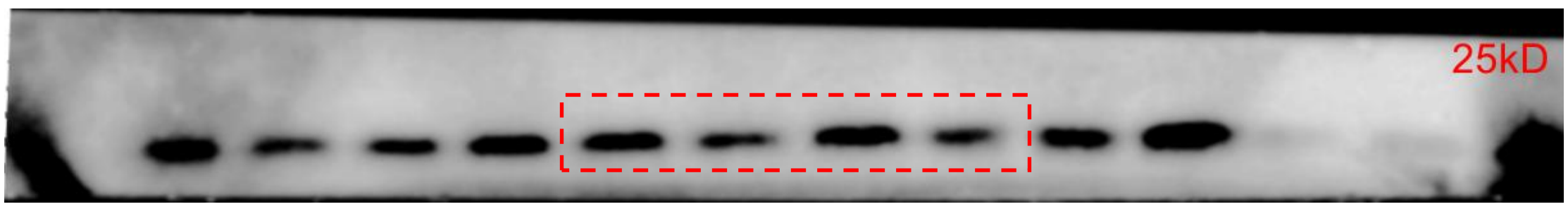

Sample 3 β-Tubulin

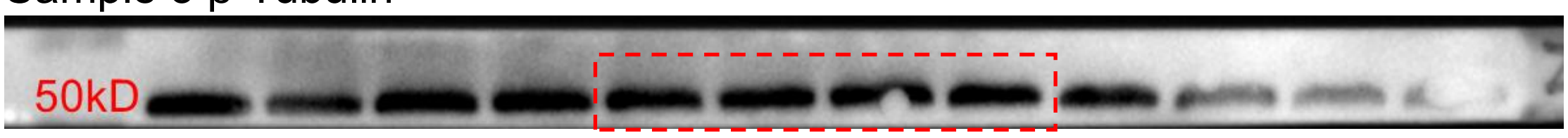

Sdhb

Sample 1 Sdhb

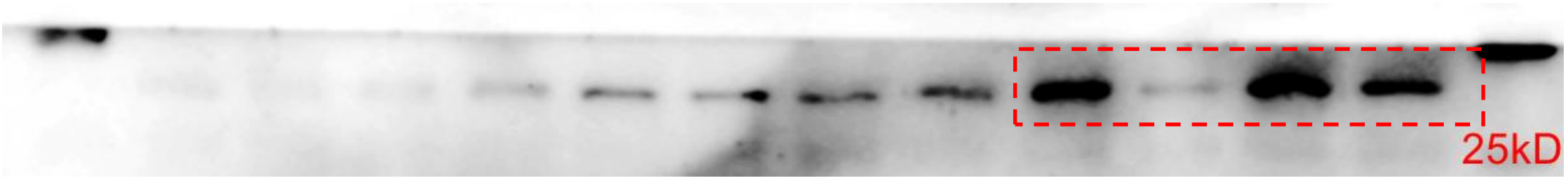

Sample 1 β-Tubulin

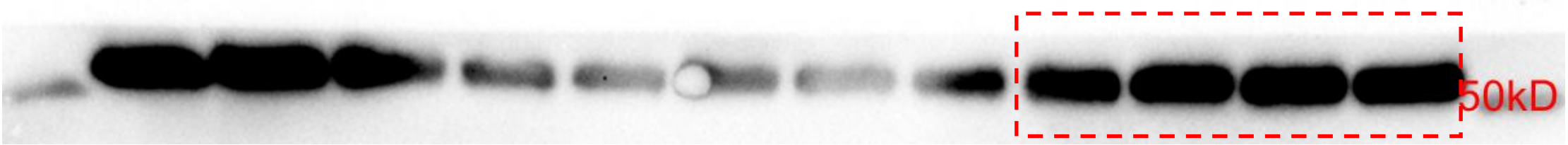

Sample 2 Sdhb

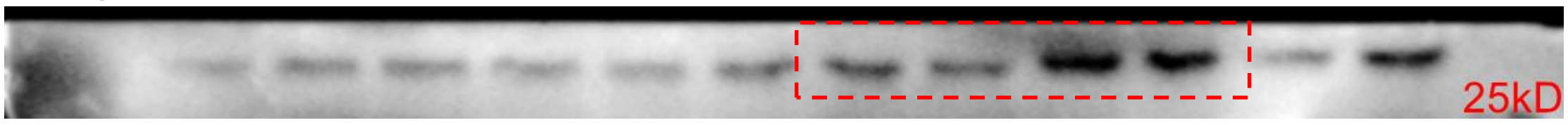

Sample 2 β-Tubulin

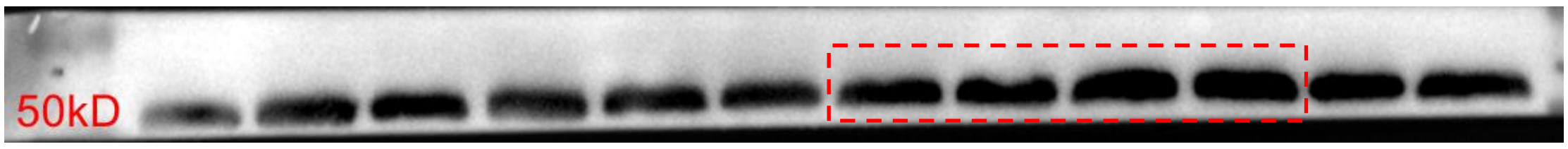

Sample 3 Sdhb

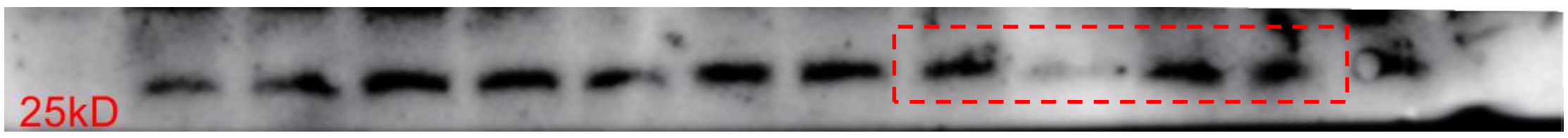

Sample 3 β-Tubulin

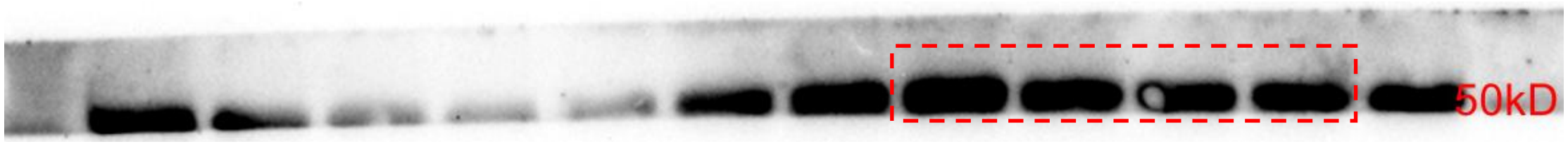

Atpaf1

Sample 1 Atpaf1

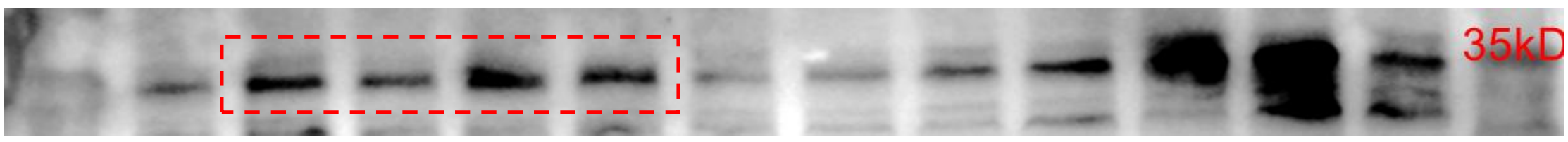

Sample 1 β-Tubulin

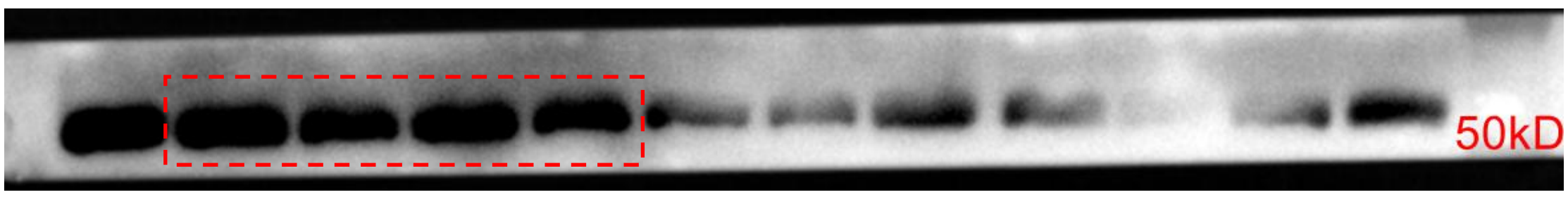

Sample 2+Sample 3 Atpaf1

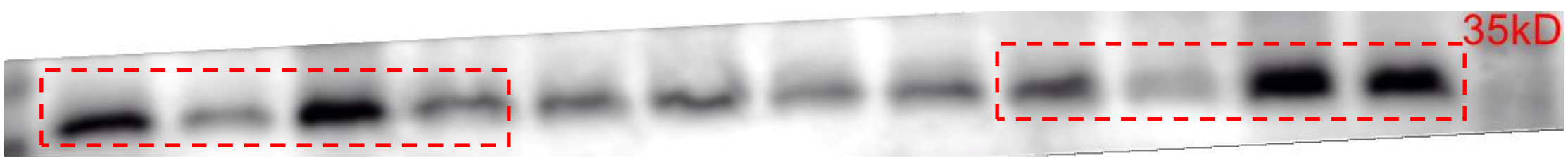

Sample 2+Sample 3 β-Tubulin

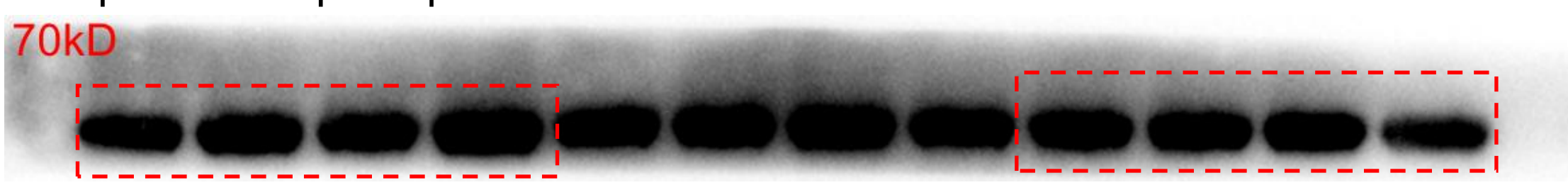

Mtco2

Sample 1 **Mtco2**

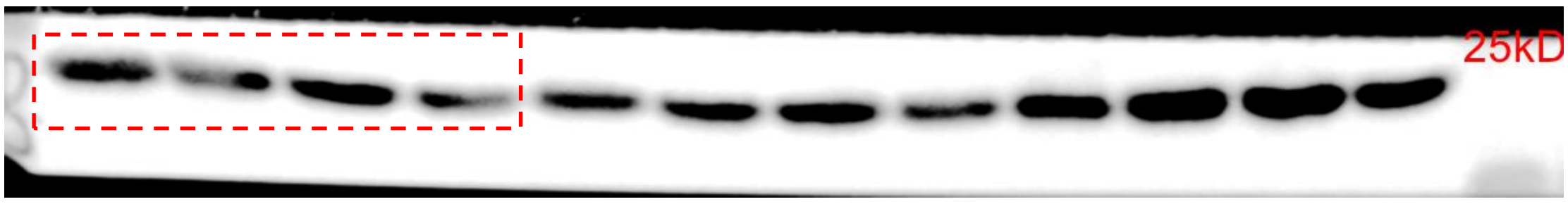

Sample 1 β-actin

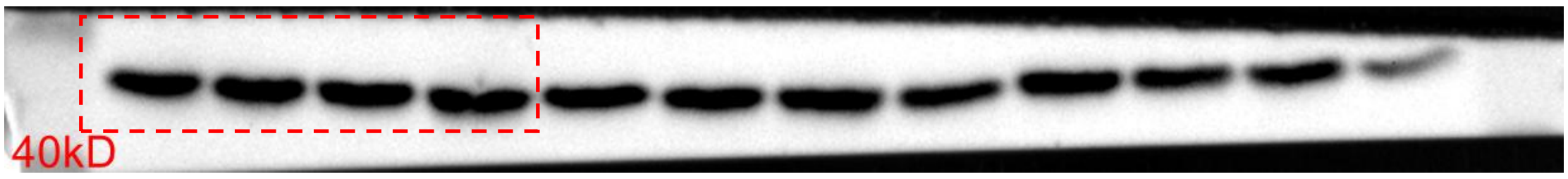

Sample 2 Mtco2

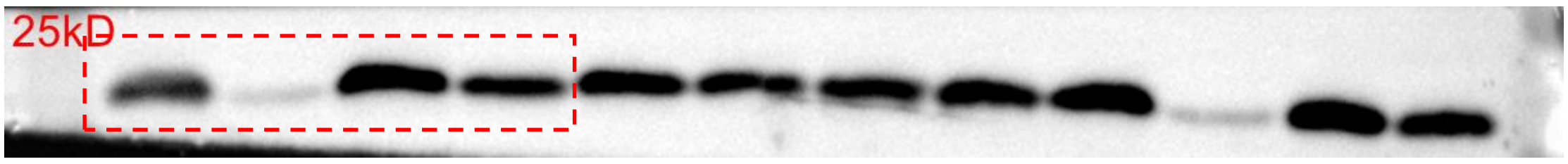

Sample 2 β-actin

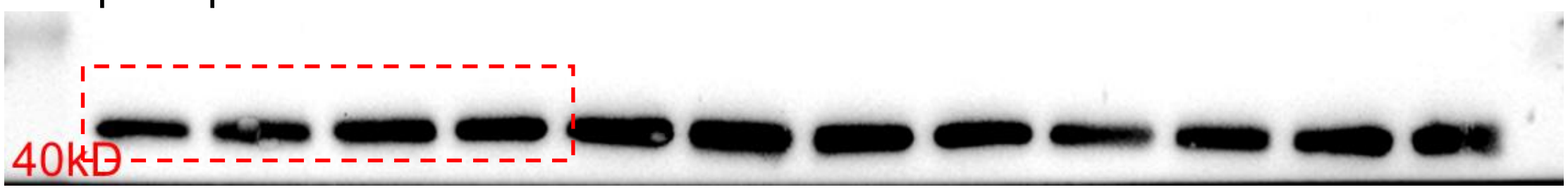

Sample 3 Mtco2

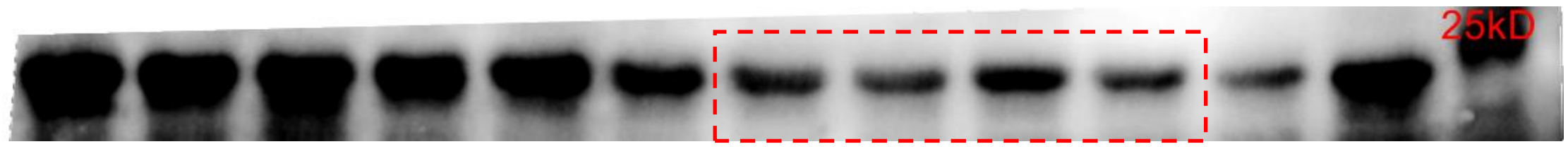

Sample 3 β-actin

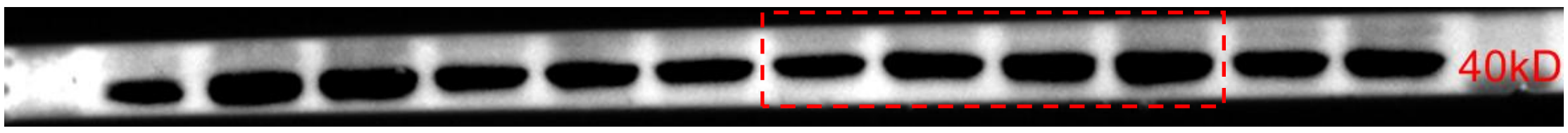

Supplementary figure 6g: Nlrp3, Cleaved Il-1β, Il-10

Nlrp3

Sample 1 Nlrp3

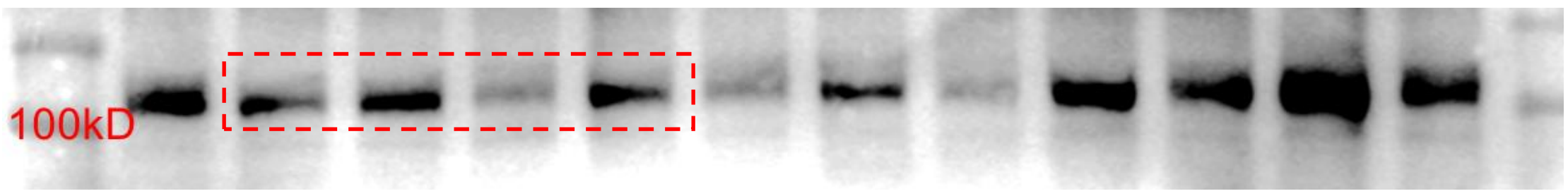

Sample 1 β-Tubulin

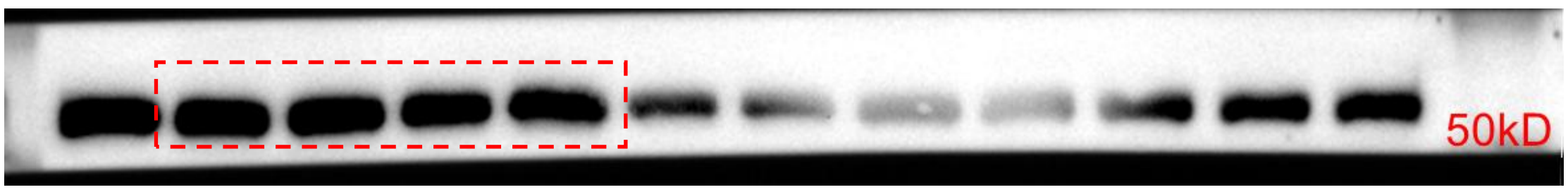

Sample 2+sample 3 NLRP3

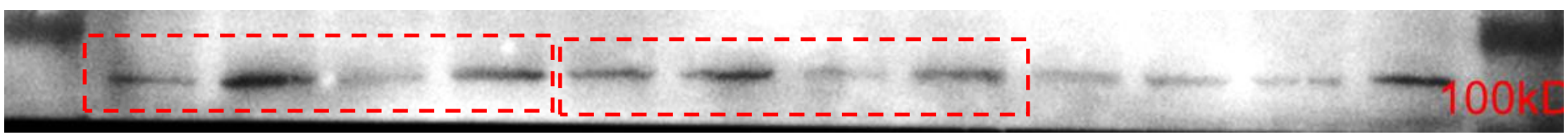

Sample 2+Sample 3 β-Tubulin

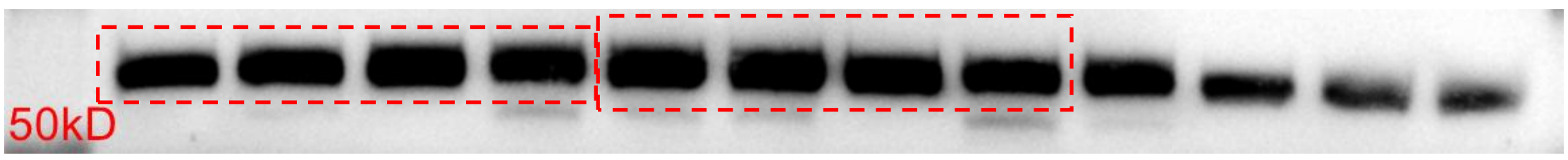

Cleaved Il-1β

Sample 1 Cleaved Il-1β

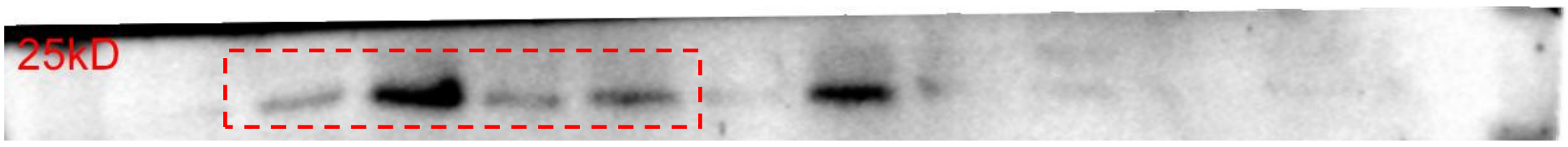

Sample 1 β-Tubulin

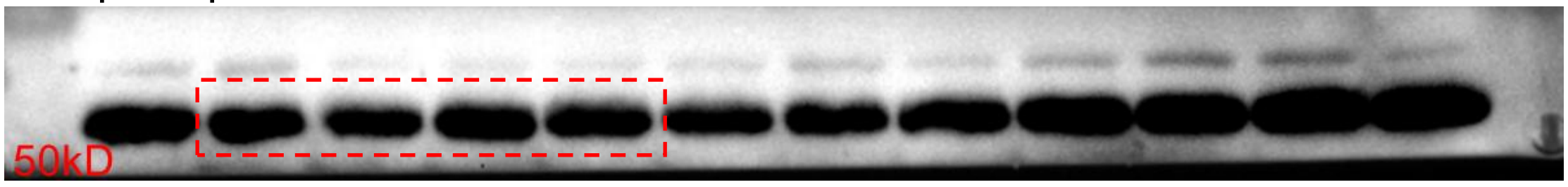

Sample 2 Cleaved Il-1β

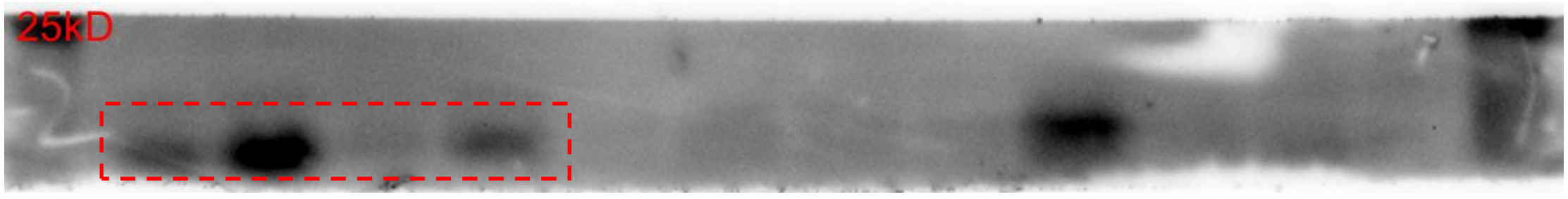

Sample 2 β-Tubulin

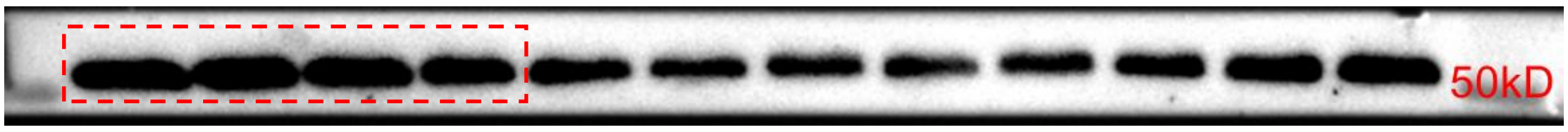

Sample 3 Cleaved Il-1β

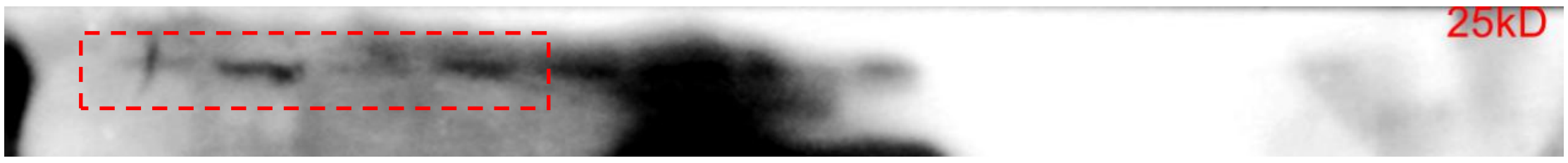

Sample 3 β-Tubulin

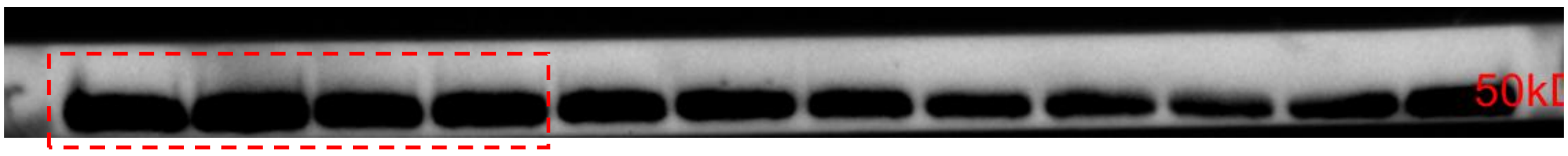

Il-10

Sample 1 Il-10

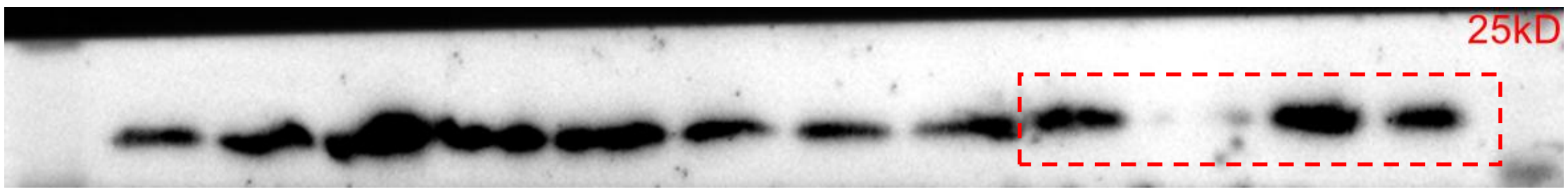

Sample 1 β-Tubulin

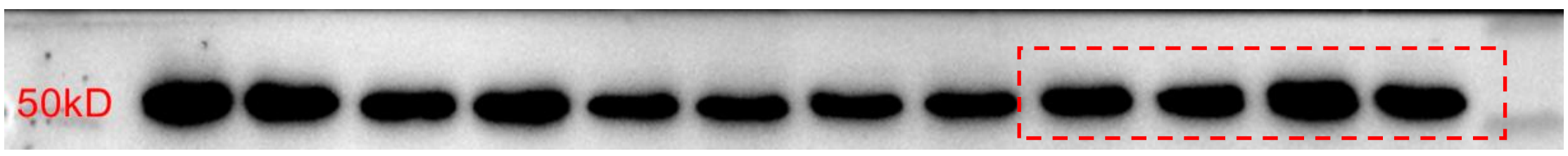

Sample 2 Il-10

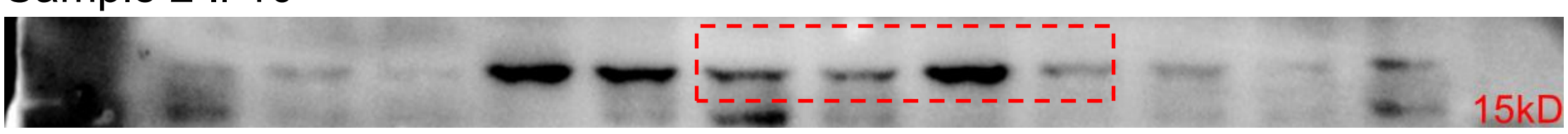

Sample 2 β-Tubulin

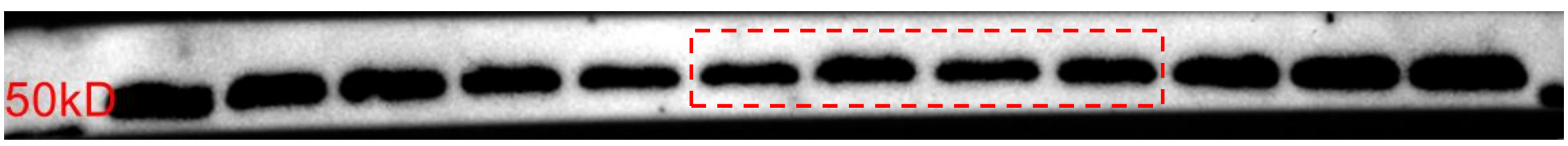

Sample 3 Il-10

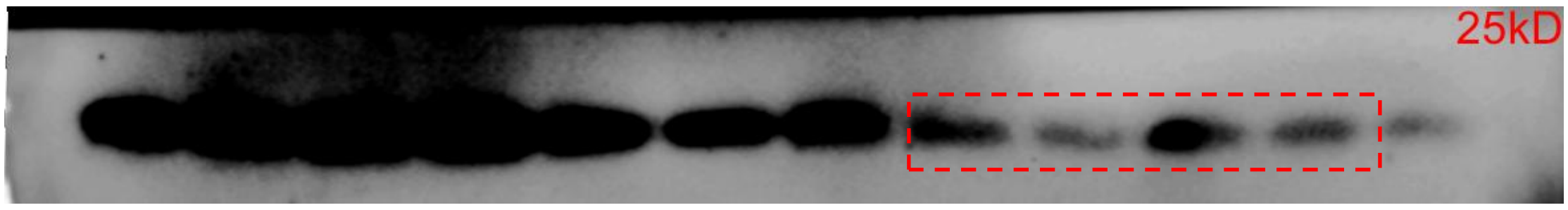

Sample 3 β-Tubulin

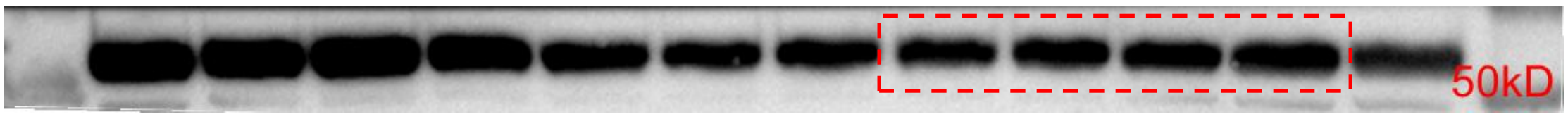

Supplementary figure 7c.

Lanes within the red dotted boxes, arranged left to right: Sample 1 (Scarmble, ShRNA-Tmem9), Sample 2 (Scarmble, ShRNA-Tmem9), Sample 3 (Scarmble, ShRNA-Tmem9).

Total Lkb1, Sample 1-3

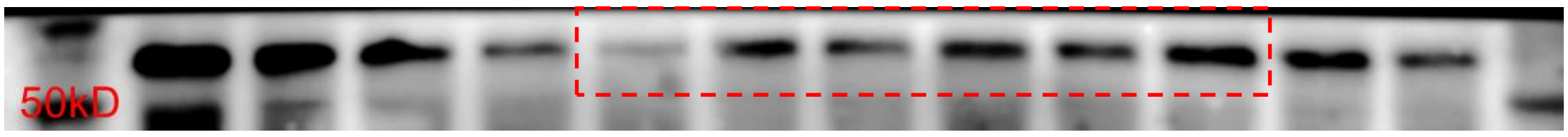

Gapdh, Sample 1-3

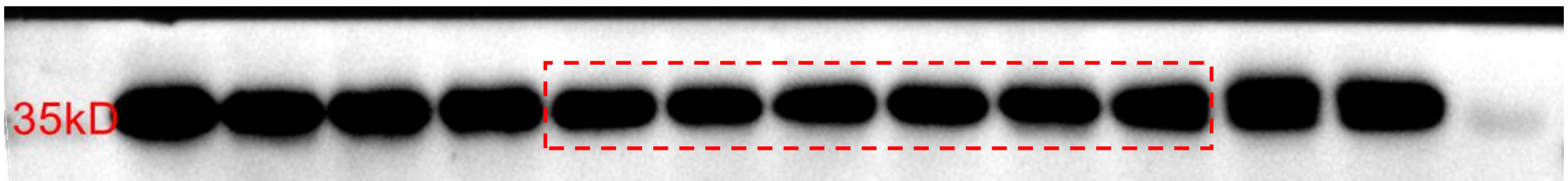

Lysosomal Lkb1, Sample 1-3

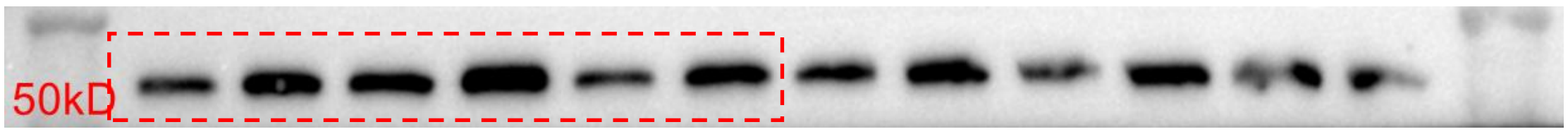

Lysosomal Lamp1, Sample 1-3

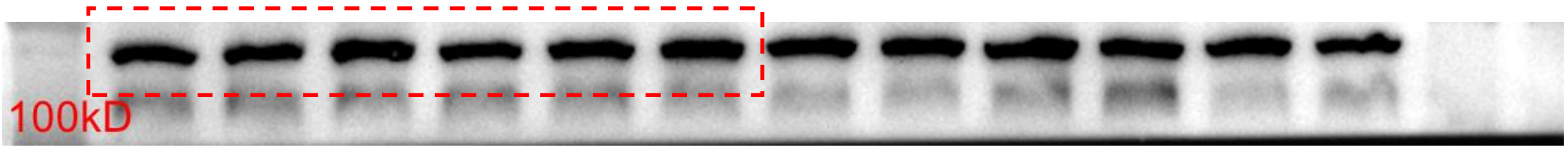

Supplementary figure 8.

Lanes within the red dotted boxes, arranged left to right: Negative Control + DMSO (NC-DMSO), Negative Control + Concanavalin A (NC-Con A), OE Tmem9 + DMSO (OE-DMSO), OE Tmem9 + Concanavalin A (OE-Con A).

a. Lkb1, Ampkα/p-Ampkα, Nlrp3, Cpt1a

Lkb1

Sample 1. Lkb1

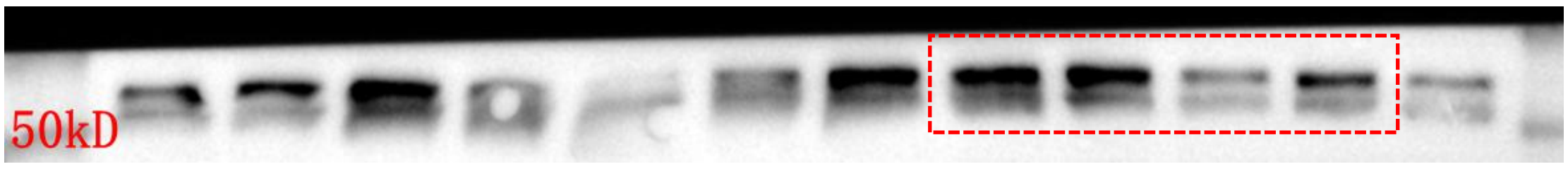

Sample 1. Gapdh

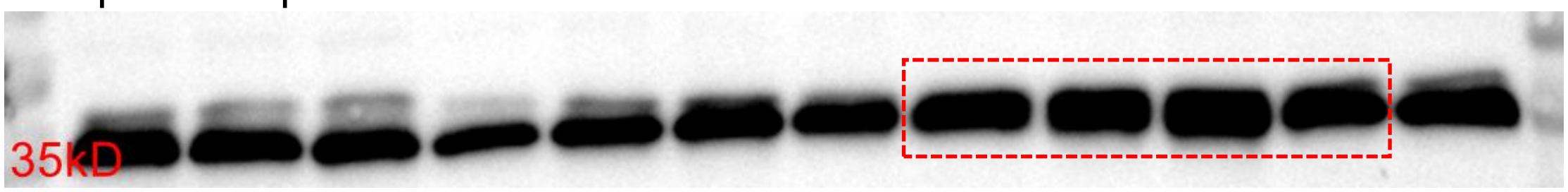

Sample 2. Lkb1

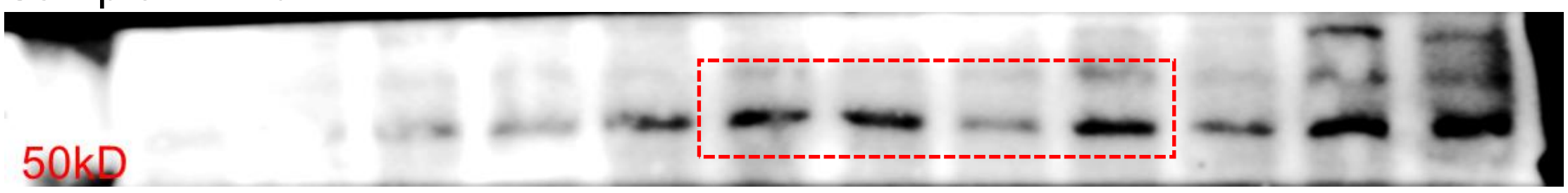

Sample 2. Gapdh

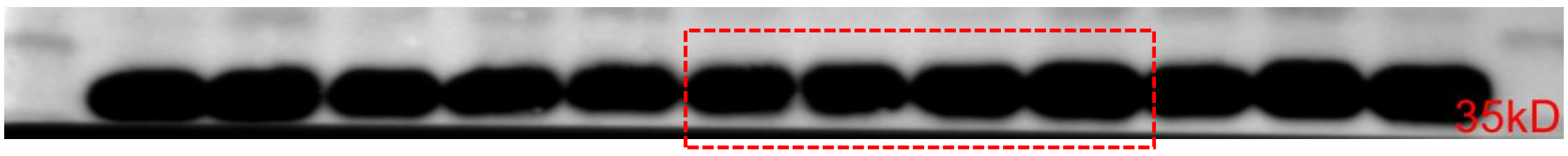

Sample 3. Lkb1

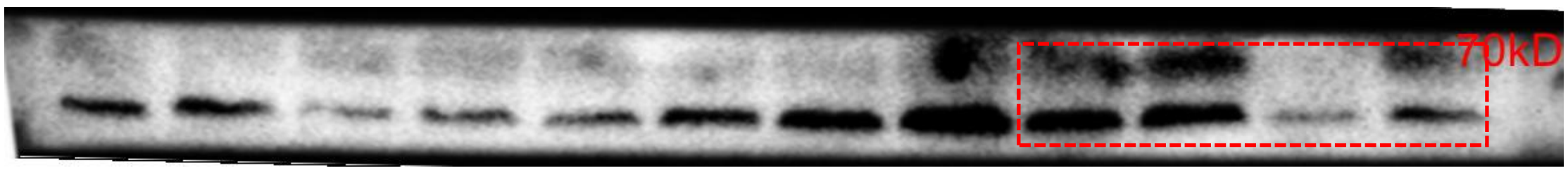

Sample 3. Gapdh

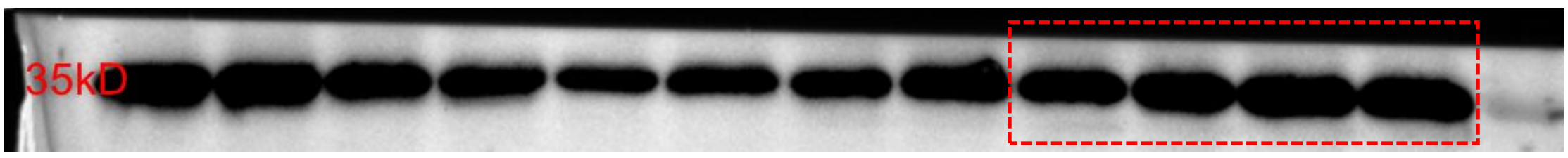

Ampkα/p-Ampkα

Sample 1. Ampkα

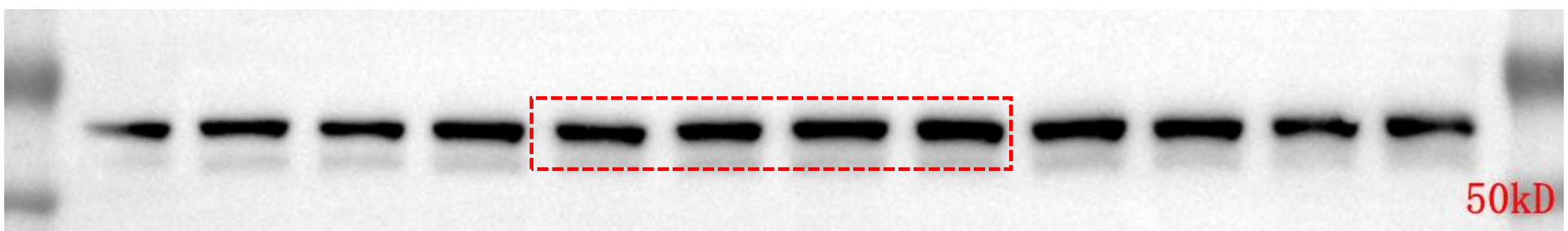

Sample 1. p-Ampkα

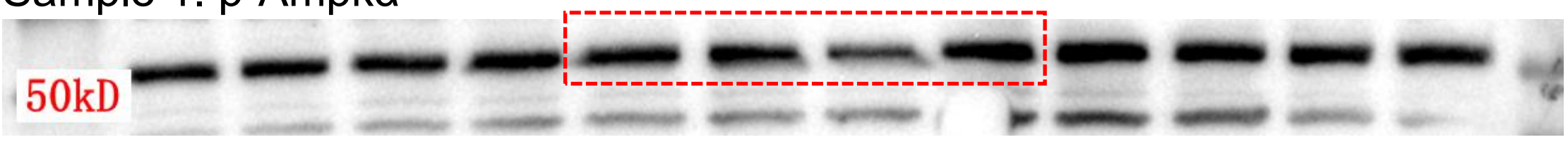

Sample 2 Ampkα

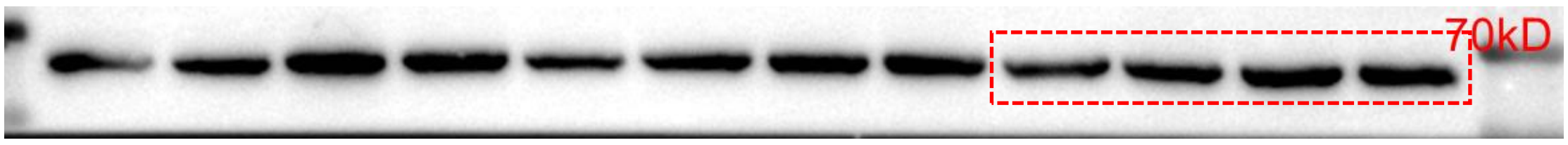

Sample 2. p-Ampkα

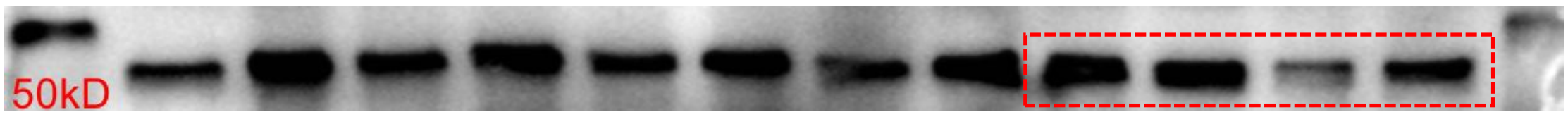

Sample 3 Ampkα

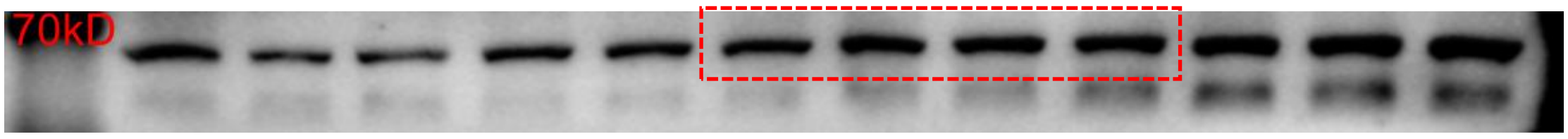

Sample 3. p-Ampkα

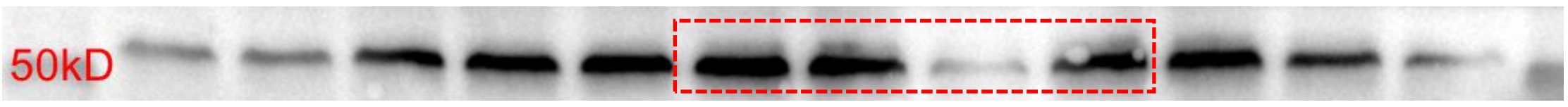

Nlrp3

Sample 1. Nlrp3

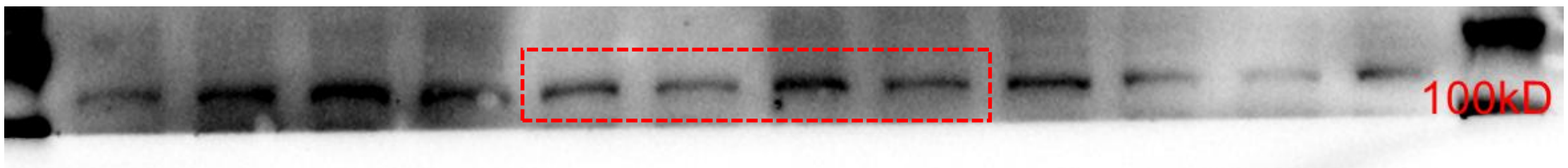

Sample 1. β actin

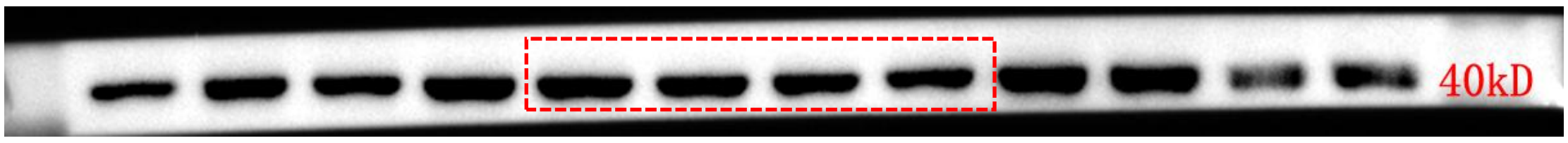

Sample 2. Nlrp3

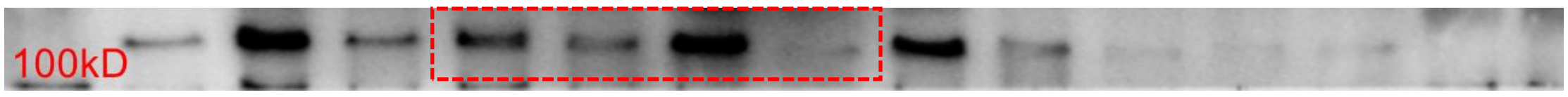

Sample 2. β actin

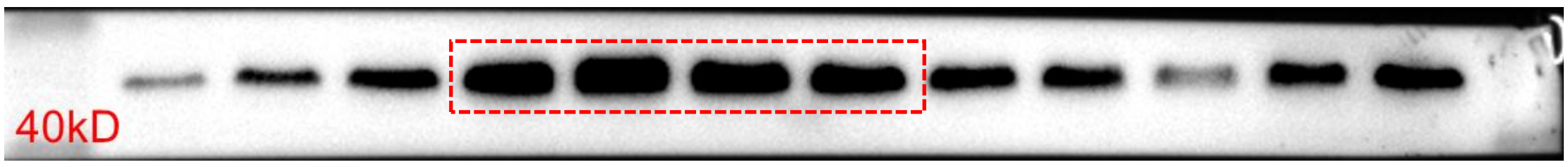

Sample 3. Nlrp3

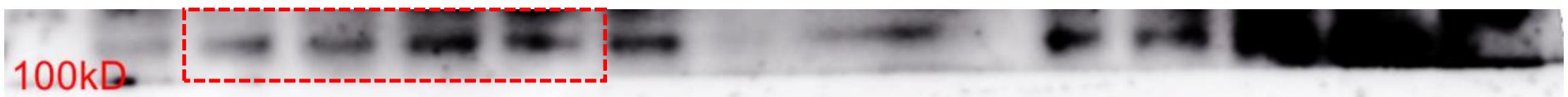

Sample 3. β actin

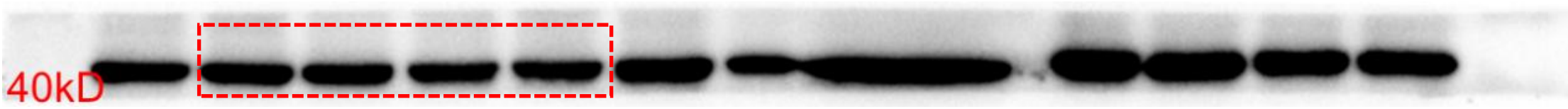

Cpt1a

Sample 1+Sample 2. Cpt1a

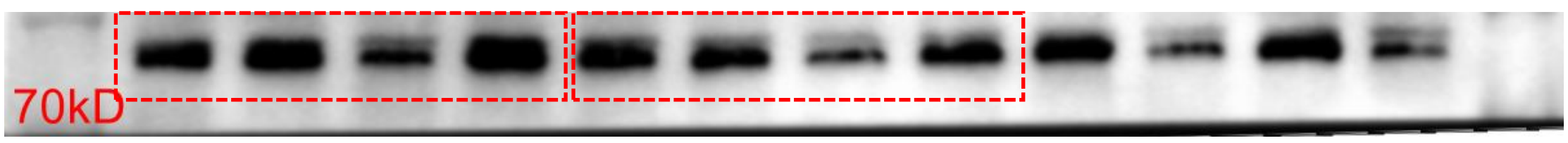

Sample 1+Sample 2. β Tubulin

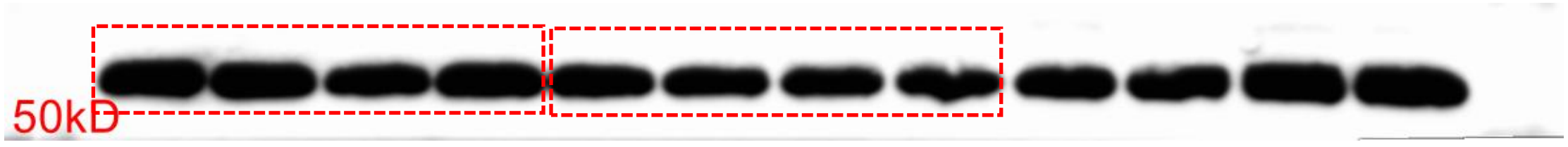

Sample3. Cpt1a

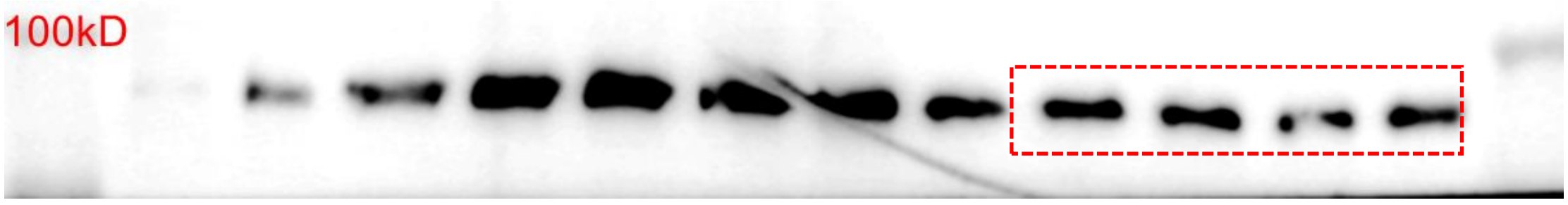

Sample3. β Tubulin

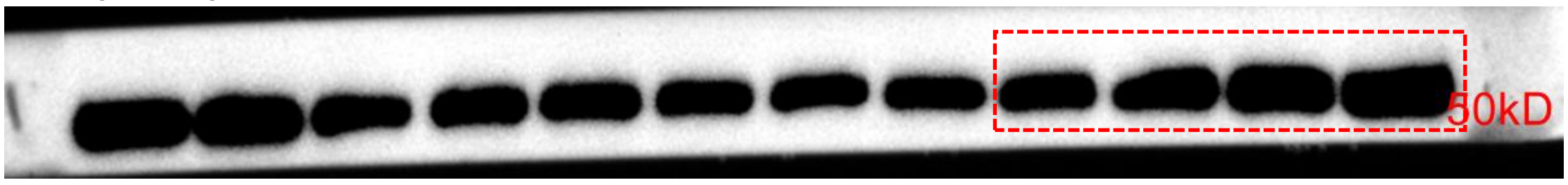

Supplementary figure 8b. Sdhb, Mtco2, Atpaf1, Il-10

Sdhb

Sample 1+Sample 2. Sdhb

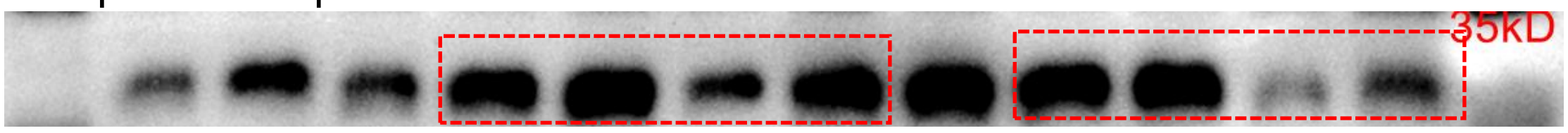

Sample 1 Mtco2

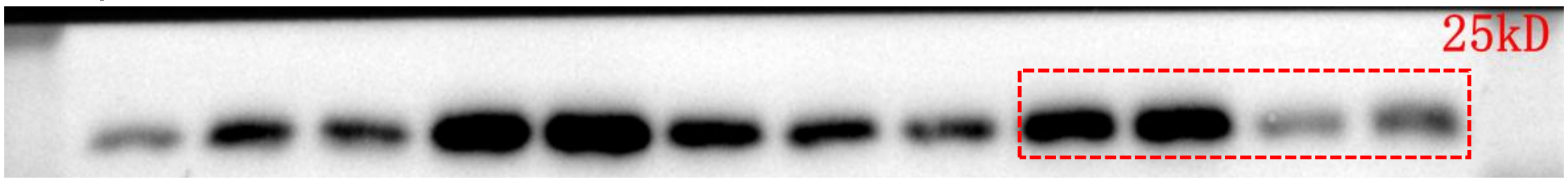

Sample 1+ 2 β-Tubulin or Sample 1+2 Sdhb; Sample 1 β-Tubulin for Mtco2

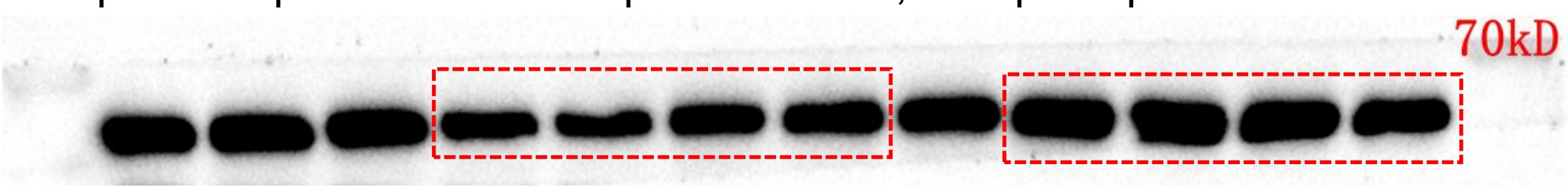

Sample 2 Mtco2

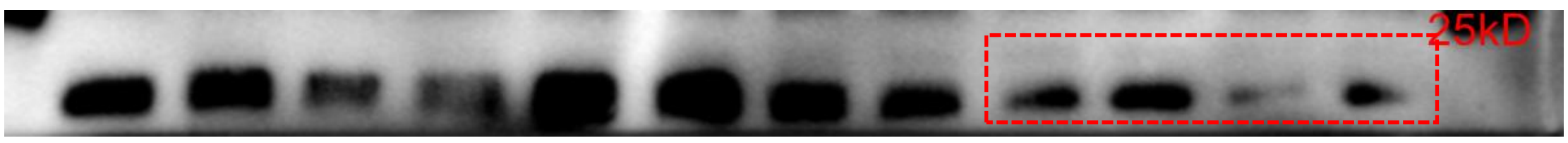

Sample 2 β-Tubulin (for Mtco2)

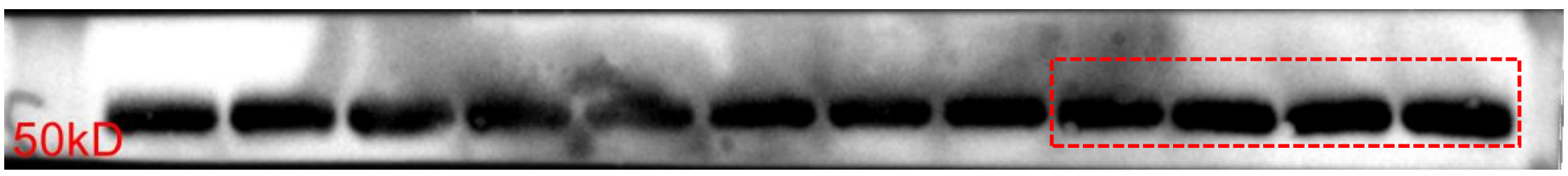

Sample 3 Sdhb

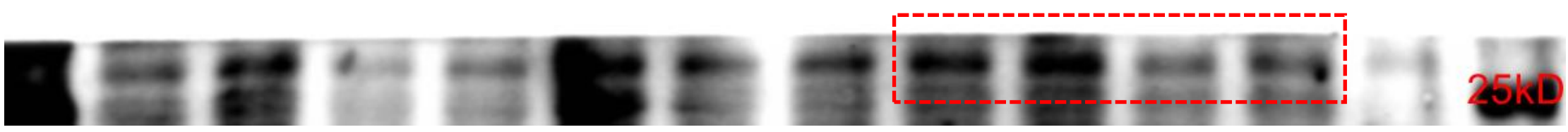

Sample 3 β-Tubulin (for Sdhb)

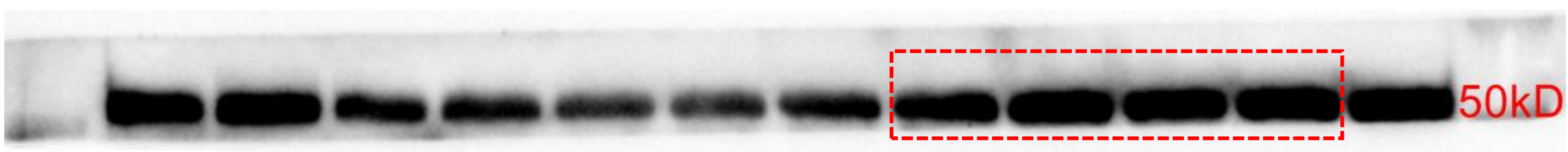

Sample 3 Mtco2

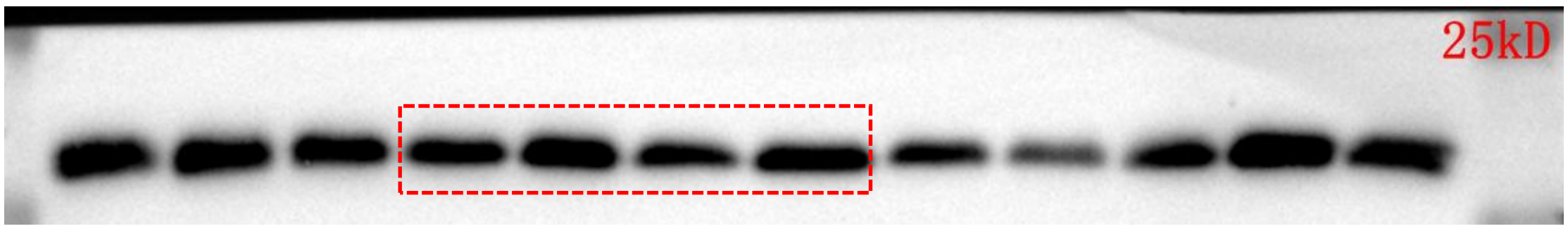

Sample 3 β-Tubulin (for Mtco2)

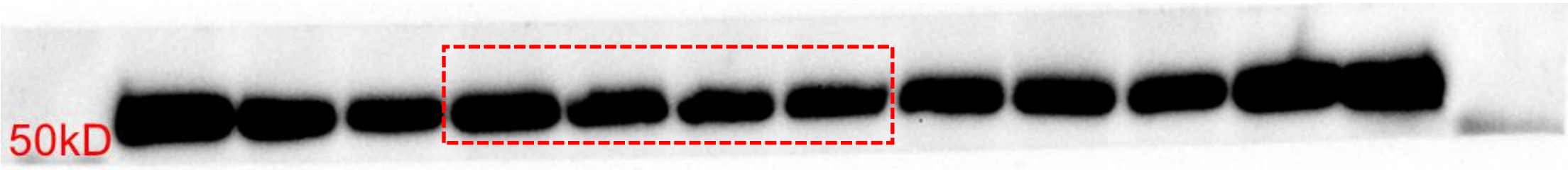

Atpaf1

Sample 1. Atpaf1

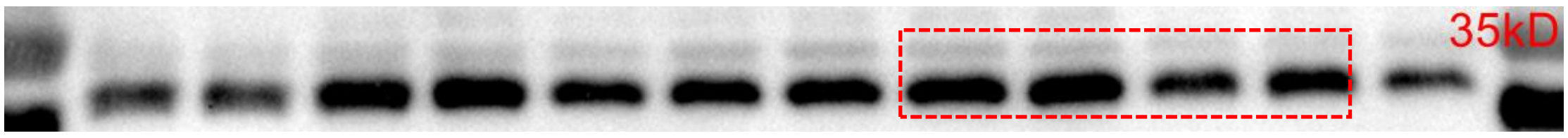

Sample 1. β-Tubulin

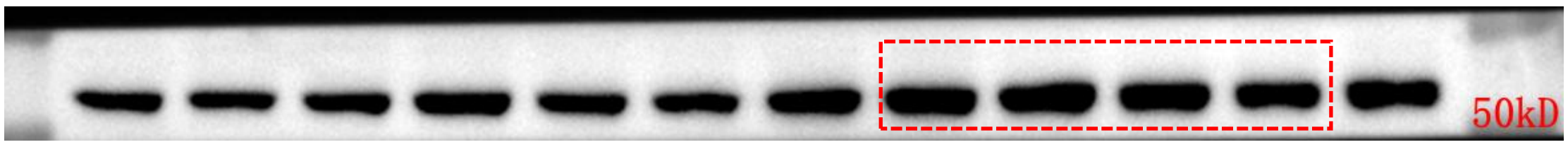

Sample 2. Atpaf1

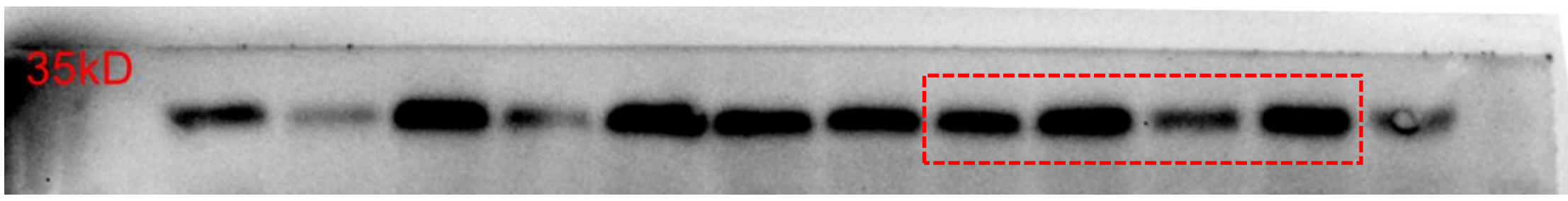

Sample 2. β-Tubulin

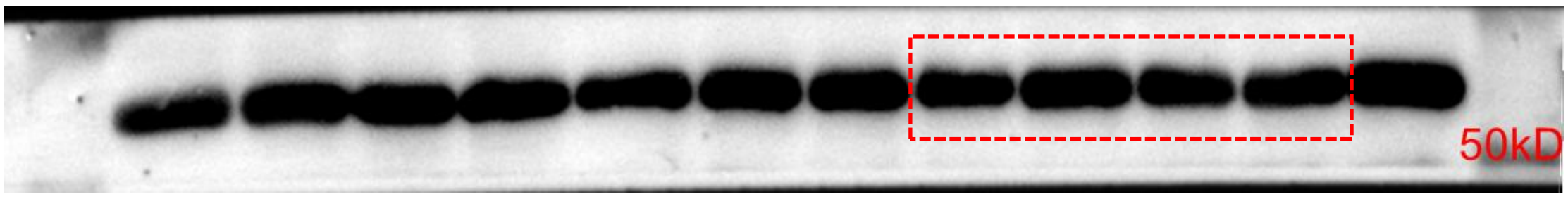

Sample 3. Atpaf1

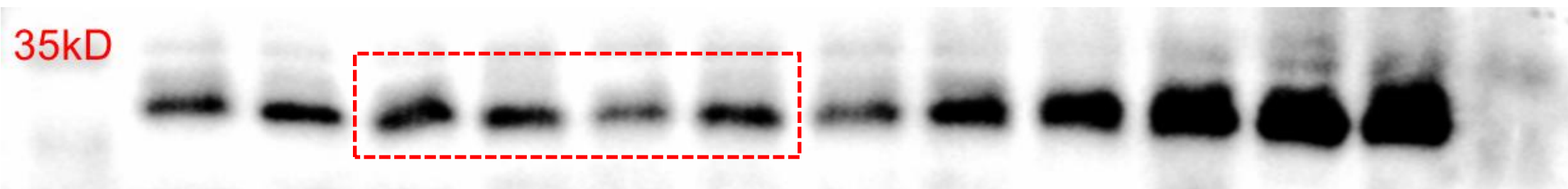

Sample 3. β-Tubulin

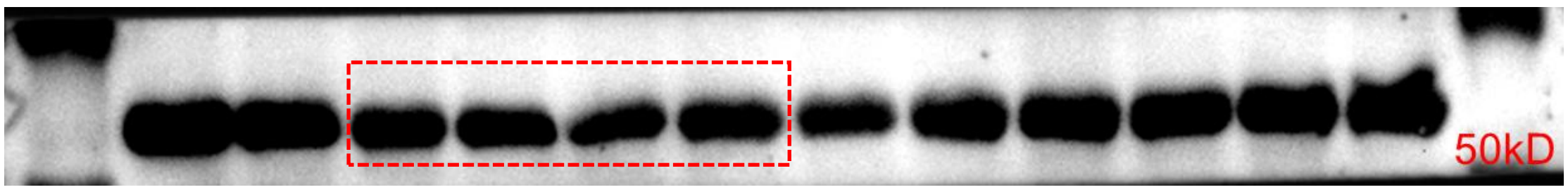

Il-10

Sample 1. Il-10

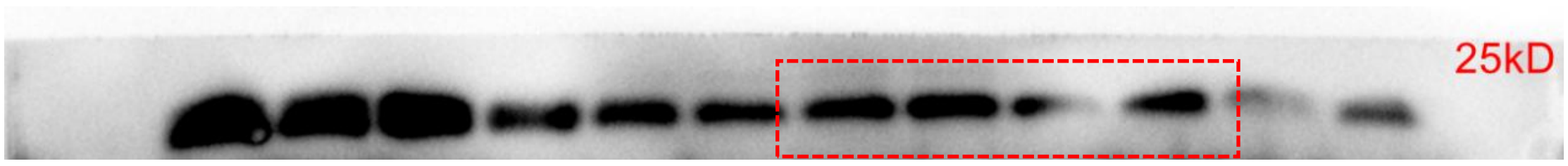

Sample 1. β-actin

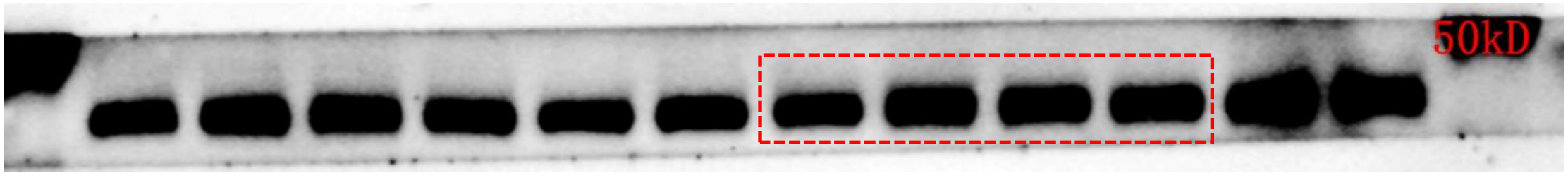

Sample 2+Sample 3. Il-10

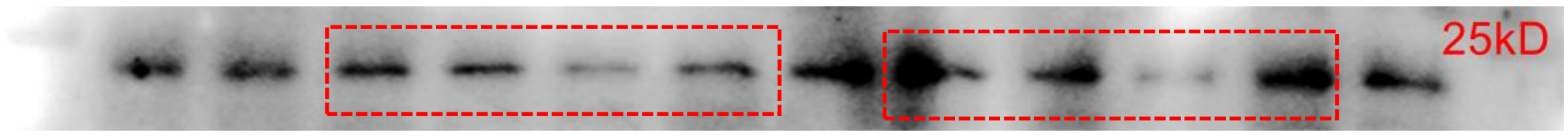

Sample 2+Sample 3.β-actin

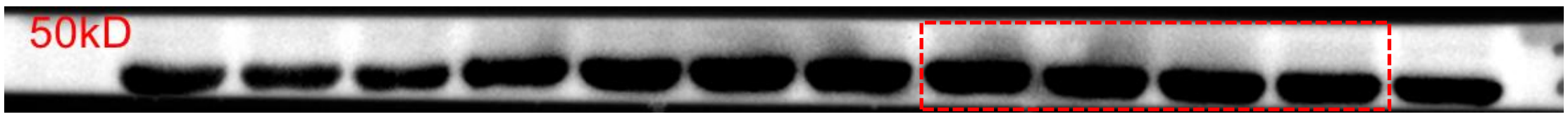

e. Inos+Cleaved Il-1β

Sample 1 Inos

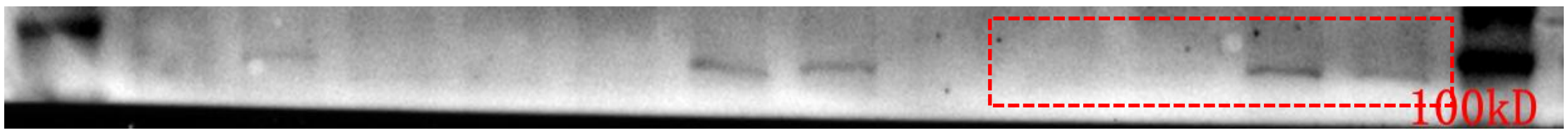

Sample 1 Cleaved Il-1β

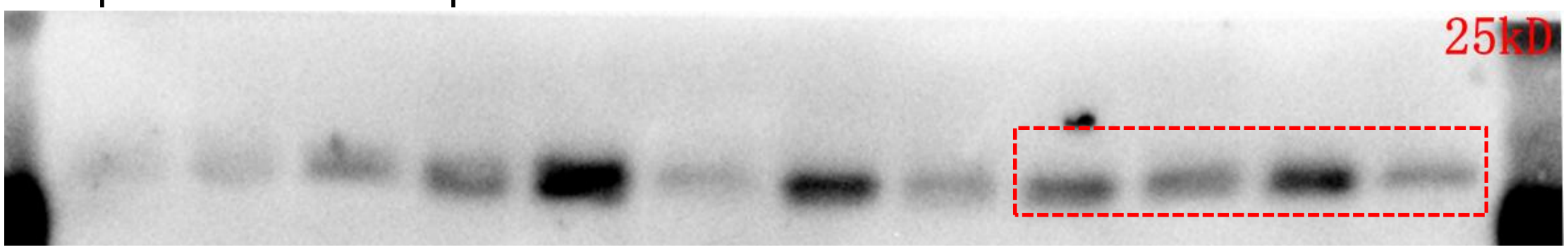

Sample 1 Gapdh

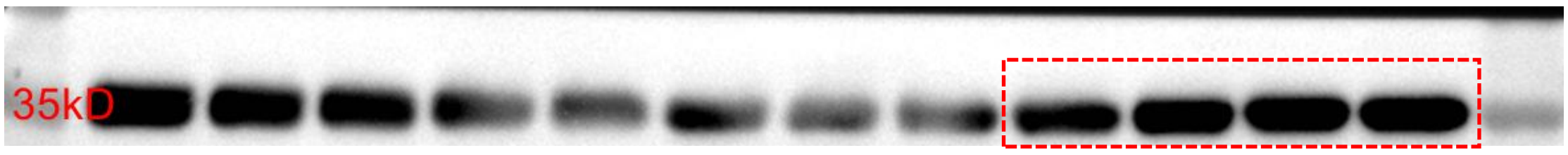

Sample 2 Inos

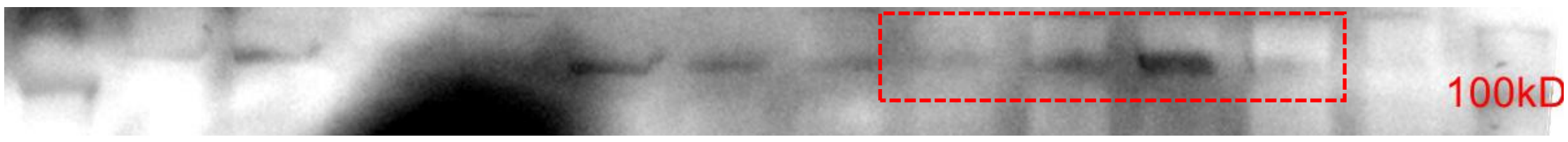

Sample 2 Gapdh

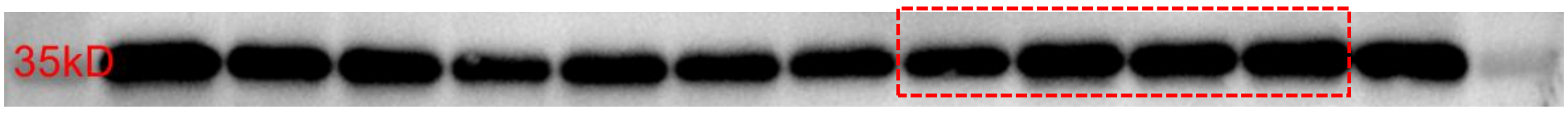

Sample 3 Inos

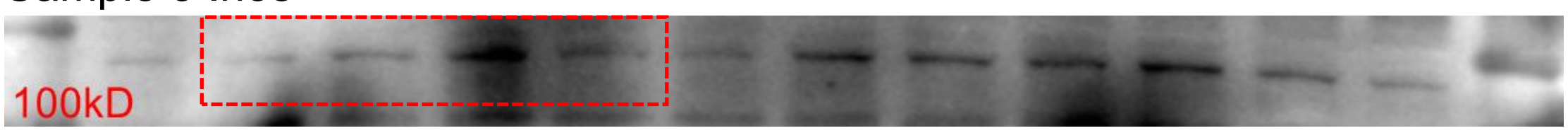

Sample 3 Gapdh

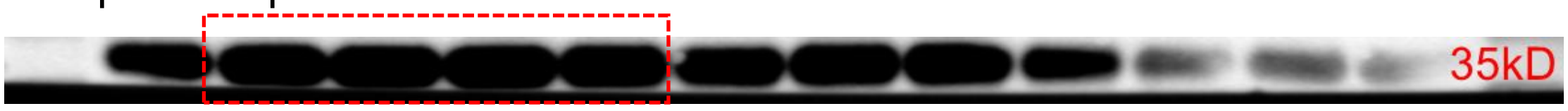

Sample 2 Cleaved Il-1β

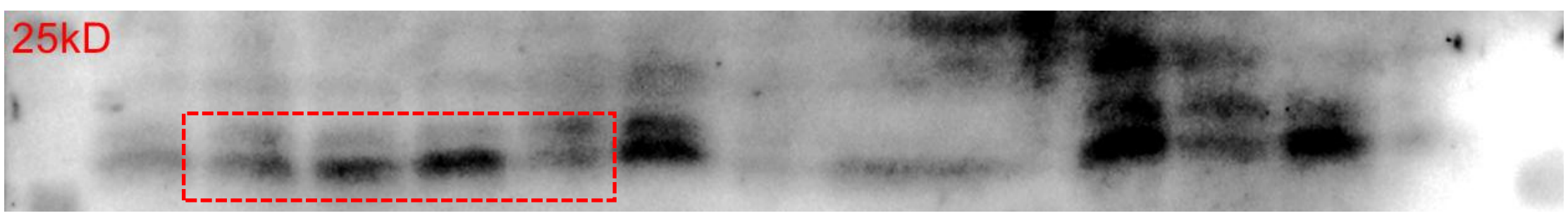

Sample 2 Gapdh

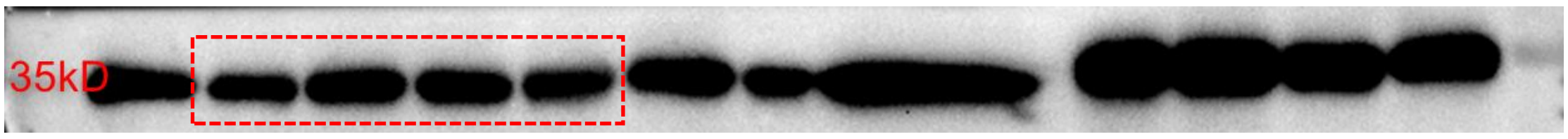

Sample 3 Cleaved Il-1β

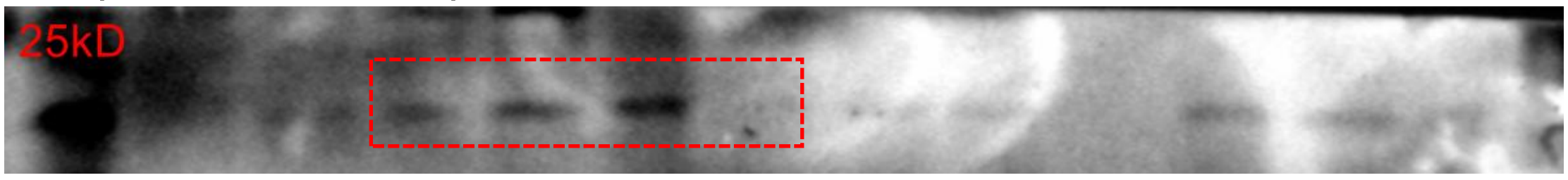

Sample 3 Gapdh

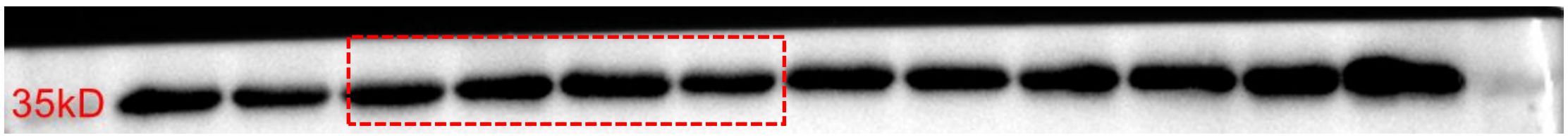

Caspase-1/Cleaved Caspase-1

Sample 1. Pro Caspase-1

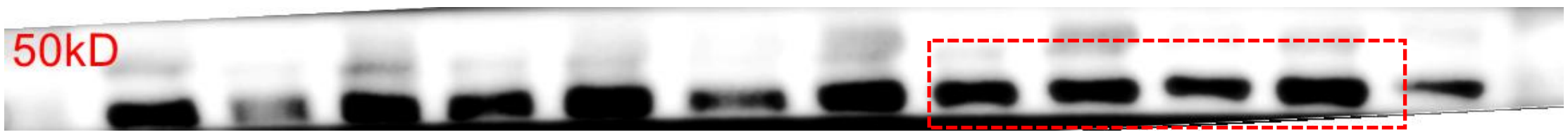

Sample 1. Cleaved Caspase-1

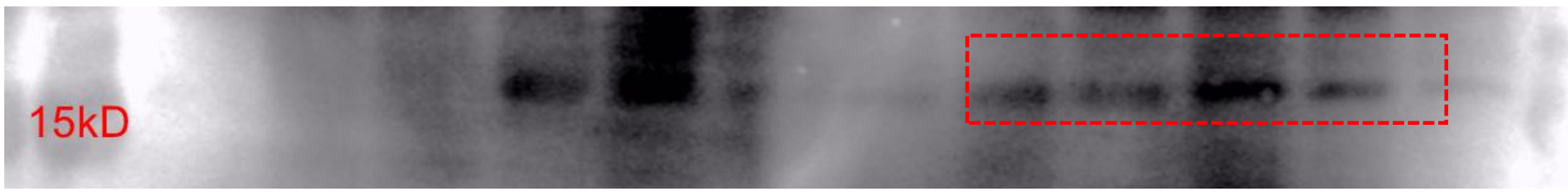

Sample 2+Sample 3. Pro Caspase-1

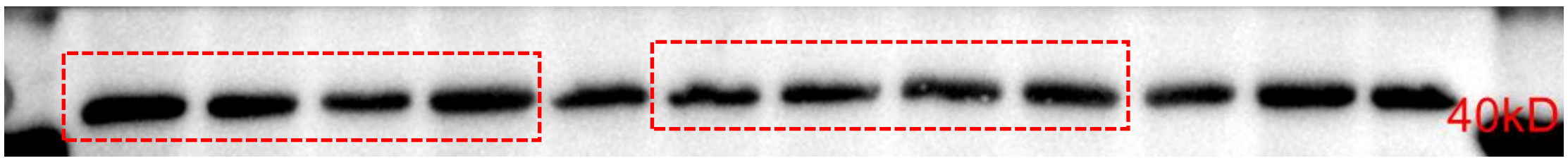

Sample 2+Sample 3. Cleaved Caspase-1

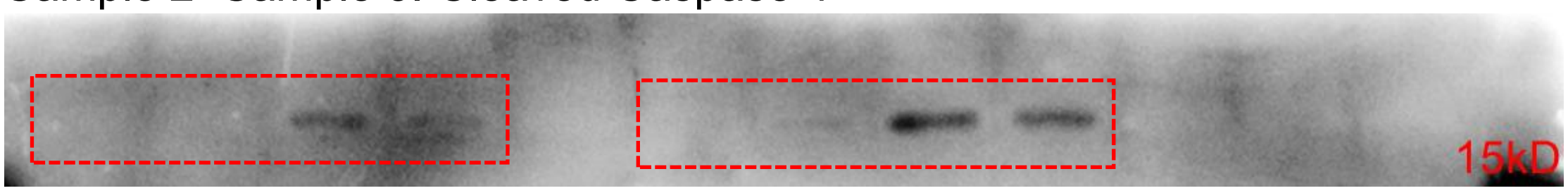

Supplement: Supplementary file 1 [file 40035_2026_583_MOESM3_ESM.pdf]
