# Supplementary material for: Exercise promotes lipophagy and mitigates cognitive impairment in Alzheimer’s disease through downregulating microglial TMEM9
Source: Transl Neurodegener. 2026 Oct 19;15:56. doi: 10.1186/s40035-026-00583-z (PMC13679003; doi:10.1186/s40035-026-00583-z)
Supplement: Supplementary file 2 [file 40035_2026_583_MOESM1_ESM.docx]

**Supplementary materials of Exercise promotes lipophagy and mitigates cognitive impairment in Alzheimer's disease through downregulating microglial TMEM9**

**Supplementary Figures and Figure legends**

**Fig. S1. Physical exercise (PE) enhanced microglial lipophagy in 5xFAD mice.** **a** Co-Immunofluorescent staining and analysis of the Perilipin 2 positive LDs in microglia across WT, control (Ctrl)- and PE-5xFAD groups, *n* = 4 male mice per experimental group, aged 8 months (100 x oil objective, 3 x magnification) . **b** Co-Immunofluorescent staining of Perilipin 2 positive LDs and NeuN or GFAP in 5xFAD mice. **c** Co-Immunofluorescent staining and analysis of CD36 expressions in microglia across WT, control (Ctrl)- and PE-5xFAD groups, *n* = 4 male mice per experimental group, aged 8 months (100 x oil objective, 3 x magnification). **d** Western blotting analysis of ATGL, HSL in cortex and hippocampus across WT, Ctrl-5xFAD and PE-5xFAD group, *n* = 3 male mice per experimental group, aged 8 months. Data are expressed as the mean ± SD, statistical significance was assessed using one-way analysis of variance (ANOVA), followed by Tukey’s multiple comparisons test.**P*<0.05; ***P*<0.01; ****P*<0.001; *****P*<0.0001.

**Fig. S2. Verification of adeno-associated lentivirus transfection.** **a** Immunofluorescent staining of GFP expressions in microglia between RNAi-*Tmem9* and PE + OE-*Tmem9* groups (20 x objective, 5 x magnification). **b-c** Western blotting analysis of TMEM9 in cortex and hippocampus across WT, Ctrl-5xFAD, PE-5xFAD, RNAi-*Tmem9* and PE+OE-*Tmem9* groups, *n* = 4 male mice per group, aged 8 months. Data are expressed as the mean ± SD, statistical significance was assessed using one-way analysis of variance (ANOVA), followed by Tukey’s multiple comparisons test. **P*<0.05; ***P*<0.01; ****P*<0.001; *****P*<0.0001.

**Fig. S3. Physical exercise enhanced microglial lipophagy in 5xFAD mice by down-regulating microglia TMEM9.** **a** Immunofluorescent staining of LC3 and Bodipy in microglia between WT, Ctrl-5xFAD, PE-5xFAD, RNAi-*Tmem9* and PE+OE-*Tmem9* groups, left pannel: 20 x objective, 5 x magnification; white dotted boxes label the magnified regions displayed in the right panel. Right panel: Higher-magnification views of the areas boxed in the left panel. **b** Bodipy fluorescent intensity and bodipy co-localized with LC3 in microglia across WT, Ctrl-5xFAD, PE-5xFAD, RNAi-*Tmem9* and PE+OE-*Tmem9* groups. *n* = 4 male mice per group, 2 field per mice, aged 8 months. Data are expressed as the mean ± SD, statistical significance was assessed using one-way analysis of variance (ANOVA), followed by Tukey’s multiple comparisons test. **P*<0.05; ***P*<0.01; ****P*<0.001; *****P*<0.0001.

**Fig. S4. Transfection of lentivirus did not affect the LDs formation induced by Oleic Acid (OA) in BV2 cells.** **a** Experimental design for ShRNA-*Tmem9* and OE-*Tmem9* lentiviral transfection, as well as OA treatment, in BV2 Cells. **b** Western blotting analysis of TMEM9 expressions across Scramble, ShRNA-*Tmem9* , Negative Control (NC), and OE-*Tmem9* groups, *n*=3 wells per groups. **c-d** Immunofluorescent staining and analysis of the Perilipin 2+ LDs induced by OA treatment across Blank, Scramble, ShRNA-*Tmem9*, NC and OE-*Tmem9* groups (20 x objective, 5 x magnification), *n* = 4 wells per groups. *Tmem9* knockdown and overexpression were achieved via lentivirus transfection. Data are expressed as the mean ± SD, statistical significance was assessed using one-way analysis of variance (ANOVA), followed by Tukey’s multiple comparisons test. **P*<0.05; ***P*<0.01; ****P*<0.001; *****P*<0.0001.

**Fig. S5. Knockdown of *Tmem9* promoted autophagy, increased mitochondrial proteins and decreased pro-inflammatory cytokines induced by oAβ in BV2 Cells under glucose-insufficiency conditions, whereas overexpression of *Tmem9* exerted opposing effects. a-b** Western blotting analysis for ATG5, P62, and LC3II/LC3I in Scramble, ShRNA-*Tmem9*, NC and OE-*Tmem9* groups after treatment with OA and oAβ (*n* = 3 well per group). **c** Quantitative Real-time PCR (qPCR) validation of transfection efficiency for shRNA-*Tmem9* and OE-*Tmem9* plasmids, statistical significance was assessed using independent-samples t test, *n* = 3 well per group. **d-e** Immunofluorescent analysis for autolysosome/autophagosome ratio (100 x oil objective, 3 x magnification) (*n* = 3 well per group, 2 field per well). **f-g** Western blotting analysis of mitochondrial complex I-V-related proteins (NDUFB10, SDHB, UQCRC2, MTCO2, and ATPAF1) and the anti-inflammatory cytokine IL-10 in the Scramble, shRNA-*Tmem9*, NC, and OE-*Tmem9* groups, *n* = 3 well per group. **h-i** Western blottings analysis of proteins related to pro-inflammatory cytokines, including NLRP3, iNOS and cleaved IL-1β among Scramble, ShRNA-*Tmem9*, NC and OE-*Tmem9* groups, *n* = 3 well per group. For western blotting, *Tmem9* knockdown and overexpression were achieved via lentiviral transfection. For immunofluorescent staining, *Tmem9* knockdown and overexpression were achieved via plasmid transfection. Data are expressed as the mean±SD, statistical significance was assessed using one-way analysis of variance (ANOVA), followed by Tukey’s multiple comparisons test. **P*<0.05; ***P*<0.01; ****P*<0.001; *****P*<0.0001.

**Fig. S6. Knockdown of *Tmem9* promoted autophagy, increased mitochondrial protein and decreased pro-inflammatory cytokines induced by oAβ in BV2 Cells under glucose-insufficiency conditions, whereas chloroquine treatment diminished its effects. a** Experimental design for ShRNA-*Tmem9* lentiviral/plasmid transfection in BV2 cells, followed by treatment with OA, oAβ with DMSO/CQ. **b-d** Western blotting analysis of ATG5, P62, LC3II/LC3I and immunofluorescent analysis of autolysosome/autophagosome ratio among Scramble and ShRNA-*Tmem9* groups treated with OA+oAβ with DMSO or CQ. **e-f** Western blotting analysis the proteins related to mitochondrial complex I-V including UQCRC2, SDHB, ATPAF1, NDUFB10 and MTCO2 across Scramble, ShRNA-*Tmem9*, NC and OE-*Tmem9* groups treated with OA+oAβ with DMSO or CQ. **g-h** Western blotting analysis of proteins related to pro-inflammatory cytokines, including NLRP3, Cleaved IL-1β, and the anti- inflammatory cytokine IL-10 among Scramble and ShRNA-*Tmem9* groups treated with OA+oAβ with DMSO or CQ. For western blotting, *Tmem9* knockdown was achieved via lentivirus transfection; for immunofluorescent staining, *Tmem9* knockdown was achieved via plasmid transfection. Data are expressed as the mean±SD, statistical significance was assessed using one-way analysis of variance (ANOVA), followed by Tukey’s multiple comparisons test. **P*<0.05; ***P*<0.01; ****P*<0.001; *****P*<0.0001.

**Fig. S7. Knockdown of *Tmem9* promotes LKB1 translocation to lysosome *in vitro*. a** Experimental design for shRNA-*Tmem9* lentiviral/Plasmid transfection, as well as OA and oAβ treatment in BV2 cells. **b** Immunofluorescence co-localization analysis of LAMP1 and LKB1 in Scramble and ShRNA-*Tmem9* cells, *n*=3 wll per group, 2 field per well. **c** Western blotting analysis the total LKB1 and lysosmal LKB1 proteins in Scramble and ShRNA-*Tmem9* cells, *n* = 3 wells per group. For western blotting, *Tmem9* knockdown was achieved via lentivirus transfection; for immunofluorescent staining, *Tmem9* knockdown was achieved via plasmid transfection. Data are expressed as the mean±SD, statistical significance was assessed using independent-samples t test. **P*<0.05; ***P*<0.01; ****P*<0.001; *****P*<0.0001.

**Fig. S8. Concanamycin A (Con A) treatment reactivated AMPKα signaling, maintained mitochondrial homeostasis, and reduced pro-inflammatory cytokines in BV2 cells with *Tmem9* overexpression following oleic acid (OA) and oAβ incubation. a-b** Western blotting analysis of LKB1, p-AMPKα/AMPKα, NLRP3, and CPT1A in NC and OE-*Tmem9* cells treated with DMSO or Con A. **c-d** Western blots analysis of SDHB, MTCO2, ATPAF1 and IL-10 in NC and OE-*Tmem9* cells treated with DMSO or Con A. **e-f** Western blotting analysis of iNOS, IL-1β, cleaved Caspase-1 in NC and OE-*Tmem9* cells treated with DMSO or Con A. *Tmem9* overexpression was implemented through lentiviral transfection. *n* = 3 wells per group. The results are expressed as the mean ± SD, statistical significance was assessed using one-way analysis of variance (ANOVA), followed by Tukey’s multiple comparisons test. **P*<0.05; ***P*<0.01; ****P*<0.001; *****P*<0.0001.

**Fig. S9. AAV injection itself had no effect on motor capacity in behavioral tests. a** Travel path tracings of mice during the novel object recognition (NOR) test. Quantitative analysis of distance traveled and novel object recognition index in the NOR test: comparison between control vs. AAV-Scramble, and PE vs. PE+AAV-NC groups, *n* =8 male mice per group, at age of 8 months. **b** Travel path tracings of mice during open field test (OFT). Quantitative analysis of distance traveled and time spent in the central area during OFT, comparison between control vs. AAV-Scramble, and PE vs. PE+AAV-NC groups, *n* =8 male mice per group, at age of 8 months. **c** Spatial learning trajectories during water maze training and probe trial in the Morris water maze. Quantitative analysis of velocity and latency to the platform on day 5 of water maze training, as well as swimming velocity and platform crossings during the probe trial, *n* =8 male mice per group, at age of 8 months. Data are expressed as the mean±SD, statistical significance was assessed using one-way analysis of variance (ANOVA), followed by Tukey’s multiple comparisons test. **P*<0.05; ***P*<0.01; ****P*<0.001; *****P*<0.0001.

**Fig. S10. CSF TMEM9 levels are negatively correlated with SUVR on ^18^F-FDG PET-CT. a** ^18^F-AV45 PET-CT montage (Scale bar = 5cm) and SUVR analysis of amyloid-β (Aβ) deposition betweeen healthy control individuals (*n*=9) and AD patients (*n*=9), statistical significance was assessed using independent-samples t test. **b** ^18^F-FDG PET-CT montage (Scale bar = 5cm) and SUVmax analysis of glucose metabolism in healthy control individuals (*n*=4) and AD patients (*n*=8), statistical significance was assessed using independent-samples t test. Correlation between average standardized uptake value ratio (SUVR) of ^18^F-AV45 and maximum standardized uptake value (SUVmax) of ^18^F-FDG in AD (*n*=5). **c** Correlation of CSF p-tau181 levels with regional brain SUVR on ^18^F-AV45 PET-CT (*n*=4). **d** Correlation of CSF TMEM9 levels with regional brain SUVmax on ^18^F-FDG PET-CT, the relevant analysis was conducted using Pearson correlation analysis (*n*=4). The results are expressed as the mean ± SD. **P*<0.05; ***P*<0.01; ****P*<0.001; *****P*<0.0001.

**Supplementary Tables**

**Table S1. Antibodies for IF and WB**

| **Host** | **Antibody** | **Manufacturer** | **Catalog No.** | **Dilution** |
| --- | --- | --- | --- | --- |
| Rabbit | Perilipin-2 Antibody | Servicebio | GB115593 | 1: 400 for IF and 1:1000 for WB |
| Rabbit | TMEM9 Antibody | Affinity Biosciences | DF12540 | 1: 400 for IF and 1:1000 for WB |
| Mouse | Iba1 Antibody | Servicebio | GB12105 | 1: 400 for IF |
| Mouse | NeuN Antibody | Sigma-Aldrich | MAB377 | 1: 400 for IF |
| Mouse | GFAP Antibody | Servicebio | GB12096 | 1: 400 for IF |
| Rabbit | CD36 Antibody | Servicebio | GB112562 | 1: 400 for IF |
| Rabbit | Iba1 Antibody | WAKO | 019-19741 | 1: 400 for IF |
| Mouse | LC3b Antibody | ABclonal | A17424 | 1: 400 for IF |
| Rat | LAMP1 Antibody | Abcam | AB24871 | 1: 400 for IF |
| Rabbit | LKB1Antibody | Affinity Biosciences | AF6453 | 1: 400 for IF, 1:1000 for WB |
| Mouse | AXIN Antibody | Proteintech | 68093-1-IG | 1: 400 for IF, 1:1000 for WB |
| Mouse | Aβ1-42 Antibody | Biolegend | 805501 | 1: 400 for IF, 1:1000 for WB |
| Rabbit | LC3 Antibody | Cell signaling Technology | 12741S | 1:1000 for WB |
| Rabbit | APG5L/ATG5 Antibody | Affinity Biosciences | DF6010 | 1:1000 for WB |
| Rabbit | β-actin Antibody | Affinity Biosciences | AF7018 | 1:1000 for WB |
| Rabbit | β-tubulin Antibody | Affinity Biosciences | AF7011 | 1:1000 for WB |
| Rabbit | GAPDH Antibody | Affinity Biosciences | AF7021 | 1:1000 for WB |
| Rabbit | LIPA (LAL) Antibody | Proteintech | 12956-1-AP | 1:1000 for WB |
| Rabbit | ATGL Antibody | Affinity Biosciences | DF7756 | 1:1000 for WB |
| Rabbit | HSL Antibody | Affinity Biosciences | AF6403 | 1:1000 for WB |
| Rabbit | NLRP3 Antibody | Affinity Biosciences | DF15549 | 1:1000 for WB |
| Rabbit | Caspase-1 Antibody | Affinity Biosciences | AF5418 | 1:1000 for WB |
| Rabbit | Cleaved-Caspase 1 (Asp296), p20 Antibody | Affinity Biosciences | AF4005 | 1:1000 for WB |
| Rabbit | IL18 Antibody | Affinity Biosciences | DF6252 | 1:1000 for WB |
| Rabbit | IL10 Antibody | Abcolonal | A20723 | 1:1000 for WB |
| Rabbit | CPT1A Antibody | Affinity Biosciences | DF12004 | 1:1000 for WB |
| Rabbit | NDUFB10 Antibody | ABclonal | A3982 | 1:1000 for WB |
| Rabbit | SDHB Antibody | Servicebio | GB111344 | 1:1000 for WB |
| Rabbit | UQCRC2 Antibody | Affinity Biosciences | DF12339 | 1:1000 for WB |
| Rabbit | MTCO2 Antibody | Affinity Biosciences | DF7867 | 1:1000 for WB |
| Rabbit | ATPAF1 Antibody | Proteintech | 18016-1-AP | 1:1000 for WB |
| Rabbit | AMPK alpha Antibody | Affinity Biosciences | AF6423 | 1:1000 for WB |
| Rabbit | Phospho-AMPK alpha (Thr172) Antibody | Affinity Biosciences | AF3423 | 1:1000 for WB |
| Rabbit | iNOS Antibody | Affinity Biosciences | AF0199 | 1:1000 for WB |
| Rabbit | IL-1β Antibody | Servicebio | GB11113-100 | 1:1000 for WB |
| Rabbit | Cleaved-IL-1 beta (Asp116) Antibody | Affinity Biosciences | AF4006 | 1:1000 for WB |
| Rabbit | ATP6AP1 Antibody | Affinity Biosciences | DF6341 | 1:1000 for WB |
| Rabbit | SQSTM1/p62 Antibody | Affinity Biosciences | AF5384 | 1:1000 for WB |
| Mouse | Tau Antibody | Proteintech | 66499-1-Ig | 1:1000 for WB |
| Rabbit | [Phospho-Tau (Thr217) antibody](https://www.ptgcn.com/products/Phospho-TAU-Thr217-Antibody-31138-1-AP.htm) | Proteintech | 31138-1-AP | 1:1000 for WB |
| / | Anti-rabbit lgG, HRP-linked Antibody | Cell Signaling Technology | 70745 | 1:5000 for WB |
| / | Anti-mouse IgG, HRP-linked Antibody | Cell Signaling Technology | 7076S | 1:5000 for WB |
| / | Anti-mouse IgG (H+L), F(ab')2 Fragment (Alexa Fluor® 647 Conjugate) | Cell Signaling Technology | 4410S | 1: 400 for IF |
| / | Anti-mouse IgG (H+L), F(ab')2 Fragment (Alexa Fluor® 488 Conjugate) | Cell Signaling Technology | 4408S | 1: 400 for IF |
| / | Anti-rabbit IgG (H+L), F(ab')2 Fragment (Alexa Fluor® 555 Conjugate) | Cell Signaling Technology | 4413S | 1: 400 for IF |

**Table S2. Characteristics of control and AD patients cohort**

| **Group** | **Sex** | **Age** | **Plasma** | **CSF** | **^18^F-AV45 PET-CT** | **^18^F-FDG PET-CT** | **MMSE Score** | **MoCA-B Score** |
| --- | --- | --- | --- | --- | --- | --- | --- | --- |
| Control 1 | Male | 69 | Yes | No | No | No | / | 26 |
| Control 2 | Female | 41 | Yes | No | No | No | 25 | 26 |
| Control 3 | Female | 62 | Yes | No | No | No | 28 | 27 |
| Control 4 | Male | 72 | Yes | No | No | No | / | 18 |
| Control 5 | Female | 70 | Yes | No | No | No | / | 26 |
| Control 6 | Female | 62 | Yes | No | No | No | / | / |
| Control 7 | Male | 69 | Yes | No | No | No | 27 | 20 |
| Control 8 | Male | 68 | Yes | No | No | No | 29 | 26 |
| Control 9 | Female | 60 | Yes | No | No | No | / | 23 |
| Control 10 | Male | 33 | Yes | No | No | No | 29 | 30 |
| Control 11 | Female | 76 | Yes | No | No | No | / | / |
| Control 12 | Female | 55 | Yes | No | No | No | 30 | 24 |
| Control 13 | Female | 75 | Yes | No | No | No | 14 | 11 |
| Control 14 | Male | 64 | Yes | No | No | No | / | 26 |
| Control 15 | Female | 60 | Yes | No | No | No | / | 25 |
| Control 16 | Male | 60 | Yes | Yes | No | No | / | / |
| Control 17 | Female | 69 | Yes | No | No | No | / | 24 |
| Control 18 | Male | 71 | Yes | No | No | No | / | 29 |
| Control 19 | Female | 72 | Yes | No | No | No | / | 27 |
| Control 20 | Male | 79 | Yes | No | No | No | / | 23 |
| Control 21 | Female | 67 | Yes | No | No | No | / | 26 |
| Control 22 | Female | 71 | Yes | No | No | No | / | 23 |
| Control 23 | Female | 62 | Yes | No | No | No | / | 23 |
| Control 24 | Male | 67 | Yes | No | No | No | / | / |
| Control 25 | Male | 72 | Yes | No | No | No | / | 17 |
| Control 26 | Male | 63 | Yes | No | No | No | / | 23 |
| Control 27 | Male | 81 | Yes | No | No | No | / | 23 |
| Control 28 | Female | 55 | Yes | No | No | No | / | 25 |
| Control 29 | Male | 61 | No | Yes | No | No | / | / |
| Control 30 | Male | 57 | No | Yes | No | No | / | / |
| Control 31 | Female | 61 | No | Yes | No | No | / | 29 |
| Control 32 | Female | 70 | No | Yes | No | No | / | / |
| Control 33 | Male | 69 | No | Yes | No | No | 20 | 17 |
| Control 34 | Female | 61 | No | No | Yes | No | / | / |
| Control 35 | Female | 68 | No | No | Yes | No | / | / |
| Control 36 | Male | 67 | No | No | Yes | Yes | / | / |
| Control 37 | Female | 55 | No | No | Yes | Yes | / | / |
| Control 38 | Female | 70 | No | No | Yes | No | / | / |
| Control 39 | Male | 80 | No | No | Yes | No | / | / |
| Control 40 | Female | 62 | No | No | Yes | Yes | / | / |
| Control 41 | Male | 88 | No | No | Yes | Yes | / | / |
| Control 42 | Female | 57 | No | No | Yes | No | / | / |
| AD1 | Female | 70 | Yes | No | No | No | / | / |
| AD2 | Female | 87 | Yes | No | No | No | 18 | 11 |
| AD3 | Male | 61 | Yes | No | No | No | 25 | 19 |
| AD4 | Female | 53 | Yes | Yes | No | No | 27 | 23 |
| AD5 | Female | 68 | Yes | Yes | No | No | 3 | / |
| AD6 | Male | 73 | Yes | Yes | Yes | Yes | / | / |
| AD7 | Female | 66 | Yes | No | Yes | Yes | 8 | / |
| AD8 | Male | 67 | Yes | No | No | No | 6 | / |
| AD9 | Male | 64 | Yes | No | No | No | 29 | 25 |
| AD10 | Male | 60 | Yes | No | Yes | No | 29 | 22 |
| AD11 | Male | 76 | Yes | No | No | No | 29 | 23 |
| AD12 | Female | 75 | Yes | No | No | No | 22 | 19 |
| AD13 | Female | 86 | Yes | No | No | No | 9 | / |
| AD14 | Female | 85 | Yes | No | No | No | 19 | 19 |
| AD15 | Female | 84 | Yes | Yes | No | Yes | / | / |
| AD16 | Female | 74 | Yes | No | Yes | Yes | 9 | 8 |
| AD17 | Male | 73 | Yes | No | No | No | / | / |
| AD18 | Male | 92 | Yes | No | No | No | / | / |
| AD19 | Female | 72 | Yes | No | Yes | No | 11 | / |
| AD20 | Female | 68 | Yes | No | Yes | No | 30 | 29 |
| AD21 | Male | 75 | Yes | No | No | No | 17 | 14 |
| AD22 | Male | 73 | Yes | No | No | No | 27 | 26 |
| AD23 | Female | 69 | Yes | No | No | No | 6 | / |
| AD24 | Male | 70 | Yes | No | No | No | 28 | 25 |
| AD25 | Male | 74 | Yes | Yes | No | No | / | / |
| AD26 | Female | 58 | Yes | No | No | No | / | / |
| AD27 | Female | 57 | No | Yes | No | No | 29 | 26 |
| AD28 | Female | 52 | No | Yes | Yes | No | / | 23 |
| AD29 | Female | 64 | No | Yes | No | No | 17 | / |
| AD30 | Male | 83 | No | Yes | No | No | 9 | 6 |
| AD31 | Male | 75 | No | Yes | Yes | Yes | / | 21 |
| AD32 | Female | 75 | No | Yes | No | No | 20 | 13 |
| AD33 | Male | 75 | No | Yes | No | No | / | / |
| AD34 | Female | 40 | No | Yes | Yes | Yes | / | 23 |
| AD35 | Female | 50 | No | Yes | No | No | 29 | 22 |
| AD36 | Male | 76 | No | Yes | No | No | 20 | 17 |
| AD37 | Male | 66 | No | Yes | No | No | / | / |
| AD38 | Female | 68 | No | No | No | Yes | / | / |
| AD39 | Female | 76 | No | No | No | Yes | / | / |

**Table S3. Correlation analyses between biomarkers and ^18^F-AV45 PET-CT**

| **^18^F-AV45 PET-CT** | **TMEM9 in plasma**  **(ng/mL)**  **(*n* = 6)** | **ATP6AP1 in plasma**  **(ng/mL)**  **(*n* = 6)** | **p-tau217 in plasma**  **(ng/L)**  **(*n* = 5)** | **TMEM9 in CSF**  **(ng/mL)**  **(*n* = 4)** | **ATP6AP1 in CSF**  **(ng/mL)**  **(*n* = 4)** | **p-tau181 in CSF**  **(ng/L)**  **(*n* = 3)** |
| --- | --- | --- | --- | --- | --- | --- |
| SUVR of left frontal cortex | *R* = -0.042,  *P* = 0.937 | *R* = 0.605,  *P* = 0.203 | *R*= -0.062,  *P* = 0.921 | *R* = -0.167,  *P* = 0.833 | *R* = -0.404,  *P* = 0.596 | *R* = 0.895,  *P* = 0.105 |
| SUVR of right frontal cortex | *R* = -0.121,  *P* = 0.819 | *R* = 0.300,  *P* = 0.564 | *R*= -0.584,  *P* = 0.302 | *R* = -0.124,  *P* = 0.876 | *R* = -0.374,  *P* = 0.626 | *R* = 0.943,  *P* = 0.057 |
| SUVR of left intraparietal cortex | *R* = -0.471,  *P* = 0.345 | *R* = 0.388,  *P* = 0.447 | *R* = 0.583, *P* = 0.302 | *R* = -0.080,  *P* = 0.920 | *R* = -0.338,  *P* = 0.662 | *R* = 0.963,  *P* = 0.037 |
| SUVR of right intraparietal cortex | *R* = -0.471,  *P* = 0.345 | *R* = 0.335,  *P* = 0.516 | *R* = 0.674, *P* = 0.212 | *R* = -0.279,  *P* = 0.721 | *R* = -0.521,  *P* = 0.479 | *R* = 0.981,  *P* = 0.019 |
| SUVR of left temporal cortex | *R* = -0.531,  *P* = 0.279 | *R* = -0.032,  *P* = 0.953 | *R* = 0.070, *P* = 0.911 | *R* = 0.280,  *P* = 0.720 | *R* = 0.034,  *P* = 0.966 | *R* = 0.771,  *P* = 0.229 |
| SUVR of right temporal cortex | *R* = -0.465,  *P* = 0.353 | *R* = 0.443,  *P* = 0.380 | *R* = 0.796, *P* = 0.107 | *R* = -0.268,  *P* = 0.732 | *R* = -0.511,  *P* = 0.489 | *R* = 0.999,  *P* = 0.001 |
| SUVR of left occipital cortex | *R* = -0.463,  *P* = 0.356 | *R* = 0.501,  *P* = 0.312 | *R*= -0.637,  *P* = 0.248 | *R* = 0.294,  *P* = 0.706 | *R* = 0.037,  *P* = 0.963 | *R* = 0.824,  *P* = 0.176 |
| SUVR of right occipital cortex | *R* = 0.111,  *P* = 0.834 | *R* = 0.187,  *P* = 0.723 | *R*= -0.619, *P* = 0.266 | *R* = 0.135,  *P* = 0.865 | *R* = -0.129,  *P* = 0.871 | *R* = 0.919,  *P* = 0.081 |

Footnote: *R* values represent Spearman’s correlation coefficients. *P* values are two‑sided, and **P* <0.05 indicates statistical significance. SUVR = standardized uptake value ratio; CSF = cerebrospinal fluid; TMEM9 = transmembrane protein 9; ATP6AP1 = ATPase H⁺transporting accessory protein 1; p-tau217 = phosphorylated tau at threonine 217; p-tau181 = phosphorylated tau at threonine 181.

**Table S4. Correlation analyses between biomarkers and ^18^F-FDG PET-CT**

| **^18^F-FDG PET-CT** | **TMEM9 in plasma**  **(ng/mL)**  **（*n* = 4）** | **ATP6AP1 in plasma**  **(ng/mL)**  **（*n* = 4）** | **p-tau217 in plasma**  **(ng/L)**  **（*n* = 3）** | **TMEM9 in CSF**  **(ng/mL)**  **（*n* = 4）** | **ATP6AP1 in CSF**  **(ng/mL)**  **（*n* = 4）** | **p-tau181 in CSF**  **(ng/L)**  **（*n* = 4）** |
| --- | --- | --- | --- | --- | --- | --- |
| SUVmax of left basal ganglion | *R* = 0.736,  *P* = 0.264 | *R* = -0.08,  *P* = 0.92 | *R* = 0.054,  *P* = 0.966 | *R* = -0.995,  *P* = 0.005 | *R* = 0.53,  *P* = 0.47 | *R* = 0.319,  *P* = 0.793 |
| SUVmax of right basal ganglion | *R* = -0.623,  *P* = 0.377 | *R* = 0.083,  *P* = 0.917 | *R* = -0.095,  *P* = 0.94 | *R* = -0.985,  *P* = 0.015 | *R* = 0.386,  *P* = 0.614 | *R* = 0.28,  *P* = 0.82 |
| SUVmax of left thalamus | *R* = -0.699,  *P* = 0.301 | *R* = -0.276,  *P* = 0.724 | *R* = -0.106,  *P* = 0.932 | *R* = -0.995,  *P* = 0.005 | *R* = 0.567,  *P* = 0.433 | *R* = 0.292,  *P* = 0.811 |
| SUVmax of right thalamus | *R* = -0.770,  *P* = 0.230 | *R* = -0.408,  *P* = 0.592 | *R* = 0.006,  *P* = 0.996 | *R* = -0.993,  *P* = 0.007 | *R* = 0.553,  *P* = 0.447 | *R* = 0.34,  *P* = 0.779 |

Footnote: *R* values represent Spearman’s correlation coefficients. *P* values are two‑sided, and *P* <0.05 indicates statistical significance. SUVmax = maximum standardized uptake value; CSF = cerebrospinal fluid; TMEM9 = transmembrane protein 9; ATP6AP1 = ATPase H⁺transporting accessory protein 1; p‑tau217 = phosphorylated tau at threonine 217; p‑tau181 = phosphorylated tau at threonine 181.
